# Signatures of Radiation‐Induced Stress and Putative Selection on Immune Targets in Chornobyl Wolves

**DOI:** 10.1111/mec.70308

**Published:** 2026-04-28

**Authors:** Cara N. Love, Stacey L. Lance, Thomas G. Hinton, Nicolas Rochette, James C. Beasley, Dmitry Shamovich, Michael E. Byrne, Brian Nadel, Sarah C. Webster, Shane C. Campbell‐Staton

**Affiliations:** ^1^ Department of Ecology and Evolutionary Biology Princeton University Princeton New Jersey USA; ^2^ Odum School University of Georgia Athens Georgia USA; ^3^ Savannah River Ecology Lab University of Georgia Aiken South Carolina USA; ^4^ Centre for Environmental Radioactivity Norwegian University of Life Sciences (NMBU) Ås Norway; ^5^ Institute of Environmental Radioactivity Fukushima University Fukushima Japan; ^6^ Warnell School of Forestry & Natural Resources Athens Georgia USA; ^7^ Sosnovy Bor Vitebsk Region Belarus; ^8^ School of Natural Resources University of Missouri Columbia Missouri USA; ^9^ Institute of Genomics and Proteomics University of California, Los Angeles Los Angeles California USA

**Keywords:** adaptation, Chornobyl, immune modulation, radiation

## Abstract

Investigating the physiological and evolutionary consequences of contaminant exposure in wild populations is critical for understanding long‐term ecological impacts of anthropogenic change. However, how and why species persist, even thrive, in highly contaminated regions in the absence of humans remains a topic of much debate. We examined the regulatory and genomic impacts of multigenerational chronic radiation exposure to grey wolves ( *Canis lupus* ) within the Chornobyl Exclusion Zone. Wolves within the exclusion zone are at an estimated seven times greater density than surrounding preserves, despite lack of physical barriers to dispersal and chronic exposure to elevated radiation dose. Demographic analyses of genetic variation and home range modelling further suggest that ecological factors may support the wolf population within the exclusion zone. Wolves within Chornobyl exhibit altered leukocyte composition and regulatory signatures within the blood transcriptome that support significant alterations to metabolic and immune response pathways, particularly those influential in DNA damage response indicating radiation‐induced immune modulation. Selection scans across genes within the blood transcriptome revealed multiple regions of accelerated Chornobyl‐specific divergence at loci with known roles in immunity and response to oncogenesis. Together, these data provide evidence that chronic exposure to ionising radiation may be a significant source of ongoing natural selection in an apex predator after a single contamination event, highlighting multigenerational impacts beyond initial exposure. Further, these results highlight the potential contributions of natural selection to species persistence and proliferation in highly contaminated ecosystems.

*Chornobyl wolves experiencing multigenerational radiation‐stress show evidence of selection on immune targets*.

## 1 Introduction

On April 26, 1986, an uncontrolled nuclear fission chain reaction in the No. 4 reactor of the Chornobyl Nuclear Power Plant resulted in one of the most severe nuclear accidents in human history. This event released an estimated ~6–7 tons of radionuclides by mass into the environment and resulted in persistent contamination across much of Europe and the former Soviet Union (IAEA 2006; United Nations 2000, 2008; OECD, Nuclear Energy Agency 2003; UK Parliament n.d.). Human inhabitants were evacuated from the most highly contaminated region surrounding the reactor site in an effort to protect human populations from potentially deleterious effects of exposure to ionising radiation (Ward 1988). However, a wide range of native wildlife has colonised, persisted and expanded within the Chornobyl Exclusion Zone (CEZ) over the subsequent decades since the incident (Nikiforov et al. 2022; Schlichting et al. 2019; Deryabina et al. 2015; Webster et al. 2016).

Studies of diverse taxa living within the CEZ have revealed variable genetic, physiological and pathological effects of chronic exposure to ionising radiation (e.g., Galván et al. 2014; Burraco and Orizaola 2022; Kesäniemi et al. 2019; Spatola et al. 2023). Such exposure can cause DNA damage, either indirectly by producing reactive species and resulting in oxidative damage, or as a direct result of DNA breaks via ionising atoms (Ward 1988; Einor et al. 2016). DNA damage can lead to genomic mutations and/or uncontrolled cell growth, which increases genetic disease and oncogenic risk (Basu 2018; Lumniczky et al. 2021). Consistant with this, previous studies have reported increased oncogenic occurrence in humans exposed to increased radiation from Chornobyl (Drozd et al. 2021; Rivkind et al. 2020). Although oncogenesis is more difficult to measure in free ranging wildlife, some studies have reported genetic abnormalities among Chornobyl biota such as elevated rates of DNA damage (Bonisoli‐Alquati et al. 2010; Morton et al. 2021), altered mutation rates (Møller and Mousseau 2015) and signs of abnormal thyroid activity (Dubovyi et al. 2021). Variation in some traits across radiation gradients in Chornobyl suggests the potential for radiation‐induced selection in the region (Galván et al. 2014; Burraco and Orizaola 2022; Kovalchuk et al. 2004; Ruiz‐González et al. 2016). Previous studies have described possible beneficial genomic characteristics with higher heterochromatin constituent copy numbers (18S rDNA and Msat‐160 satellite) in bank voles, *Myodes glareolus* , from highly contaminated versus less contaminated areas of the CEZ (Jernfors et al. 2021), potentially conferring increased DNA damage protection (Qiu 2015). In laboratory experiments, fibroblasts from bank voles from the most highly contaminated regions of the CEZ recovered from gamma radiation dose (10 Gy) faster than those from a reference region outside the CEZ (Mustonen et al. 2018). However, the severity of effects on physiology, gene mutation, DNA damage and immune response have ranged from undetectable (e.g., Burraco et al. 2021; Goodman et al. 2019; Meeks et al. 2009; Dillon et al. 2024) to significant (e.g., Galván et al. 2014; Burraco and Orizaola 2022; Kesäniemi et al. 2019), depending on experimental context, study organism and limitations in methods of estimating radiation dose (Beaugelin‐Seiller et al. 2020; Bontrager et al. 2024). As a result, the long‐term biological implications of chronic radiation exposure for resident wildlife remains unclear (Beresford and Copplestone 2011; Smith 2008).

Examining immunological responses to chronic environmental radiation exposure, such as that observed in the CEZ, is essential for understanding the long‐term biological impacts of radiation on organismal physiology, performance and fitness in the wild. Radiation is known to have diverse systemic effects on immunological function. Acute high‐dose laboratory exposure can have direct immunotoxic effects such as lymphopenia (Nakamura et al. 1990) while also stimulating molecular machinery responsible for DNA damage response and repair, much of which is regulated by immune mechanisms (Nastasi et al. 2020). Chronic radiation exposure differs significantly from acute, high‐dose radiation exposure, as it generally occurs at lower doses and over extended time periods, potentially modulating immune system function in more subtle and complex ways (Lumniczky et al. 2021). Low‐dose radiation exposure may lead to permanent impairments in immune function by accelerating immune senescence, thereby increasing susceptibility to a broad range of radiation‐induced pathologies including early‐onset age‐related symptoms and cancer (Lumniczky et al. 2021). The relationship between radiation exposure and immunological responses can be linear, as seen with lower blood cell counts in radiation‐dosed macaques ( *Macaca fuscata* ; Ochiai et al. 2014), but clinical studies in humans and cross‐species comparisons within field and lab studies demonstrate effects are often more dynamic and non‐linear (Lumniczky et al. 2021; Tovstuha et al. 2022). In particular, ionising radiation, such as that widely permeating in the CEZ ecosystem, induces DNA lesions such as double and single strand breaks, primarily through the generation of free radicals (Santivasi and Xia 2014; Ward 1988). These radicals attack DNA, proteins and lipids, leading to direct and indirect damage, which in turn can lead to significant biological consequences, including cell death, mutations and cancer. Radiation‐induced DNA damage activates multiple repair pathways, which frequently interact with immune cells recruited to the affected sites, resulting in either immunostimulatory or immunosuppressive outcomes (Wang et al. 2022). These responses are frequently characterised by modulated chemokine and cytokine regulation, oxidative stress responses and chronic inflammation (Lumniczky et al. 2021). Studies of human cohorts, atomic bomb survivors, medical models and CEZ wildlife describe consistent shifts in immune parameters between exposed and unexposed individuals (Lumniczky et al. 2021; Ochiai et al. 2014; Kusunoki et al. 2010). With research on biodiversity within the CEZ collectively highlighting complex relationships between radiation exposure and haematological responses (Lumniczky et al. 2021; Tovstuha et al. 2022; Charruau et al. 2016; Bauer et al. 2018; Lafferty and Holt 2003). These studies suggest that increased radiation exposure interacts with immune function in ways that may accelerate immune senescence, modulate immune phenotypes and elevate risk of age‐related degenerative disorders and cancer immune responses. Collectively, these findings highlight immunological mechanisms as a key mediator of radiation stress and critical mechanisms for plastic physiological response to combat detrimental impacts of increased radiation dose (Lumniczky et al. 2021; Nastasi et al. 2020).

Research on chronic radiation exposure and immune regualroty and genomic alterations may reveal novel insights into how radiation affects immune homeostasis, inflammation pathways and susceptibility to infections, oncogenesis and/or autoimmune diseases (Lumniczky et al. 2021). Insights from such studies can inform our understanding of potential risks to human populations living in areas of elevated radiation and help refine safety standards for occupational and environmental exposure. Moreover, studying the immunological effects of chronic radiation in Chornobyl's unique ecosystem provides an unparalleled opportunity to explore resilience and resistance mechanisms in both humans and wildlife.

Previous studies have shown that human‐mediated environmental contaminant exposure can contribute to fine‐scale local adaptation, as seen in some urban and aquatic landscapes (Reid et al. 2016; Salmón et al. 2021; Whitehead et al. 2017). If ionising radiation is a significant source of biological stress across multiple generations, selection on standing genetic variation may produce adaptive responses that more efficiently mitigate maladaptive physiological effects, offering potential models for radiation exposure treatments via enhancement of radiation tolerance. Such research also has implications for public health policies, especially in regions prone to nuclear accidents or those considering nuclear energy expansion. However, the extent to which ionising radiation serves as a significant source of selection in wild populations occupying regions of high radionuclide contamination is largely unknown, though some studies suggest a potential role of local adaptation in the region (Burraco and Orizaola 2022; Car et al. 2023; Dillon et al. 2023).

Mammals are among the most radiosensitive taxa (Whicker and Schultz 1982) and long‐lived mammals are particularly sensitive due to bioaccumulation of high internal levels of radioactivity over an organism's lifespan (Higley et al. 2003). As a result, these species may also be disproportionately impacted by the accumulation of genotoxic and immune modulatory effects of chronic radiation exposure. Among mammals, grey wolves ( *Canis lupus* ) are an intriguing model for studying the effects of radiation exposure on wildlife as they are longer lived than many previously studied species in Chornobyl (living up to 7–8 years) and have experienced chronic radiation exposure for ~7 generations (or 30 years after the reactor exploded; Mech et al. 2016) through environmental and bioaccumulated exposure (Hinton et al. 2019). However, despite the potential physiological and genomic stress of increased radiation exposure, grey wolves have been shown to have 7× higher population density than surrounding preserves (Deryabina et al. 2015). We hypothesised that chronic multigenerational exposure may be a significant source of natural selection targeting aspects of the mammalian immune response and DNA damage repair within the CEZ population. By examining regulatory and genetic variation among grey wolves occupying the CEZ, we searched for molecular signatures of radiation‐induced stress and site‐specific selection associated with multigenerational chronic exposure. Towards this end, we compared regulatory and genomic variation within blood transcriptomes of CEZ wolves to those from a geographically proximate control site in Northern Belarus (BLR) with background levels of radiation (Izrael and Bogdevich 2009) to examine regulatory plasticity and genetic evidence of selection within the CEZ. Additionally, we utilised previously published blood transcriptome data from Yellowstone National Park, USA (YLS) wolf population (Charruau et al. 2016) as an outgroup to control for possible confounding factors such as geography, demography and environment, to quantify Chornobyl‐specific patterns of expression diverging between two geographically proximate and admixed groups (CEZ and BLR).

## 2 Materials and Methods

### 2.1 Study Site Description

The Chornobyl nuclear reactor exploded in 1986, releasing an estimated ~6–7 tons of radionuclides by mass into the atmosphere, causing high contamination levels in local villages and surrounding ecosystems (IAEA 2006; United Nations 2000, 2008; OECD, Nuclear Energy Agency 2003; UK Parliament n.d.). Much of the radiation settled over Eastern and Central Europe, with the highest concentrations falling in what is now northern Ukraine and southern Belarus (IAEA 2006). To mitigate the impacts of the subsequent irradiation in the CEZ, a 4762 km^2^ exclusion zone was established with more than 200,000 people evacuated from the most highly contaminated regions. The Polesye State Radiation Ecological Reserve (PSRER) serves as the managing entity of the largest portion of the CEZ, found on the Belarussian side, and hosts wide spatial heterogeneity in radionuclide contamination distribution (soil contamination levels of 40–> 7000 kBq/m^2 137^Cs). This contaminant gradient, a diverse mammal community (Schlichting et al. 2019; Webster et al. 2016, Nikiforov et al. 2022), lack of human activity in the CEZ, and extensive hunting in the surrounding landscape provide a unique study system to investigate the long‐term implications of sentinel species living in a radioactively contaminated landscape.

### 2.2 Sample Collection and Demography

We captured wolves across the contamination gradient in the PSRER during November 2014 (*N* = 9) and November 2016 (*N* = 2) with a humane non‐lethal capture technique utilising modified foothold traps. Descriptive measurements and sample collection were performed including radiological internal dose measurements (Hinton et al. 2019) and aging based on tooth‐wear (Gipson et al. 2014). We implemented all animal capture and handling in accordance with University of Georgia Animal Care and Use protocol A2015 05‐004‐Y2‐A1 and A2012 08‐044. We collected blood samples from the CEZ wolves, immediately transferred samples to RNAlater for stabilisation of cellular RNA, and stored samples at −20°C until transferred to the University of Georgia's Savannah River Ecology Lab, USA, where they were stored at −80°C until RNA isolation occurred. We did not observe obvious signs of malnourishment or apparent phenotypic abnormalities when processing each wolf.

All CEZ wolves from 2014 were utilised for all transcriptome profiling given the robust radiation dose estimates collected for these individuals, and to limit confounding environmental variables contributing to individual variation in expression across sampling years. When identifying putative genes under selection and population structure analyses, we included the wolves from both 2014 and 2016 to increase confidence in characterising genomic variability within and specific to the CEZ wolves.

We collected grey wolf whole blood reference samples from a region in Northern Belarus (*N* = 9; BLR) with background levels of radiation (Izrael and Bogdevich 2009) and from previously published data from Yellowstone National Park, North America (*N* = 27; YLS; Figure S5; Charruau et al. 2016). To obtain samples from Northern Belarus, also collected in 2014–2016, we collaborated with regionally permitted hunting organisations within < 400 km from the PSRER (Figure S1). This range falls well within the regularly documented dispersal distance of grey wolves (Byrne et al. 2018; Kojola et al. 2009),7 including direct documentation of a young wolf that travelled at least 369 km from its natal range in the CEZ (Byrne et al. 2018). Blood samples were collected immediately, and we processed them in the same manner described above for CEZ wolves. We also collected muscle and liver tissue samples from these animals and stored them at −20°C for later ^137^Cs quantification. No individuals were killed for the purposes of this research. All BLR samples were from sites characterised by limited human activity, mosaics of mixed hardwood and coniferous forests, with freshwater systems dispersed throughout, and exhibiting habitat similar to what is found in the PSRER.

Wolf generation times are not well documented from the region of Chornobyl, so for the purposes of estimating generations post reactor explosion, we extrapolated generation times from other regions of Europe and North America (Mech et al. 2016; Mergeay et al. 2024; Vonholdt et al. 2008; Hedrick et al. 2014). More precise estimates of generation times have been derived from North American populations, including generation time of 4.16 years in Yellowstone National Park (Vonholdt et al. 2008), 4.00 years in Isle Royale (Hedrick et al. 2014), 4.2–4.7 years in Superior National Forest and Minnesota, USA (Mech et al. 2016) and 5.0 years in Germany (Mergeay et al. 2024). Given the limited regional estimates, we extrapolate our generation time from average generation time across Europe and north American (4.37) to estimate generation time since the Chornobyl reactor exploded.

### 2.3 Home Range Estimation, Dose Rates and Life‐Time Dose From ^137^Cs and ^90^Sr

We quantified radiation dose rates caused from sources external to the CEZ wolves with GPS collars (Vectronic Aerospace GmbH, Berlin, Germany) equipped with an integrated electronic dosimeter (Mirion Technologies; Hinton et al. 2015) attached to each animal at the time of capture (Hinton et al. 2019). Collars were programmed to collect a GPS location and radiation exposure reading every 35 min and transmit data remotely through the Globalstar satellite communication network, with an automatic drop‐off mechanism set to release 1 May 2015. We used autocorrelated kernel density estimation (AKDE; Kolberg et al. 2020) to estimate utilisation distributions (UD) and delineate home ranges (95% UD contour) for each GPS‐collared wolf (*N* = 8, 6 females and 2 males) using the ctmm package (Georgakilas et al. 2015) in R (R Core Team 2022). One wolf, a young male, engaged in long‐distance dispersal movements beyond the CEZ in February 2015 (Smith 2008). As such, we estimated a home range for this wolf based on movement data collected prior to dispersal, when the wolf was resident in the CEZ. Wolves were tracked from 165 to 180 days, resulting in ~6600 individual locations and ^137^Cs external dose rates per wolf (Hinton et al. 2019). The average external dose rate (μGy/h) for each wolf was used as a component in their total dose rate (Table S1). We also measured each animal's dose rate caused by internal ^137^Cs contamination levels (Bq/kg; Table S1). As described in Hinton et al. (2019) a calibrated 1‐cm^3^ Cadmium–Zinc–Telluride (CZT) radiation detector system, operated by a portable computer, was placed under the animal's flank muscle, whereas the animal was anaesthetised, to quantify activity concentrations (Bq/kg) of ^137^Cs in each animal. A ^137^Cs dose coefficient of 2.7e‐4 was obtained from ICRP (2017), based on an average wolf mass of 35 kg, and used to convert internal ^137^Cs activity concentrations to internal dose rates (μGy/h; Table S1). Lifetime dose estimates are calculated by multiplying dose rate calculation for each individual by individual age (Supporting Information).

For wolves collected in BLR, we quantified internal ^137^Cs concentrations from lyophilised, homogenised muscle tissues. Laboratory analyses of ^137^Cs were performed using a Packard Cobra II auto‐gamma counter (Model Cobra II 5003; Packard Instruments Co., Meriden, CT, USA). Dry activity concentrations (Bq/g, dry weight) were then converted to wet activity concentrations (Bq/g, wet mass) using wet: dry tissue mass ratios. We used the same ^137^Cs internal dose coefficient used for the Chornobyl wolves on animals from BLR to convert internal activity concentrations to dose rates (μGy/h). External dose rates for BLR wolves were derived from ^137^Cs soil contamination maps for northern Belarus (Guermentchuk et al. 1997) using an external dose coefficient of 1.1e‐4 (Ulanovsky et al. 2017). Previous work on these ^137^Cs dose rate estimation techniques with the methods utilised in CEZ wolves have shown good agreement in estimated dose (Hinton et al. 2019; Supporting Information).

We estimated ^90^Sr internal dose rates from the area‐weighted ^90^Sr soil contamination within each CEZ wolf's GPS‐determined home range. Using soil contamination levels specific to each animal's home range results in a strong correlation to internal contamination levels (Hinton et al. 2019). We implemented a ^90^Sr concentration ratio of 0.86 (International Atomic Energy Agency 2010) converted soil contamination to animal tissue activity concentrations, and a dose coefficient of 6.38e‐4 (Ulanovsky et al. 2017) to derive internal dose rates (μGy/h) for each individual CEZ wolf. External dose from ^90^Sr in BLR wolves was not considered because its dose coefficient is 7.6e‐12 (Ulanovsky et al. 2017), eight orders of magnitude less than that for ^137^Cs. Likewise, Sr. deposition in areas where BLR wolves were collected was not a factor in their background dose rates (Supporting Information).

### 2.4 RNA‐Sequencing and Transcriptome Processing

We purified total RNA from blood samples stabilised in RNAlater using RiboPure Blood Kit (Life Technologies 2011). We sequenced whole blood transcriptomes from all wolves with Illumina NGS Stranded RNA library preparation and 2 × 75 bp paired‐end sequencing performed on an Illumina NextSeq platform. All samples were run across four lanes. We surveyed the quality of all reads using FastQC (v0.11.8, default parameters; Andrews 2010) and filtered reads using Trimmomatic (v0.38, ILLUMINACLIP LEADING:20 TRAILING:20 SLIDINGWINDOW:5:20 MINLEN: [80%]; Bolger et al. 2014) to remove low quality reads and adapters. We then mapped quality‐controlled reads to the CanFam3.1 (Hoeppner et al. 2014) genome using a two‐pass approach in STAR (v.2.7.1a, –outFilterMultimapNmax 1 –outFilterMismatchNmax [10%] –outSJfilterCountTotalMin 10 5 5 5 –outSJfilterCountUniqueMin 10 5 5 5; Dobin et al. 2013) and quantified read counts with htseq‐counts (v0.12.4; Anders et al. 2015). We further confirmed lack of bacterial contamination using Kraken (Wood and Salzberg 2014) and excluded haemoglobin genes for all downstream analyses. Previously collected RNAseq data from Asia and North America were processed and filtered in the same manner as described above.

### 2.5 Blood Cell Type Deconvolution

To infer cell type composition for each sample, we mapped wolf genes to their one–one orthologs in *Mus musculus* using the Ensembl BioMart (v98/sep2019; Cunningham et al. 2022) and examined expression levels with GEDIT v3.28 (Nadel et al. 2020) based on CPM‐normalised expression levels, as recommended by the authors of the tool. We utilised the reference cell type profiles derived from the Tabula Muris cell atlas (Schaum et al. 2018) provided with the tool, and otherwise default settings.

### 2.6 Population Structure Characterisation

To quantify patterns of genomic admixture across the CEZ boundary with respect to wider geographic patterns, we analysed genetic variation and initial biogeographical ancestry analysis (Alexander et al. 2009) demonstrates distinct structure between the Asian (Inner Mongolia (*N* = 4; Liu et al. 2017) and Tibet (*N* = 4; Liu et al. 2017)), North American (YLS; *N* = 27; Goodman et al. 2019) and European (BLR (*N* = 9) and CEZ (*N* = 11)) wolf populations. We mapped filtered RNAseq reads to the CanFam3.1 genome (Hoeppner et al. 2014) utilising the Broad Institute recommended two‐pass approach with STAR (v.2.7.1a; Dobin et al. 2013). We called SNPs with GATK (McKenna et al. 2010) HaplotypeCaller and GenotypeGVCF using the recommended RNAseq parameters and removed PCR duplicates with the MarkDuplicates function. We selected autosomal genotypes in PLINK (v1.90b.3.45; Purcell et al. 2007) and utilised VCFtools (v0.1.14; Danecek et al. 2011) to select genotypes with depth (DP) > 8, genotype quality (GQ) > 20, biallelic SNPs with < 20% missing data. This resulted in 27,017 SNPs (Appendix S1).

To prepare data for admixture analyses we first filtered SnpEff v5.1d (Cingolani et al. 2012) annotated SNPs for ‘neutral’ (synonymous) SNPs using the CanFam3.1 genome SnpSift (Cingolani et al. 2012), to help isolate ‘silent variants’. Filtering for ‘silent’ variants resulted in 7388 SNPs. We further filtered for linkage disequilibrium by applying recommended (Alexander et al. 2009) filtering parameters (–indep‐pairwise 50 10 0.1) in PLINK (Purcell et al. 2007). The remaining 1539 SNPs where used to implement unsupervised clustering with ADMIXTURE (v1.3.0‐0; Alexander et al. 2009) across all populations with default criterion and run ADMIXTURE for *K* 2–6. We identified an optimal *K* of three utilising the 5‐fold cross‐validation method in ADMIXTURE (Figure S4). We additionally assess population structure by performing principal components analysis of allelic variation utilising the *‐pca* function in PLINK (Purcell et al. 2007) on all populations. We further assessed ‘silent’ SNP filtering versus whole dataset impacts on structure inference, as well as phylogenetic relationship, and gene flow in more detail within the Supporting Information.

To further assess fine‐scale genetic structure between the CEZ and the most geographically proximate population (BLR), which may have remained undetected in the larger analyses, we performed principal components analysis of allelic variation and examined sub‐structure admixture between CEZ wolves and the most geographically proximate population, BLR, as described above (Appendix S1 and Figures S4–S7).

### 2.7 Characterisation of Co‐Expression Modules and Radiation Correlated Expression

For characterisation of co‐expression modules, we performed WGCNA, using the WGCNA package (Langfelder and Horvath 2008), and wolves from CEZ (2014 individuals only, *N* = 9), BLR (*N* = 9) and YLS (*N* = 27). We filtered transcripts for average read count > 10 and log transformed *cpm* normalised (edgeR; Robinson et al. 2009) read counts. We then utilised mean pairwise correlations between samples to identify outlier samples (Figure S1A) which were then removed from subsequent analyses. Utilising a soft thresholding approach, we approximated a scale free topological network to compare an adjacency matrix and utilised the power of 15 to construct co‐expression modules (Figure S1B). We then performed topological overlap to create a cluster dendrogram with signed Pearson correlations while implementing a minimum cluster size of 40 genes and merging closely correlated modules (*R* ^2^ = 0.95, Figure S1B). We utilised student asymptotic *p*‐value for correlation using WGCNA's *corPvalueStudent* function to examine trait: module relationships (Appendix S1).

To characterise divergent module expression patterns between populations we used linear models using the *lm* command from *stats* package (R Core Team 2024). We performed these models on module eigengene values and utilised population (Europe and North America) to better contextualise transcriptomic variation within a broader evolutionary framework. We then examined module eigengene values across radiation condition within the European population (Low Dose [BLR] and Elevated Dose [CEZ]) due to their greater ecological, geographic and genetic similarity. The North American population was included as an outgroup due to its genetic distinctiveness from the CEZ and BLR populations, allowing us to contextualise transcriptomic variation within a wider evolutionary context. Specifically, it provides a reference point for assessing whether observed expression patterns in CEZ and BLR are likely to be driven by radiation exposure or reflect deeper lineage‐level divergence.

To more explicitly evaluate whether radiation dose rate significantly influences module expression patterns, we fit generalised linear mixed models (GLMMs) with the *lmer* command from *lme4* package (Bates et al. 2015) using total ^90^Sr & ^137^Cs (μGy/h) dose rate data exclusively from the CEZ. We fit two models using a the full model including total dose rate, age and relative monocyte and granulocyte proportions as fixed effects, with sex included as a random intercept, and a reduced model excluding dose rate or dose rate and age. The two models were then compared using the *anova()* function to test for significant improvement in model fit. Once modules of interest were identified, we characterised gene ontology categories with gprofiler2 (v0.2.2; Kolberg et al. 2020), using the *clfamiliaris* reference, implementing an unordered query while filtering for only significant results (corrected *p* < 0.05) using FDR correction for multiple tests, and a custom background of the wolf blood transcriptome data and identified driver terms (Supporting Information).

We further examined transcriptome patterns associated with individual dose within the CEZ wolf population, by examining transcript specific correlation with total ^137^Cs and ^90^Sr dose rates. Specifically, we calculated transcript‐level correlation coefficients and identified genes falling within the top 0.95 quantile of correlation distribution (Spearman's *ρ*; *p* < 0.05) by characterising gene significance (*GS*_*i*_; Langfelder and Horvath 2008) with *cor* and *corPvalueStudent* as described in WGCNA (Langfelder and Horvath 2008). This gene subset represents the most strongly dose‐associated transcripts and facilitates candidate gene and pathway prioritisation. We described gene ontology terms of this gene set using gprofiler2 (v0.2.2; Kolberg et al. 2020) while implementing an unordered query with the *clfamiliaris* reference and calculated significance with FDR correction (Supporting Information).

A priori hypotheses suggest immune signalling and DNA repair pathways are critical in radiation response and would be modulated in elevated radiation environments. We examine KEGG pathway profiles in four pathways underlying radiation induced stress (Georgakilas et al. 2015). These pathways include immune signalling pathways: chemokine signalling pathway (KEGG: cfa04062) and cytokine‐cytokine signalling pathway (KEGG: cfa04062); and DNA damage repair pathways: mismatch repair pathway (KEGG: cfa03430) and base pair excision repair pathway (KEGG: cfa03410). Principal component analysis (PCA) of pathway expression was performed with the package *FactoMineR* in R (Lê et al. 2008) and plotted with 95% confidence ellipses using the function *stat_ellipse* in *ggplot2* (Wickham 2011).

### 2.8 Identification of Candidate Genomic Regions Under Selection

To identify putative genes under selection, we used QC and coverage filtered SNPs, as detailed above, to compute population genetic statistics (Weir and Cockerham *F*_ST_) using VCFtools. To test for signatures of selection specific to the CEZ wolf population, we used RNAseq data from BLR, CEZ and YLS and utilised Cavalli‐Sforza transformed *F*_ST_ in calculating population branch excess (PBE; Shpak et al. 2024). PBE was calculated for CEZ wolves at single nucleotide resolution. This multibranch statistic uses sister (BLR) and outgroup (YLS) lineages to polarise genetic divergence to a focal lineage (CEZ) after its separation from the other populations, accounting for the demographic relationships among the lineages being compared (Shpak et al. 2024) to isolate genomic regions displaying elevated rates of site‐specific divergence. PBE builds off of the population branch statistic (PBS), employing an expected PBS value at each locus and quantifying the degree to which PBS for the focal population exceeds that which is expected. This comparison allows PBE to more adequately account for complex demographic histories and more specifically identify lineage specific positive selection (Shpak et al. 2024).

Negative PBE values were set to 0 before *z*‐score normalising PBE values and assessing the empirical distribution to calculate *p*‐value with the *pnorm* function in R. Multiple test correction was applied using the *p.adjust* (method = fdr) command in the *stats* package in R. Our outlier threshold is based on the empirical distribution of the PBE statistic, where we used a total empirical distribution threshold cutoff of FDR‐corrected −log_10_(*p*) > 8 in identifying outlier SNPs. We then utilised a more conservative threshold (FDR‐corrected −log_10_(*p*) > 15) to identify extreme CEZ outliers which further minimises the chance of false positives among outlier candidate assessment. Genes with significant CEZ PBE values represent genes that have undergone population‐specific differentiation consistent with positive selection (Shpak et al. 2024; Yi et al. 2010; Appendix S1).

To identify gene ontology categories of all outlier SNPs we utilised gprofiler2 (Kolberg et al. 2020) in R by applying an unordered query, filtering for only significant results (corrected *p* < 0.05), and utilising a custom background consisting of all annotated SNPs from the wolf blood transcriptome. We further annotated SNP function with SnpEff v5.1d utilising the CanFam3.1 reference genome (Cingolani et al. 2012).

## 3 Results

### 3.1 Radiation Dose

**FIGURE 1:**
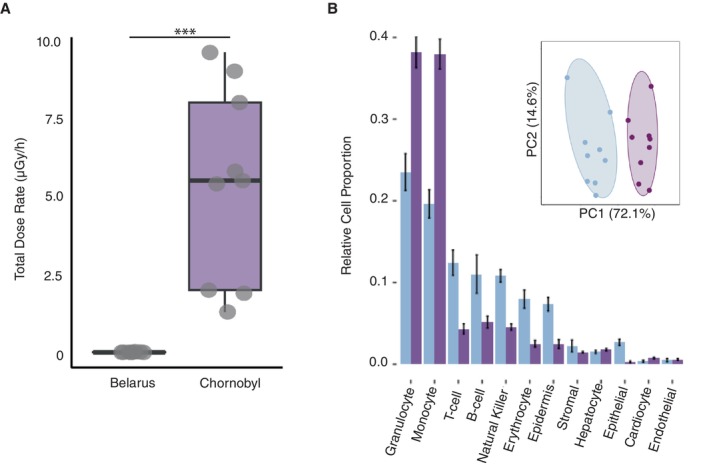
Total radiation dose and blood cell proportions. (A) Total (internal and external) ^137^Cs & ^90^Sr dose rate (μGy/h) of wolves from Northern Belarus (blue) and Chornobyl (dark purple). Boxes represent means and standard errors (SE) with standard deviations (SD) as whiskers. (B) Relative proportions of blood cell types identified in Northern Belarus and Chornobyl wolf populations. Insert depicts principal component (PCA) dispersion depicting total relative blood cell type abundances in wolves from Northern Belarus and Chornobyl. The percent of explained variance for PC1 and PC2 is 72.1% and 14.6%, respectively.

We characterised radiation dose in wolves from CEZ and a geographically proximate reference site outside the Chornobyl Exclusion Zone (BLR) and compared internal activity (Bq/kg) and total (internal and external) dose (μGy/h) (or yearly dose [mGy/y] extrapolated from μGy/h). We found significantly higher internal ^137^Cs activity (Bq/kg) and total (internal and external) ^137^Cs dose (μGy/h) in wolves from the CEZ than Belarus (BLR). The CEZ wolves experienced significantly elevated cumulative ^137^Cs and ^90^Sr dose rates (CEZ: 5.4 ± 3.1 μGy/h) compared to the BLR wolves (0.02 ± 0.01 μGy/h; *t*‐test: *t* = 5.16, df = 8.00, *p* = 8.65 × 10^−4^). We also estimated each wolf's lifetime dose (mGy) by multiplying individual age by the dose rate (μGy/h) (Supporting Information). Mean lifetime doses experienced by CEZ wolves were ~250 times greater than mean lifetime doses of their BLR counterparts (CEZ: 124 ± 93 mGy; BLR: 0.5 ± 0.3 mGy; *t*‐test: *t* = 3.97, df = 8.00, *p* = 4.10 × 10^−3^, Figure 1A). All ^90^Sr and total ^137^Cs and ^90^Sr dose estimates are reported in Table S1.

### 3.2 Altered Blood Cell Composition

Blood cell deconvolution results show the CEZ site differs significantly in several leukocyte cell type proportions when compared to those from BLR (per cell type Welch's two sample *t*‐test, *p* < 0.01, Figure 1B). The CEZ wolves exhibited significantly decreased lymphocyte proportions (Welch's two sample *t*‐test: T cells, *t* = −4.85, df = 10.53, *p* = 5.8e‐04; B cells, *t* = −2.39, df = 9.54, *p* = 0.039), and elevated granulocyte (Welch's two‐sample *t*‐test, *t* = 4.98, df = 15.54, *p* = 1.5e‐04) and monocyte proportions (Welch's two‐sample *t*‐test, *t* = 7.28, df = 15.94, *p* = 1.9e‐06) when compared to BLR wolves (Figure 1B).

### 3.3 Chornobyl Wolf Home Range and Population Structure

**FIGURE 2:**
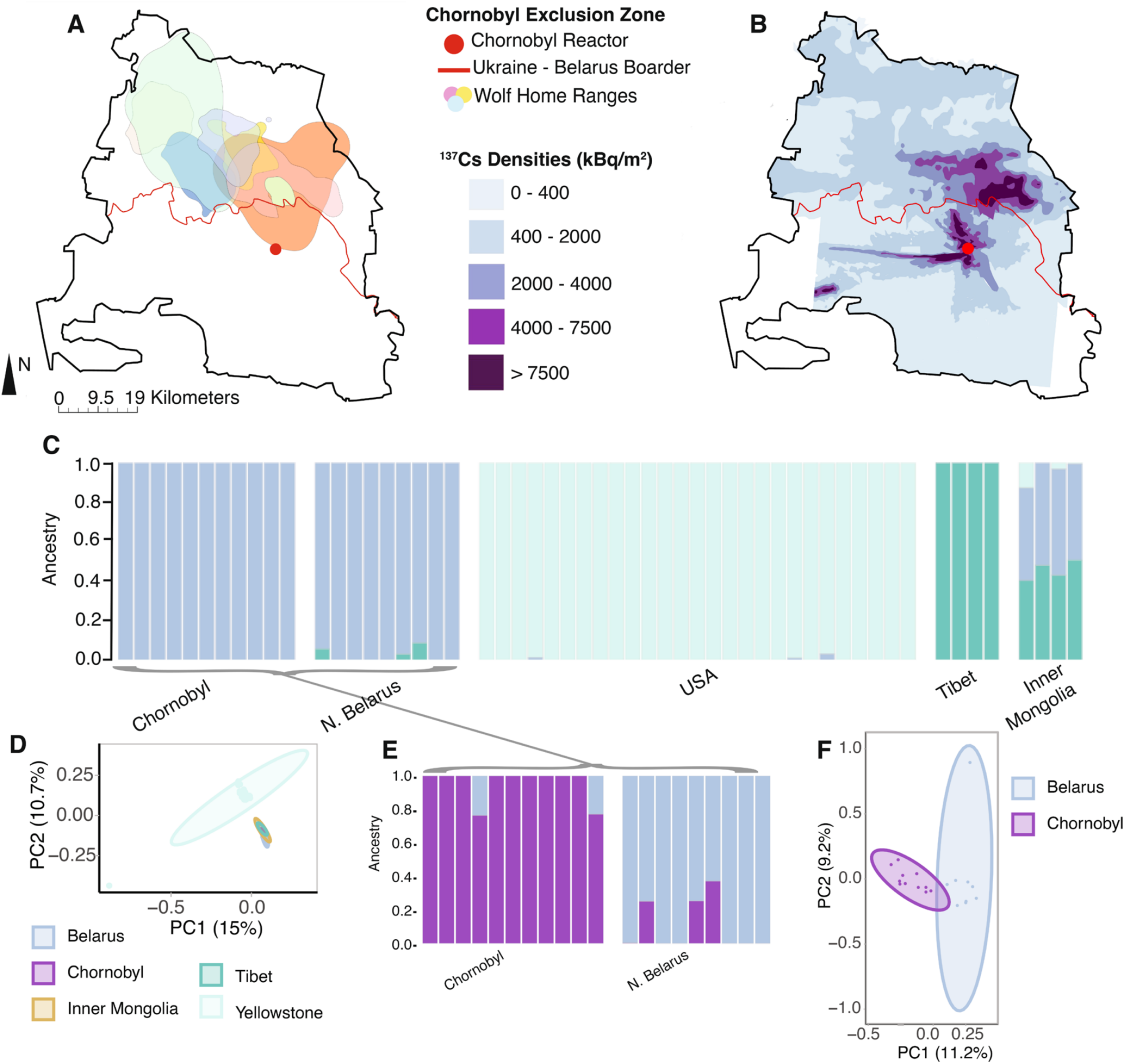
Chornobyl wolves reside within the highly contaminated exclusion zone and exhibit low rates of admixture with reference populations. (A) Home range distributions depicting 95% use distribution contours for eight grey wolves from Chornobyl (exclusion zone boundary outlined in black) overlap (B) with high levels of soil ^137^Cs densities (kBq/m^2^) within the Chornobyl exclusion zone. (C) Sparse nonnegative matrix factorization ancestry analysis (*K* = 3) of wolf populations across Asia, Europe and the North American (USA) utilising LD filtered synonymous loci (*N* = 1539). (D) PCA of genetic variability across all wolf groups. (E) Admixture between Chornobyl wolves and the closest geographic reference population in Northern Belarus in *K* = 2 (CV estimated *K* = 1) display a levels of local admixture between the two sites (examined at *K* = 2). (F) Fine‐scale structure analysis of Chornobyl wolves Northern Belarus suggests sub‐structure between the two populations, with dispersion of genetic diversity in principle component analysis (95% confidence ellipses) between the two populations.

Home ranges of 8 adult wolves tracked via GPS telemetry ranged from 28.1 to 597.8 km^2^ (mean = 254.7 km^2^) and were largely constrained within the CEZ boundary (Figure 2A,B). Initial genome‐wide ancestry analysis (Alexander et al. 2009) demonstrates distinct structure between the North American (YLS), European (BLR and CEZ) and Asian (Mongolia and Tibet) wolf populations (Figure 2C,D and Figure S3).

Subsequent analysis of fine‐scale population structure between CEZ and the most geographically proximate site (BLR) highlights low levels of population substructure across the boundary of the CEZ. ADMIXTURE cross‐validation analyses describe CEZ and BLR as one population (Figure S5), and when examining ADMIXTURE *k* = 2, CEZ and BLR wolves exhibit genomic ancestry from both sites (Figure 2E). Fine scale principal components analysis of allelic variation across BLR and CEZ wolves additionally highlights some level of genetic divergence between the BLR and CEZ sites (Figure 2F and Figure S6).

Examination of *f*‐statistics further support population differentiation across the North American, Europe and Asian populations (Table S2), and subsequent *qpgraph* an analyses suggest gene flow between BLR, CEZ, though proportion estimates vary by model (Figure S7 and Table S3).

### 3.4 Regulatory Divergence in Chornobyl

Weighted gene co‐expression network analysis (WGCNA; Langfelder and Horvath 2008) identified 13 co‐expression modules (Figures S2 and S3) within the blood transcriptome. Of these, ten modules were significantly associated with population‐of‐origin (North America vs. Europe; linear models, *p* < 0.001; Figure S3).

To investigate the effects of radiation exposure on module gene expression we first examined the significant main effect of radiation condition in low and elevated exposed wolves from BLR and CEZ, respectively, given their greater ecological, geographic and genetic similarity. We identified 11 of the 13 modules (linear models, *p* < 0.004, Figure S3), highlighting potential site‐specific systemic physiological consequences of the local environment and/or radiation stress in the CEZ in immune, metabolic and DNA repair pathways (Table S2).

**FIGURE 3:**
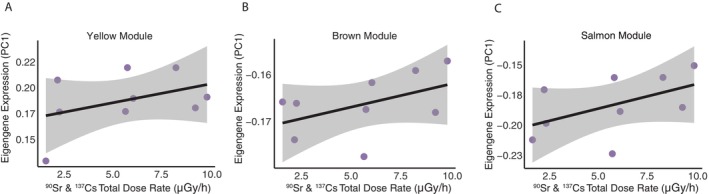
Identification of coregulatory modules associated with radiation exposure. Three of the 13 modules identified with weighted gene correlation network analysis (WGCNA) are uniquely significantly correlated with total ^137^Cs & ^90^Sr radiation dose rate in Chornobyl wolves.

**FIGURE 4:**
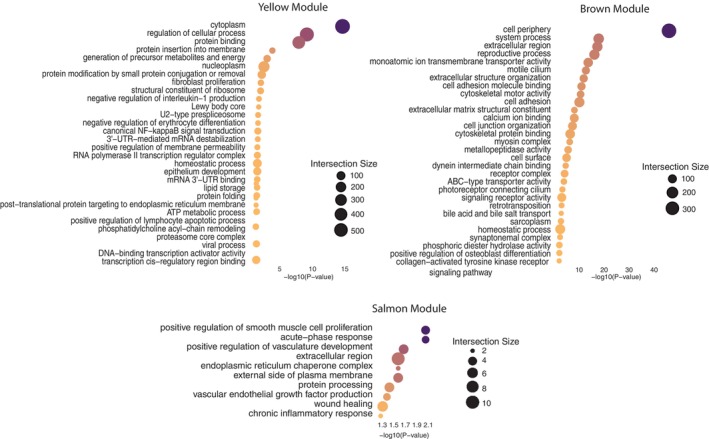
Gene ontology enrichment of modules displaying significant interactions with radiation dose. Gene ontology enrichment for the top 30 significant driver GO terms of three modules (Yellow, Brown and Salmon) displaying significant correlation with total dose rate, characterized with g:ProfileR2 and FDR corrected. Modules Yellow and Brown show a significant interaction with age and dose rate, whereas the salmon module displays a significant correlation with dose rate, as well as an interaction between dose and age.

**FIGURE 5:**
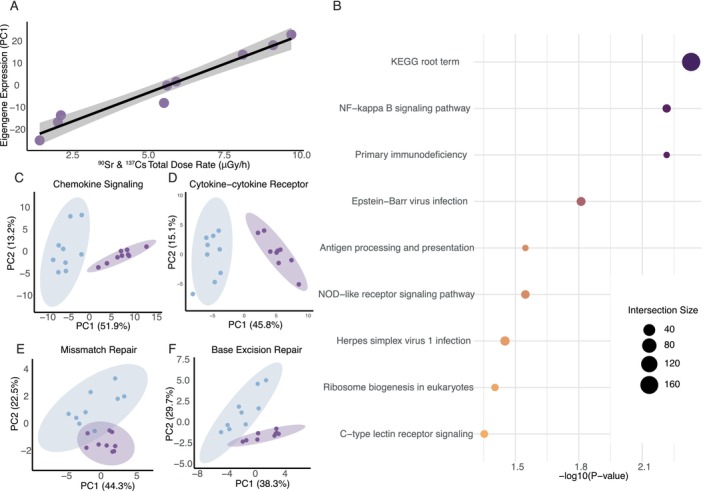
Gene expression significantly correlated with radiation dose: (A) PC1 of expression for the 359 genes in whole blood transcriptomes from Chornobyl wolves (N=9) correlated with total ^90^Sr and ^137^Cs dose rates (μGy/h). (B) Gene ontology enrichment for significant GO terms from total dose correlated genes, characterized with g:ProfileR2 and FDR corrected. (C–F) PCA of immune and DNA repair pathway expression in blood transcriptomes across Chornobyl (purple) and Belarus (blue) wolves.

Because radiation dose is highly correlated with site‐of‐origin, we utilised our individual level radiation dose measures to search for expression profiles displaying significant associations with individual variation in total (^137^Cs and ^90^Sr) dose rate (μGy/h) across the blood transcriptome only within the CEZ. Three modules (Salmon, Yellow and Brown) showed a significant influence of radiation dose rate with module expression within CEZ wolves (likelihood‐ratio test: *p* < 0.04; Figures 3, 4, 5 and Figure S1D). The Salmon module displayed a significant effect of dose rate on eigengene expression (likelihood‐ratio test: *χ* ^2^(1) = 5.5162, *p* = 4.471e‐07), as well as a significant interaction between dose and age (likelihood‐ratio test: *χ* ^2^(1) = 5.5162, *p* = 0.019). This module highlights inflammation and radiation induced damage response mechanisms including acute inflammatory response (GO:0002526, *p* = 0.0087), DNA damage repair mechanisms such as endoplasmic reticulum chaperone complex (GO:0034663, *p* = 0.03), acute‐phase response (GO:0006953, *p* = 0.009) and vasculature development (GO:1904018, *p* = 0.021; GO:1901342, *p* = 0.023). Interestingly this module also highlights processes important in wound healing and/or tumour growth (e.g., regulation of angiogenesis (GO:0045766, *p* = 0.021; GO:0045765, *p* = 0.085) and fever generation (GO:0001660, *p* = 0.009, GO:0031620, *p* = 0.045)) (Figures 3, 4, Figure S1D and Table S1).

The Yellow module also displayed a significant effect of dose (likelihood‐ratio test: *χ* ^2^(1) = 7.456, *p* = 0.006) and an interaction between dose and age (likelihood‐ratio test: *χ* ^2^(1) = 10.829, *p* = 0.001). This module highlighted a number of oncogenic specific pathways (GO:0002357; *p* = 0.047, KEGG:05200; *p* = 0.045) in particular leukaemia (KEGG:05166; *p* < 0.000, KEGG:05221; *p =* 0.001) and transcriptional misregulation in cancer (KEGG:05202; *p* = 0.008), as well as diver metabolic (e.g., GO:0006091; *p* = 0.001, GO:0019915; *p* = 0.026, GO:0046034; *p* = 0.030) and DNA repair mechanism pathways (e.g., GO:0007249; *p* = 0.02, GO:0061158; *p* = 0.020) (Figures 3, 4, Figure S2–S3 and Table S1).

Finally, the Brown module displayed a significant interaction between dose and age (*χ* ^2^(1) = 4.13, *p* = 0.042, Figures 3, 4 and Figure S1D) and highlights driver terms influencing cell development and stability (e.g., ion transport GO:0015075, *p* = 3.77E‐14, GO:0031402 = 0.020, cell–cell interactions, GO:0050839, *p* = 3.96E‐12; and cellular structure GO:0005201, *p* = 3.11E‐09, GO:0034330, *p* = 2.10E‐08) (Figures 3, 4, Figure S2–S3 and Table S1).

To gain a better understanding of radiation correlated expression, we further examined transcriptome wide correlation with radiation dose within the CEZ, revealing 359 genes with expression profiles highly correlated with total dose rate (top 0.95 quantile). Gene ontology enrichment of these radiation‐associated candidate genes revealed enrichment for immune regulatory and stress response pathways (Figure 5, Data S1), when compared to the entire gene complement of expressed genes within the grey wolf blood transcriptome dataset (*N* = 11,601 total blood transcripts). This gene set was enriched for immune modulatory pathways such as primary immunodeficiency (KEGG:05340, *p* = 0.005), antigen processing and presentation (KEGG:04612, *p* = 0.005), NOD‐like receptor signalling pathway (KEGG:04621, *p* = 0.024) and NF‐kappa B signalling pathway (KEGG:04064, *p* = 0.005), to name a few (Figure 5, Figure S1D and Table S2).

Finally, examination of known radiation modulated pathways indicative of immune signalling and DNA repair display distinct clustering in PCA space in wolves from Chornobyl and Belarus (Figure 5C–F).

### 3.5 Wolf Demographics

As age and sex demographics could be confounding variables influencing blood cell composition and gene expression patterns, we confirmed a lack of demographic differences within the CEZ and BLR individuals included in this study. There were no significant differences in age (*t*‐test: *t* = −1.1011, df = 12.686, *p* = 0.2913) or sex (chi‐square: *χ* ^2^ = 0, df = 1, *p* = 1.0) between the CEZ and BLR individuals.

### 3.6 Signatures of Selection on Immune Regulatory Genes

**FIGURE 6:**
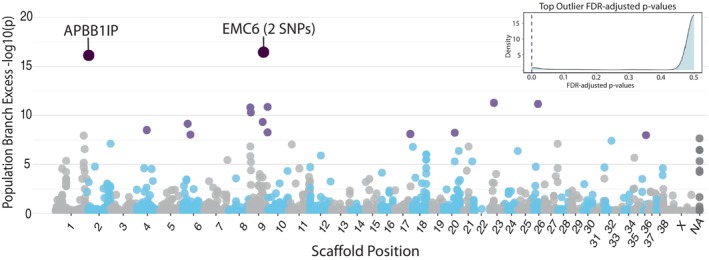
Signatures of genetic divergence within Chornobyl wolves: Manhattan plot of genetic divergence (population branch excess; PBE) within the Chornobyl wolf population after divergence from Belarus and Yellowstone National Park populations. Scaffolds are alternatively coloured grey and blue. The 17 SNPs exhibiting significant (PBE, −log_10_(*p*) > 8) divergence within the CEZ are highlighted in light purple. The three most differentiated SNPs (PBE, −log_10_(*p*) > 15) are highlighted in dark purple, with gene names. FDR corrected *p*‐values of top three outlier SNPs are highlighted by dashed bar within the insert.

Taking advantage of the lineage specificity allowed by PBE (Shpak et al. 2024), we identified regions of the wolf genome displaying significant genetic differentiation in CEZ wolves after their divergence from BLR and YLS (Figure 6). We identified 17 SNPs with significant divergence along the CEZ lineage (CEZ PBE, −log_10_(*p*) > 8; falling within 9 genes: APBB1IP, TRPV4, GGTA1P, CYTH1, EXOC7, PRKCB, CLTB, GGTA1P, DYNC1I2 and proximal to 6 genes: EMC6, CMTM6, CPD, RHOA, HIST2H2BE, LITAF). All nine SNPs found within annotated gene coordinates fell within exonic regions. The additional eight SNPs falling just outside annotated CanFam3.1 gene regions can still have regulatory consequences and were characterised by the most proximate annotation (distance to nearest annotation: mean = 769.38 ± 516.06 bp, median = 831 bp) for downstream analyses.

The 17 SNPs identified as CEZ‐specific outliers (PBE, FDR‐corrected −log_10_(*p*) > 6; Figure 6) were enriched for one driver term, cell cortex (GO:0005938: *p* = 0.014), and six KEGG pathways including Rap1 signalling pathway (KEGG:04015: *p* = 0.032), Wnt signalling pathway (KEGG:04310: *p* = 0.032) and parathyroid hormone synthesis, secretion and action pathway (KEGG:04928: *p* = 0.044) (Data S3), highlighting pathways influential in maintaining cell and tissue integrity, cell migration, division and differentiation and DNA damage response (Zhang and Yu 2023; Flormann et al. 2024; Zhang et al. 2017; Murray et al. 2005).

Identification of the most extreme outlier loci revealed three SNPs (CEZ PBE, FDR‐corrected −log_10_(*p*) > 12), one of which fell within the gene APBB1IP, and two of which fall just outside the annotated gene coordinates of EMC6. Annotation of these most extreme outlier loci reveals potential functional modifications to candidate genes. The APBB1IP outlier is located within exon 7 and is annotated as a missense variant, potentially altering APBB1IP protein structure, function, or stability. Both the EMC6 SNPs fall 64 and 65 bp, respectively, downstream of the annotated Ensembl CanFam3.1 EMC6 gene region, and 916–917 bp upstream from the gene TAX1BP3. Functional annotation of these loci reveals possible functional impact as downstream modifier loci to EMC6, upstream modifier variants of TAX1BP3, modifier intergenic loci for EMC6 and a novel gene ENSCAFG00000023152, or a downstream modifier of ENSCAFG00000023152.

## 4 Discussion

### 4.1 Chornobyl Wolves Experience Elevated Radiation Dose Rates

If local radiation exposure is a driving and persistent source of stress to the wolves within the CEZ, we would expect them to be exposed to significantly elevated radiation dose rates. To test this hypothesis, we quantified and compared radiation dose rates in wolves from the CEZ and a site outside the exclusion zone in northern Belarus (BLR), which is within the regularly documented dispersal capabilities of grey wolves (< 400 km; Kusunoki et al. 2010; Charruau et al. 2016). We characterise radiation dose in wolves from the CEZ and this geographically proximate reference site, by comparing internal ^137^Cs activity (Bq/kg) and total (internal and external) ^137^Cs and ^90^Sr dose (mG) in wolves from the CEZ and BLR. We found that CEZ wolves experienced significantly elevated cumulative dose rates compared to the BLR wolves, and CEZ lifetime mean dose (mGy) was ~250 times greater than mean lifetime doses of their BLR counterparts (Figure 1A).

Determining radiation dose rates to biota is challenging because of the myriad internal and external factors needing to be considered. Radiation doses to biota as a result of the Chornobyl accident occur through internal doses from the intake of ^90^Sr and ^137^Cs contaminated food, water and air. In addition, external doses occur when wildlife are irradiated from environmental components (e.g., soil, vegetation, litter) as they traverse their contaminated home ranges. Strontium‐90 emits a beta particle that can contribute to internal dose but lacks sufficient energy to be a major contributor to external dose. Caesium‐137 emits relatively high energy gamma photons that contribute to both internal and external dose. We quantified and compared radiation dose rates in wolves from the CEZ and BLR exposed to both pathways (internal + external) and both radionuclides at the individual level. Historically, dose is generally estimated at one time point and at the site of capture for an individual in Chornobyl; however, our dose estimates are built off of long‐term monitoring over many months, allowing for more robust individual level dose monitoring and total dose estimates. Although these dose estimates are arguably some of the most robust provided for a large mammal to date, wolf territories may shift over time, possibly resulting in temporal shifts in dose across a wolf's lifetime, and age estimation based on tooth wear can only be approximate. As such, these models may still under‐ or over‐estimate true cumulative chronic exposure histories to some degree. Additionally, our dose rate models work to harmonise the spatial scale for dose estimates between the CEZ and BLR individuals. However, the difference in methodological approaches necessitated in each location could influence precision of exact comparisons between the CEZ and BLR dose rate estimates.

In CEZ wolves, the mean dose rate of 5.4 μGy/h (= 47 mGy/y) is approximately 50 times greater than the recommended dose rate limit (1 mSv/y) for the general human populace (ICRP 2007; Kai et al. 2020), double the recommended dose rate for radiation workers (20 mSv/y ICRP limit; ICRP 1997) but less than the screening dose rate of 10 μGy/h currently thought protective of wildlife populations within an environmental risk framework (Andersson et al. 2009; Garnier‐Laplace et al. 2010). It is important to note, however, that wildlife‐specific thresholds are still highly debated due to a multitude of reasons, spanning the difficulties in accurately measuring dose in wild organisms to the struggle in disentangling the direct effects of radiation from the indirect effects of radiation contaminated environments such as larger ecological shifts (Bontrager et al. 2024; Barnthouse 1995; Sykes 2020).

We found that cumulative dose rates and estimated lifetime dose are significantly elevated in CEZ wolves. Although there is no established threshold of radiation dose for cancer risk, any increase in radiation dose comes with an incremental increase in oncogenic risk (Brenner et al. 2003; Nuclear Regulatory Commission n.d.). Taken together, these data suggest that radiation exposure may be a significant source of physiological stress for wolves within the CEZ.

### 4.2 Admixture Across the CEZ Boundary

Rapidly changing stress conditions across environmental gradients over short geographic distances can create conditions that favour fine‐scale local adaptation within a species or population (e.g., Whitehead et al. 2011; Combs et al. 2018), even in the face of gene flow (e.g., Salmón et al. 2021). Given the elevated radiation doses experienced by CEZ wolves, we hypothesised that chronic radiation exposure may act as a significant selective pressure, targeting aspects of the mammalian immune response and DNA damage repair in wolves inhabiting the CEZ. Under this hypothesis, ionising radiation may influence the health, survival and fitness of naïve wolves entering the CEZ, altering the rate of gene flow between the CEZ and surrounding sites and contributing to rapid local adaptation of the native CEZ wolves.

Given the multiple generations over which radiation has been elevated within the CEZ, we searched for evidence of population substructure associated with the local differences in ambient radiation. Initial admixture analysis (Alexander et al. 2009) demonstrates genomic structure between the North American (YLS) and Eurasian (Mongolia, Tibet, BLR and CEZ) wolf populations (Figure 2, Figures S4 and S5), and Eurasian wolves display limited admixture with Asian wolves (Figure 2C,D) as compared to the North American wolf population (Figures S4 and S5), supporting previously identified population structure patterns in the species (Pilot et al. 2019). CEZ and BLR sites are classified as one population, though closer examination of fine‐scale genetic divergence between the CEZ and BLR sites revealed a gradient of admixture across the boundary of the CEZ (Figure 2E) and post hoc principal components analysis of allelic variation between CEZ and BLR suggests some level of allelic segregation between the BLR and CEZ Eastern European sites (Figure 2F), potentially suggesting some level of local adaptation despite admixture with surrounding regions. Although admixture gradients are consistent with limited gene flow, these data are unable to directly estimate the direction or timing of migration. To further understand the dynamics of wolf movement within the CEZ, we used GPS telemetry data to quantify home ranges of 8 adult CEZ wolves. These data provide evidence that CEZ wolves are largely residing within the CEZ boundary and comparison of home range size across studies suggest CEZ wolf home ranges appear on the lower end of published home ranges for grey wolves, likely due to abundant prey resources, low anthropogenic disturbance, and elevated conspecific density within the CEZ (Mattisson et al. 2013; Vorel et al. 2024; Mancinelli et al. 2018; Lichwa‐Schneringer et al. 2025). Nearly all wolves tracked in our study were post‐dispersal age and thus not expected to disperse beyond their existing territories. However, our prior report of a young wolf dispersing from the CEZ to the surrounding regions (Byrne et al. 2018) further supports evidence of dispersal and possible admixture between the CEZ and surrounding sites. Further studies regarding wolf ecology within the region are warranted, particularly as these collar data are unidirectional and not a complete story of wolf movement in the region. Collectively, these data suggest a robust resident wolf population within the CEZ and set the stage for possible local adaptation to occur within the CEZ as (Figure 2D,E) elevated stress of radiation exposure may have the potential to facilitate rapid local adaptation to site‐specific environmental pressures such as exposure to elevated ionising radiation levels within the CEZ.

### 4.3 Altered Blood Cell Composition

If the elevated radiation doses experienced by the CEZ wolf population have resulted in persistent physiological stress, we expected to see significant differences in signatures of immune function between CEZ and BLR due to the persistent exposure to a genotoxic environment experienced by the CEZ population.

Given the elevated radiation doses displayed by the CEZ wolves, we searched for evidence of immune stress within the CEZ that may result from chronic multigenerational radiation exposure. Mammalian blood plays a key role in radiation induced physiological stress response and the immune system respons can be strongly affected by ionising radiation exposure (Nakamura et al. 1990; Kusunoki et al. 2010) and are critical in repsponding to the cellular and DNA damage. To test this hypothesis, we searched for signatures of radiation‐induced immune modulation by comparing blood cell type ratios between CEZ and BLR via cell deconvolution analyses (Nadel et al. 2020).

The observed shifts in lymphocyte, granulocyte and monocyte composition are consistent with biological signatures of immunological stress in mammals (Nakamura et al. 1990; Kusunoki et al. 2010), supporting our hypothesis that radiation exposure within the CEZ is a significant source of biological stress and consistent with previous data reported for other species within the region (Tovstuha et al. 2022; Lypska et al. 2022; Camplani et al. 1999). There were no significant differences in potentially confounding variables such as age or sex distributions between the CEZ and BLR individuals. The use of a non‐target reference for deconvolution could introduce unintentional biases to the data; however, mice have been used as a model for immunological studies given ancestrally derived homology between humans, mice and canines (Park et al. 2016), and consistent use of this reference across radiation conditions suggests differences observed are indicative of true overarching immune modulation between the two sites. Additional precision would be a when a canine reference becomes available. Additionally, blood cell proportions alone do not directly inform specific causation or individual levels of immune competence. Moreover, differences in immune cell profiles observed between these wild populations may also be influenced by variation in temporal variability (Lochmiller et al. 1994), demography (Charruau et al. 2016), infection history (Bauer et al. 2018), oncogenesis (Wiguna et al. 2022), radiation‐induced immune modulation (Nakamura et al. 1990) and/or interactive effects therein (Lafferty and Holt 2003).

Interestingly, blood cell proportions within the CEZ reflect immunological modulation observed in radiation‐exposed cancer patients and experimentally irradiated laboratory models, with decreasing adaptive immune cells and increased relative proportions of innate immune cells (Ghandhi et al. 2019; Paganetti 2023). Observed trends further support radiation‐specific patterns of immune modulation, with the most radiosensitive immune cell types, T cells and B cells (Paganetti 2023), displaying decreasing proportion in the CEZ, whereas many innate immune cell types show increasing trends with dose.

### 4.4 Regulatory Signatures of Immune Stress and DNA Damage in Chornobyl Wolves

To gain a clearer mechanistic understanding of the immunological patterns revealed through blood cell deconvolution analyses, we searched for divergent patterns of gene expression within the CEZ that may underpin radiation‐induced stress responses. Modules characterised through WGCNA represent groups of genes that may interact in the same functional/regulatory networks (Horvath 2011) and can help identify physiological consequences of site‐specific expression patterns. Gene ontology enrichment of the regulatory modules showing distinct CEZ‐specific divergence highlighted immune, metabolic and DNA repair pathways, mechanisms potentially conserved across species' response to radiation exposure due to their characterisation in previously described transcriptional studies in the CEZ (Kesäniemi et al. 2019; Jernfors et al. 2018).

The three co‐regulatory models (Salmon, Yellow and Brown modules) displaying significant interactions with individual level radiation dose in the CEZ highlight radiation induced stress mechanisms. Eigengene expression for each of these modules displayed a significant interaction of radiation dose and age, and many of the pathways enriched within these modules are relevant to radiation induced senescence physiology, suggesting that inflammation and DNA damage are underlying or driving dose response mechanisms. Radiation‐induced senescense operates through interconneced mechanisms including DNA damage, oxidative stress, telomere shortening, inflamation, and/or stem cell exhaustion, leading to cellular dysfunction, oncogenesis and/or relative lifespan reductions (Kim et al. 2023). The radiation correlated modules reflect each of these processes. The Yellow module enriched for DNA damage repair and aging pathways, including four oncogenic specific pathways highlighting leukaemia and transcriptional misregulation in cancer. Interestingly diver term enrichment describes inflammation mechanisms such as positive regulation of acute inflammatory response and negative regulation of interleukin‐1 production as important underpinnings of inflammatory radiation response mechanisms. The Brown module simmilarly enriched for radiation‐induced pathways influential in aging. Highlighting transcriptional co‐activator malfunction which contributes to pathway modulation influential in radiation induced aging, in particular alterations in co‐activators such as PDZ‐binding motif and PGC‐1a can accelerate the senesces process (Anderson and Prolla 2009; Jeong et al. 2017).

The Salmon module captures downstream radiation damage response mechanisms including endoplasmic reticulum chaperone activity, acute‐phase signalling, and vasculature remodeling. Disruptions in endoplasmic reticulum (ER) function by ionising radiation can impair protein folding and calcium ion storage (Lin et al. 2019), influencing DNA repair capacity and radiation resistance in irradiated cells (Oommen and Prise 2013). ER chaperone pathway enrichment may therefore reflect cellular attempts to manage radiation‐induced ER stress and resulting cellular apoptosis (Lee et al. 2014). Additionally, acute‐phase response and upregulation of vasculature development pathways simmilarly mirror documented responses in cancer patients receiving radiation therapy to mitigate the apoptotic responses (Kim et al. 2020). Together these modules sugest DNA damage, inflammation, and ER stress are primary dose‐response drivers, potentially acting as converging mechanisms driving radiation stress and radiation‐induced senescense in the CEZ wolves as these stressors may accumulate as an individual ages.

The radiation‐correlated candidate gene set further suggests cell signalling, immunological modulation and DNA damage responses significantly correlate with individual level radiation dose rate in this wild, free ranging species. Many of the pathways highlighted in this gene set have estalished associations with immune modulation and radiation induced DNA damage. Enriched of pathways, such as primary immune deficiency, are associated with recurrent infections, allergies, autoimmunity and cancer in humans (Renzi et al. 2020), suggesting dynamic and persistant immune modulation in the face of chronic radiation exposure. Immune cell types have varying sensitivity to ionizing radiation, and radiation induced immunodeficiency can have cascading impacts on health (Lumniczky et al. 2021). Immune regulation not only plays a dynamic role in DNA damage mitigation, but may also contribute to this process through response mechanisms and increasing cellular stress (Lumniczky et al. 2021; Tong et al. 2024) NF‐kappa B signalling further mediates immune cell responses to DNA damage, influencing cell survival while predominantly targeting the p100/p52—RelB NF‐κB complex (Budke et al. 2022). The enriched antigen processing and presentation pathways additionally play crucial roles by linking genotoxic stress signalling to downstream inflammation and immune responses (Tseng‐Rogenski et al. 2015; Cohen et al. 2015; Novak et al. 2020). While the C‐type lectin receptor signalling pathway plays a critical role in recognising and responding to cell death (Drouin et al. 2020). Many of these putative biological processes are often targeted in cancer therapies, where they are exploited for their interactions with DNA damage to promote tumor cell apoptosis and increase treatment efficacy (Briukhovetska et al. 2021; Eluard et al. 2020).

Supporting these results, examination of a priori hypothesised immune signalling and DNA repair mechanisms with known modulation with radiation exposure (Lumniczky et al. 2021) characterises distinct clustering in expression within the CEZ wolves. The chemokine signalling and cytokine‐cytokine signalling pathways are conserved pathways in immune cell signalling important in radiation response (Lumniczky et al. 2021). As innate immune cells recognize cell damage from radiation exposure and process antigens they release various soluble immune mediators called cytokines and chemokines, which drive inflammation and attract adaptive immune cells (Commins et al. 2010). Mismatch repair and base pair excision repair pathways are likewise conserved forms of DNA damage repair underlying radiation induced genomic damage (Chatterjee and Walker 2017; Kinsella 2009). Both of which show simmilar divergence in expression within the CEZ wolves. These data further suggest Chornobyl wolves experience altered immune modulation and DNA repair stress.

Collectively, these regulatory signatures within the CEZ complement those found from cellular deconvolution analyses in highlighting biological signatures of immune stress associated with local radiation exposure within the CEZ, and support the hypothesis of chronic effects of elevated radiation doses experienced by CEZ wolves. Although many of these pathway interpretations are based on human physiology, true wolf‐specific physiological annotations are limited and may differ. Canids provide a widely utilised model for human physiology and cancer research, due to many conserved immune and oncogenic processes (Schiffman and Breen 2015; Chow et al. 2024). The homology between canid and human responses may therefore allow dose‐response mechanisms identified here to inform broader hypotheses about radiation exposure and dose response mechanisms; though these findings warrant further investigation.

### 4.5 Signatures of Selection on Immune Regulatory Genes

Finally, given the biological signatures of increased radiation exposure, immunological and radiation‐induced stress, and possible limitations to gene flow into the CEZ, we asked if chronic multigenerational radiation exposure over the last ~7 generations may be a significant source of pressure driving local contemporary natural selection for the CEZ wolves. With conservative outlier thresholds, we identified 17 SNPs as regions displaying significant genetic differentiation in the CEZ wolves after their divergence from BLR and YLS. These SNPs were enriched for KEGG terms such as Rap1 signalling pathway, Wnt signalling pathway and parathyroid hormone synthesis, secretion and action, highlighting pathways influential in maintaining cell and tissue integrity, cell–cell signalling and DNA damage response (Zhang et al. 2017, 2018; Liu et al. 2022; Khattar et al. 2019).

The two candidate genes (EMC6 & APBB1IP) showing the highest level of CEZ divergence have known roles in mammalian immunity and/or anti‐tumour immune response across a broad range of cancer cell types. Candidate locus one falls within the protein coding region of gene APBB1IP. APBB1IP is a regulator of cell adhesion and migration and affects multiple functions of innate and adaptive immunity, influential in mitigating cancer cell migration (Ge et al. 2021). APBB1IP significantly interacts with APBB1, which plays a crucial role in maintaining cancer stem cell and epithelial‐to‐mesenchymal transition properties, and enhances radiation resistance in lung cancer cells (Lee et al. 2017). APBB1IP is also a prognostic biomarker across cancers, and differential expression is associated with immune‐cell infiltration, particularly by CD8+ T cells and natural killer (NK) cells (Ge et al. 2021). The protein, APBB1IP, interacts with several immune‐related proteins, including RAP1A/B, TLN1/2 and VCL, potentially modulating immune cell behaviour and recruitment (Ge et al. 2021). The putative APBB1IP outlier identified within CEZ wolves is annotated as a missense variant, potentially resulting in altered protein structure. Expression of APBB1IP is associated with immune cell infiltration and prognosis in various cancers and expression varies between different cancer types (Andersson et al. 2009), perhaps suggesting APBB1IP as a candidate for adaptive anti‐tumour immunity in CEZ wolves.

The second and third candidate loci fall just downstream of EMC6, a protein subunit that belongs to the endoplasmic reticulum membrane protein complex and is a regulator of autophagy, apoptosis, ferroptosis, cuproptosis and immune response in cancers (Zhou et al. 2024). EMC6 expression is associated with tumour cell growth, migration and invasion (Li et al. 2019) and differential expression can affect the infiltration of immune cells such as CD4+ T cells, CD8+ T cells and macrophages (Wang et al. 2017). Annotation of these loci reveals potential functional modifications to candidate genes upstream or downstream of the candidate variants. Collectively, these annotations imply functional consequences of the putative targets, which could have beneficial consequences on innate and adaptive immune cell function and stress response in the face of chronic radiation stress.

These genomic signatures of selection suggest that chronic, multigenerational exposure to ionising radiation may be a significant selective force for the wolf population within the CEZ. Previous work on melanism in tree frogs and radiation resistance in fibroblasts from voles has highlighted possible physiological shifts indicative of radiation‐specific adaptation protecting against DNA damage within Chornobyl (Burraco and Orizaola 2022; Mustonen et al. 2018). The interrelated functions of our candidate loci suggest that selection within the CEZ has targeted processes involved in the immune response, particularly those associated with DNA damage, and may have functional implications for the formation, proliferation, growth and/or metastasis of tumour cells in the face of persistent radiation insult. However, the identified candidate genes are broadly involved in several basic biological functions and only represent genes expressed within the blood transcriptome, limiting our analyses of selection to those relevant to blood‐specific and systemic processes detectable within the circulatory system. Immune cells are a physiologically crucial tissue in recognising and mitigating biological responses to ionising radiation. By examining signatures of selection specifically within the blood transcriptome we are able to identify putative targets that may confer added resistance or resilience to radiation‐induced stress within this key tissue type. Although these data further inform the regulatory and cellular deconvolution patterns described herein, further study will be needed to explore genome‐wide patterns of selection outside the context of blood physiology, identify specific allelic targets of selection within each locus and their mechanisms of effect on whole‐organism physiology, performance and fitness. In addition, further examination of geographically broadened patterns of geneflow, genetic drift and simulation frameworks for putative targets under selection within the CEZ will help inform further long‐term consequences of chronic radiation exposure. These data will additionally help examine the presumably interactive effects of differing human pressures across the CEZ boundary. This is of particular interest as wolf populations within the CEZ appear to be persisting at large numbers with densities seven times greater than in nearby BLR (Deryabina et al. 2015), despite chronic and persistently elevated radiation exposure. Further examination of CEZ wolf demography and population viability would also build nicely off these findings to inform the larger ecological consequences of the putative targets of selection.

## 5 Conclusions

Understanding the impact of environmental contamination on wildlife has become an important aspect of biological studies focused on anthropogenic change (e.g., Saaristo et al. 2018). Investigations of rapid evolutionary response to contaminant exposure are critical for evaluating the ecological and evolutionary impacts of such events (Bay et al. 2017). Here, we demonstrate that a single contamination event can have biological impacts at the physiological, regulatory and genomic levels for multiple generations, with the potential to alter the evolutionary trajectory of populations experiencing elevated radiation exposure. Examining adaptation in wild populations exposed to carcinogenic contaminants may help inform disease and therapeutic endpoints important in human health. Gaining further information about the immunological and evolutionary impacts of contaminant exposure may be key for understanding the lasting biological impacts and adaptive significance of environmental pollutants for wildlife and humans globally. Furthermore, by understanding the intricate effects of long‐term radiation exposure on the immune system and cancer biology, scientists can better anticipate and mitigate its health consequences, contributing to improved emergency preparedness and long‐term ecological management.

## Author Contributions

Conceptualisation: C.N.L., S.L.L. and S.C.C.‐S. Methodology: C.N.L., S.C.C.‐S. and T.G.H. Formal analysis: C.N.L., S.C.C.‐S., N.R. and B.N. Investigation: C.N.L., D.S., S.C.W. and M.E.B. Visualisation: C.N.L., S.C.C.‐S., N.R. and M.B. Funding acquisition: C.N.L., S.L.L., T.G.H., J.C.B. and S.C.C.‐S. Project administration: C.N.L., S.L.L. and S.C.C.‐S. Supervision: C.N.L., S.L.L. and S.C.C.‐S. Writing – original draft: C.N.L., S.C.C.‐S., S.L.L., T.G.H., M.E.B., N.R. and J.C.B. Writing – review and editing: C.N.L., S.C.C.‐S., S.L.L., T.G.H., M.E.B., N.R. and J.C.B.

## Funding

This study was supported by US Department of Energy Award: S.L.L., J.C.B.: DE‐FC09‐07SR22506, DE‐EM0004391 and DE‐EM0005228. National Geographic Society Awards NGS: S.L.L., J.C.B.: #EC0629‐13 and #934413. Research Council of Norway—Centers of Excellence: T.G.H.: #223268/F50. National Science Foundation: C.N.L.: #2209074. Princeton University. Institut de Radioprotection et de Sûrete Nucleaire: T.G.H. Norwegian Radiation Protection Authority: T.G.H. University of Georgia Graduate School: C.N.L. Pew Biomedical Scholarship: S.C.C.‐S: #00036035. Princeton Catalysis Initiative/GenMab US Inc.: S.C.C.‐S.: #AWD1007502. USDA National Institute of Food and Agriculture, McIntire Stennis: M.E.B.: #1015915. None of these sponsors or funders played a role in the study design, data collection and analysis, decision to publish, or preparation of the manuscript.

## Disclosure

Benefits generated: Benefits from this research accrue from the sharing of our data and results on public databases as described above.

Disclaimer: This report was prepared as an account of work sponsored by an agency of the United States Government. Neither the United States Government nor any agency thereof, nor any of their employees, makes any warranty, express or implied, or assumes any legal liability or responsibility for the accuracy, completeness, or usefulness of any information, apparatus, product, or process disclosed, or represents that its use would not infringe privately owned rights. Reference herein to any specific commercial product, process, or service by trade name, trademark, manufacturer, or otherwise does not necessarily constitute or imply its endorsement, recommendation, or favouring by the United States Government or any agency thereof. The views and opinions of authors expressed herein do not necessarily state or reflect those of the United States Government or any agency thereof.

## Conflicts of Interest

The authors declare no conflicts of interest.

## Supporting information


**Data S1:** mec70308‐sup‐0001‐DataS1.pdf.

**Data S2:** mec70308‐sup‐0002‐DataS2.pdf.

**Data S3:** mec70308‐sup‐0003‐DataS3.pdf.

**Figure S1:** mec70308‐sup‐0004‐AppendixS1.pdf.
**Figure S2:** mec70308‐sup‐0004‐AppendixS1.pdf.
**Figure S3:** mec70308‐sup‐0004‐AppendixS1.pdf.
**Figure S4:** mec70308‐sup‐0004‐AppendixS1.pdf.
**Figure S5:** mec70308‐sup‐0004‐AppendixS1.pdf.
**Figure S6:** mec70308‐sup‐0004‐AppendixS1.pdf.
**Figure S7:** mec70308‐sup‐0004‐AppendixS1.pdf.
**Figure S8:** mec70308‐sup‐0004‐AppendixS1.pdf.
**Figure S9:** mec70308‐sup‐0004‐AppendixS1.pdf.
**Table S1:** mec70308‐sup‐0004‐AppendixS1.pdf.
**Table S2:** mec70308‐sup‐0004‐AppendixS1.pdf.
**Table S3:** mec70308‐sup‐0004‐AppendixS1.pdf.
**Table S4:** mec70308‐sup‐0004‐AppendixS1.pdf.
**Table S5:** mec70308‐sup‐0004‐AppendixS1.pdf.

## Data Availability

Concurrent data are stored in the NCBI Sequence Read Archive (SRA) under accessions: PRJNA318909 & PRJNA353740. Original data are reposited on Dryad (https://doi.org/10.5061/dryad.cz8w9gjj9). Additional metadata are available at https://github.com/CNLove‐260/GW_transcriptome/ and within the manuscript and supplementary documentation.
