# Supplementary material for: Signatures of Radiation‐Induced Stress and Putative Selection on Immune Targets in Chornobyl Wolves
Source: Mol Ecol. 2026 Apr 28;35:e70308. doi: 10.1111/mec.70308 (PMC13123633; doi:10.1111/mec.70308)
Supplement: Supplementary file 1 — Data S1: mec70308‐sup‐0001‐DataS1.pdf. [file MEC-35-e70308-s004.pdf]

Black Module GO Enrichment									
p value	*term size*	*query size*	*overlap size*	*precision*	*recall*	*term id*	*source*	*term name*	*highlighted*
0.00218958	5049	288	166	0.576388889	0.032877798	GO:0065007	GO:BP	biological regulation	TRUE
0.007811834	4906	288	159	0.552083333	0.032409295	GO:0050789	GO:BP	regulation of biological process	FALSE
0.026413162	4750	288	152	0.527777778	0.032	GO:0050794	GO:BP	regulation of cellular process	FALSE
0.031199184	483	288	27	0.09375	0.055900621	GO:0032101	GO:BP	regulation of response to external stimulus	FALSE
0.031199184	1010	288	45	0.15625	0.044554455	GO:0048584	GO:BP	positive regulation of response to stimulus	FALSE
0.031199184	1301	288	55	0.190972222	0.042275173	GO:0009056	GO:BP	catabolic process	TRUE
0.031199184	372	288	23	0.079861111	0.061827957	GO:0031347	GO:BP	regulation of defense response	FALSE
0.031199184	653	288	33	0.114583333	0.050535988	GO:0080134	GO:BP	regulation of response to stress	FALSE
0.03360183	1736	288	67	0.232638889	0.03859447	GO:0048583	GO:BP	regulation of response to stimulus	FALSE
0.03360183	689	288	34	0.118055556	0.04934688	GO:0006952	GO:BP	defense response	FALSE
0.041351745	477	288	26	0.090277778	0.054507338	GO:0098542	GO:BP	defense response to other organism	FALSE
0.047409402	608	288	30	0.104166667	0.049342105	GO:0043207	GO:BP	response to external biotic stimulus	FALSE
0.047409402	2729	288	94	0.326388889	0.034444852	GO:0048518	GO:BP	positive regulation of biological process	FALSE
0.047409402	189	288	14	0.048611111	0.074074074	GO:0006644	GO:BP	phospholipid metabolic process	TRUE
0.047409402	261	288	17	0.059027778	0.0651341	GO:0002764	GO:BP	immune response-regulating signaling pathway	FALSE
0.047409402	254	288	17	0.059027778	0.066929134	GO:0002757	GO:BP	immune response-activating signaling pathway	TRUE
0.047409402	608	288	30	0.104166667	0.049342105	GO:0051707	GO:BP	response to other organism	FALSE
0.047409402	305	288	19	0.065972222	0.062295082	GO:0009896	GO:BP	positive regulation of catabolic process	FALSE
0.047409402	910	288	40	0.138888889	0.043956044	GO:0009605	GO:BP	response to external stimulus	FALSE
2.76E-08	3584	288	141	0.489583333	0.039341518	GO:0016020	GO:CC	membrane	TRUE
0.000483399	575	288	34	0.118055556	0.059130435	GO:0031984	GO:CC	organelle subcompartment	FALSE
0.001676531	449	288	27	0.09375	0.06013363	GO:0098827	GO:CC	endoplasmic reticulum subcompartment	FALSE
0.001676531	453	288	27	0.09375	0.059602649	GO:0042175	GO:CC	nuclear outer membrane-endoplasmic reticulum membrane network	FALSE
0.001676531	1403	288	60	0.208333333	0.042765502	GO:0031090	GO:CC	organelle membrane	FALSE
0.001676531	443	288	27	0.09375	0.060948081	GO:0005789	GO:CC	endoplasmic reticulum membrane	FALSE
0.003803795	5580	288	171	0.59375	0.030645161	GO:0005737	GO:CC	cytoplasm	FALSE
0.007321136	819	288	38	0.131944444	0.046398046	GO:0005783	GO:CC	endoplasmic reticulum	FALSE
0.014102602	1933	288	71	0.246527778	0.036730471	GO:0012505	GO:CC	endomembrane system	FALSE
0.017113646	196	288	14	0.048611111	0.071428571	GO:0000139	GO:CC	Golgi membrane	FALSE
0.020421645	7	288	3	0.010416667	0.428571429	GO:0036513	GO:CC	Derlin-1 retrotranslocation complex	FALSE
0.033684398	748	288	33	0.114583333	0.044117647	GO:0098588	GO:CC	bounding membrane of organelle	FALSE
0.047122722	705	288	31	0.107638889	0.043971631	GO:0005794	GO:CC	Golgi apparatus	FALSE
0.00934324	2938	288	103	0.357638889	0.035057862	GO:0036094	GO:MF	small molecule binding	TRUE
0.00934324	1735	288	70	0.243055556	0.040345821	GO:0043169	GO:MF	cation binding	FALSE
0.00934324	1702	288	68	0.236111111	0.039952996	GO:0046872	GO:MF	metal ion binding	FALSE

0.014661307	2844	288	99	0.34375	0.034810127	GO:0043167	GO:MF	ion binding	FALSE
0.018140751	3652	288	120	0.416666667	0.032858708	GO:0003824	GO:MF	catalytic activity	TRUE
0.003098377	92	288	11	0.038194444	0.119565217	KEGG:04071	KEGG	Sphingolipid signaling pathway	FALSE
0.003098377	52	288	8	0.027777778	0.153846154	KEGG:04724	KEGG	Glutamatergic synapse	FALSE
0.003098377	37	288	7	0.024305556	0.189189189	KEGG:04730	KEGG	Long-term depression	FALSE
0.006913834	62	288	8	0.027777778	0.129032258	KEGG:04750	KEGG	Inflammatory mediator regulation of TRP channels	FALSE
0.006913834	79	288	9	0.03125	0.113924051	KEGG:04728	KEGG	Dopaminergic synapse	FALSE
0.013186665	315	288	19	0.065972222	0.06031746	KEGG:05200	KEGG	Pathways in cancer	FALSE
0.014697397	41	288	6	0.020833333	0.146341463	KEGG:04720	KEGG	Long-term potentiation	FALSE
0.014697397	92	288	9	0.03125	0.097826087	KEGG:04921	KEGG	Oxytocin signaling pathway	FALSE
0.018365702	60	288	7	0.024305556	0.116666667	KEGG:04912	KEGG	GnRH signaling pathway	FALSE
0.020632531	65	288	7	0.024305556	0.107692308	KEGG:04270	KEGG	Vascular smooth muscle contraction	FALSE
0.020632531	82	288	8	0.027777778	0.097560976	KEGG:04070	KEGG	Phosphatidylinositol signaling system	FALSE
0.020632531	63	288	7	0.024305556	0.111111111	KEGG:00562	KEGG	Inositol phosphate metabolism	FALSE
0.020632531	19	288	4	0.013888889	0.210526316	KEGG:05143	KEGG	African trypanosomiasis	FALSE
0.023886812	88	288	8	0.027777778	0.090909091	KEGG:04310	KEGG	Wnt signaling pathway	FALSE
0.023886812	50	288	6	0.020833333	0.12	KEGG:04916	KEGG	Melanogenesis	FALSE
0.023886812	69	288	7	0.024305556	0.101449275	KEGG:03015	KEGG	mRNA surveillance pathway	FALSE
0.033768364	74	288	7	0.024305556	0.094594595	KEGG:05224	KEGG	Breast cancer	FALSE

Blue Module GO Enrichment

p value	*term size*	*query size*	*overlap size*	*precision*	*recall*	*term id*	*source*	*term name*	*highlighted*
5.01E-16	5049	1766	942	0.533408834	0.186571598	GO:0065007	GO:BP	biological regulation	TRUE
9.73E-16	4906	1766	917	0.519252548	0.186913983	GO:0050789	GO:BP	regulation of biological process	FALSE
5.48E-14	4750	1766	883	0.5	0.185894737	GO:0050794	GO:BP	regulation of cellular process	FALSE
2.83E-09	2801	1766	544	0.30804077	0.194216351	GO:0019222	GO:BP	regulation of metabolic process	FALSE
1.00E-07	3470	1766	643	0.36409966	0.185302594	GO:0050896	GO:BP	response to stimulus	FALSE
1.55E-07	2949	1766	557	0.315402039	0.188877586	GO:0051716	GO:BP	cellular response to stimulus	FALSE
2.93E-07	849	1766	195	0.110419026	0.229681979	GO:0036211	GO:BP	protein modification process	FALSE
2.93E-07	2241	1766	438	0.24801812	0.195448461	GO:0009889	GO:BP	regulation of biosynthetic process	FALSE
3.60E-07	2561	1766	490	0.277463194	0.191331511	GO:0060255	GO:BP	regulation of macromolecule metabolic process	FALSE
4.09E-07	2729	1766	517	0.292751982	0.189446684	GO:0048518	GO:BP	positive regulation of biological process	FALSE
5.23E-07	2590	1766	493	0.279161948	0.19034749	GO:0048522	GO:BP	positive regulation of cellular process	FALSE
5.23E-07	2179	1766	425	0.240656852	0.195043598	GO:0010556	GO:BP	regulation of macromolecule biosynthetic process	FALSE
5.88E-07	2472	1766	473	0.26783692	0.191343042	GO:0051179	GO:BP	localization	TRUE
1.01E-06	1707	1766	343	0.194224236	0.200937317	GO:0051641	GO:BP	cellular localization	FALSE
1.82E-06	1840	1766	364	0.206115515	0.197826087	GO:0019538	GO:BP	protein metabolic process	FALSE
2.24E-06	2304	1766	441	0.249716874	0.19140625	GO:0080090	GO:BP	regulation of primary metabolic process	FALSE
2.58E-06	1688	1766	337	0.190826727	0.19964455	GO:0019219	GO:BP	regulation of nucleobase-containing compound metabolic process	FALSE
2.76E-06	490	1766	122	0.069082673	0.248979592	GO:0044087	GO:BP	regulation of cellular component biogenesis	FALSE

2.76E-06	733	1766	168	0.095130238	0.229195089	GO:0046907	GO:BP	intracellular transport	FALSE
2.82E-06	777	1766	176	0.099660249	0.226512227	GO:0016192	GO:BP	vesicle-mediated transport	FALSE
3.04E-06	2133	1766	411	0.232729332	0.192686357	GO:0010468	GO:BP	regulation of gene expression	FALSE
3.32E-06	1938	1766	378	0.214043035	0.19504644	GO:0006810	GO:BP	transport	FALSE
4.50E-06	1736	1766	343	0.194224236	0.197580645	GO:0048583	GO:BP	regulation of response to stimulus	FALSE
5.14E-06	956	1766	207	0.117214043	0.216527197	GO:0043412	GO:BP	macromolecule modification	FALSE
5.79E-06	1747	1766	344	0.194790487	0.196908987	GO:0006996	GO:BP	organelle organization	FALSE
2.02E-05	1696	1766	332	0.18799547	0.195754717	GO:0006950	GO:BP	response to stress	FALSE
2.39E-05	1301	1766	264	0.149490374	0.20292083	GO:0009056	GO:BP	catabolic process	FALSE
2.57E-05	2466	1766	459	0.2599094	0.186131387	GO:0023052	GO:BP	signaling	FALSE
3.62E-05	531	1766	125	0.070781427	0.235404896	GO:0043687	GO:BP	post-translational protein modification	FALSE
3.63E-05	2115	1766	400	0.226500566	0.189125296	GO:0051234	GO:BP	establishment of localization	FALSE
4.55E-05	178	1766	54	0.030577576	0.303370787	GO:0048193	GO:BP	Golgi vesicle transport	FALSE
5.11E-05	2476	1766	458	0.259343148	0.184975767	GO:0007154	GO:BP	cell communication	FALSE
5.11E-05	1010	1766	211	0.119479049	0.208910891	GO:0048584	GO:BP	positive regulation of response to stimulus	FALSE
5.93E-05	1605	1766	313	0.177236693	0.195015576	GO:0009893	GO:BP	positive regulation of metabolic process	FALSE
7.21E-05	1420	1766	281	0.159116648	0.197887324	GO:0035556	GO:BP	intracellular signal transduction	FALSE
8.89E-05	504	1766	118	0.066817667	0.234126984	GO:0070647	GO:BP	protein modification by small protein conjugation or removal	FALSE
0.000119278	1294	1766	258	0.146092865	0.199381762	GO:0042221	GO:BP	response to chemical	FALSE
0.000124241	392	1766	96	0.054360136	0.244897959	GO:0016567	GO:BP	protein ubiquitination	FALSE
0.000132898	1443	1766	283	0.160249151	0.196119196	GO:0006351	GO:BP	DNA-templated transcription	FALSE
0.000141988	986	1766	204	0.115515289	0.206896552	GO:0051649	GO:BP	establishment of localization in cell	FALSE
0.000153725	1517	1766	295	0.167044168	0.194462755	GO:0051252	GO:BP	regulation of RNA metabolic process	FALSE
0.000279437	1474	1766	286	0.161947905	0.194029851	GO:0023051	GO:BP	regulation of signaling	FALSE
0.000297	2314	1766	425	0.240656852	0.18366465	GO:0007165	GO:BP	signal transduction	FALSE
0.000341321	428	1766	101	0.057191393	0.235981308	GO:0032446	GO:BP	protein modification by small protein conjugation	FALSE
0.000355762	667	1766	145	0.082106455	0.217391304	GO:0009967	GO:BP	positive regulation of signal transduction	FALSE
0.000468432	490	1766	112	0.063420159	0.228571429	GO:0051130	GO:BP	positive regulation of cellular component organization	FALSE
0.000473574	727	1766	155	0.087768969	0.213204952	GO:0023056	GO:BP	positive regulation of signaling	FALSE
0.000473574	1367	1766	266	0.150622877	0.194586686	GO:2001141	GO:BP	regulation of RNA biosynthetic process	FALSE
0.000473574	1361	1766	265	0.150056625	0.194709772	GO:0006355	GO:BP	regulation of DNA-templated transcription	FALSE
0.000484884	179	1766	51	0.028878822	0.284916201	GO:1902903	GO:BP	regulation of supramolecular fiber organization	FALSE
0.000520029	1470	1766	283	0.160249151	0.192517007	GO:0010646	GO:BP	regulation of cell communication	FALSE
0.000554659	2522	1766	456	0.258210646	0.180808882	GO:0032501	GO:BP	multicellular organismal process	FALSE
0.000619776	1074	1766	215	0.121744054	0.20018622	GO:0051128	GO:BP	regulation of cellular component organization	FALSE

0.000708529	583	1766	128	0.072480181	0.219554031	GO:0033043	GO:BP	regulation of organelle organization	FALSE
0.000764706	1259	1766	246	0.139297848	0.195393169	GO:0070727	GO:BP	cellular macromolecule localization	FALSE
0.000904422	1333	1766	258	0.146092865	0.193548387	GO:0009966	GO:BP	regulation of signal transduction	FALSE
0.000904422	720	1766	152	0.086070215	0.211111111	GO:0010647	GO:BP	positive regulation of cell communication	FALSE
0.000969197	1252	1766	244	0.138165345	0.194888179	GO:0008104	GO:BP	protein localization	FALSE
0.001006457	818	1766	169	0.095696489	0.206601467	GO:0141124	GO:BP	intracellular signaling cassette	FALSE
0.001011019	2409	1766	435	0.246319366	0.180572852	GO:0032502	GO:BP	developmental process	FALSE
0.001011019	1455	1766	278	0.157417894	0.191065292	GO:0033036	GO:BP	macromolecule localization	FALSE
0.00106956	986	1766	198	0.11211778	0.200811359	GO:0010558	GO:BP	negative regulation of macromolecule biosynthetic process	FALSE
0.001251713	21	1766	12	0.006795017	0.571428571	GO:0034063	GO:BP	stress granule assembly	TRUE
0.001376347	251	1766	64	0.036240091	0.25498008	GO:1901137	GO:BP	carbohydrate derivative biosynthetic process	FALSE
0.001501066	262	1766	66	0.037372593	0.251908397	GO:0010638	GO:BP	positive regulation of organelle organization	FALSE
0.001501066	61	1766	23	0.013023783	0.37704918	GO:1902117	GO:BP	positive regulation of organelle assembly	FALSE
0.001633371	420	1766	96	0.054360136	0.228571429	GO:1901135	GO:BP	carbohydrate derivative metabolic process	FALSE
0.001633371	145	1766	42	0.023782559	0.289655172	GO:0007033	GO:BP	vacuole organization	FALSE
0.001672598	78	1766	27	0.015288788	0.346153846	GO:0007030	GO:BP	Golgi organization	FALSE
0.001683822	238	1766	61	0.034541336	0.256302521	GO:0044089	GO:BP	positive regulation of cellular component biogenesis	FALSE
0.00194587	1470	1766	278	0.157417894	0.189115646	GO:0010604	GO:BP	positive regulation of macromolecule metabolic process	FALSE
0.00194587	1009	1766	200	0.113250283	0.198216056	GO:0009890	GO:BP	negative regulation of biosynthetic process	FALSE
0.002202297	533	1766	116	0.065685164	0.217636023	GO:0010629	GO:BP	negative regulation of gene expression	FALSE
0.002430439	2347	1766	421	0.238391846	0.179377929	GO:0048519	GO:BP	negative regulation of biological process	FALSE
0.002430439	897	1766	180	0.101925255	0.200668896	GO:1902531	GO:BP	regulation of intracellular signal transduction	FALSE
0.00253221	262	1766	65	0.036806342	0.248091603	GO:0010256	GO:BP	endomembrane system organization	FALSE
0.002539884	134	1766	39	0.022083805	0.291044776	GO:0051258	GO:BP	protein polymerization	FALSE
0.002810971	168	1766	46	0.026047565	0.273809524	GO:0000209	GO:BP	protein polyubiquitination	FALSE
0.003520795	811	1766	164	0.092865232	0.202219482	GO:0070887	GO:BP	cellular response to chemical stimulus	FALSE
0.003625697	2247	1766	403	0.22819932	0.179350245	GO:0048856	GO:BP	anatomical structure development	FALSE
0.003625697	113	1766	34	0.019252548	0.300884956	GO:0070085	GO:BP	glycosylation	FALSE
0.003625697	1156	1766	223	0.126274066	0.192906574	GO:0010605	GO:BP	negative regulation of macromolecule metabolic process	FALSE
0.004201748	936	1766	185	0.104756512	0.197649573	GO:0045935	GO:BP	positive regulation of nucleobase-containing compound metabolic process	FALSE

0.004744592	776	1766	157	0.088901472	0.202319588	GO:0009057	GO:BP	macromolecule catabolic process	FALSE
0.004835182	227	1766	57	0.032276331	0.251101322	GO:0007507	GO:BP	heart development	TRUE
0.004964407	1080	1766	209	0.118346546	0.193518519	GO:0065008	GO:BP	regulation of biological quality	FALSE
0.005229419	158	1766	43	0.024348811	0.272151899	GO:0009100	GO:BP	glycoprotein metabolic process	FALSE
0.005229419	134	1766	38	0.021517554	0.28358209	GO:0009101	GO:BP	glycoprotein biosynthetic process	FALSE
0.005272993	97	1766	30	0.016987542	0.309278351	GO:0032271	GO:BP	regulation of protein polymerization	FALSE
0.005327871	218	1766	55	0.031143828	0.252293578	GO:0043254	GO:BP	regulation of protein-containing complex assembly	FALSE
0.005399602	2284	1766	407	0.230464326	0.178196147	GO:0048523	GO:BP	negative regulation of cellular process	FALSE
0.006287595	724	1766	147	0.083238958	0.203038674	GO:0006508	GO:BP	proteolysis	FALSE
0.006597178	28	1766	13	0.007361268	0.464285714	GO:0031113	GO:BP	regulation of microtubule polymerization	FALSE
0.00662903	1247	1766	236	0.133635334	0.18925421	GO:0009892	GO:BP	negative regulation of metabolic process	FALSE
0.00675788	455	1766	99	0.05605889	0.217582418	GO:0051603	GO:BP	proteolysis involved in protein catabolic process	FALSE
0.008906196	162	1766	43	0.024348811	0.265432099	GO:0016197	GO:BP	endosomal transport	FALSE
0.009317624	44	1766	17	0.009626274	0.386363636	GO:0046785	GO:BP	microtubule polymerization	FALSE
0.009824428	566	1766	118	0.066817667	0.208480565	GO:0033365	GO:BP	protein localization to organelle	FALSE
0.009864697	372	1766	83	0.046998867	0.22311828	GO:0061024	GO:BP	membrane organization	TRUE
0.010168494	477	1766	102	0.057757644	0.213836478	GO:1902533	GO:BP	positive regulation of intracellular signal transduction	FALSE
0.011152101	57	1766	20	0.011325028	0.350877193	GO:0080171	GO:BP	lytic vacuole organization	FALSE
0.011152101	57	1766	20	0.011325028	0.350877193	GO:0007040	GO:BP	lysosome organization	FALSE
0.0114484	251	1766	60	0.033975085	0.239043825	GO:0051493	GO:BP	regulation of cytoskeleton organization	FALSE
0.011585316	1170	1766	221	0.125141563	0.188888889	GO:0051239	GO:BP	regulation of multicellular organismal process	FALSE
0.012930104	300	1766	69	0.039071348	0.23	GO:0006914	GO:BP	autophagy	FALSE
0.012930104	84	1766	26	0.014722537	0.30952381	GO:0051650	GO:BP	establishment of vesicle localization	FALSE
0.012930104	300	1766	69	0.039071348	0.23	GO:0061919	GO:BP	process utilizing autophagic mechanism	FALSE
0.01417685	355	1766	79	0.044733862	0.222535211	GO:0006886	GO:BP	intracellular protein transport	FALSE
0.014659992	361	1766	80	0.045300113	0.221606648	GO:0019941	GO:BP	modification-dependent protein catabolic process	FALSE
0.014659992	1086	1766	206	0.116647792	0.189686924	GO:0007166	GO:BP	cell surface receptor signaling pathway	FALSE
0.015251043	851	1766	166	0.093997735	0.19506463	GO:0071705	GO:BP	nitrogen compound transport	FALSE
0.015251043	42	1766	16	0.009060023	0.380952381	GO:0002709	GO:BP	regulation of T cell mediated immunity	TRUE
0.015251043	723	1766	144	0.081540204	0.199170124	GO:0044281	GO:BP	small molecule metabolic process	TRUE
0.015326621	758	1766	150	0.084937712	0.197889182	GO:0045893	GO:BP	positive regulation of DNA-templated transcription	FALSE

0.015385584	362	1766	80	0.045300113	0.220994475	GO:0043632	GO:BP	modification-dependent macromolecule catabolic process	FALSE
0.01595957	90	1766	27	0.015288788	0.3	GO:0046488	GO:BP	phosphatidylinositol metabolic process	TRUE
0.01595957	655	1766	132	0.074745187	0.201526718	GO:0015031	GO:BP	protein transport	FALSE
0.01608644	72	1766	23	0.013023783	0.319444444	GO:0061025	GO:BP	membrane fusion	FALSE
0.016183681	352	1766	78	0.04416761	0.221590909	GO:0006511	GO:BP	ubiquitin-dependent protein catabolic process	FALSE
0.016506039	760	1766	150	0.084937712	0.197368421	GO:1902680	GO:BP	positive regulation of RNA biosynthetic process	FALSE
0.017016465	1745	1766	314	0.177802945	0.179942693	GO:0007275	GO:BP	multicellular organism development	FALSE
0.017764917	744	1766	147	0.083238958	0.197580645	GO:0009888	GO:BP	tissue development	FALSE
0.018519634	1051	1766	199	0.112684032	0.189343482	GO:0006366	GO:BP	transcription by RNA polymerase II	FALSE
0.018906062	39	1766	15	0.008493771	0.384615385	GO:0006493	GO:BP	protein O-linked glycosylation	FALSE
0.018906062	43	1766	16	0.009060023	0.372093023	GO:0071677	GO:BP	positive regulation of mononuclear cell migration	TRUE
0.018906062	653	1766	131	0.074178935	0.200612557	GO:0080134	GO:BP	regulation of response to stress	FALSE
0.018906062	839	1766	163	0.092298981	0.194278903	GO:0051254	GO:BP	positive regulation of RNA metabolic process	FALSE
0.019510699	14	1766	8	0.004530011	0.571428571	GO:0060765	GO:BP	regulation of androgen receptor signaling pathway	TRUE
0.019562365	344	1766	76	0.043035108	0.220930233	GO:0097435	GO:BP	supramolecular fiber organization	FALSE
0.021386918	552	1766	113	0.06398641	0.204710145	GO:0009894	GO:BP	regulation of catabolic process	FALSE
0.021907928	28	1766	12	0.006795017	0.428571429	GO:0002711	GO:BP	positive regulation of T cell mediated immunity	FALSE
0.021907928	52	1766	18	0.010192525	0.346153846	GO:0002456	GO:BP	T cell mediated immunity	FALSE
0.022175269	265	1766	61	0.034541336	0.230188679	GO:0090407	GO:BP	organophosphate biosynthetic process	TRUE
0.022175269	102	1766	29	0.016421291	0.284313725	GO:0006486	GO:BP	protein glycosylation	FALSE
0.022175269	102	1766	29	0.016421291	0.284313725	GO:0043413	GO:BP	macromolecule glycosylation	FALSE
0.022175269	126	1766	34	0.019252548	0.26984127	GO:0110053	GO:BP	regulation of actin filament organization	FALSE
0.022175269	6	1766	5	0.002831257	0.833333333	GO:0001967	GO:BP	suckling behavior	TRUE
0.022175269	48	1766	17	0.009626274	0.354166667	GO:0031110	GO:BP	regulation of microtubule polymerization or depolymerization	FALSE
0.022175269	308	1766	69	0.039071348	0.224025974	GO:0009792	GO:BP	embryo development ending in birth or egg hatching	FALSE
0.022361421	88	1766	26	0.014722537	0.295454545	GO:0051648	GO:BP	vesicle localization	FALSE
0.02366677	566	1766	115	0.065118913	0.203180212	GO:0030163	GO:BP	protein catabolic process	FALSE
0.024797615	304	1766	68	0.038505096	0.223684211	GO:0043009	GO:BP	chordate embryonic development	FALSE
0.026125033	4	1766	4	0.002265006	1	GO:0071696	GO:BP	ectodermal placode development	FALSE
0.028119331	62	1766	20	0.011325028	0.322580645	GO:0002687	GO:BP	positive regulation of leukocyte migration	FALSE
0.028764489	194	1766	47	0.026613817	0.242268041	GO:0030522	GO:BP	intracellular receptor signaling pathway	FALSE
0.028764489	138	1766	36	0.020385051	0.260869565	GO:1902115	GO:BP	regulation of organelle assembly	FALSE

0.030724511	76	1766	23	0.013023783	0.302631579	GO:0006888	GO:BP	endoplasmic reticulum to Golgi vesicle-mediated transport	FALSE
0.030934144	67	1766	21	0.01189128	0.313432836	GO:0031109	GO:BP	microtubule polymerization or depolymerization	FALSE
0.030934144	114	1766	31	0.017553794	0.271929825	GO:0006282	GO:BP	regulation of DNA repair	TRUE
0.030934144	15	1766	8	0.004530011	0.533333333	GO:0045880	GO:BP	positive regulation of smoothened signaling pathway	FALSE
0.031431824	216	1766	51	0.028878822	0.236111111	GO:0051656	GO:BP	establishment of organelle localization	FALSE
0.031431824	958	1766	181	0.102491506	0.188935282	GO:0033554	GO:BP	cellular response to stress	FALSE
0.033209061	12	1766	7	0.00396376	0.583333333	GO:0048199	GO:BP	vesicle targeting, to, from or within Golgi	FALSE
0.036143452	482	1766	99	0.05605889	0.205394191	GO:0030097	GO:BP	hemopoiesis	TRUE
0.036848817	471	1766	97	0.054926387	0.205944798	GO:0009790	GO:BP	embryo development	FALSE
0.039460579	38	1766	14	0.00792752	0.368421053	GO:0044088	GO:BP	regulation of vacuole organization	FALSE
0.039809093	495	1766	101	0.057191393	0.204040404	GO:0040011	GO:BP	locomotion	TRUE
0.040244078	484	1766	99	0.05605889	0.204545455	GO:0019637	GO:BP	organophosphate metabolic process	FALSE
0.040244078	305	1766	67	0.037938845	0.219672131	GO:0009896	GO:BP	positive regulation of catabolic process	FALSE
0.040244078	349	1766	75	0.042468856	0.214899713	GO:0006338	GO:BP	chromatin remodeling	TRUE
0.040531719	1102	1766	204	0.115515289	0.185117967	GO:0002376	GO:BP	immune system process	TRUE
0.040601984	92	1766	26	0.014722537	0.282608696	GO:0007034	GO:BP	vacuolar transport	FALSE
0.041477481	262	1766	59	0.033408834	0.22519084	GO:1903131	GO:BP	mononuclear cell differentiation	TRUE
0.042447608	284	1766	63	0.035673839	0.221830986	GO:0051640	GO:BP	organelle localization	FALSE
0.043398279	83	1766	24	0.013590034	0.289156627	GO:0070507	GO:BP	regulation of microtubule cytoskeleton organization	FALSE
0.043654556	520	1766	105	0.059456399	0.201923077	GO:0001775	GO:BP	cell activation	FALSE
0.043684757	334	1766	72	0.040770102	0.215568862	GO:0019725	GO:BP	cellular homeostasis	TRUE
0.043684757	1195	1766	219	0.12400906	0.183263598	GO:0009891	GO:BP	positive regulation of biosynthetic process	FALSE
0.045202425	173	1766	42	0.023782559	0.242774566	GO:1903311	GO:BP	regulation of mRNA metabolic process	FALSE
0.045880563	807	1766	154	0.087202718	0.190830235	GO:0045184	GO:BP	establishment of protein localization	FALSE
0.045992592	56	1766	18	0.010192525	0.321428571	GO:0090174	GO:BP	organelle membrane fusion	FALSE
0.046694393	291	1766	64	0.036240091	0.219931271	GO:0000165	GO:BP	MAPK cascade	FALSE
0.049851013	31	1766	12	0.006795017	0.387096774	GO:0045815	GO:BP	transcription initiation-coupled chromatin remodeling	TRUE
4.42E-27	5580	1766	1067	0.60419026	0.191218638	GO:0005737	GO:CC	cytoplasm	TRUE
1.20E-16	1933	1766	428	0.242355606	0.221417486	GO:0012505	GO:CC	endomembrane system	FALSE
1.70E-11	2171	1766	446	0.252548131	0.205435283	GO:0005654	GO:CC	nucleoplasm	FALSE
1.57E-10	1403	1766	307	0.173839185	0.218816821	GO:0031090	GO:CC	organelle membrane	FALSE
3.60E-10	3584	1766	671	0.3799547	0.187220982	GO:0016020	GO:CC	membrane	FALSE
1.35E-09	2131	1766	428	0.242355606	0.200844674	GO:0005829	GO:CC	cytosol	FALSE
1.19E-08	2745	1766	524	0.296715742	0.190892532	GO:0043233	GO:CC	organelle lumen	FALSE
1.19E-08	2745	1766	524	0.296715742	0.190892532	GO:0031974	GO:CC	membrane-enclosed lumen	FALSE
1.19E-08	2745	1766	524	0.296715742	0.190892532	GO:0070013	GO:CC	intracellular organelle lumen	FALSE
6.64E-08	575	1766	142	0.080407701	0.246956522	GO:0031984	GO:CC	organelle subcompartment	FALSE
8.17E-08	1035	1766	227	0.128539071	0.219323671	GO:1902494	GO:CC	catalytic complex	FALSE
1.06E-07	2518	1766	480	0.27180068	0.190627482	GO:0031981	GO:CC	nuclear lumen	FALSE
1.51E-07	705	1766	165	0.093431484	0.234042553	GO:0005794	GO:CC	Golgi apparatus	FALSE
5.08E-06	525	1766	125	0.070781427	0.238095238	GO:0016604	GO:CC	nuclear body	FALSE

7.04E-06	318	1766	84	0.047565119	0.264150943	GO:0005773	GO:CC	vacuole	FALSE
8.78E-06	819	1766	178	0.100792752	0.217338217	GO:0005783	GO:CC	endoplasmic reticulum	FALSE
1.38E-05	541	1766	126	0.071347678	0.232902033	GO:1990234	GO:CC	transferase complex	FALSE
2.89E-05	375	1766	93	0.052661382	0.248	GO:0012506	GO:CC	vesicle membrane	FALSE
3.36E-05	748	1766	162	0.091732729	0.21657754	GO:0098588	GO:CC	bounding membrane of organelle	FALSE
3.36E-05	189	1766	55	0.031143828	0.291005291	GO:0005769	GO:CC	early endosome	FALSE
5.28E-05	821	1766	174	0.098527746	0.211936663	GO:0097708	GO:CC	intracellular vesicle	FALSE
5.31E-05	366	1766	90	0.050962627	0.245901639	GO:0030659	GO:CC	cytoplasmic vesicle membrane	FALSE
5.48E-05	879	1766	184	0.10419026	0.209328783	GO:0031982	GO:CC	vesicle	FALSE
5.71E-05	818	1766	173	0.097961495	0.211491443	GO:0031410	GO:CC	cytoplasmic vesicle	FALSE
7.87E-05	611	1766	135	0.076443941	0.220949264	GO:0140535	GO:CC	intracellular protein-containing complex	FALSE
0.00011866	137	1766	42	0.023782559	0.306569343	GO:0098791	GO:CC	Golgi apparatus subcompartment	FALSE
0.00014788	461	1766	106	0.06002265	0.229934924	GO:0005768	GO:CC	endosome	FALSE
0.000151956	50	1766	21	0.01189128	0.42	GO:0099023	GO:CC	vesicle tethering complex	FALSE
0.000229687	252	1766	65	0.036806342	0.257936508	GO:0016607	GO:CC	nuclear speck	FALSE
0.000229687	71	1766	26	0.014722537	0.366197183	GO:0031901	GO:CC	early endosome membrane	FALSE
0.000294763	453	1766	103	0.058323896	0.227373068	GO:0042175	GO:CC	nuclear outer membrane-endoplasmic reticulum membrane network	FALSE
0.000306446	260	1766	66	0.037372593	0.253846154	GO:0000323	GO:CC	lytic vacuole	FALSE
0.000306446	260	1766	66	0.037372593	0.253846154	GO:0005764	GO:CC	lysosome	FALSE
0.000308867	449	1766	102	0.057757644	0.227171492	GO:0098827	GO:CC	endoplasmic reticulum subcompartment	FALSE
0.000614194	43	1766	18	0.010192525	0.418604651	GO:0005798	GO:CC	Golgi-associated vesicle	FALSE
0.000631176	419	1766	95	0.053793884	0.22673031	GO:0000785	GO:CC	chromatin	TRUE
0.000751438	217	1766	56	0.031710079	0.258064516	GO:0010008	GO:CC	endosome membrane	FALSE
0.000751438	443	1766	99	0.05605889	0.223476298	GO:0005789	GO:CC	endoplasmic reticulum membrane	FALSE
0.001013604	529	1766	114	0.064552661	0.215500945	GO:0098796	GO:CC	membrane protein complex	FALSE
0.002510574	176	1766	46	0.026047565	0.261363636	GO:0005774	GO:CC	vacuolar membrane	FALSE
0.00281281	192	1766	49	0.027746319	0.255208333	GO:0000151	GO:CC	ubiquitin ligase complex	FALSE
0.002871277	44	1766	17	0.009626274	0.386363636	GO:0000118	GO:CC	histone deacetylase complex	FALSE
0.004199551	93	1766	28	0.01585504	0.301075269	GO:0005802	GO:CC	trans-Golgi network	FALSE
0.006864397	17	1766	9	0.005096263	0.529411765	GO:0032588	GO:CC	trans-Golgi network membrane	FALSE
0.007000673	149	1766	39	0.022083805	0.261744966	GO:0035770	GO:CC	ribonucleoprotein granule	FALSE
0.007679796	140	1766	37	0.020951302	0.264285714	GO:0005765	GO:CC	lysosomal membrane	FALSE
0.007679796	140	1766	37	0.020951302	0.264285714	GO:0098852	GO:CC	lytic vacuole membrane	FALSE
0.010072469	137	1766	36	0.020385051	0.262773723	GO:0036464	GO:CC	cytoplasmic ribonucleoprotein granule	FALSE
0.017240185	126	1766	33	0.018686297	0.261904762	GO:0031461	GO:CC	cullin-RING ubiquitin ligase complex	FALSE
0.017240185	146	1766	37	0.020951302	0.253424658	GO:0005770	GO:CC	late endosome	FALSE
0.017618184	78	1766	23	0.013023783	0.294871795	GO:0140534	GO:CC	endoplasmic reticulum protein-containing complex	FALSE
0.021469158	27	1766	11	0.006228766	0.407407407	GO:0030660	GO:CC	Golgi-associated vesicle membrane	FALSE
0.021711595	31	1766	12	0.006795017	0.387096774	GO:0030119	GO:CC	AP-type membrane coat adaptor complex	FALSE
0.023642007	13	1766	7	0.00396376	0.538461538	GO:0030008	GO:CC	TRAPP complex	FALSE
0.025149112	10	1766	6	0.003397508	0.6	GO:0008250	GO:CC	oligosaccharyltransferase complex	FALSE
0.027516162	120	1766	31	0.017553794	0.258333333	GO:0030135	GO:CC	coated vesicle	FALSE
0.035913507	50	1766	16	0.009060023	0.32	GO:0005795	GO:CC	Golgi stack	FALSE

0.037629107	5	1766	4	0.002265006	0.8	GO:0097543	GO:CC	ciliary inversin compartment	TRUE
0.037629107	14	1766	7	0.00396376	0.5	GO:0070971	GO:CC	endoplasmic reticulum exit site	FALSE
0.044041763	84	1766	23	0.013023783	0.273809524	GO:0055037	GO:CC	recycling endosome	FALSE
0.046363415	8	1766	5	0.002831257	0.625	GO:0030123	GO:CC	AP-3 adaptor complex	FALSE
0.048573041	61	1766	18	0.010192525	0.295081967	GO:0030117	GO:CC	membrane coat	FALSE
0.048573041	61	1766	18	0.010192525	0.295081967	GO:0048475	GO:CC	coated membrane	FALSE
0.049036494	66	1766	19	0.010758777	0.287878788	GO:0071013	GO:CC	catalytic step 2 spliceosome	FALSE
0.049036494	3	1766	3	0.001698754	1	GO:0031264	GO:CC	death-inducing signaling complex	TRUE
0.049036494	3	1766	3	0.001698754	1	GO:0035868	GO:CC	alphav-beta3 integrin-HMGB1 complex	TRUE
0.049036494	3	1766	3	0.001698754	1	GO:0097342	GO:CC	ripoptosome	FALSE
5.95E-18	3652	1766	727	0.411664779	0.199069003	GO:0003824	GO:MF	catalytic activity	TRUE
2.21E-09	1519	1766	325	0.18403171	0.21395655	GO:0140096	GO:MF	catalytic activity, acting on a protein	FALSE
2.93E-09	3253	1766	615	0.348244621	0.189056256	GO:0005515	GO:MF	protein binding	TRUE
2.93E-09	2938	1766	563	0.318799547	0.191626957	GO:0036094	GO:MF	small molecule binding	TRUE
9.51E-09	2844	1766	544	0.30804077	0.191279887	GO:0043167	GO:MF	ion binding	FALSE
1.68E-08	1453	1766	307	0.173839185	0.211286992	GO:0016740	GO:MF	transferase activity	FALSE
1.98E-06	1115	1766	237	0.134201586	0.212556054	GO:0019899	GO:MF	enzyme binding	FALSE
1.17E-05	1456	1766	292	0.165345413	0.200549451	GO:0043168	GO:MF	anion binding	FALSE
1.36E-05	556	1766	131	0.074178935	0.235611511	GO:0030674	GO:MF	protein-macromolecule adaptor activity	TRUE
3.44E-05	1446	1766	287	0.162514156	0.198478562	GO:1901363	GO:MF	heterocyclic compound binding	FALSE
6.43E-05	1388	1766	275	0.155719139	0.198126801	GO:1901265	GO:MF	nucleoside phosphate binding	FALSE
6.43E-05	1376	1766	273	0.154586636	0.198401163	GO:0000166	GO:MF	nucleotide binding	FALSE
7.61E-05	619	1766	139	0.078708947	0.224555735	GO:0060090	GO:MF	molecular adaptor activity	FALSE
0.000286901	1197	1766	238	0.134767837	0.198830409	GO:0035639	GO:MF	purine ribonucleoside triphosphate binding	FALSE
0.000364447	1272	1766	250	0.141562854	0.196540881	GO:0017076	GO:MF	purine nucleotide binding	FALSE
0.000526229	698	1766	149	0.084371461	0.213467049	GO:0016772	GO:MF	transferase activity, transferring phosphorus-containing groups	FALSE
0.000851109	1229	1766	240	0.13590034	0.195280716	GO:0032553	GO:MF	ribonucleotide binding	FALSE
0.001017775	1657	1766	311	0.17610419	0.187688594	GO:0016787	GO:MF	hydrolase activity	FALSE
0.001017775	1222	1766	238	0.134767837	0.194762684	GO:0032555	GO:MF	purine ribonucleotide binding	FALSE
0.001130523	1337	1766	257	0.145526614	0.192221391	GO:0097367	GO:MF	carbohydrate derivative binding	FALSE
0.001380524	136	1766	40	0.022650057	0.294117647	GO:0016757	GO:MF	glycosyltransferase activity	FALSE
0.001380524	1702	1766	317	0.179501699	0.186251469	GO:0046872	GO:MF	metal ion binding	FALSE
0.00151487	1735	1766	322	0.182332956	0.185590778	GO:0043169	GO:MF	cation binding	FALSE
0.003325142	993	1766	195	0.110419026	0.196374622	GO:0042802	GO:MF	identical protein binding	FALSE
0.003805055	594	1766	125	0.070781427	0.21043771	GO:0016301	GO:MF	kinase activity	FALSE
0.003805055	549	1766	117	0.066251416	0.213114754	GO:0016773	GO:MF	phosphotransferase activity, alcohol group as acceptor	FALSE
0.004592549	387	1766	87	0.049263873	0.224806202	GO:0019900	GO:MF	kinase binding	FALSE
0.004592549	344	1766	79	0.044733862	0.229651163	GO:0019901	GO:MF	protein kinase binding	FALSE
0.004855097	199	1766	51	0.028878822	0.256281407	GO:0061629	GO:MF	RNA polymerase II-specific DNA-binding transcription factor binding	FALSE
0.005679391	320	1766	74	0.041902605	0.23125	GO:0019904	GO:MF	protein domain specific binding	FALSE
0.006116947	937	1766	183	0.103624009	0.195304162	GO:0030554	GO:MF	adenyl nucleotide binding	FALSE

0.008294161	472	1766	101	0.057191393	0.213983051	GO:0046983	GO:MF	protein dimerization activity	FALSE
0.00903298	418	1766	91	0.051528879	0.217703349	GO:0016746	GO:MF	acyltransferase activity	FALSE
0.009726457	876	1766	171	0.096828992	0.195205479	GO:0005524	GO:MF	ATP binding	FALSE
0.010836426	89	1766	27	0.015288788	0.303370787	GO:0016758	GO:MF	hexosyltransferase activity	FALSE
0.011491945	254	1766	60	0.033975085	0.236220472	GO:0004842	GO:MF	ubiquitin-protein transferase activity	FALSE
0.012274092	473	1766	100	0.056625142	0.21141649	GO:0004672	GO:MF	protein kinase activity	FALSE
0.015304057	316	1766	71	0.040203851	0.224683544	GO:0008134	GO:MF	transcription factor binding	FALSE
0.017035224	888	1766	171	0.096828992	0.192567568	GO:0032559	GO:MF	adenyl ribonucleotide binding	FALSE
0.020448926	192	1766	47	0.026613817	0.244791667	GO:0019003	GO:MF	GDP binding	FALSE
0.021849915	648	1766	129	0.073046433	0.199074074	GO:0016817	GO:MF	hydrolase activity, acting on acid anhydrides	FALSE
0.021849915	648	1766	129	0.073046433	0.199074074	GO:0016818	GO:MF	hydrolase activity, acting on acid anhydrides, in phosphorus-containing anhydrides	FALSE
0.023936567	644	1766	128	0.072480181	0.198757764	GO:0016462	GO:MF	pyrophosphatase activity	FALSE
0.026542272	253	1766	58	0.032842582	0.229249012	GO:0003712	GO:MF	transcription coregulator activity	FALSE
0.031072665	271	1766	61	0.034541336	0.225092251	GO:0019787	GO:MF	ubiquitin-like protein transferase activity	FALSE
0.031072665	26	1766	11	0.006228766	0.423076923	GO:0019905	GO:MF	syntaxin binding	TRUE
0.031211409	266	1766	60	0.033975085	0.22556391	GO:0140297	GO:MF	DNA-binding transcription factor binding	FALSE
0.036895608	279	1766	62	0.035107588	0.222222222	GO:0016755	GO:MF	aminoacyltransferase activity	FALSE
0.036895608	984	1766	184	0.10419026	0.18699187	GO:0003677	GO:MF	DNA binding	TRUE
0.03931151	346	1766	74	0.041902605	0.213872832	GO:0042803	GO:MF	protein homodimerization activity	FALSE
0.03931151	16	1766	8	0.004530011	0.5	GO:0000030	GO:MF	mannosyltransferase activity	FALSE
0.040714669	169	1766	41	0.023216308	0.24260355	GO:0061630	GO:MF	ubiquitin protein ligase activity	FALSE
0.040714669	336	1766	72	0.040770102	0.214285714	GO:0005525	GO:MF	GTP binding	FALSE
0.040714669	615	1766	121	0.068516421	0.196747967	GO:0017111	GO:MF	ribonucleoside triphosphate phosphatase activity	FALSE
0.044614819	664	1766	129	0.073046433	0.194277108	GO:0140110	GO:MF	transcription regulator activity	FALSE
7.49E-07	4554	1766	804	0.455266138	0.17654809	KEGG:00000	KEGG	KEGG root term	FALSE
0.009749683	138	1766	39	0.022083805	0.282608696	KEGG:04140	KEGG	Autophagy - animal	FALSE
0.016081281	188	1766	48	0.027180068	0.255319149	KEGG:05132	KEGG	Salmonella infection	FALSE
0.03338475	92	1766	27	0.015288788	0.293478261	KEGG:04919	KEGG	Thyroid hormone signaling pathway	FALSE
0.048811273	909	1766	172	0.097395243	0.189218922	KEGG:01100	KEGG	Metabolic pathways	FALSE
0.048811273	101	1766	28	0.01585504	0.277227723	KEGG:04142	KEGG	Lysosome	FALSE

**Brown Module GO Enrichment**									
p value	*term size*	*query size*	*overlap size*	*precision*	*recall*	*term id*	*source*	*term name*	*highlighted*
5.85E-19	524	951	113	0.118822292	0.215648855	GO:0003008	GO:BP	system process	TRUE
3.19E-18	1054	951	176	0.185068349	0.166982922	GO:0048513	GO:BP	animal organ development	FALSE
8.38E-18	2247	951	299	0.314405889	0.133066311	GO:0048856	GO:BP	anatomical structure development	FALSE
8.38E-18	2522	951	326	0.342797056	0.12926249	GO:0032501	GO:BP	multicellular organismal process	FALSE

8.38E-18	2409	951	315	0.331230284	0.130759651	GO:0032502	GO:BP	developmental process	FALSE
3.00E-17	485	951	103	0.108307045	0.212371134	GO:0022414	GO:BP	reproductive process	TRUE
1.73E-16	1427	951	211	0.221871714	0.147862649	GO:0048731	GO:BP	system development	FALSE
5.48E-16	297	951	74	0.077812829	0.249158249	GO:0050877	GO:BP	nervous system process	FALSE
5.75E-15	76	951	34	0.03575184	0.447368421	GO:0003341	GO:BP	cilium movement	FALSE
1.08E-14	429	951	90	0.094637224	0.20979021	GO:0006811	GO:BP	monoatomic ion transport	FALSE
1.08E-14	616	951	114	0.119873817	0.185064935	GO:0030030	GO:BP	cell projection organization	FALSE
3.17E-14	334	951	76	0.079915878	0.22754491	GO:0034220	GO:BP	monoatomic ion transmembrane transport	FALSE
4.98E-14	606	951	111	0.116719243	0.183168317	GO:0120036	GO:BP	plasma membrane bounded cell projection organization	FALSE
4.98E-14	1037	951	161	0.169295478	0.155255545	GO:0009653	GO:BP	anatomical structure morphogenesis	FALSE
2.35E-13	132	951	43	0.045215563	0.325757576	GO:0007600	GO:BP	sensory perception	FALSE
5.60E-13	113	951	39	0.041009464	0.345132743	GO:0043062	GO:BP	extracellular structure organization	TRUE
8.27E-13	1745	951	231	0.242902208	0.132378223	GO:0007275	GO:BP	multicellular organism development	FALSE
1.12E-12	1602	951	216	0.227129338	0.134831461	GO:0030154	GO:BP	cell differentiation	FALSE
1.12E-12	1602	951	216	0.227129338	0.134831461	GO:0048869	GO:BP	cellular developmental process	FALSE
1.45E-12	459	951	89	0.093585699	0.193899782	GO:0030182	GO:BP	neuron differentiation	FALSE
1.48E-12	580	951	104	0.10935857	0.179310345	GO:0055085	GO:BP	transmembrane transport	FALSE
1.70E-12	112	951	38	0.039957939	0.339285714	GO:0030198	GO:BP	extracellular matrix organization	FALSE
1.70E-12	112	951	38	0.039957939	0.339285714	GO:0045229	GO:BP	external encapsulating structure organization	FALSE
2.12E-12	356	951	75	0.078864353	0.210674157	GO:0019953	GO:BP	sexual reproduction	FALSE
8.93E-12	1123	951	163	0.171398528	0.145146928	GO:0048468	GO:BP	cell development	FALSE
1.02E-11	91	951	33	0.034700315	0.362637363	GO:0007286	GO:BP	spermatid development	FALSE
1.07E-11	191	951	50	0.052576236	0.261780105	GO:0007018	GO:BP	microtubule-based movement	FALSE
1.10E-11	113	951	37	0.038906414	0.327433628	GO:0042391	GO:BP	regulation of membrane potential	FALSE
1.10E-11	485	951	90	0.094637224	0.18556701	GO:0048699	GO:BP	generation of neurons	FALSE
1.47E-11	206	951	52	0.054679285	0.252427184	GO:0048232	GO:BP	male gamete generation	FALSE
1.53E-11	386	951	77	0.080967403	0.199481865	GO:0048666	GO:BP	neuron development	FALSE
1.82E-11	59	951	26	0.027339642	0.440677966	GO:0050954	GO:BP	sensory perception of mechanical stimulus	FALSE
2.12E-11	64	951	27	0.028391167	0.421875	GO:0060285	GO:BP	cilium-dependent cell motility	FALSE
2.12E-11	64	951	27	0.028391167	0.421875	GO:0001539	GO:BP	cilium or flagellum-dependent cell motility	FALSE
2.21E-11	94	951	33	0.034700315	0.35106383	GO:0048515	GO:BP	spermatid differentiation	FALSE
3.02E-11	561	951	98	0.103049422	0.174688057	GO:0007155	GO:BP	cell adhesion	TRUE
6.58E-11	62	951	26	0.027339642	0.419354839	GO:0060294	GO:BP	cilium movement involved in cell motility	FALSE
8.07E-11	308	951	65	0.068349106	0.211038961	GO:0098660	GO:BP	inorganic ion transmembrane transport	FALSE
1.10E-10	2476	951	292	0.307045216	0.117932149	GO:0007154	GO:BP	cell communication	FALSE
1.26E-10	59	951	25	0.026288118	0.423728814	GO:0030317	GO:BP	flagellated sperm motility	FALSE
1.26E-10	59	951	25	0.026288118	0.423728814	GO:0097722	GO:BP	sperm motility	FALSE
1.31E-10	34	951	19	0.01997897	0.558823529	GO:0044458	GO:BP	motile cilium assembly	FALSE
2.09E-10	194	951	48	0.050473186	0.24742268	GO:0007283	GO:BP	spermatogenesis	FALSE
9.03E-10	41	951	20	0.021030494	0.487804878	GO:0001508	GO:BP	action potential	FALSE
1.16E-09	327	951	65	0.068349106	0.198776758	GO:0003006	GO:BP	developmental process involved in reproduction	FALSE

1.18E-09	282	951	59	0.062039958	0.209219858	GO:0098655	GO:BP	monoatomic cation transmembrane transport	FALSE
1.21E-09	275	951	58	0.060988433	0.210909091	GO:0007276	GO:BP	gamete generation	FALSE
1.21E-09	351	951	68	0.07150368	0.193732194	GO:0009887	GO:BP	animal organ morphogenesis	FALSE
1.54E-09	34	951	18	0.018927445	0.529411765	GO:0035082	GO:BP	axoneme assembly	FALSE
1.58E-09	307	951	62	0.065194532	0.201954397	GO:0048609	GO:BP	multicellular organismal reproductive process	FALSE
2.09E-09	193	951	46	0.048370137	0.238341969	GO:0060271	GO:BP	cilium assembly	FALSE
3.77E-09	143	951	38	0.039957939	0.265734266	GO:0007281	GO:BP	germ cell development	FALSE
5.15E-09	205	951	47	0.049421661	0.229268293	GO:0044782	GO:BP	cilium organization	FALSE
5.73E-09	2466	951	283	0.297581493	0.114760746	GO:0023052	GO:BP	signaling	FALSE
6.59E-09	365	951	68	0.07150368	0.18630137	GO:0006812	GO:BP	monoatomic cation transport	FALSE
8.35E-09	194	951	45	0.047318612	0.231958763	GO:0007423	GO:BP	sensory organ development	FALSE
9.31E-09	839	951	122	0.128286015	0.145411204	GO:0007399	GO:BP	nervous system development	FALSE
1.17E-08	11	951	10	0.010515247	0.909090909	GO:0086010	GO:BP	membrane depolarization during action potential	FALSE
1.17E-08	284	951	57	0.059936909	0.200704225	GO:0120031	GO:BP	plasma membrane bounded cell projection assembly	FALSE
1.22E-08	149	951	38	0.039957939	0.255033557	GO:0022412	GO:BP	cellular process involved in reproduction in multicellular organism	FALSE
1.50E-08	286	951	57	0.059936909	0.199300699	GO:0030031	GO:BP	cell projection assembly	FALSE
1.76E-08	272	951	55	0.057833859	0.202205882	GO:0034330	GO:BP	cell junction organization	TRUE
1.86E-08	693	951	105	0.110410095	0.151515152	GO:0048870	GO:BP	cell motility	FALSE
2.45E-08	598	951	94	0.098843323	0.157190635	GO:0022008	GO:BP	neurogenesis	FALSE
3.96E-08	24	951	14	0.014721346	0.583333333	GO:0120316	GO:BP	sperm flagellum assembly	FALSE
4.41E-08	123	951	33	0.034700315	0.268292683	GO:0072001	GO:BP	renal system development	FALSE
4.96E-08	311	951	59	0.062039958	0.189710611	GO:0030001	GO:BP	metal ion transport	FALSE
5.44E-08	468	951	78	0.082018927	0.166666667	GO:0072359	GO:BP	circulatory system development	FALSE
6.23E-08	274	951	54	0.056782334	0.197080292	GO:0098662	GO:BP	inorganic cation transmembrane transport	FALSE
6.80E-08	46	951	19	0.01997897	0.413043478	GO:0001578	GO:BP	microtubule bundle formation	FALSE
7.20E-08	51	951	20	0.021030494	0.392156863	GO:0007127	GO:BP	meiosis I	FALSE
7.20E-08	51	951	20	0.021030494	0.392156863	GO:0007605	GO:BP	sensory perception of sound	FALSE
1.09E-07	744	951	108	0.113564669	0.14516129	GO:0009888	GO:BP	tissue development	FALSE
1.56E-07	53	951	20	0.021030494	0.377358491	GO:0061982	GO:BP	meiosis I cell cycle process	FALSE
1.63E-07	480	951	78	0.082018927	0.1625	GO:0007017	GO:BP	microtubule-based process	FALSE
1.81E-07	3470	951	365	0.383806519	0.10518732	GO:0050896	GO:BP	response to stimulus	FALSE
2.09E-07	16	951	11	0.011566772	0.6875	GO:0086065	GO:BP	cell communication involved in cardiac conduction	FALSE
3.96E-07	2472	951	274	0.288117771	0.110841424	GO:0051179	GO:BP	localization	FALSE
4.00E-07	108	951	29	0.030494217	0.268518519	GO:0051321	GO:BP	meiotic cell cycle	FALSE
4.06E-07	2115	951	241	0.253417455	0.113947991	GO:0051234	GO:BP	establishment of localization	FALSE
4.76E-07	206	951	43	0.045215563	0.208737864	GO:0048667	GO:BP	cell morphogenesis involved in neuron differentiation	FALSE
4.76E-07	331	951	59	0.062039958	0.178247734	GO:0031175	GO:BP	neuron projection development	FALSE
5.36E-07	62	951	21	0.022082019	0.338709677	GO:0006820	GO:BP	monoatomic anion transport	FALSE
6.97E-07	33	951	15	0.015772871	0.454545455	GO:0070252	GO:BP	actin-mediated cell contraction	FALSE
8.17E-07	118	951	30	0.031545741	0.254237288	GO:0001822	GO:BP	kidney development	FALSE

1.09E-06	70	951	22	0.023133544	0.314285714	GO:0097485	GO:BP	neuron projection guidance	FALSE
1.09E-06	70	951	22	0.023133544	0.314285714	GO:0007411	GO:BP	axon guidance	FALSE
1.96E-06	84	951	24	0.025236593	0.285714286	GO:0140013	GO:BP	meiotic nuclear division	FALSE
1.96E-06	50	951	18	0.018927445	0.36	GO:0035725	GO:BP	sodium ion transmembrane transport	FALSE
2.49E-06	73	951	22	0.023133544	0.301369863	GO:0006814	GO:BP	sodium ion transport	FALSE
2.94E-06	46	951	17	0.01787592	0.369565217	GO:0030048	GO:BP	actin filament-based movement	FALSE
2.95E-06	92	951	25	0.026288118	0.27173913	GO:0090596	GO:BP	sensory organ morphogenesis	FALSE
3.16E-06	153	951	34	0.03575184	0.222222222	GO:0003013	GO:BP	circulatory system process	FALSE
3.17E-06	146	951	33	0.034700315	0.226027397	GO:0001654	GO:BP	eye development	FALSE
4.39E-06	148	951	33	0.034700315	0.222972973	GO:0048880	GO:BP	sensory system development	FALSE
4.39E-06	148	951	33	0.034700315	0.222972973	GO:0150063	GO:BP	visual system development	FALSE
5.28E-06	389	951	63	0.066246057	0.161953728	GO:0000902	GO:BP	cell morphogenesis	FALSE
7.02E-06	29	951	13	0.013669821	0.448275862	GO:0061337	GO:BP	cardiac conduction	FALSE
7.02E-06	29	951	13	0.013669821	0.448275862	GO:0007156	GO:BP	homophilic cell adhesion via plasma membrane adhesion molecules	FALSE
7.55E-06	17	951	10	0.010515247	0.588235294	GO:0086002	GO:BP	cardiac muscle cell action potential involved in contraction	FALSE
7.55E-06	17	951	10	0.010515247	0.588235294	GO:0003351	GO:BP	epithelial cilium movement involved in extracellular fluid movement	FALSE
8.07E-06	25	951	12	0.012618297	0.48	GO:0007129	GO:BP	homologous chromosome pairing at meiosis	FALSE
8.07E-06	97	951	25	0.026288118	0.257731959	GO:1903046	GO:BP	meiotic cell cycle process	FALSE
8.07E-06	25	951	12	0.012618297	0.48	GO:0086003	GO:BP	cardiac muscle cell contraction	FALSE
8.45E-06	21	951	11	0.011566772	0.523809524	GO:0086001	GO:BP	cardiac muscle cell action potential	FALSE
8.77E-06	8	951	7	0.007360673	0.875	GO:0086069	GO:BP	bundle of His cell to Purkinje myocyte communication	FALSE
8.96E-06	221	951	42	0.044164038	0.190045249	GO:0048812	GO:BP	neuron projection morphogenesis	FALSE
1.02E-05	5049	951	488	0.513144059	0.096652803	GO:0065007	GO:BP	biological regulation	FALSE
1.03E-05	30	951	13	0.013669821	0.433333333	GO:0009581	GO:BP	detection of external stimulus	FALSE
1.03E-05	30	951	13	0.013669821	0.433333333	GO:0009582	GO:BP	detection of abiotic stimulus	FALSE
1.21E-05	155	951	33	0.034700315	0.212903226	GO:0050808	GO:BP	synapse organization	FALSE
1.30E-05	141	951	31	0.032597266	0.219858156	GO:0008015	GO:BP	blood circulation	FALSE
1.61E-05	447	951	68	0.07150368	0.15212528	GO:0060429	GO:BP	epithelium development	FALSE
1.63E-05	172	951	35	0.036803365	0.203488372	GO:0061564	GO:BP	axon development	FALSE
1.68E-05	377	951	60	0.063091483	0.159151194	GO:0007167	GO:BP	enzyme-linked receptor protein signaling pathway	FALSE
1.70E-05	2314	951	250	0.262881178	0.108038029	GO:0007165	GO:BP	signal transduction	FALSE
1.74E-05	227	951	42	0.044164038	0.185022026	GO:0007507	GO:BP	heart development	FALSE
1.74E-05	309	951	52	0.054679285	0.16828479	GO:0001568	GO:BP	blood vessel development	FALSE
1.76E-05	52	951	17	0.01787592	0.326923077	GO:0098656	GO:BP	monoatomic anion transmembrane transport	FALSE
1.78E-05	129	951	29	0.030494217	0.224806202	GO:0043010	GO:BP	camera-type eye development	FALSE
1.79E-05	449	951	68	0.07150368	0.151447661	GO:0035295	GO:BP	tube development	FALSE
1.96E-05	27	951	12	0.012618297	0.444444444	GO:0051899	GO:BP	membrane depolarization	FALSE
2.03E-05	1938	951	215	0.226077813	0.110939112	GO:0006810	GO:BP	transport	FALSE

2.03E-05	328	951	54	0.056782334	0.164634146	GO:0001944	GO:BP	vasculature development	FALSE
2.10E-05	15	951	9	0.009463722	0.6	GO:0007288	GO:BP	sperm axoneme assembly	FALSE
2.16E-05	2949	951	306	0.321766562	0.103763988	GO:0051716	GO:BP	cellular response to stimulus	FALSE
2.48E-05	65	951	19	0.01997897	0.292307692	GO:0015698	GO:BP	inorganic anion transport	FALSE
2.62E-05	231	951	42	0.044164038	0.181818182	GO:0120039	GO:BP	plasma membrane bounded cell projection morphogenesis	FALSE
2.89E-05	12	951	8	0.008412198	0.666666667	GO:0050974	GO:BP	detection of mechanical stimulus involved in sensory perception	FALSE
2.91E-05	232	951	42	0.044164038	0.181034483	GO:0048858	GO:BP	cell projection morphogenesis	FALSE
2.94E-05	9	951	7	0.007360673	0.777777778	GO:0048265	GO:BP	response to pain	FALSE
2.94E-05	28	951	12	0.012618297	0.428571429	GO:0045143	GO:BP	homologous chromosome segregation	FALSE
2.95E-05	43	951	15	0.015772871	0.348837209	GO:0050953	GO:BP	sensory perception of light stimulus	FALSE
3.21E-05	33	951	13	0.013669821	0.393939394	GO:0070192	GO:BP	chromosome organization involved in meiotic cell cycle	FALSE
3.35E-05	163	951	33	0.034700315	0.202453988	GO:0031589	GO:BP	cell-substrate adhesion	FALSE
3.65E-05	1080	951	132	0.138801262	0.122222222	GO:0065008	GO:BP	regulation of biological quality	FALSE
4.21E-05	20	951	10	0.010515247	0.5	GO:0006858	GO:BP	extracellular transport	FALSE
4.74E-05	158	951	32	0.033648791	0.202531646	GO:0007409	GO:BP	axonogenesis	FALSE
4.92E-05	56	951	17	0.01787592	0.303571429	GO:0098742	GO:BP	cell-cell adhesion via plasma-membrane adhesion molecules	FALSE
6.75E-05	30	951	12	0.012618297	0.4	GO:0002027	GO:BP	regulation of heart rate	FALSE
7.30E-05	124	951	27	0.028391167	0.217741935	GO:0003012	GO:BP	muscle system process	FALSE
8.02E-05	209	951	38	0.039957939	0.181818182	GO:0007610	GO:BP	behavior	FALSE
8.04E-05	64	951	18	0.018927445	0.28125	GO:0048592	GO:BP	eye morphogenesis	FALSE
8.04E-05	64	951	18	0.018927445	0.28125	GO:0060041	GO:BP	retina development in camera-type eye	FALSE
8.75E-05	462	951	67	0.070452156	0.145021645	GO:0048646	GO:BP	anatomical structure formation involved in morphogenesis	FALSE
0.000104507	236	951	41	0.043112513	0.173728814	GO:0048729	GO:BP	tissue morphogenesis	FALSE
0.000117983	22	951	10	0.010515247	0.454545455	GO:0033555	GO:BP	multicellular organismal response to stress	FALSE
0.000124389	135	951	28	0.029442692	0.207407407	GO:0044057	GO:BP	regulation of system process	FALSE
0.000130901	14	951	8	0.008412198	0.571428571	GO:0050982	GO:BP	detection of mechanical stimulus	FALSE
0.000131676	315	951	50	0.052576236	0.158730159	GO:0007267	GO:BP	cell-cell signaling	FALSE
0.000134667	18	951	9	0.009463722	0.5	GO:0045494	GO:BP	photoreceptor cell maintenance	FALSE
0.000137987	198	951	36	0.03785489	0.181818182	GO:0099536	GO:BP	synaptic signaling	FALSE
0.000154872	670	951	88	0.092534175	0.131343284	GO:0007010	GO:BP	cytoskeleton organization	FALSE
0.000165777	49	951	15	0.015772871	0.306122449	GO:0060048	GO:BP	cardiac muscle contraction	FALSE
0.00019845	11	951	7	0.007360673	0.636363636	GO:0015701	GO:BP	bicarbonate transport	FALSE
0.000201234	28	951	11	0.011566772	0.392857143	GO:0050906	GO:BP	detection of stimulus involved in sensory perception	FALSE
0.000201234	102	951	23	0.024185068	0.225490196	GO:0006936	GO:BP	muscle contraction	FALSE
0.000211704	75	951	19	0.01997897	0.253333333	GO:0003015	GO:BP	heart process	FALSE
0.00021208	56	951	16	0.016824395	0.285714286	GO:0048839	GO:BP	inner ear development	FALSE
0.000222577	19	951	9	0.009463722	0.473684211	GO:0140527	GO:BP	reciprocal homologous recombination	FALSE

0.000222577	19	951	9	0.009463722	0.473684211	GO:0007131	GO:BP	reciprocal meiotic recombination	FALSE
0.000228709	8	951	6	0.006309148	0.75	GO:0062149	GO:BP	detection of stimulus involved in sensory perception of pain	FALSE
0.000228709	8	951	6	0.006309148	0.75	GO:0086019	GO:BP	cell-cell signaling involved in cardiac conduction	FALSE
0.000287549	29	951	11	0.011566772	0.379310345	GO:0042490	GO:BP	mechanoreceptor differentiation	FALSE
0.000289999	119	951	25	0.026288118	0.210084034	GO:0070588	GO:BP	calcium ion transmembrane transport	FALSE
0.000316919	40	951	13	0.013669821	0.325	GO:0007601	GO:BP	visual perception	FALSE
0.000317869	150	951	29	0.030494217	0.193333333	GO:0007178	GO:BP	cell surface receptor protein serine/threonine kinase signaling pathway	FALSE
0.000329702	58	951	16	0.016824395	0.275862069	GO:0006941	GO:BP	striated muscle contraction	FALSE
0.000341283	71	951	18	0.018927445	0.253521127	GO:0060047	GO:BP	heart contraction	FALSE
0.000350543	4906	951	464	0.487907466	0.094578068	GO:0050789	GO:BP	regulation of biological process	FALSE
0.000353418	20	951	9	0.009463722	0.45	GO:0035825	GO:BP	homologous recombination	FALSE
0.000398827	12	951	7	0.007360673	0.583333333	GO:0032197	GO:BP	retrotransposition	TRUE
0.000403614	25	951	10	0.010515247	0.4	GO:0060042	GO:BP	retina morphogenesis in camera-type eye	FALSE
0.000409864	41	951	13	0.013669821	0.317073171	GO:0006821	GO:BP	chloride transport	FALSE
0.000411588	16	951	8	0.008412198	0.5	GO:0086091	GO:BP	regulation of heart rate by cardiac conduction	FALSE
0.000421636	53	951	15	0.015772871	0.283018868	GO:0048593	GO:BP	camera-type eye morphogenesis	FALSE
0.000492886	194	951	34	0.03575184	0.175257732	GO:0002009	GO:BP	morphogenesis of an epithelium	FALSE
0.000493589	146	951	28	0.029442692	0.191780822	GO:0015711	GO:BP	organic anion transport	FALSE
0.000498073	236	951	39	0.041009464	0.165254237	GO:0030855	GO:BP	epithelial cell differentiation	FALSE
0.000528171	54	951	15	0.015772871	0.277777778	GO:0051606	GO:BP	detection of stimulus	FALSE
0.00053904	21	951	9	0.009463722	0.428571429	GO:0001895	GO:BP	retina homeostasis	FALSE
0.00053904	48	951	14	0.014721346	0.291666667	GO:0072006	GO:BP	nephron development	FALSE
0.000568072	9	951	6	0.006309148	0.666666667	GO:0050951	GO:BP	sensory perception of temperature stimulus	FALSE
0.000568072	6	951	5	0.005257624	0.833333333	GO:0141005	GO:BP	transposable element silencing by heterochromatin formation	FALSE
0.000568072	6	951	5	0.005257624	0.833333333	GO:0140966	GO:BP	piRNA-mediated heterochromatin formation	FALSE
0.000568072	6	951	5	0.005257624	0.833333333	GO:0015721	GO:BP	bile acid and bile salt transport	TRUE
0.000590824	264	951	42	0.044164038	0.159090909	GO:0048514	GO:BP	blood vessel morphogenesis	FALSE
0.000594809	344	951	51	0.05362776	0.148255814	GO:0030029	GO:BP	actin filament-based process	FALSE
0.000722138	111	951	23	0.024185068	0.207207207	GO:0007160	GO:BP	cell-matrix adhesion	FALSE
0.00072594	13	951	7	0.007360673	0.538461538	GO:0032196	GO:BP	transposition	FALSE
0.00072594	62	951	16	0.016824395	0.258064516	GO:0043583	GO:BP	ear development	FALSE
0.000728532	190	951	33	0.034700315	0.173684211	GO:0099537	GO:BP	trans-synaptic signaling	FALSE
0.000780527	69	951	17	0.01787592	0.246376812	GO:1903522	GO:BP	regulation of blood circulation	FALSE
0.000818152	112	951	23	0.024185068	0.205357143	GO:0071560	GO:BP	cellular response to transforming growth factor beta stimulus	FALSE
0.000918117	98	951	21	0.022082019	0.214285714	GO:0007179	GO:BP	transforming growth factor beta receptor signaling pathway	FALSE

0.000966588	152	951	28	0.029442692	0.184210526	GO:0034329	GO:BP	cell junction assembly	FALSE
0.000974536	57	951	15	0.015772871	0.263157895	GO:0008016	GO:BP	regulation of heart contraction	FALSE
0.001031841	129	951	25	0.026288118	0.19379845	GO:0007389	GO:BP	pattern specification process	FALSE
0.001085681	45	951	13	0.013669821	0.288888889	GO:0098661	GO:BP	inorganic anion transmembrane transport	FALSE
0.001085681	45	951	13	0.013669821	0.288888889	GO:0045132	GO:BP	meiotic chromosome segregation	FALSE
0.001111333	28	951	10	0.010515247	0.357142857	GO:0060113	GO:BP	inner ear receptor cell differentiation	FALSE
0.00114749	4	951	4	0.004206099	1	GO:0051026	GO:BP	chiasma assembly	FALSE
0.001192621	10	951	6	0.006309148	0.6	GO:0010526	GO:BP	transposable element silencing	FALSE
0.00121514	187	951	32	0.033648791	0.171122995	GO:0098916	GO:BP	anterograde trans-synaptic signaling	FALSE
0.00121514	187	951	32	0.033648791	0.171122995	GO:0007268	GO:BP	chemical synaptic transmission	FALSE
0.001286841	346	951	50	0.052576236	0.144508671	GO:0098609	GO:BP	cell-cell adhesion	FALSE
0.001286841	34	951	11	0.011566772	0.323529412	GO:1902476	GO:BP	chloride transmembrane transport	FALSE
0.001364983	116	951	23	0.024185068	0.198275862	GO:0071559	GO:BP	response to transforming growth factor beta	FALSE
0.001390324	338	951	49	0.051524711	0.144970414	GO:0000226	GO:BP	microtubule cytoskeleton organization	FALSE
0.001390427	725	951	89	0.093585699	0.122758621	GO:0042592	GO:BP	homeostatic process	TRUE
0.001506571	66	951	16	0.016824395	0.242424242	GO:0099173	GO:BP	postsynapse organization	FALSE
0.001544958	358	951	51	0.05362776	0.142458101	GO:0035239	GO:BP	tube morphogenesis	FALSE
0.001578084	250	951	39	0.041009464	0.156	GO:0007420	GO:BP	brain development	FALSE
0.001597917	157	951	28	0.029442692	0.178343949	GO:0006816	GO:BP	calcium ion transport	FALSE
0.001597917	7	951	5	0.005257624	0.714285714	GO:0090660	GO:BP	cerebrospinal fluid circulation	FALSE
0.001597917	7	951	5	0.005257624	0.714285714	GO:0048266	GO:BP	behavioral response to pain	FALSE
0.001609995	141	951	26	0.027339642	0.184397163	GO:0141091	GO:BP	transforming growth factor beta receptor superfamily signaling pathway	FALSE
0.001770314	103	951	21	0.022082019	0.203883495	GO:0015849	GO:BP	organic acid transport	FALSE
0.001770314	103	951	21	0.022082019	0.203883495	GO:0046942	GO:BP	carboxylic acid transport	FALSE
0.002033831	15	951	7	0.007360673	0.466666667	GO:0098900	GO:BP	regulation of action potential	FALSE
0.002077988	1086	951	123	0.129337539	0.113259669	GO:0007166	GO:BP	cell surface receptor signaling pathway	FALSE
0.002232941	11	951	6	0.006309148	0.545454545	GO:0098901	GO:BP	regulation of cardiac muscle cell action potential	FALSE
0.002528957	145	951	26	0.027339642	0.179310345	GO:0099177	GO:BP	regulation of trans-synaptic signaling	FALSE
0.002528957	145	951	26	0.027339642	0.179310345	GO:0050804	GO:BP	modulation of chemical synaptic transmission	FALSE
0.003172535	26	951	9	0.009463722	0.346153846	GO:0001738	GO:BP	morphogenesis of a polarized epithelium	FALSE
0.003172535	26	951	9	0.009463722	0.346153846	GO:0045669	GO:BP	positive regulation of osteoblast differentiation	TRUE
0.00325741	16	951	7	0.007360673	0.4375	GO:0019226	GO:BP	transmission of nerve impulse	FALSE
0.003304056	613	951	76	0.079915878	0.123980424	GO:0016477	GO:BP	cell migration	FALSE
0.00345603	278	951	41	0.043112513	0.147482014	GO:0060322	GO:BP	head development	FALSE

0.003695081	8	951	5	0.005257624	0.625	GO:0038063	GO:BP	collagen-activated tyrosine kinase receptor signaling pathway	TRUE
0.003695081	8	951	5	0.005257624	0.625	GO:0098911	GO:BP	regulation of ventricular cardiac muscle cell action potential	FALSE
0.003695081	8	951	5	0.005257624	0.625	GO:0034587	GO:BP	piRNA processing	TRUE
0.003960634	12	951	6	0.006309148	0.5	GO:0086005	GO:BP	ventricular cardiac muscle cell action potential	FALSE
0.004211171	27	951	9	0.009463722	0.333333333	GO:0061326	GO:BP	renal tubule development	FALSE
0.004211171	27	951	9	0.009463722	0.333333333	GO:1901890	GO:BP	positive regulation of cell junction assembly	FALSE
0.004211171	27	951	9	0.009463722	0.333333333	GO:0006835	GO:BP	dicarboxylic acid transport	FALSE
0.004211171	27	951	9	0.009463722	0.333333333	GO:0032835	GO:BP	glomerulus development	FALSE
0.004458421	5	951	4	0.004206099	0.8	GO:1903779	GO:BP	regulation of cardiac conduction	FALSE
0.004458421	5	951	4	0.004206099	0.8	GO:0086042	GO:BP	cardiac muscle cell-cardiac muscle cell adhesion	FALSE
0.004458421	5	951	4	0.004206099	0.8	GO:0141196	GO:BP	transposable element silencing by piRNA-mediated DNA methylation	FALSE
0.004458421	5	951	4	0.004206099	0.8	GO:0141176	GO:BP	gene silencing by piRNA-directed DNA methylation	FALSE
0.004458421	5	951	4	0.004206099	0.8	GO:0086073	GO:BP	bundle of His cell-Purkinje myocyte adhesion involved in cell communication	FALSE
0.004458421	5	951	4	0.004206099	0.8	GO:0050965	GO:BP	detection of temperature stimulus involved in sensory perception of pain	FALSE
0.004729145	972	951	110	0.115667718	0.113168724	GO:0050793	GO:BP	regulation of developmental process	FALSE
0.004871043	247	951	37	0.038906414	0.149797571	GO:0055080	GO:BP	monoatomic cation homeostasis	FALSE
0.005526301	40	951	11	0.011566772	0.275	GO:0021954	GO:BP	central nervous system neuron development	FALSE
0.005526301	28	951	9	0.009463722	0.321428571	GO:0007140	GO:BP	male meiotic nuclear division	FALSE
0.00580066	145	951	25	0.026288118	0.172413793	GO:0030900	GO:BP	forebrain development	FALSE
0.006097875	333	951	46	0.048370137	0.138138138	GO:0007417	GO:BP	central nervous system development	FALSE
0.006381728	13	951	6	0.006309148	0.461538462	GO:0006956	GO:BP	complement activation	TRUE
0.006508979	23	951	8	0.008412198	0.347826087	GO:0046530	GO:BP	photoreceptor cell differentiation	FALSE
0.006545892	251	951	37	0.038906414	0.147410359	GO:0050801	GO:BP	monoatomic ion homeostasis	FALSE
0.007027501	18	951	7	0.007360673	0.388888889	GO:0001754	GO:BP	eye photoreceptor cell differentiation	FALSE
0.007053932	9	951	5	0.005257624	0.555555556	GO:0019228	GO:BP	neuronal action potential	FALSE
0.007053932	9	951	5	0.005257624	0.555555556	GO:0006958	GO:BP	complement activation, classical pathway	FALSE
0.007859668	522	951	65	0.068349106	0.124521073	GO:0070925	GO:BP	organelle assembly	FALSE
0.008456323	1170	951	127	0.133543638	0.108547009	GO:0051239	GO:BP	regulation of multicellular organismal process	FALSE
0.00882323	24	951	8	0.008412198	0.333333333	GO:0099068	GO:BP	postsynapse assembly	FALSE
0.009404745	30	951	9	0.009463722	0.3	GO:0055117	GO:BP	regulation of cardiac muscle contraction	FALSE
0.009411893	150	951	25	0.026288118	0.166666667	GO:0014706	GO:BP	striated muscle tissue development	FALSE

0.009581663	109	951	20	0.021030494	0.183486239	GO:0001894	GO:BP	tissue homeostasis	FALSE
0.00961948	78	951	16	0.016824395	0.205128205	GO:0048738	GO:BP	cardiac muscle tissue development	FALSE
0.009846403	14	951	6	0.006309148	0.428571429	GO:1903115	GO:BP	regulation of actin filament-based movement	FALSE
0.009846403	14	951	6	0.006309148	0.428571429	GO:0086004	GO:BP	regulation of cardiac muscle cell contraction	FALSE
0.009877797	19	951	7	0.007360673	0.368421053	GO:0019233	GO:BP	sensory perception of pain	FALSE
0.010083531	239	951	35	0.036803365	0.146443515	GO:0120035	GO:BP	regulation of plasma membrane bounded cell projection organization	FALSE
0.010147056	3	951	3	0.003154574	1	GO:0007567	GO:BP	parturition	TRUE
0.010147056	110	951	20	0.021030494	0.181818182	GO:0060249	GO:BP	anatomical structure homeostasis	FALSE
0.010147056	3	951	3	0.003154574	1	GO:0060073	GO:BP	micturition	FALSE
0.010147056	3	951	3	0.003154574	1	GO:0086016	GO:BP	AV node cell action potential	FALSE
0.010147056	3	951	3	0.003154574	1	GO:0086027	GO:BP	AV node cell to bundle of His cell signaling	FALSE
0.010147056	3	951	3	0.003154574	1	GO:0032253	GO:BP	dense core granule localization	TRUE
0.010147056	3	951	3	0.003154574	1	GO:0086015	GO:BP	SA node cell action potential	FALSE
0.010147056	3	951	3	0.003154574	1	GO:0001710	GO:BP	mesodermal cell fate commitment	TRUE
0.010147056	3	951	3	0.003154574	1	GO:0086067	GO:BP	AV node cell to bundle of His cell communication	FALSE
0.010147056	3	951	3	0.003154574	1	GO:0141006	GO:BP	transposable element silencing by piRNA-mediated heterochromatin formation	FALSE
0.010147056	3	951	3	0.003154574	1	GO:0021889	GO:BP	olfactory bulb interneuron differentiation	FALSE
0.010147056	3	951	3	0.003154574	1	GO:0070445	GO:BP	regulation of oligodendrocyte progenitor proliferation	TRUE
0.010147056	3	951	3	0.003154574	1	GO:0086070	GO:BP	SA node cell to atrial cardiac muscle cell communication	FALSE
0.010147056	3	951	3	0.003154574	1	GO:0086018	GO:BP	SA node cell to atrial cardiac muscle cell signaling	FALSE
0.01032162	240	951	35	0.036803365	0.145833333	GO:0031344	GO:BP	regulation of cell projection organization	FALSE
0.010541653	401	951	52	0.054679285	0.12967581	GO:0048878	GO:BP	chemical homeostasis	FALSE
0.010625406	6	951	4	0.004206099	0.666666667	GO:0050961	GO:BP	detection of temperature stimulus involved in sensory perception	FALSE
0.010625406	6	951	4	0.004206099	0.666666667	GO:0016048	GO:BP	detection of temperature stimulus	FALSE
0.010625406	6	951	4	0.004206099	0.666666667	GO:0050910	GO:BP	detection of mechanical stimulus involved in sensory perception of sound	FALSE
0.010625406	6	951	4	0.004206099	0.666666667	GO:0120197	GO:BP	mucociliary clearance	FALSE
0.010835852	25	951	8	0.008412198	0.32	GO:0014855	GO:BP	striated muscle cell proliferation	TRUE
0.011003092	95	951	18	0.018927445	0.189473684	GO:0015850	GO:BP	organic hydroxy compound transport	FALSE
0.011491107	10	951	5	0.005257624	0.5	GO:0030318	GO:BP	melanocyte differentiation	TRUE

0.011491107	10	951	5	0.005257624	0.5	GO:0099084	GO:BP	postsynaptic specialization organization	FALSE
0.011491107	10	951	5	0.005257624	0.5	GO:0006857	GO:BP	oligopeptide transport	TRUE
0.011491107	10	951	5	0.005257624	0.5	GO:0038065	GO:BP	collagen-activated signaling pathway	FALSE
0.011551127	80	951	16	0.016824395	0.2	GO:1901888	GO:BP	regulation of cell junction assembly	FALSE
0.011698639	224	951	33	0.034700315	0.147321429	GO:0000280	GO:BP	nuclear division	FALSE
0.011698639	215	951	32	0.033648791	0.148837209	GO:0001525	GO:BP	angiogenesis	FALSE
0.011701685	58	951	13	0.013669821	0.224137931	GO:0006836	GO:BP	neurotransmitter transport	FALSE
0.013144707	81	951	16	0.016824395	0.197530864	GO:0016358	GO:BP	dendrite development	FALSE
0.013215147	244	951	35	0.036803365	0.143442623	GO:0071363	GO:BP	cellular response to growth factor stimulus	FALSE
0.013447074	15	951	6	0.006309148	0.4	GO:0001736	GO:BP	establishment of planar polarity	FALSE
0.013447074	15	951	6	0.006309148	0.4	GO:0007164	GO:BP	establishment of tissue polarity	FALSE
0.013637154	45	951	11	0.011566772	0.244444444	GO:0015718	GO:BP	monocarboxylic acid transport	FALSE
0.013882559	52	951	12	0.012618297	0.230769231	GO:0035265	GO:BP	organ growth	FALSE
0.014351402	4750	951	435	0.457413249	0.091578947	GO:0050794	GO:BP	regulation of cellular process	FALSE
0.014588071	156	951	25	0.026288118	0.16025641	GO:0060537	GO:BP	muscle tissue development	FALSE
0.015173673	331	951	44	0.046267087	0.132930514	GO:0022603	GO:BP	regulation of anatomical structure morphogenesis	FALSE
0.015857519	60	951	13	0.013669821	0.216666667	GO:0035107	GO:BP	appendage morphogenesis	FALSE
0.015857519	60	951	13	0.013669821	0.216666667	GO:0035108	GO:BP	limb morphogenesis	FALSE
0.016204348	53	951	12	0.012618297	0.226415094	GO:0010631	GO:BP	epithelial cell migration	FALSE
0.016204348	53	951	12	0.012618297	0.226415094	GO:0090130	GO:BP	tissue migration	FALSE
0.016204348	53	951	12	0.012618297	0.226415094	GO:0090132	GO:BP	epithelium migration	FALSE
0.016798911	21	951	7	0.007360673	0.333333333	GO:0060419	GO:BP	heart growth	FALSE
0.017198692	68	951	14	0.014721346	0.205882353	GO:0021953	GO:BP	central nervous system neuron differentiation	FALSE
0.018159742	11	951	5	0.005257624	0.454545455	GO:0031048	GO:BP	regulatory ncRNA-mediated heterochromatin formation	FALSE
0.018159742	11	951	5	0.005257624	0.454545455	GO:0034367	GO:BP	protein-containing complex remodeling	TRUE
0.018159742	11	951	5	0.005257624	0.454545455	GO:0060219	GO:BP	camera-type eye photoreceptor cell differentiation	FALSE
0.018159742	11	951	5	0.005257624	0.454545455	GO:0019433	GO:BP	triglyceride catabolic process	TRUE
0.018574401	84	951	16	0.016824395	0.19047619	GO:0007611	GO:BP	learning or memory	FALSE
0.018902316	16	951	6	0.006309148	0.375	GO:0002455	GO:BP	humoral immune response mediated by circulating immunoglobulin	FALSE
0.020086007	134	951	22	0.023133544	0.164179104	GO:0010975	GO:BP	regulation of neuron projection development	FALSE
0.020721849	7	951	4	0.004206099	0.571428571	GO:0007588	GO:BP	excretion	FALSE
0.020802819	101	951	18	0.018927445	0.178217822	GO:0050890	GO:BP	cognition	FALSE
0.020954979	93	951	17	0.01787592	0.182795699	GO:0099111	GO:BP	microtubule-based transport	FALSE
0.021797853	118	951	20	0.021030494	0.169491525	GO:0003002	GO:BP	regionalization	FALSE
0.021908132	55	951	12	0.012618297	0.218181818	GO:0098739	GO:BP	import across plasma membrane	FALSE
0.022421693	48	951	11	0.011566772	0.229166667	GO:0060840	GO:BP	artery development	FALSE
0.023429007	86	951	16	0.016824395	0.186046512	GO:0048762	GO:BP	mesenchymal cell differentiation	FALSE
0.023997387	63	951	13	0.013669821	0.206349206	GO:0009612	GO:BP	response to mechanical stimulus	FALSE

0.024168777	499	951	60	0.063091483	0.120240481	GO:0051094	GO:BP	positive regulation of developmental process	FALSE
0.024551279	254	951	35	0.036803365	0.137795276	GO:0070848	GO:BP	response to growth factor	FALSE
0.025245703	71	951	14	0.014721346	0.197183099	GO:0090257	GO:BP	regulation of muscle system process	FALSE
0.025301827	56	951	12	0.012618297	0.214285714	GO:0001764	GO:BP	neuron migration	FALSE
0.025982584	17	951	6	0.006309148	0.352941176	GO:0071711	GO:BP	basement membrane organization	FALSE
0.025982584	17	951	6	0.006309148	0.352941176	GO:0042461	GO:BP	photoreceptor cell development	FALSE
0.026092484	49	951	11	0.011566772	0.224489796	GO:0060972	GO:BP	left/right pattern formation	FALSE
0.026510414	471	951	57	0.059936909	0.121019108	GO:0009790	GO:BP	embryo development	FALSE
0.02713751	12	951	5	0.005257624	0.416666667	GO:0021772	GO:BP	olfactory bulb development	FALSE
0.02713751	12	951	5	0.005257624	0.416666667	GO:0021884	GO:BP	forebrain neuron development	FALSE
0.02713751	29	951	8	0.008412198	0.275862069	GO:0008608	GO:BP	attachment of spindle microtubules to kinetochore	TRUE
0.02713751	12	951	5	0.005257624	0.416666667	GO:0042462	GO:BP	eye photoreceptor cell development	FALSE
0.027948968	23	951	7	0.007360673	0.304347826	GO:0003407	GO:BP	neural retina development	FALSE
0.027948968	23	951	7	0.007360673	0.304347826	GO:0043279	GO:BP	response to alkaloid	TRUE
0.028672527	57	951	12	0.012618297	0.210526316	GO:0050680	GO:BP	negative regulation of epithelial cell proliferation	TRUE
0.028672527	57	951	12	0.012618297	0.210526316	GO:0002040	GO:BP	sprouting angiogenesis	FALSE
0.028772984	257	951	35	0.036803365	0.13618677	GO:0048285	GO:BP	organelle fission	FALSE
0.029554912	50	951	11	0.011566772	0.22	GO:0099175	GO:BP	regulation of postsynapse organization	FALSE
0.029554912	43	951	10	0.010515247	0.23255814	GO:1902414	GO:BP	protein localization to cell junction	TRUE
0.029554912	4	951	3	0.003154574	0.75	GO:0034374	GO:BP	low-density lipoprotein particle remodeling	TRUE
0.029554912	4	951	3	0.003154574	0.75	GO:1904778	GO:BP	positive regulation of protein localization to cell cortex	TRUE
0.029554912	4	951	3	0.003154574	0.75	GO:0045636	GO:BP	positive regulation of melanocyte differentiation	FALSE
0.029554912	4	951	3	0.003154574	0.75	GO:0032466	GO:BP	negative regulation of cytokinesis	TRUE
0.029554912	4	951	3	0.003154574	0.75	GO:1904776	GO:BP	regulation of protein localization to cell cortex	FALSE
0.029554912	4	951	3	0.003154574	0.75	GO:0045634	GO:BP	regulation of melanocyte differentiation	FALSE
0.029554912	4	951	3	0.003154574	0.75	GO:0021800	GO:BP	cerebral cortex tangential migration	TRUE
0.029554912	4	951	3	0.003154574	0.75	GO:0048496	GO:BP	maintenance of animal organ identity	FALSE
0.029554912	4	951	3	0.003154574	0.75	GO:0050966	GO:BP	detection of mechanical stimulus involved in sensory perception of pain	FALSE
0.029554912	4	951	3	0.003154574	0.75	GO:0070444	GO:BP	oligodendrocyte progenitor proliferation	FALSE
0.029554912	4	951	3	0.003154574	0.75	GO:0086012	GO:BP	membrane depolarization during cardiac muscle cell action potential	FALSE
0.029554912	4	951	3	0.003154574	0.75	GO:0050955	GO:BP	thermoception	FALSE
0.029554912	36	951	9	0.009463722	0.25	GO:0006942	GO:BP	regulation of striated muscle contraction	FALSE

0.029554912	4	951	3	0.003154574	0.75	GO:0014719	GO:BP	skeletal muscle satellite cell activation	TRUE
0.029554912	50	951	11	0.011566772	0.22	GO:0003279	GO:BP	cardiac septum development	FALSE
0.029977498	97	951	17	0.01787592	0.175257732	GO:0060485	GO:BP	mesenchyme development	FALSE
0.03312143	18	951	6	0.006309148	0.333333333	GO:0009880	GO:BP	embryonic pattern specification	FALSE
0.03312143	18	951	6	0.006309148	0.333333333	GO:0007528	GO:BP	neuromuscular junction development	FALSE
0.03312143	18	951	6	0.006309148	0.333333333	GO:0035315	GO:BP	hair cell differentiation	FALSE
0.033334438	66	951	13	0.013669821	0.196969697	GO:0003205	GO:BP	cardiac chamber development	FALSE
0.033346033	8	951	4	0.004206099	0.5	GO:0034505	GO:BP	tooth mineralization	TRUE
0.033346033	8	951	4	0.004206099	0.5	GO:0010996	GO:BP	response to auditory stimulus	FALSE
0.033346033	8	951	4	0.004206099	0.5	GO:0070286	GO:BP	axonemal dynein complex assembly	FALSE
0.033346033	8	951	4	0.004206099	0.5	GO:0035672	GO:BP	oligopeptide transmembrane transport	FALSE
0.033346033	8	951	4	0.004206099	0.5	GO:0097106	GO:BP	postsynaptic density organization	FALSE
0.033346033	51	951	11	0.011566772	0.215686275	GO:0035051	GO:BP	cardiocyte differentiation	FALSE
0.033380329	24	951	7	0.007360673	0.291666667	GO:0030199	GO:BP	collagen fibril organization	FALSE
0.033380329	338	951	43	0.045215563	0.127218935	GO:0046903	GO:BP	secretion	FALSE
0.033380329	24	951	7	0.007360673	0.291666667	GO:0021879	GO:BP	forebrain neuron differentiation	FALSE
0.037014566	13	951	5	0.005257624	0.384615385	GO:0021988	GO:BP	olfactory lobe development	FALSE
0.037625519	234	951	32	0.033648791	0.136752137	GO:0007186	GO:BP	G protein-coupled receptor signaling pathway	FALSE
0.038146864	52	951	11	0.011566772	0.211538462	GO:0048167	GO:BP	regulation of synaptic plasticity	FALSE
0.039860845	170	951	25	0.026288118	0.147058824	GO:0098813	GO:BP	nuclear chromosome segregation	FALSE
0.040347923	38	951	9	0.009463722	0.236842105	GO:1905515	GO:BP	non-motile cilium assembly	FALSE
0.040706908	60	951	12	0.012618297	0.2	GO:0009566	GO:BP	fertilization	FALSE
0.042235474	25	951	7	0.007360673	0.28	GO:0046620	GO:BP	regulation of organ growth	FALSE
0.042235474	25	951	7	0.007360673	0.28	GO:0097120	GO:BP	receptor localization to synapse	TRUE
0.042755528	19	951	6	0.006309148	0.315789474	GO:0055017	GO:BP	cardiac muscle tissue growth	FALSE
0.042755528	19	951	6	0.006309148	0.315789474	GO:0042908	GO:BP	xenobiotic transport	FALSE
0.043697562	101	951	17	0.01787592	0.168316832	GO:0021537	GO:BP	telencephalon development	FALSE
0.045605634	209	951	29	0.030494217	0.138755981	GO:0098771	GO:BP	inorganic ion homeostasis	FALSE
0.045605634	209	951	29	0.030494217	0.138755981	GO:0009611	GO:BP	response to wounding	TRUE
0.046024897	61	951	12	0.012618297	0.196721311	GO:2000241	GO:BP	regulation of reproductive process	FALSE
0.047148586	77	951	14	0.014721346	0.181818182	GO:0045216	GO:BP	cell-cell junction organization	FALSE
0.048474205	128	951	20	0.021030494	0.15625	GO:0055074	GO:BP	calcium ion homeostasis	FALSE
4.26E-47	1925	951	337	0.354363828	0.175064935	GO:0071944	GO:CC	cell periphery	TRUE
4.78E-31	1765	951	284	0.298633018	0.160906516	GO:0005886	GO:CC	plasma membrane	FALSE
1.37E-29	807	951	168	0.176656151	0.208178439	GO:0042995	GO:CC	cell projection	FALSE
5.72E-28	771	951	160	0.168243954	0.207522698	GO:0120025	GO:CC	plasma membrane bounded cell projection	FALSE
1.03E-24	119	951	53	0.05573081	0.445378151	GO:0031012	GO:CC	extracellular matrix	FALSE
1.41E-24	120	951	53	0.05573081	0.441666667	GO:0030312	GO:CC	external encapsulating structure	FALSE
1.11E-18	819	951	146	0.153522608	0.178266178	GO:0030054	GO:CC	cell junction	FALSE
1.11E-18	399	951	92	0.096740273	0.230576441	GO:0098590	GO:CC	plasma membrane region	FALSE
1.94E-18	476	951	102	0.107255521	0.214285714	GO:0005576	GO:CC	extracellular region	TRUE

1.52E-15	300	951	72	0.075709779	0.24	GO:0005929	GO:CC	cilium	FALSE
3.09E-15	3584	951	410	0.431125131	0.114397321	GO:0016020	GO:CC	membrane	FALSE
3.17E-14	346	951	76	0.079915878	0.219653179	GO:0043005	GO:CC	neuron projection	FALSE
7.64E-14	97	951	36	0.03785489	0.371134021	GO:0031514	GO:CC	motile cilium	TRUE
8.24E-14	40	951	23	0.024185068	0.575	GO:0005604	GO:CC	basement membrane	FALSE
9.95E-14	56	951	27	0.028391167	0.482142857	GO:0062023	GO:CC	collagen-containing extracellular matrix	FALSE
7.15E-13	155	951	45	0.047318612	0.290322581	GO:0045177	GO:CC	apical part of cell	FALSE
1.13E-11	61	951	26	0.027339642	0.426229508	GO:0034703	GO:CC	cation channel complex	FALSE
3.41E-11	59	951	25	0.026288118	0.423728814	GO:0005930	GO:CC	axoneme	FALSE
3.41E-11	59	951	25	0.026288118	0.423728814	GO:0097014	GO:CC	ciliary plasm	FALSE
3.61E-11	84	951	30	0.031545741	0.357142857	GO:0097060	GO:CC	synaptic membrane	FALSE
3.61E-11	123	951	37	0.038906414	0.300813008	GO:0016324	GO:CC	apical plasma membrane	FALSE
7.39E-11	325	951	66	0.069400631	0.203076923	GO:0005615	GO:CC	extracellular space	FALSE
7.39E-11	1139	951	159	0.167192429	0.139596137	GO:0005856	GO:CC	cytoskeleton	FALSE
1.10E-10	67	951	26	0.027339642	0.388059701	GO:0097729	GO:CC	9+2 motile cilium	FALSE
2.65E-10	85	951	29	0.030494217	0.341176471	GO:0034702	GO:CC	monoatomic ion channel complex	FALSE
9.29E-10	21	951	14	0.014721346	0.666666667	GO:0005581	GO:CC	collagen trimer	FALSE
1.43E-09	102	951	31	0.032597266	0.303921569	GO:0099568	GO:CC	cytoplasmic region	FALSE
2.09E-09	201	951	46	0.048370137	0.228855721	GO:0098797	GO:CC	plasma membrane protein complex	FALSE
2.94E-09	11	951	10	0.010515247	0.909090909	GO:0034706	GO:CC	sodium channel complex	FALSE
3.49E-09	539	951	88	0.092534175	0.163265306	GO:0045202	GO:CC	synapse	FALSE
8.21E-09	284	951	56	0.058885384	0.197183099	GO:0070161	GO:CC	anchoring junction	FALSE
3.98E-08	8	951	8	0.008412198	1	GO:0001518	GO:CC	voltage-gated sodium channel complex	FALSE
6.45E-08	35	951	16	0.016824395	0.457142857	GO:0042734	GO:CC	presynaptic membrane	FALSE
7.32E-08	180	951	40	0.042060988	0.222222222	GO:0098793	GO:CC	presynapse	FALSE
9.95E-08	89	951	26	0.027339642	0.292134831	GO:0032838	GO:CC	plasma membrane bounded cell projection cytoplasm	FALSE
1.55E-07	62	951	21	0.022082019	0.338709677	GO:0036126	GO:CC	sperm flagellum	FALSE
1.73E-07	193	951	41	0.043112513	0.212435233	GO:0005911	GO:CC	cell-cell junction	FALSE
2.27E-07	29	951	14	0.014721346	0.482758621	GO:0016459	GO:CC	myosin complex	TRUE
6.73E-07	363	951	61	0.064143007	0.168044077	GO:0099081	GO:CC	supramolecular polymer	FALSE
8.37E-07	46	951	17	0.01787592	0.369565217	GO:0042383	GO:CC	sarcolemma	FALSE
9.10E-07	146	951	33	0.034700315	0.226027397	GO:1990351	GO:CC	transporter complex	FALSE
1.08E-06	572	951	84	0.088328076	0.146853147	GO:0099080	GO:CC	supramolecular complex	FALSE
2.13E-06	130	951	30	0.031545741	0.230769231	GO:1902495	GO:CC	transmembrane transporter complex	FALSE
2.38E-06	25	951	12	0.012618297	0.48	GO:0098839	GO:CC	postsynaptic density membrane	FALSE
3.15E-06	30	951	13	0.013669821	0.433333333	GO:0099634	GO:CC	postsynaptic specialization membrane	FALSE
3.48E-06	56	951	18	0.018927445	0.321428571	GO:0045211	GO:CC	postsynaptic membrane	FALSE
3.57E-06	11	951	8	0.008412198	0.727272727	GO:0098644	GO:CC	complex of collagen trimers	FALSE
4.05E-06	265	951	47	0.049421661	0.177358491	GO:0009986	GO:CC	cell surface	TRUE
6.11E-06	27	951	12	0.012618297	0.444444444	GO:0030286	GO:CC	dynein complex	FALSE
9.37E-06	358	951	57	0.059936909	0.159217877	GO:0099512	GO:CC	supramolecular fiber	FALSE
1.22E-05	99	951	24	0.025236593	0.242424242	GO:0099572	GO:CC	postsynaptic specialization	FALSE
1.38E-05	93	951	23	0.024185068	0.247311828	GO:0014069	GO:CC	postsynaptic density	FALSE
1.43E-05	236	951	42	0.044164038	0.177966102	GO:0015629	GO:CC	actin cytoskeleton	FALSE
1.43E-05	39	951	14	0.014721346	0.358974359	GO:0035869	GO:CC	ciliary transition zone	FALSE
2.48E-05	139	951	29	0.030494217	0.208633094	GO:0043235	GO:CC	receptor complex	TRUE
2.59E-05	64	951	18	0.018927445	0.28125	GO:0098862	GO:CC	cluster of actin-based cell projections	FALSE
2.77E-05	97	951	23	0.024185068	0.237113402	GO:0032279	GO:CC	asymmetric synapse	FALSE
3.29E-05	164	951	32	0.033648791	0.195121951	GO:0030424	GO:CC	axon	FALSE

5.51E-05	108	951	24	0.025236593	0.222222222	GO:0098984	GO:CC	neuron to neuron synapse	FALSE
8.00E-05	39	951	13	0.013669821	0.333333333	GO:0097733	GO:CC	photoreceptor cell cilium	FALSE
0.000117973	52	951	15	0.015772871	0.288461538	GO:0097730	GO:CC	non-motile cilium	FALSE
0.000121984	20	951	9	0.009463722	0.45	GO:0014704	GO:CC	intercalated disc	FALSE
0.000143078	16	951	8	0.008412198	0.5	GO:0048786	GO:CC	presynaptic active zone	FALSE
0.000155146	185	951	33	0.034700315	0.178378378	GO:0036477	GO:CC	somatodendritic compartment	FALSE
0.000188881	21	951	9	0.009463722	0.428571429	GO:0032391	GO:CC	photoreceptor connecting cilium	TRUE
0.000238291	214	951	36	0.03785489	0.168224299	GO:0098794	GO:CC	postsynapse	FALSE
0.000311482	44	951	13	0.013669821	0.295454545	GO:0097731	GO:CC	9+0 non-motile cilium	FALSE
0.000420399	4	951	4	0.004206099	1	GO:0005587	GO:CC	collagen type IV trimer	FALSE
0.000420399	4	951	4	0.004206099	1	GO:0005579	GO:CC	membrane attack complex	FALSE
0.000432225	146	951	27	0.028391167	0.184931507	GO:0005938	GO:CC	cell cortex	FALSE
0.000446245	14	951	7	0.007360673	0.5	GO:0005891	GO:CC	voltage-gated calcium channel complex	FALSE
0.000446245	14	951	7	0.007360673	0.5	GO:0030315	GO:CC	T-tubule	FALSE
0.00073633	119	951	23	0.024185068	0.193277311	GO:0045178	GO:CC	basal part of cell	FALSE
0.000736775	30	951	10	0.010515247	0.333333333	GO:0016528	GO:CC	sarcoplasm	TRUE
0.000736775	15	951	7	0.007360673	0.466666667	GO:0044304	GO:CC	main axon	FALSE
0.000736775	201	951	33	0.034700315	0.164179104	GO:0098978	GO:CC	glutamatergic synapse	FALSE
0.000821099	25	951	9	0.009463722	0.36	GO:0034704	GO:CC	calcium channel complex	FALSE
0.000903186	98	951	20	0.021030494	0.204081633	GO:0016323	GO:CC	basolateral plasma membrane	FALSE
0.001114751	92	951	19	0.01997897	0.206521739	GO:0098858	GO:CC	actin-based cell projection	FALSE
0.001121593	26	951	9	0.009463722	0.346153846	GO:0098982	GO:CC	GABA-ergic synapse	FALSE
0.001254362	32	951	10	0.010515247	0.3125	GO:0044291	GO:CC	cell-cell contact zone	FALSE
0.001380357	109	951	21	0.022082019	0.19266055	GO:0009925	GO:CC	basal plasma membrane	FALSE
0.001391404	12	951	6	0.006309148	0.5	GO:0097228	GO:CC	sperm principal piece	FALSE
0.00154069	5	951	4	0.004206099	0.8	GO:0005614	GO:CC	interstitial matrix	FALSE
0.00154069	5	951	4	0.004206099	0.8	GO:0098645	GO:CC	collagen network	FALSE
0.00154069	5	951	4	0.004206099	0.8	GO:0046581	GO:CC	intercellular canaliculus	FALSE
0.00154069	5	951	4	0.004206099	0.8	GO:0098688	GO:CC	parallel fiber to Purkinje cell synapse	FALSE
0.00154069	5	951	4	0.004206099	0.8	GO:0098642	GO:CC	network-forming collagen trimer	FALSE
0.001624954	17	951	7	0.007360673	0.411764706	GO:0099086	GO:CC	synaptonemal structure	FALSE
0.001624954	17	951	7	0.007360673	0.411764706	GO:0000795	GO:CC	synaptonemal complex	TRUE
0.001896251	89	951	18	0.018927445	0.202247191	GO:0043025	GO:CC	neuronal cell body	FALSE
0.001990871	137	951	24	0.025236593	0.175182482	GO:0030425	GO:CC	dendrite	FALSE
0.001990871	137	951	24	0.025236593	0.175182482	GO:0097447	GO:CC	dendritic tree	FALSE
0.002175051	23	951	8	0.008412198	0.347826087	GO:0016529	GO:CC	sarcoplasmic reticulum	FALSE
0.00236951	9	951	5	0.005257624	0.555555556	GO:0002177	GO:CC	manchette	FALSE
0.002611819	55	951	13	0.013669821	0.236363636	GO:0005875	GO:CC	microtubule associated complex	FALSE
0.002801747	42	951	11	0.011566772	0.261904762	GO:0005903	GO:CC	brush border	FALSE
0.003520362	3	951	3	0.003154574	1	GO:0043194	GO:CC	axon initial segment	FALSE
0.003520362	3	951	3	0.003154574	1	GO:0042627	GO:CC	chylomicron	FALSE
0.003520362	3	951	3	0.003154574	1	GO:1990454	GO:CC	L-type voltage-gated calcium channel complex	FALSE
0.003520362	3	951	3	0.003154574	1	GO:0099569	GO:CC	presynaptic cytoskeleton	FALSE
0.003592058	6	951	4	0.004206099	0.666666667	GO:0005583	GO:CC	fibrillar collagen trimer	FALSE
0.003592058	6	951	4	0.004206099	0.666666667	GO:0098643	GO:CC	banded collagen fibril	FALSE
0.003592058	6	951	4	0.004206099	0.666666667	GO:0005858	GO:CC	axonemal dynein complex	FALSE
0.003592058	6	951	4	0.004206099	0.666666667	GO:0048787	GO:CC	presynaptic active zone membrane	FALSE
0.003592058	755	951	87	0.09148265	0.115231788	GO:0015630	GO:CC	microtubule cytoskeleton	FALSE
0.003909481	65	951	14	0.014721346	0.215384615	GO:0030017	GO:CC	sarcomere	FALSE

0.00579678	46	951	11	0.011566772	0.239130435	GO:0043296	GO:CC	apical junction complex	FALSE
0.006355196	11	951	5	0.005257624	0.454545455	GO:0045495	GO:CC	pole plasm	FALSE
0.006355196	76	951	15	0.015772871	0.197368421	GO:0043292	GO:CC	contractile muscle fiber	FALSE
0.006355196	11	951	5	0.005257624	0.454545455	GO:0060076	GO:CC	excitatory synapse	FALSE
0.006355196	11	951	5	0.005257624	0.454545455	GO:0060293	GO:CC	germ plasm	FALSE
0.006355196	11	951	5	0.005257624	0.454545455	GO:0043186	GO:CC	P granule	TRUE
0.00727921	7	951	4	0.004206099	0.571428571	GO:0033391	GO:CC	chromatoid body	TRUE
0.007661769	118	951	20	0.021030494	0.169491525	GO:0031253	GO:CC	cell projection membrane	FALSE
0.0094314	42	951	10	0.010515247	0.238095238	GO:0070160	GO:CC	tight junction	FALSE
0.009733914	12	951	5	0.005257624	0.416666667	GO:0016342	GO:CC	catenin complex	FALSE
0.009733914	12	951	5	0.005257624	0.416666667	GO:0000939	GO:CC	inner kinetochore	TRUE
0.010022679	23	951	7	0.007360673	0.304347826	GO:0097225	GO:CC	sperm midpiece	FALSE
0.010536369	113	951	19	0.01997897	0.168141593	GO:0044297	GO:CC	cell body	FALSE
0.010855314	4	951	3	0.003154574	0.75	GO:0043256	GO:CC	laminin complex	FALSE
0.010855314	4	951	3	0.003154574	0.75	GO:0031262	GO:CC	Ndc80 complex	TRUE
0.010855314	4	951	3	0.003154574	0.75	GO:0071546	GO:CC	pi-body	FALSE
0.012200873	18	951	6	0.006309148	0.333333333	GO:0031526	GO:CC	brush border membrane	FALSE
0.012365454	24	951	7	0.007360673	0.291666667	GO:0001750	GO:CC	photoreceptor outer segment	FALSE
0.012365454	74	951	14	0.014721346	0.189189189	GO:0030016	GO:CC	myofibril	FALSE
0.012365454	24	951	7	0.007360673	0.291666667	GO:0044306	GO:CC	neuron projection terminus	FALSE
0.012413815	44	951	10	0.010515247	0.227272727	GO:0019897	GO:CC	extrinsic component of plasma membrane	FALSE
0.013219843	13	951	5	0.005257624	0.384615385	GO:0099738	GO:CC	cell cortex region	FALSE
0.013219843	13	951	5	0.005257624	0.384615385	GO:0030057	GO:CC	desmosome	FALSE
0.013219843	160	951	24	0.025236593	0.15	GO:0000793	GO:CC	condensed chromosome	FALSE
0.013219843	13	951	5	0.005257624	0.384615385	GO:0032589	GO:CC	neuron projection membrane	FALSE
0.014257073	45	951	10	0.010515247	0.222222222	GO:0031674	GO:CC	I band	FALSE
0.014484348	60	951	12	0.012618297	0.2	GO:0005912	GO:CC	adherens junction	FALSE
0.014995226	25	951	7	0.007360673	0.28	GO:0032421	GO:CC	stereocilium bundle	FALSE
0.017035746	39	951	9	0.009463722	0.230769231	GO:0005923	GO:CC	bicellular tight junction	FALSE
0.018407655	14	951	5	0.005257624	0.357142857	GO:0031941	GO:CC	filamentous actin	TRUE
0.018407655	86	951	15	0.015772871	0.174418605	GO:0036064	GO:CC	ciliary basal body	FALSE
0.018807191	9	951	4	0.004206099	0.444444444	GO:0031672	GO:CC	A band	FALSE
0.018942787	47	951	10	0.010515247	0.212765957	GO:0030175	GO:CC	filopodium	FALSE
0.020414959	104	951	17	0.01787592	0.163461538	GO:0030055	GO:CC	cell-substrate junction	FALSE
0.021324069	5	951	3	0.003154574	0.6	GO:0016327	GO:CC	apicolateral plasma membrane	FALSE
0.021324069	140	951	21	0.022082019	0.15	GO:0000775	GO:CC	chromosome, centromeric region	TRUE
0.021324069	5	951	3	0.003154574	0.6	GO:0036128	GO:CC	CatSper complex	FALSE
0.021324069	5	951	3	0.003154574	0.6	GO:0043083	GO:CC	synaptic cleft	FALSE
0.021324069	5	951	3	0.003154574	0.6	GO:0098831	GO:CC	presynaptic active zone cytoplasmic component	FALSE
0.021324069	5	951	3	0.003154574	0.6	GO:0160111	GO:CC	axonemal A tubule inner sheath	TRUE
0.027362817	10	951	4	0.004206099	0.4	GO:0033017	GO:CC	sarcoplasmic reticulum membrane	FALSE
0.027362817	10	951	4	0.004206099	0.4	GO:0000800	GO:CC	lateral element	FALSE
0.028090166	2	951	2	0.002103049	1	GO:0002139	GO:CC	stereocilia coupling link	FALSE
0.028090166	2	951	2	0.002103049	1	GO:0036156	GO:CC	inner dynein arm	FALSE
0.028090166	2	951	2	0.002103049	1	GO:0005606	GO:CC	laminin-1 complex	FALSE
0.028090166	2	951	2	0.002103049	1	GO:0000802	GO:CC	transverse filament	FALSE
0.028090166	2	951	2	0.002103049	1	GO:0097209	GO:CC	epidermal lamellar body	TRUE
0.028090166	2	951	2	0.002103049	1	GO:0048788	GO:CC	cytoskeleton of presynaptic active zone	FALSE
0.028536849	278	951	35	0.036803365	0.125899281	GO:0099513	GO:CC	polymeric cytoskeletal fiber	FALSE

0.02925407	43	951	9	0.009463722	0.209302326	GO:0000794	GO:CC	condensed nuclear chromosome	FALSE
0.035166272	52	951	10	0.010515247	0.192307692	GO:0030863	GO:CC	cortical cytoskeleton	FALSE
0.035166272	52	951	10	0.010515247	0.192307692	GO:0016328	GO:CC	lateral plasma membrane	FALSE
0.035179253	529	951	59	0.062039958	0.111531191	GO:0098796	GO:CC	membrane protein complex	FALSE
0.036563683	11	951	4	0.004206099	0.363636364	GO:0005879	GO:CC	axonemal microtubule	FALSE
0.036563683	11	951	4	0.004206099	0.363636364	GO:1990777	GO:CC	lipoprotein particle	FALSE
0.036563683	6	951	3	0.003154574	0.5	GO:0030893	GO:CC	meiotic cohesin complex	TRUE
0.036563683	11	951	4	0.004206099	0.363636364	GO:0034358	GO:CC	plasma lipoprotein particle	FALSE
8.96E-15	308	951	74	0.077812829	0.24025974	GO:0015075	GO:MF	monoatomic ion transmembrane transporter activity	TRUE
4.94E-14	165	951	50	0.052576236	0.303030303	GO:0005216	GO:MF	monoatomic ion channel activity	FALSE
2.00E-13	528	951	100	0.105152471	0.189393939	GO:0005215	GO:MF	transporter activity	FALSE
2.29E-13	476	951	93	0.097791798	0.195378151	GO:0022857	GO:MF	transmembrane transporter activity	FALSE
3.27E-13	294	951	68	0.07150368	0.231292517	GO:0015318	GO:MF	inorganic molecular entity transmembrane transporter activity	FALSE
5.80E-13	191	951	52	0.054679285	0.272251309	GO:0046873	GO:MF	metal ion transmembrane transporter activity	FALSE
6.24E-13	180	951	50	0.052576236	0.277777778	GO:0022803	GO:MF	passive transmembrane transporter activity	FALSE
6.24E-13	180	951	50	0.052576236	0.277777778	GO:0015267	GO:MF	channel activity	FALSE
4.43E-12	128	951	40	0.042060988	0.3125	GO:0050839	GO:MF	cell adhesion molecule binding	TRUE
1.01E-11	63	951	27	0.028391167	0.428571429	GO:0003774	GO:MF	cytoskeletal motor activity	TRUE
4.22E-11	125	951	38	0.039957939	0.304	GO:0005261	GO:MF	monoatomic cation channel activity	FALSE
5.48E-11	257	951	58	0.060988433	0.225680934	GO:0008324	GO:MF	monoatomic cation transmembrane transporter activity	FALSE
6.59E-11	63	951	26	0.027339642	0.412698413	GO:0015081	GO:MF	sodium ion transmembrane transporter activity	FALSE
1.39E-10	249	951	56	0.058885384	0.224899598	GO:0022890	GO:MF	inorganic cation transmembrane transporter activity	FALSE
2.88E-09	21	951	14	0.014721346	0.666666667	GO:0005201	GO:MF	extracellular matrix structural constituent	TRUE
3.40E-09	262	951	55	0.057833859	0.209923664	GO:0005509	GO:MF	calcium ion binding	TRUE
7.70E-09	76	951	26	0.027339642	0.342105263	GO:0022836	GO:MF	gated channel activity	FALSE
4.47E-08	12	951	10	0.010515247	0.833333333	GO:0030020	GO:MF	extracellular matrix structural constituent conferring tensile strength	FALSE
4.47E-08	12	951	10	0.010515247	0.833333333	GO:0008569	GO:MF	minus-end-directed microtubule motor activity	FALSE
1.51E-07	481	951	78	0.082018927	0.162162162	GO:0008092	GO:MF	cytoskeletal protein binding	TRUE
4.17E-07	11	951	9	0.009463722	0.818181818	GO:0005248	GO:MF	voltage-gated sodium channel activity	FALSE
4.82E-07	57	951	20	0.021030494	0.350877193	GO:0120544	GO:MF	polypeptide conformation or assembly isomerase activity	FALSE
4.82E-07	42	951	17	0.01787592	0.404761905	GO:0022834	GO:MF	ligand-gated channel activity	FALSE
4.82E-07	42	951	17	0.01787592	0.404761905	GO:0015276	GO:MF	ligand-gated monoatomic ion channel activity	FALSE

4.82E-07	186	951	40	0.042060988	0.215053763	GO:0022804	GO:MF	active transmembrane transporter activity	FALSE
1.01E-06	101	951	27	0.028391167	0.267326733	GO:0008237	GO:MF	metallopeptidase activity	TRUE
1.57E-06	61	951	20	0.021030494	0.327868852	GO:0005262	GO:MF	calcium channel activity	FALSE
1.79E-06	2844	951	303	0.318611987	0.106540084	GO:0043167	GO:MF	ion binding	FALSE
1.93E-06	62	951	20	0.021030494	0.322580645	GO:0008509	GO:MF	monoatomic anion transmembrane transporter activity	FALSE
1.93E-06	23	951	12	0.012618297	0.52173913	GO:0005272	GO:MF	sodium channel activity	FALSE
4.27E-06	38	951	15	0.015772871	0.394736842	GO:0003777	GO:MF	microtubule motor activity	FALSE
5.81E-06	66	951	20	0.021030494	0.303030303	GO:0005178	GO:MF	integrin binding	FALSE
6.20E-06	1657	951	191	0.20084122	0.115268558	GO:0016787	GO:MF	hydrolase activity	FALSE
7.24E-06	73	951	21	0.022082019	0.287671233	GO:0015085	GO:MF	calcium ion transmembrane transporter activity	FALSE
7.25E-06	14	951	9	0.009463722	0.642857143	GO:0015106	GO:MF	bicarbonate transmembrane transporter activity	FALSE
7.98E-06	2938	951	307	0.322818086	0.104492852	GO:0036094	GO:MF	small molecule binding	FALSE
8.88E-06	937	951	120	0.126182965	0.128068303	GO:0030554	GO:MF	adenyl nucleotide binding	FALSE
9.38E-06	18	951	10	0.010515247	0.555555556	GO:0045505	GO:MF	dynein intermediate chain binding	TRUE
1.35E-05	876	951	113	0.118822292	0.128995434	GO:0005524	GO:MF	ATP binding	FALSE
1.49E-05	888	951	114	0.119873817	0.128378378	GO:0032559	GO:MF	adenyl ribonucleotide binding	FALSE
1.70E-05	19	951	10	0.010515247	0.526315789	GO:0051959	GO:MF	dynein light intermediate chain binding	FALSE
2.33E-05	112	951	26	0.027339642	0.232142857	GO:0015291	GO:MF	secondary active transmembrane transporter activity	FALSE
3.98E-05	45	951	15	0.015772871	0.333333333	GO:0015108	GO:MF	chloride transmembrane transporter activity	FALSE
4.81E-05	30	951	12	0.012618297	0.4	GO:0140359	GO:MF	ABC-type transporter activity	TRUE
9.84E-05	60	951	17	0.01787592	0.283333333	GO:0015103	GO:MF	inorganic anion transmembrane transporter activity	FALSE
9.99E-05	214	951	38	0.039957939	0.177570093	GO:0003779	GO:MF	actin binding	FALSE
0.000184398	179	951	33	0.034700315	0.184357542	GO:0016887	GO:MF	ATP hydrolysis activity	FALSE
0.000207071	104	951	23	0.024185068	0.221153846	GO:0008514	GO:MF	organic anion transmembrane transporter activity	FALSE
0.000220542	1337	951	152	0.159831756	0.11368736	GO:0097367	GO:MF	carbohydrate derivative binding	FALSE
0.000221547	352	951	53	0.05573081	0.150568182	GO:0008233	GO:MF	peptidase activity	FALSE
0.000244621	3253	951	324	0.340694006	0.099600369	GO:0005515	GO:MF	protein binding	FALSE
0.000295691	12	951	7	0.007360673	0.583333333	GO:0004181	GO:MF	metallocarboxypeptidase activity	FALSE
0.000297315	59	951	16	0.016824395	0.271186441	GO:0008238	GO:MF	exopeptidase activity	FALSE
0.000341404	395	951	57	0.059936909	0.144303797	GO:0060089	GO:MF	molecular transducer activity	FALSE
0.000341404	395	951	57	0.059936909	0.144303797	GO:0038023	GO:MF	signaling receptor activity	TRUE
0.000382739	42	951	13	0.013669821	0.30952381	GO:0008201	GO:MF	heparin binding	FALSE
0.000595209	22	951	9	0.009463722	0.409090909	GO:0015370	GO:MF	solute:sodium symporter activity	FALSE
0.000595209	22	951	9	0.009463722	0.409090909	GO:0004180	GO:MF	carboxypeptidase activity	FALSE
0.000608071	38	951	12	0.012618297	0.315789474	GO:0015294	GO:MF	solute:monoatomic cation symporter activity	FALSE
0.000726415	33	951	11	0.011566772	0.333333333	GO:0045296	GO:MF	cadherin binding	FALSE
0.000859176	1702	951	182	0.191377497	0.10693302	GO:0046872	GO:MF	metal ion binding	FALSE

0.000941021	65	951	16	0.016824395	0.246153846	GO:0005539	GO:MF	glycosaminoglycan binding	FALSE
0.000997135	46	951	13	0.013669821	0.282608696	GO:0015293	GO:MF	symporter activity	FALSE
0.001107152	29	951	10	0.010515247	0.344827586	GO:0019199	GO:MF	transmembrane receptor protein kinase activity	FALSE
0.001359382	1456	951	158	0.166140904	0.108516484	GO:0043168	GO:MF	anion binding	FALSE
0.001493257	30	951	10	0.010515247	0.333333333	GO:0099094	GO:MF	ligand-gated monoatomic cation channel activity	FALSE
0.001622531	1735	951	183	0.192429022	0.105475504	GO:0043169	GO:MF	cation binding	FALSE
0.001827189	403	951	55	0.057833859	0.136476427	GO:0140657	GO:MF	ATP-dependent activity	FALSE
0.001827189	1272	951	140	0.14721346	0.110062893	GO:0017076	GO:MF	purine nucleotide binding	FALSE
0.002138463	63	951	15	0.015772871	0.238095238	GO:0015297	GO:MF	antiporter activity	FALSE
0.002293741	172	951	29	0.030494217	0.168604651	GO:0120543	GO:MF	macromolecular conformation isomerase activity	FALSE
0.002446929	21	951	8	0.008412198	0.380952381	GO:0004714	GO:MF	transmembrane receptor protein tyrosine kinase activity	FALSE
0.002557459	1197	951	132	0.138801262	0.110275689	GO:0035639	GO:MF	purine ribonucleoside triphosphate binding	FALSE
0.002557459	234	951	36	0.03785489	0.153846154	GO:0004888	GO:MF	transmembrane signaling receptor activity	FALSE
0.002840075	1222	951	134	0.140904311	0.109656301	GO:0032555	GO:MF	purine ribonucleotide binding	FALSE
0.002943512	27	951	9	0.009463722	0.333333333	GO:0005254	GO:MF	chloride channel activity	FALSE
0.003144842	33	951	10	0.010515247	0.303030303	GO:1901682	GO:MF	sulfur compound transmembrane transporter activity	FALSE
0.003170244	52	951	13	0.013669821	0.25	GO:0008081	GO:MF	phosphoric diester hydrolase activity	TRUE
0.003467711	1229	951	134	0.140904311	0.109031733	GO:0032553	GO:MF	ribonucleotide binding	FALSE
0.0043797	468	951	60	0.063091483	0.128205128	GO:0005102	GO:MF	signaling receptor binding	FALSE
0.004414906	13	951	6	0.006309148	0.461538462	GO:0005245	GO:MF	voltage-gated calcium channel activity	FALSE
0.00442386	61	951	14	0.014721346	0.229508197	GO:0042626	GO:MF	ATPase-coupled transmembrane transporter activity	FALSE
0.00442386	61	951	14	0.014721346	0.229508197	GO:0046943	GO:MF	carboxylic acid transmembrane transporter activity	FALSE
0.00442386	61	951	14	0.014721346	0.229508197	GO:0005342	GO:MF	organic acid transmembrane transporter activity	FALSE
0.00442386	23	951	8	0.008412198	0.347826087	GO:0098631	GO:MF	cell adhesion mediator activity	FALSE
0.004688147	9	951	5	0.005257624	0.555555556	GO:0035673	GO:MF	oligopeptide transmembrane transporter activity	TRUE
0.005161935	55	951	13	0.013669821	0.236363636	GO:0004725	GO:MF	protein tyrosine phosphatase activity	TRUE
0.006030142	36	951	10	0.010515247	0.277777778	GO:0005253	GO:MF	monoatomic anion channel activity	FALSE
0.006546659	14	951	6	0.006309148	0.428571429	GO:0005217	GO:MF	intracellularly ligand-gated monoatomic ion channel activity	FALSE
0.006633688	3	951	3	0.003154574	1	GO:0086007	GO:MF	voltage-gated calcium channel activity involved in cardiac muscle cell action potential	FALSE

0.006633688	3	951	3	0.003154574	1	GO:0033284	GO:MF	ATPase-coupled carboxylic acid transmembrane transporter activity	FALSE
0.006633688	3	951	3	0.003154574	1	GO:0033283	GO:MF	ATPase-coupled organic acid transmembrane transporter activity	FALSE
0.006633688	3	951	3	0.003154574	1	GO:0015432	GO:MF	ABC-type bile acid transporter activity	FALSE
0.006633688	3	951	3	0.003154574	1	GO:0008046	GO:MF	axon guidance receptor activity	FALSE
0.006633688	3	951	3	0.003154574	1	GO:0005219	GO:MF	ryanodine-sensitive calcium-release channel activity	TRUE
0.006633688	3	951	3	0.003154574	1	GO:0033285	GO:MF	ATPase-coupled monocarboxylic acid transmembrane transporter activity	FALSE
0.006634404	50	951	12	0.012618297	0.24	GO:0004222	GO:MF	metalloendopeptidase activity	FALSE
0.006880255	37	951	10	0.010515247	0.27027027	GO:0005244	GO:MF	voltage-gated monoatomic ion channel activity	FALSE
0.006880255	6	951	4	0.004206099	0.666666667	GO:0140410	GO:MF	monoatomic cation:bicarbonate symporter activity	FALSE
0.007141416	31	951	9	0.009463722	0.290322581	GO:0005518	GO:MF	collagen binding	FALSE
0.007446489	10	951	5	0.005257624	0.5	GO:0099095	GO:MF	ligand-gated monoatomic anion channel activity	FALSE
0.00937858	74	951	15	0.015772871	0.202702703	GO:0005319	GO:MF	lipid transporter activity	FALSE
0.009567934	578	951	69	0.072555205	0.119377163	GO:0044877	GO:MF	protein-containing complex binding	FALSE
0.010014796	98	951	18	0.018927445	0.183673469	GO:0051015	GO:MF	actin filament binding	FALSE
0.010092454	39	951	10	0.010515247	0.256410256	GO:0022832	GO:MF	voltage-gated channel activity	FALSE
0.010445361	75	951	15	0.015772871	0.2	GO:0016298	GO:MF	lipase activity	TRUE
0.011543213	27	951	8	0.008412198	0.296296296	GO:0022843	GO:MF	voltage-gated monoatomic cation channel activity	FALSE
0.01190971	11	951	5	0.005257624	0.454545455	GO:0005044	GO:MF	scavenger receptor activity	TRUE
0.013153104	7	951	4	0.004206099	0.571428571	GO:0141147	GO:MF	intracellularly calcium-gated channel activity	FALSE
0.013153104	7	951	4	0.004206099	0.571428571	GO:0140900	GO:MF	chloride:bicarbonate antiporter activity	FALSE
0.013153104	7	951	4	0.004206099	0.571428571	GO:0140829	GO:MF	bicarbonate:monoatomic anion antiporter activity	FALSE
0.013153104	214	951	31	0.032597266	0.144859813	GO:0004175	GO:MF	endopeptidase activity	FALSE
0.013153104	7	951	4	0.004206099	0.571428571	GO:0005229	GO:MF	intracellularly calcium-gated chloride channel activity	FALSE
0.015017403	86	951	16	0.016824395	0.186046512	GO:0008236	GO:MF	serine-type peptidase activity	FALSE
0.01624211	1376	951	142	0.149316509	0.103197674	GO:0000166	GO:MF	nucleotide binding	FALSE
0.01671848	17	951	6	0.006309148	0.352941176	GO:0004114	GO:MF	3',5'-cyclic-nucleotide phosphodiesterase activity	FALSE
0.017217835	12	951	5	0.005257624	0.416666667	GO:0030507	GO:MF	spectrin binding	FALSE
0.017217835	1446	951	148	0.155625657	0.102351314	GO:1901363	GO:MF	heterocyclic compound binding	FALSE
0.017217835	12	951	5	0.005257624	0.416666667	GO:0004806	GO:MF	triacylglycerol lipase activity	FALSE
0.017217835	64	951	13	0.013669821	0.203125	GO:0052689	GO:MF	carboxylic ester hydrolase activity	FALSE
0.017910193	96	951	17	0.01787592	0.177083333	GO:0016829	GO:MF	lyase activity	TRUE

0.018015341	88	951	16	0.016824395	0.181818182	GO:0017171	GO:MF	serine hydrolase activity	FALSE
0.019131766	4	951	3	0.003154574	0.75	GO:0015125	GO:MF	bile acid transmembrane transporter activity	FALSE
0.019131766	4	951	3	0.003154574	0.75	GO:0048407	GO:MF	platelet-derived growth factor binding	FALSE
0.019131766	4	951	3	0.003154574	0.75	GO:0031402	GO:MF	sodium ion binding	TRUE
0.019536433	73	951	14	0.014721346	0.191780822	GO:0015399	GO:MF	primary active transmembrane transporter activity	FALSE
0.020671682	1388	951	142	0.149316509	0.102305476	GO:1901265	GO:MF	nucleoside phosphate binding	FALSE
0.021005858	18	951	6	0.006309148	0.333333333	GO:0004112	GO:MF	cyclic-nucleotide phosphodiesterase activity	FALSE
0.021005858	18	951	6	0.006309148	0.333333333	GO:0005452	GO:MF	solute:inorganic anion antiporter activity	FALSE
0.021388088	8	951	4	0.004206099	0.5	GO:0015116	GO:MF	sulfate transmembrane transporter activity	FALSE
0.027362997	76	951	14	0.014721346	0.184210526	GO:0004252	GO:MF	serine-type endopeptidase activity	FALSE
0.027362997	68	951	13	0.013669821	0.191176471	GO:0005516	GO:MF	calmodulin binding	FALSE
0.027362997	255	951	34	0.03575184	0.133333333	GO:0042578	GO:MF	phosphoric ester hydrolase activity	FALSE
0.02753445	19	951	6	0.006309148	0.315789474	GO:0042910	GO:MF	xenobiotic transmembrane transporter activity	FALSE
0.029838336	46	951	10	0.010515247	0.217391304	GO:0019838	GO:MF	growth factor binding	FALSE
0.033057673	14	951	5	0.005257624	0.357142857	GO:0099604	GO:MF	ligand-gated calcium channel activity	FALSE
0.033057673	14	951	5	0.005257624	0.357142857	GO:0042805	GO:MF	actinin binding	FALSE
0.033729804	78	951	14	0.014721346	0.179487179	GO:1901681	GO:MF	sulfur compound binding	FALSE
0.034537671	615	951	69	0.072555205	0.112195122	GO:0017111	GO:MF	ribonucleoside triphosphate phosphatase activity	FALSE
0.035079505	20	951	6	0.006309148	0.3	GO:0140828	GO:MF	metal cation:monoatomic cation antiporter activity	FALSE
0.035315496	33	951	8	0.008412198	0.242424242	GO:0008235	GO:MF	metalloexopeptidase activity	FALSE
0.037228997	63	951	12	0.012618297	0.19047619	GO:0004620	GO:MF	phospholipase activity	FALSE
0.038193339	5	951	3	0.003154574	0.6	GO:0008510	GO:MF	sodium:bicarbonate symporter activity	FALSE
0.038193339	5	951	3	0.003154574	0.6	GO:0015431	GO:MF	ABC-type glutathione S-conjugate transporter activity	FALSE
0.038193339	5	951	3	0.003154574	0.6	GO:0034584	GO:MF	piRNA binding	TRUE
0.038193339	5	951	3	0.003154574	0.6	GO:0042937	GO:MF	tripeptide transmembrane transporter activity	FALSE
0.038193339	5	951	3	0.003154574	0.6	GO:0048763	GO:MF	calcium-induced calcium release activity	FALSE
0.042558648	15	951	5	0.005257624	0.333333333	GO:0009975	GO:MF	cyclase activity	FALSE
0.042558648	15	951	5	0.005257624	0.333333333	GO:0016849	GO:MF	phosphorus-oxygen lyase activity	FALSE
0.045639029	246	951	32	0.033648791	0.130081301	GO:0016853	GO:MF	isomerase activity	FALSE
0.047112151	10	951	4	0.004206099	0.4	GO:0004016	GO:MF	adenylate cyclase activity	FALSE
0.047112151	10	951	4	0.004206099	0.4	GO:0015385	GO:MF	sodium:proton antiporter activity	FALSE
0.047112151	10	951	4	0.004206099	0.4	GO:0051393	GO:MF	alpha-actinin binding	FALSE
0.047112151	10	951	4	0.004206099	0.4	GO:0005310	GO:MF	dicarboxylic acid transmembrane transporter activity	FALSE

0.047112151	10	951	4	0.004206099	0.4	GO:0008559	GO:MF	ABC-type xenobiotic transporter activity	FALSE
0.047551007	2	951	2	0.002103049	1	GO:0062063	GO:MF	BBSome binding	TRUE
0.047551007	2	951	2	0.002103049	1	GO:0048101	GO:MF	calmodulin-activated 3',5'-cyclic-GMP phosphodiesterase activity	TRUE
0.047551007	2	951	2	0.002103049	1	GO:0004117	GO:MF	calmodulin-activated dual specificity 3',5'-cyclic-GMP, 3',5'-cyclic-AMP phosphodiesterase activity	TRUE
0.047551007	2	951	2	0.002103049	1	GO:0005314	GO:MF	high-affinity L-glutamate transmembrane transporter activity	FALSE
0.047551007	2	951	2	0.002103049	1	GO:0016434	GO:MF	rRNA (cytosine) methyltransferase activity	TRUE
0.047551007	2	951	2	0.002103049	1	GO:0005427	GO:MF	proton-dependent oligopeptide secondary active transmembrane transporter activity	FALSE
0.047551007	2	951	2	0.002103049	1	GO:0097603	GO:MF	temperature-gated ion channel activity	FALSE
0.047551007	2	951	2	0.002103049	1	GO:0008066	GO:MF	glutamate receptor activity	FALSE
0.047551007	2	951	2	0.002103049	1	GO:0015501	GO:MF	glutamate:sodium symporter activity	FALSE
4.94E-15	56	951	29	0.030494217	0.517857143	KEGG:04512	KEGG	ECM-receptor interaction	FALSE
6.94E-12	53	951	25	0.026288118	0.471698113	KEGG:04974	KEGG	Protein digestion and absorption	FALSE
8.80E-09	55	951	22	0.023133544	0.4	KEGG:05412	KEGG	Arrhythmogenic right ventricular cardiomyopathy	FALSE
8.74E-08	120	951	32	0.033648791	0.266666667	KEGG:04814	KEGG	Motor proteins	FALSE
6.40E-06	59	951	19	0.01997897	0.322033898	KEGG:05410	KEGG	Hypertrophic cardiomyopathy	FALSE
7.21E-06	60	951	19	0.01997897	0.316666667	KEGG:05414	KEGG	Dilated cardiomyopathy	FALSE
1.44E-05	31	951	13	0.013669821	0.419354839	KEGG:02010	KEGG	ABC transporters	FALSE
3.30E-05	141	951	30	0.031545741	0.212765957	KEGG:04510	KEGG	Focal adhesion	FALSE
3.39E-05	211	951	39	0.041009464	0.184834123	KEGG:04151	KEGG	PI3K-Akt signaling pathway	FALSE
0.000154004	38	951	13	0.013669821	0.342105263	KEGG:04976	KEGG	Bile secretion	FALSE
0.000260908	64	951	17	0.01787592	0.265625	KEGG:05146	KEGG	Amoebiasis	FALSE
0.000967405	51	951	14	0.014721346	0.274509804	KEGG:04972	KEGG	Pancreatic secretion	FALSE
0.001215204	40	951	12	0.012618297	0.3	KEGG:04610	KEGG	Complement and coagulation cascades	FALSE
0.006884642	41	951	11	0.011566772	0.268292683	KEGG:04924	KEGG	Renin secretion	FALSE
0.009288581	4554	951	421	0.442691903	0.092446201	KEGG:00000	KEGG	KEGG root term	FALSE
0.010988112	58	951	13	0.013669821	0.224137931	KEGG:04080	KEGG	Neuroactive ligand-receptor interaction	FALSE
0.010988112	112	951	20	0.021030494	0.178571429	KEGG:04020	KEGG	Calcium signaling pathway	FALSE
0.010988112	65	951	14	0.014721346	0.215384615	KEGG:04514	KEGG	Cell adhesion molecules	FALSE
0.010988112	20	951	7	0.007360673	0.35	KEGG:04742	KEGG	Taste transduction	FALSE
0.011427074	97	951	18	0.018927445	0.18556701	KEGG:04360	KEGG	Axon guidance	FALSE
0.012111814	210	951	31	0.032597266	0.147619048	KEGG:05165	KEGG	Human papillomavirus infection	FALSE
0.012111814	39	951	10	0.010515247	0.256410256	KEGG:04911	KEGG	Insulin secretion	FALSE
0.014176496	40	951	10	0.010515247	0.25	KEGG:04721	KEGG	Synaptic vesicle cycle	FALSE
0.016487954	55	951	12	0.012618297	0.218181818	KEGG:04726	KEGG	Serotonergic synapse	FALSE
0.019433433	49	951	11	0.011566772	0.224489796	KEGG:04713	KEGG	Circadian entrainment	FALSE
0.023862284	81	951	15	0.015772871	0.185185185	KEGG:00230	KEGG	Purine metabolism	FALSE
0.029772446	52	951	11	0.011566772	0.211538462	KEGG:04724	KEGG	Glutamatergic synapse	FALSE
0.036043769	32	951	8	0.008412198	0.25	KEGG:04979	KEGG	Cholesterol metabolism	FALSE

Green Module GO Enrichment									
p value	*term size*	*query size*	*overlap size*	*precision*	*recall*	*term id*	*source*	*term name*	*highlighted*
1.35E-07	5049	744	410	0.551075269	0.081204199	GO:0065007	GO:BP	biological regulation	TRUE
8.75E-07	3470	744	299	0.40188172	0.086167147	GO:0050896	GO:BP	response to stimulus	FALSE
1.20E-06	4906	744	394	0.529569892	0.080309825	GO:0050789	GO:BP	regulation of biological process	FALSE
1.30E-06	4750	744	383	0.514784946	0.080631579	GO:0050794	GO:BP	regulation of cellular process	FALSE
2.76E-05	2729	744	238	0.319892473	0.087211433	GO:0048518	GO:BP	positive regulation of biological process	FALSE
4.67E-05	2949	744	252	0.338709677	0.085452696	GO:0051716	GO:BP	cellular response to stimulus	FALSE
7.52E-05	1102	744	114	0.153225806	0.103448276	GO:0002376	GO:BP	immune system process	FALSE
7.84E-05	2466	744	216	0.290322581	0.087591241	GO:0023052	GO:BP	signaling	FALSE
9.25E-05	2590	744	224	0.301075269	0.086486486	GO:0048522	GO:BP	positive regulation of cellular process	FALSE
9.25E-05	2476	744	216	0.290322581	0.08723748	GO:0007154	GO:BP	cell communication	FALSE
9.39E-05	2314	744	204	0.274193548	0.088159032	GO:0007165	GO:BP	signal transduction	FALSE
0.000127993	613	744	72	0.096774194	0.117455139	GO:0016477	GO:BP	cell migration	FALSE
0.00016425	248	744	38	0.051075269	0.153225806	GO:0005975	GO:BP	carbohydrate metabolic process	TRUE
0.00019028	73	744	18	0.024193548	0.246575342	GO:0016051	GO:BP	carbohydrate biosynthetic process	FALSE
0.000250851	2284	744	199	0.267473118	0.087127846	GO:0048523	GO:BP	negative regulation of cellular process	FALSE
0.000278039	2347	744	203	0.272849462	0.086493396	GO:0048519	GO:BP	negative regulation of biological process	FALSE
0.000278039	2472	744	212	0.284946237	0.085760518	GO:0051179	GO:BP	localization	TRUE
0.000302229	693	744	77	0.103494624	0.111111111	GO:0048870	GO:BP	cell motility	FALSE
0.000462955	2522	744	214	0.287634409	0.084853291	GO:0032501	GO:BP	multicellular organismal process	FALSE
0.001081818	482	744	57	0.076612903	0.118257261	GO:0030097	GO:BP	hemopoiesis	FALSE
0.001081818	1938	744	170	0.228494624	0.087719298	GO:0006810	GO:BP	transport	FALSE
0.001576207	777	744	81	0.108870968	0.104247104	GO:0016192	GO:BP	vesicle-mediated transport	FALSE
0.002042589	2115	744	181	0.24327957	0.085579196	GO:0051234	GO:BP	establishment of localization	FALSE
0.002339342	520	744	59	0.079301075	0.113461538	GO:0001775	GO:BP	cell activation	FALSE
0.002417522	1736	744	153	0.205645161	0.088133641	GO:0048583	GO:BP	regulation of response to stimulus	FALSE
0.003606882	470	744	54	0.072580645	0.114893617	GO:0045321	GO:BP	leukocyte activation	FALSE
0.003803333	1294	744	119	0.159946237	0.091962906	GO:0042221	GO:BP	response to chemical	FALSE
0.003803333	1470	744	132	0.177419355	0.089795918	GO:0010646	GO:BP	regulation of cell communication	FALSE
0.003803333	2241	744	188	0.252688172	0.08389112	GO:0009889	GO:BP	regulation of biosynthetic process	FALSE
0.003803333	2801	744	227	0.305107527	0.081042485	GO:0019222	GO:BP	regulation of metabolic process	FALSE
0.003831976	103	744	19	0.025537634	0.184466019	GO:0019318	GO:BP	hexose metabolic process	FALSE
0.003831976	315	744	40	0.053763441	0.126984127	GO:0002521	GO:BP	leukocyte differentiation	FALSE
0.003831976	168	744	26	0.034946237	0.154761905	GO:0016049	GO:BP	cell growth	TRUE
0.003831976	1474	744	132	0.177419355	0.089552239	GO:0023051	GO:BP	regulation of signaling	FALSE
0.004993	2133	744	179	0.240591398	0.083919362	GO:0010468	GO:BP	regulation of gene expression	FALSE
0.005372887	2179	744	182	0.244623656	0.083524553	GO:0010556	GO:BP	regulation of macromolecule biosynthetic process	FALSE
0.006736918	336	744	41	0.055107527	0.12202381	GO:0040007	GO:BP	growth	FALSE
0.006777524	720	744	73	0.09811828	0.101388889	GO:0010647	GO:BP	positive regulation of cell communication	FALSE

0.008629505	727	744	73	0.09811828	0.100412655	GO:0023056	GO:BP	positive regulation of signaling	FALSE
0.008629505	179	744	26	0.034946237	0.145251397	GO:0006869	GO:BP	lipid transport	FALSE
0.008629505	189	744	27	0.036290323	0.142857143	GO:0010876	GO:BP	lipid localization	FALSE
0.008629505	1301	744	117	0.157258065	0.089930822	GO:0009056	GO:BP	catabolic process	TRUE
0.008692788	2561	744	207	0.278225806	0.080827802	GO:0060255	GO:BP	regulation of macromolecule metabolic process	FALSE
0.009367388	606	744	63	0.084677419	0.103960396	GO:0042127	GO:BP	regulation of cell population proliferation	FALSE
0.009367388	112	744	19	0.025537634	0.169642857	GO:0005996	GO:BP	monosaccharide metabolic process	FALSE
0.010524503	369	744	43	0.057795699	0.116531165	GO:0098657	GO:BP	import into cell	FALSE
0.011587857	2247	744	184	0.247311828	0.08188696	GO:0048856	GO:BP	anatomical structure development	FALSE
0.011988622	1010	744	94	0.126344086	0.093069307	GO:0048584	GO:BP	positive regulation of response to stimulus	FALSE
0.013283213	818	744	79	0.106182796	0.096577017	GO:0141124	GO:BP	intracellular signaling cassette	FALSE
0.014000961	667	744	67	0.090053763	0.100449775	GO:0009967	GO:BP	positive regulation of signal transduction	FALSE
0.01434793	240	744	31	0.041666667	0.129166667	GO:0030099	GO:BP	myeloid cell differentiation	FALSE
0.014952577	1602	744	137	0.184139785	0.085518102	GO:0048869	GO:BP	cellular developmental process	FALSE
0.014952577	1602	744	137	0.184139785	0.085518102	GO:0030154	GO:BP	cell differentiation	FALSE
0.014952577	546	744	57	0.076612903	0.104395604	GO:1901700	GO:BP	response to oxygen-containing compound	FALSE
0.014952577	811	744	78	0.10483871	0.096177559	GO:0070887	GO:BP	cellular response to chemical stimulus	FALSE
0.015241726	81	744	15	0.02016129	0.185185185	GO:0006006	GO:BP	glucose metabolic process	FALSE
0.01665936	1333	744	117	0.157258065	0.087771943	GO:0009966	GO:BP	regulation of signal transduction	FALSE
0.016758942	1361	744	119	0.159946237	0.087435709	GO:0006355	GO:BP	regulation of DNA-templated transcription	FALSE
0.017786678	10	744	5	0.00672043	0.5	GO:0050832	GO:BP	defense response to fungus	TRUE
0.018101595	101	744	17	0.022849462	0.168316832	GO:0001667	GO:BP	ameboidal-type cell migration	FALSE
0.019085675	1367	744	119	0.159946237	0.087051939	GO:2001141	GO:BP	regulation of RNA biosynthetic process	FALSE
0.019317099	1840	744	153	0.205645161	0.083152174	GO:0019538	GO:BP	protein metabolic process	TRUE
0.019317099	2409	744	193	0.259408602	0.080116231	GO:0032502	GO:BP	developmental process	FALSE
0.019621636	1086	744	98	0.13172043	0.090239411	GO:0007166	GO:BP	cell surface receptor signaling pathway	FALSE
0.019621636	42	744	10	0.01344086	0.238095238	GO:0042982	GO:BP	amyloid precursor protein metabolic process	FALSE
0.020380632	3	744	3	0.004032258	1	GO:2001286	GO:BP	regulation of caveolin-mediated endocytosis	TRUE
0.021171806	423	744	46	0.061827957	0.108747045	GO:1901701	GO:BP	cellular response to oxygen-containing compound	FALSE
0.021289518	1443	744	124	0.166666667	0.085932086	GO:0006351	GO:BP	DNA-templated transcription	FALSE
0.021913615	762	744	73	0.09811828	0.095800525	GO:0008283	GO:BP	cell population proliferation	FALSE
0.022457236	16	744	6	0.008064516	0.375	GO:0051703	GO:BP	biological process involved in intraspecies interaction between organisms	TRUE
0.022457236	437	744	47	0.063172043	0.107551487	GO:0040012	GO:BP	regulation of locomotion	FALSE
0.024044752	403	744	44	0.059139785	0.109181141	GO:0046649	GO:BP	lymphocyte activation	FALSE
0.024044752	588	744	59	0.079301075	0.100340136	GO:0045595	GO:BP	regulation of cell differentiatio	FALSE

0.024044752	145	744	21	0.028225806	0.144827586	GO:0007033	GO:BP	vacuole organization	TRUE
0.024044752	910	744	84	0.112903226	0.092307692	GO:0009605	GO:BP	response to external stimulus	FALSE
0.024044752	309	744	36	0.048387097	0.116504854	GO:0030036	GO:BP	actin cytoskeleton organization	TRUE
0.024902256	357	744	40	0.053763441	0.112044818	GO:0030155	GO:BP	regulation of cell adhesion	FALSE
0.028732747	211	744	27	0.036290323	0.127962085	GO:0019221	GO:BP	cytokine-mediated signaling pathway	FALSE
0.029597377	1051	744	94	0.126344086	0.08943863	GO:0006366	GO:BP	transcription by RNA polymerase II	FALSE
0.029621506	1420	744	121	0.162634409	0.085211268	GO:0035556	GO:BP	intracellular signal transduction	FALSE
0.029621506	774	744	73	0.09811828	0.094315245	GO:0048585	GO:BP	negative regulation of response to stimulus	FALSE
0.029621506	212	744	27	0.036290323	0.127358491	GO:0045785	GO:BP	positive regulation of cell adhesion	FALSE
0.029780124	1080	744	96	0.129032258	0.088888889	GO:0065008	GO:BP	regulation of biological quality	FALSE
0.029780124	422	744	45	0.060483871	0.106635071	GO:2000145	GO:BP	regulation of cell motility	FALSE
0.029780124	138	744	20	0.02688172	0.144927536	GO:0001558	GO:BP	regulation of cell growth	FALSE
0.029884337	987	744	89	0.119623656	0.090172239	GO:0006357	GO:BP	regulation of transcription by RNA polymerase II	FALSE
0.032959843	561	744	56	0.075268817	0.099821747	GO:0007155	GO:BP	cell adhesion	FALSE
0.032959843	727	744	69	0.092741935	0.094910591	GO:0002682	GO:BP	regulation of immune system process	FALSE
0.032967389	2304	744	183	0.245967742	0.079427083	GO:0080090	GO:BP	regulation of primary metabolic process	FALSE
0.032975687	689	744	66	0.088709677	0.095791001	GO:0006952	GO:BP	defense response	FALSE
0.03467135	354	744	39	0.052419355	0.110169492	GO:0071345	GO:BP	cellular response to cytokine stimulus	FALSE
0.034836138	1696	744	140	0.188172043	0.08254717	GO:0006950	GO:BP	response to stress	FALSE
0.034836138	18	744	6	0.008064516	0.333333333	GO:0009250	GO:BP	glucan biosynthetic process	FALSE
0.034836138	18	744	6	0.008064516	0.333333333	GO:0005978	GO:BP	glycogen biosynthetic process	FALSE
0.034836138	1170	744	102	0.137096774	0.087179487	GO:0051239	GO:BP	regulation of multicellular organismal process	FALSE
0.03654617	392	744	42	0.056451613	0.107142857	GO:0034097	GO:BP	response to cytokine	FALSE
0.03665403	309	744	35	0.047043011	0.113268608	GO:0006897	GO:BP	endocytosis	FALSE
0.03665403	274	744	32	0.043010753	0.116788321	GO:0042110	GO:BP	T cell activation	FALSE
0.037316193	393	744	42	0.056451613	0.106870229	GO:1901652	GO:BP	response to peptide	FALSE
0.040810385	1123	744	98	0.13172043	0.087266251	GO:0048468	GO:BP	cell development	FALSE
0.044042588	897	744	81	0.108870968	0.090301003	GO:1902531	GO:BP	regulation of intracellular signal transduction	FALSE
0.044042588	33	744	8	0.010752688	0.242424242	GO:0046323	GO:BP	D-glucose import	TRUE
0.044042588	13	744	5	0.00672043	0.384615385	GO:0034249	GO:BP	negative regulation of amide metabolic process	TRUE
0.044042588	13	744	5	0.00672043	0.384615385	GO:0001774	GO:BP	microglial cell activation	TRUE
0.044042588	33	744	8	0.010752688	0.242424242	GO:0042987	GO:BP	amyloid precursor protein catabolic process	FALSE
0.04513442	8	744	4	0.005376344	0.5	GO:1903319	GO:BP	positive regulation of protein maturation	FALSE
0.04513442	8	744	4	0.005376344	0.5	GO:0045725	GO:BP	positive regulation of glycogen biosynthetic process	FALSE
0.04513442	8	744	4	0.005376344	0.5	GO:0010954	GO:BP	positive regulation of protein processing	TRUE

0.045337648	4	744	3	0.004032258	0.75	GO:0140213	GO:BP	negative regulation of long-chain fatty acid import into cell	FALSE
0.045337648	26	744	7	0.009408602	0.269230769	GO:0000271	GO:BP	polysaccharide biosynthetic process	FALSE
0.045337648	4	744	3	0.004032258	0.75	GO:2000192	GO:BP	negative regulation of fatty acid transport	FALSE
0.045337648	4	744	3	0.004032258	0.75	GO:0010746	GO:BP	regulation of long-chain fatty acid import across plasma membrane	FALSE
0.045337648	4	744	3	0.004032258	0.75	GO:0010748	GO:BP	negative regulation of long-chain fatty acid import across plasma membrane	TRUE
0.046653146	304	744	34	0.045698925	0.111842105	GO:0006954	GO:BP	inflammatory response	FALSE
0.047457411	201	744	25	0.033602151	0.124378109	GO:1903706	GO:BP	regulation of hemopoiesis	FALSE
4.12E-18	5580	744	480	0.64516129	0.086021505	GO:0005737	GO:CC	cytoplasm	TRUE
8.68E-09	3584	744	312	0.419354839	0.087053571	GO:0016020	GO:CC	membrane	FALSE
8.49E-08	1933	744	188	0.252688172	0.097258148	GO:0012505	GO:CC	endomembrane system	FALSE
2.54E-06	1403	744	141	0.189516129	0.100498931	GO:0031090	GO:CC	organelle membrane	FALSE
2.81E-06	318	744	48	0.064516129	0.150943396	GO:0005773	GO:CC	vacuole	FALSE
4.22E-05	879	744	94	0.126344086	0.106939704	GO:0031982	GO:CC	vesicle	FALSE
6.92E-05	818	744	88	0.11827957	0.107579462	GO:0031410	GO:CC	cytoplasmic vesicle	FALSE
7.08E-05	821	744	88	0.11827957	0.107186358	GO:0097708	GO:CC	intracellular vesicle	FALSE
9.72E-05	260	744	38	0.051075269	0.146153846	GO:0000323	GO:CC	lytic vacuole	FALSE
9.72E-05	260	744	38	0.051075269	0.146153846	GO:0005764	GO:CC	lysosome	FALSE
0.000150032	461	744	56	0.075268817	0.121475054	GO:0005768	GO:CC	endosome	FALSE
0.000318912	748	744	79	0.106182796	0.105614973	GO:0098588	GO:CC	bounding membrane of organelle	FALSE
0.002478765	819	744	81	0.108870968	0.098901099	GO:0005783	GO:CC	endoplasmic reticulum	FALSE
0.003697706	1925	744	162	0.217741935	0.084155844	GO:0071944	GO:CC	cell periphery	FALSE
0.003697706	705	744	71	0.095430108	0.10070922	GO:0005794	GO:CC	Golgi apparatus	FALSE
0.004378675	2131	744	176	0.23655914	0.082590333	GO:0005829	GO:CC	cytosol	FALSE
0.005896124	1765	744	149	0.200268817	0.084419263	GO:0005886	GO:CC	plasma membrane	FALSE
0.01001034	2171	744	176	0.23655914	0.081068632	GO:0005654	GO:CC	nucleoplasm	TRUE
0.01001034	375	744	42	0.056451613	0.112	GO:0012506	GO:CC	vesicle membrane	FALSE
0.017233285	7	744	4	0.005376344	0.571428571	GO:0070776	GO:CC	MOZ/MORF histone acetyltransferase complex	TRUE
0.017233285	99	744	16	0.021505376	0.161616162	GO:0005925	GO:CC	focal adhesion	TRUE
0.019679942	366	744	40	0.053763441	0.109289617	GO:0030659	GO:CC	cytoplasmic vesicle membrane	FALSE
0.020729382	217	744	27	0.036290323	0.124423963	GO:0010008	GO:CC	endosome membrane	FALSE
0.022287692	443	744	46	0.061827957	0.103837472	GO:0005789	GO:CC	endoplasmic reticulum membrane	FALSE
0.024688913	41	744	9	0.012096774	0.219512195	GO:0042641	GO:CC	actomyosin	TRUE
0.024688913	104	744	16	0.021505376	0.153846154	GO:0030055	GO:CC	cell-substrate junction	FALSE
0.024688913	8	744	4	0.005376344	0.5	GO:0070775	GO:CC	H3 histone acetyltransferase complex	FALSE
0.025368431	449	744	46	0.061827957	0.102449889	GO:0098827	GO:CC	endoplasmic reticulum subcompartment	FALSE
0.029442901	453	744	46	0.061827957	0.101545254	GO:0042175	GO:CC	nuclear outer membrane-endoplasmic reticulum membrane network	FALSE
0.029633142	51	744	10	0.01344086	0.196078431	GO:0035097	GO:CC	histone methyltransferase complex	FALSE
0.029646122	14	744	5	0.00672043	0.357142857	GO:0005847	GO:CC	mRNA cleavage and polyadenylation specificity factor complex	TRUE
0.035280487	44	744	9	0.012096774	0.204545455	GO:0032587	GO:CC	ruffle membrane	TRUE

0.04121758	37	744	8	0.010752688	0.216216216	GO:0001725	GO:CC	stress fiber	FALSE
0.04121758	37	744	8	0.010752688	0.216216216	GO:0097517	GO:CC	contractile actin filament bundle	FALSE
0.045040426	83	744	13	0.017473118	0.156626506	GO:1904949	GO:CC	ATPase complex	TRUE
0.048757168	10	744	4	0.005376344	0.4	GO:0150005	GO:CC	enzyme activator complex	TRUE
0.048757168	65	744	11	0.014784946	0.169230769	GO:0070603	GO:CC	SWI/SNF superfamily-type complex	FALSE
0.000104332	3253	744	272	0.365591398	0.083615125	GO:0005515	GO:MF	protein binding	TRUE
0.001022898	3652	744	292	0.392473118	0.079956188	GO:0003824	GO:MF	catalytic activity	TRUE
0.007352273	993	744	96	0.129032258	0.096676737	GO:0042802	GO:MF	identical protein binding	FALSE
0.007352273	1115	744	105	0.141129032	0.094170404	GO:0019899	GO:MF	enzyme binding	FALSE
0.007352273	134	744	22	0.029569892	0.164179104	GO:0016747	GO:MF	acyltransferase activity, transferring groups other than amino-acyl groups	FALSE
0.007352273	134	744	22	0.029569892	0.164179104	GO:0003713	GO:MF	transcription coactivator activity	TRUE
0.007488832	619	744	65	0.087365591	0.105008078	GO:0060090	GO:MF	molecular adaptor activity	FALSE
0.008209189	253	744	33	0.044354839	0.130434783	GO:0003712	GO:MF	transcription coregulator activity	FALSE
0.008209189	51	744	12	0.016129032	0.235294118	GO:0016410	GO:MF	N-acyltransferase activity	FALSE
0.008209189	1519	744	133	0.178763441	0.087557604	GO:0140096	GO:MF	catalytic activity, acting on a protein	FALSE
0.014719829	556	744	58	0.077956989	0.104316547	GO:0030674	GO:MF	protein-macromolecule adaptor activity	FALSE
0.019297693	10	744	5	0.00672043	0.5	GO:0043997	GO:MF	histone H4K12 acetyltransferase activity	FALSE
0.02336576	28	744	8	0.010752688	0.285714286	GO:0004402	GO:MF	histone acetyltransferase activity	FALSE
0.025131963	16	744	6	0.008064516	0.375	GO:0010485	GO:MF	histone H4 acetyltransferase activity	FALSE
0.026758348	664	744	65	0.087365591	0.097891566	GO:0140110	GO:MF	transcription regulator activity	FALSE
0.029295601	266	744	32	0.043010753	0.120300752	GO:0140297	GO:MF	DNA-binding transcription factor binding	FALSE
0.030018434	30	744	8	0.010752688	0.266666667	GO:0061733	GO:MF	protein-lysine-acetyltransferase activity	FALSE
0.033338111	316	744	36	0.048387097	0.113924051	GO:0008134	GO:MF	transcription factor binding	FALSE
0.034385125	12	744	5	0.00672043	0.416666667	GO:0042800	GO:MF	histone H3K4 methyltransferase activity	TRUE
0.038971463	1657	744	137	0.184139785	0.082679541	GO:0016787	GO:MF	hydrolase activity	FALSE
0.042096677	40	744	9	0.012096774	0.225	GO:0008080	GO:MF	N-acetyltransferase activity	FALSE
0.04638518	33	744	8	0.010752688	0.242424242	GO:0034212	GO:MF	protein N-acetyltransferase activity	FALSE
0.049152927	4	744	3	0.004032258	0.75	GO:0036041	GO:MF	long-chain fatty acid binding	TRUE
6.11E-05	4554	744	358	0.481182796	0.078612209	KEGG:00000	KEGG	KEGG root term	FALSE
0.003633087	133	744	22	0.029569892	0.165413534	KEGG:05203	KEGG	Viral carcinogenesis	FALSE
0.003633087	33	744	10	0.01344086	0.303030303	KEGG:00510	KEGG	N-Glycan biosynthesis	FALSE
0.04136311	42	744	9	0.012096774	0.214285714	KEGG:04330	KEGG	Notch signaling pathway	FALSE
0.04136311	48	744	10	0.01344086	0.208333333	KEGG:03250	KEGG	Viral life cycle - HIV-1	FALSE
0.04136311	76	744	13	0.017473118	0.171052632	KEGG:04666	KEGG	Fc gamma R-mediated phagocytosis	FALSE
0.04136311	87	744	14	0.018817204	0.16091954	KEGG:03082	KEGG	ATP-dependent chromatin remodeling	FALSE
0.04136311	25	744	7	0.009408602	0.28	KEGG:00513	KEGG	Various types of N-glycan biosynthesis	FALSE
0.04136311	146	744	20	0.02688172	0.136986301	KEGG:04810	KEGG	Regulation of actin cytoskeleton	FALSE
0.049847922	45	744	9	0.012096774	0.2	KEGG:00561	KEGG	Glycerolipid metabolism	FALSE

0.049847922	101	744	15	0.02016129	0.148514851	KEGG:04142	KEGG	Lysosome	FALSE
0.049847922	100	744	15	0.02016129	0.15	KEGG:04530	KEGG	Tight junction	FALSE

Greenyellow Module GO Enrichment

p value	*term size*	*query size*	*overlap size*	*precision*	*recall*	*term id*	*source*	*term name*	*highlighted*
3.14E-05	4750	134	87	0.649253731	0.018315789	GO:0050794	GO:BP	regulation of cellular process	TRUE
3.55E-05	4906	134	88	0.656716418	0.01793722	GO:0050789	GO:BP	regulation of biological process	FALSE
4.45E-05	5049	134	89	0.664179104	0.017627253	GO:0065007	GO:BP	biological regulation	FALSE
0.015325748	2314	134	47	0.350746269	0.02031115	GO:0007165	GO:BP	signal transduction	FALSE
0.028873784	67	134	6	0.044776119	0.089552239	GO:0106106	GO:BP	cold-induced thermogenesis	TRUE
0.028873784	777	134	22	0.164179104	0.028314028	GO:0016192	GO:BP	vesicle-mediated transport	FALSE
0.028873784	369	134	14	0.104477612	0.037940379	GO:0098657	GO:BP	import into cell	TRUE
0.028873784	2466	134	47	0.350746269	0.019059205	GO:0023052	GO:BP	signaling	FALSE
0.028873784	1420	134	32	0.23880597	0.022535211	GO:0035556	GO:BP	intracellular signal transduction	FALSE
0.028873784	2	134	2	0.014925373	1	GO:0038159	GO:BP	C-X-C chemokine receptor CXCR4 signaling pathway	TRUE
0.028873784	67	134	6	0.044776119	0.089552239	GO:0120161	GO:BP	regulation of cold-induced thermogenesis	FALSE
0.029278996	2476	134	47	0.350746269	0.018982229	GO:0007154	GO:BP	cell communication	FALSE
0.030585133	72	134	6	0.044776119	0.083333333	GO:1990845	GO:BP	adaptive thermogenesis	FALSE
0.032415804	2949	134	53	0.395522388	0.017972194	GO:0051716	GO:BP	cellular response to stimulus	FALSE
0.035305725	309	134	12	0.089552239	0.038834951	GO:0006897	GO:BP	endocytosis	FALSE
0.046764509	3470	134	59	0.440298507	0.017002882	GO:0050896	GO:BP	response to stimulus	FALSE
0.046764509	3	134	2	0.014925373	0.666666667	GO:0021849	GO:BP	neuroblast division in subventricular zone	TRUE
0.046764509	83	134	6	0.044776119	0.072289157	GO:0001659	GO:BP	temperature homeostasis	FALSE
0.046764509	3	134	2	0.014925373	0.666666667	GO:0007625	GO:BP	grooming behavior	TRUE
0.04761192	2729	134	49	0.365671642	0.017955295	GO:0048518	GO:BP	positive regulation of biological process	FALSE
0.047645219	1938	134	38	0.28358209	0.019607843	GO:0006810	GO:BP	transport	FALSE
0.047645219	2590	134	47	0.350746269	0.018146718	GO:0048522	GO:BP	positive regulation of cellular process	FALSE
0.000500827	1765	134	42	0.313432836	0.023796034	GO:0005886	GO:CC	plasma membrane	TRUE
0.000963069	1925	134	43	0.320895522	0.022337662	GO:0071944	GO:CC	cell periphery	FALSE
0.005596345	3584	134	63	0.470149254	0.017578125	GO:0016020	GO:CC	membrane	FALSE
0.011814132	821	134	21	0.156716418	0.025578563	GO:0097708	GO:CC	intracellular vesicle	FALSE
0.011814132	879	134	22	0.164179104	0.025028441	GO:0031982	GO:CC	vesicle	FALSE
0.011814132	5580	134	85	0.634328358	0.015232975	GO:0005737	GO:CC	cytoplasm	FALSE
0.011814132	366	134	13	0.097014925	0.035519126	GO:0030659	GO:CC	cytoplasmic vesicle membrane	FALSE
0.011814132	461	134	15	0.111940299	0.032537961	GO:0005768	GO:CC	endosome	FALSE
0.011814132	375	134	13	0.097014925	0.034666667	GO:0012506	GO:CC	vesicle membrane	FALSE
0.011814132	818	134	21	0.156716418	0.025672372	GO:0031410	GO:CC	cytoplasmic vesicle	TRUE
0.011814132	73	134	6	0.044776119	0.082191781	GO:0009898	GO:CC	cytoplasmic side of plasma membrane	FALSE
0.019238704	95	134	6	0.044776119	0.063157895	GO:0098562	GO:CC	cytoplasmic side of membrane	FALSE
0.020431819	217	134	9	0.067164179	0.041474654	GO:0010008	GO:CC	endosome membrane	FALSE
0.029818936	72	134	5	0.037313433	0.069444444	GO:0019898	GO:CC	extrinsic component of membrane	FALSE
0.045241589	748	134	18	0.134328358	0.024064171	GO:0098588	GO:CC	bounding membrane of organelle	FALSE
0.048848108	7	134	2	0.014925373	0.285714286	GO:0097651	GO:CC	phosphatidylinositol 3-kinase complex, class I	TRUE

0.006198584	113	134	8	0.059701493	0.07079646	KEGG:04062	KEGG	Chemokine signaling pathway	FALSE
0.006198584	189	134	10	0.074626866	0.052910053	KEGG:04144	KEGG	Endocytosis	FALSE
0.014104273	4554	134	73	0.544776119	0.016029864	KEGG:00000	KEGG	KEGG root term	FALSE
0.025051343	88	134	6	0.044776119	0.068181818	KEGG:04611	KEGG	Platelet activation	FALSE
0.030133831	131	134	7	0.052238806	0.053435115	KEGG:05167	KEGG	Kaposi sarcoma-associated herpesvirus infection	FALSE

Magenta Module GO Enrichment

p value	*term size*	*query size*	*overlap size*	*precision*	*recall*	*term id*	*source*	*term name*	*highlighted*
0.000364719	35	159	7	0.044025157	0.2	GO:0035914	GO:BP	skeletal muscle cell differentiation	FALSE
0.000364719	251	159	16	0.100628931	0.06374502	GO:0061061	GO:BP	muscle structure development	TRUE
0.000364719	368	159	20	0.125786164	0.054347826	GO:0006412	GO:BP	translation	TRUE
0.001039135	64	159	8	0.050314465	0.125	GO:0002181	GO:BP	cytoplasmic translation	FALSE
0.001039135	86	159	9	0.056603774	0.104651163	GO:0001666	GO:BP	response to hypoxia	TRUE
0.001039135	87	159	9	0.056603774	0.103448276	GO:0036293	GO:BP	response to decreased oxygen levels	FALSE
0.001039135	1840	159	49	0.308176101	0.026630435	GO:0019538	GO:BP	protein metabolic process	FALSE
0.001039135	613	159	24	0.150943396	0.039151713	GO:0016477	GO:BP	cell migration	TRUE
0.00110632	1294	159	38	0.238993711	0.029366306	GO:0042221	GO:BP	response to chemical	FALSE
0.001161967	172	159	12	0.075471698	0.069767442	GO:0042254	GO:BP	ribosome biogenesis	TRUE
0.001631291	96	159	9	0.056603774	0.09375	GO:0070482	GO:BP	response to oxygen levels	FALSE
0.00218905	4	159	3	0.018867925	0.75	GO:0060426	GO:BP	lung vasculature development	TRUE
0.00218905	1195	159	35	0.220125786	0.029288703	GO:0009891	GO:BP	positive regulation of biosynthetic process	TRUE
0.002362941	811	159	27	0.169811321	0.033292232	GO:0070887	GO:BP	cellular response to chemical stimulus	FALSE
0.002362941	495	159	20	0.125786164	0.04040404	GO:0040011	GO:BP	locomotion	FALSE
0.002362941	1102	159	33	0.20754717	0.029945554	GO:0002376	GO:BP	immune system process	FALSE
0.002362941	1162	159	34	0.213836478	0.029259897	GO:0010557	GO:BP	positive regulation of macromolecule biosynthetic process	FALSE
0.003340149	554	159	21	0.132075472	0.037906137	GO:0010628	GO:BP	positive regulation of gene expression	FALSE
0.00344935	693	159	24	0.150943396	0.034632035	GO:0048870	GO:BP	cell motility	FALSE
0.003518489	5	159	3	0.018867925	0.6	GO:0002227	GO:BP	innate immune response in mucosa	TRUE
0.004075842	2590	159	58	0.364779874	0.022393822	GO:0048522	GO:BP	positive regulation of cellular process	FALSE
0.004350399	713	159	24	0.150943396	0.033660589	GO:0006955	GO:BP	immune response	FALSE
0.004350399	2729	159	60	0.377358491	0.021986075	GO:0048518	GO:BP	positive regulation of biological process	FALSE
0.004350399	1602	159	41	0.257861635	0.025593009	GO:0048869	GO:BP	cellular developmental process	FALSE
0.004350399	1602	159	41	0.257861635	0.025593009	GO:0030154	GO:BP	cell differentiation	FALSE
0.00530637	71	159	7	0.044025157	0.098591549	GO:0007519	GO:BP	skeletal muscle tissue development	FALSE
0.00530637	124	159	9	0.056603774	0.072580645	GO:0060326	GO:BP	cell chemotaxis	FALSE
0.005851853	31	159	5	0.031446541	0.161290323	GO:0002183	GO:BP	cytoplasmic translational initiation	FALSE
0.006016118	552	159	20	0.125786164	0.036231884	GO:0045944	GO:BP	positive regulation of transcription by RNA polymerase II	FALSE

0.006016118	936	159	28	0.176100629	0.02991453	GO:0045935	GO:BP	positive regulation of nucleobase-containing compound metabolic process	FALSE
0.006016118	839	159	26	0.163522013	0.030989273	GO:0051254	GO:BP	positive regulation of RNA metabolic process	FALSE
0.006016118	74	159	7	0.044025157	0.094594595	GO:0060538	GO:BP	skeletal muscle organ development	FALSE
0.006794385	653	159	22	0.13836478	0.033690658	GO:0051240	GO:BP	positive regulation of multicellular organismal process	FALSE
0.006838163	103	159	8	0.050314465	0.077669903	GO:0045444	GO:BP	fat cell differentiation	FALSE
0.006896197	7	159	3	0.018867925	0.428571429	GO:0140507	GO:BP	granzyme-mediated programmed cell death signaling pathway	TRUE
0.007580298	758	159	24	0.150943396	0.031662269	GO:0045893	GO:BP	positive regulation of DNA-templated transcription	FALSE
0.007682536	760	159	24	0.150943396	0.031578947	GO:1902680	GO:BP	positive regulation of RNA biosynthetic process	FALSE
0.00816024	19	159	4	0.025157233	0.210526316	GO:0032660	GO:BP	regulation of interleukin-17 production	TRUE
0.00816024	19	159	4	0.025157233	0.210526316	GO:0032620	GO:BP	interleukin-17 production	FALSE
0.008689237	57	159	6	0.037735849	0.105263158	GO:0042274	GO:BP	ribosomal small subunit biogenesis	FALSE
0.009102573	8	159	3	0.018867925	0.375	GO:0019731	GO:BP	antibacterial humoral response	TRUE
0.009102573	8	159	3	0.018867925	0.375	GO:0002385	GO:BP	mucosal immune response	FALSE
0.009583531	1470	159	37	0.232704403	0.025170068	GO:0010604	GO:BP	positive regulation of macromolecule metabolic process	FALSE
0.010134676	113	159	8	0.050314465	0.07079646	GO:0007517	GO:BP	muscle organ development	FALSE
0.010457471	688	159	22	0.13836478	0.031976744	GO:0044419	GO:BP	biological process involved in interspecies interaction between organisms	FALSE
0.010457471	21	159	4	0.025157233	0.19047619	GO:0007566	GO:BP	embryo implantation	FALSE
0.010989079	9	159	3	0.018867925	0.333333333	GO:0046697	GO:BP	decidualization	FALSE
0.010989079	1605	159	39	0.245283019	0.024299065	GO:0009893	GO:BP	positive regulation of metabolic process	FALSE
0.010989079	9	159	3	0.018867925	0.333333333	GO:0061844	GO:BP	antimicrobial humoral immune response mediated by antimicrobial peptide	FALSE
0.010989079	744	159	23	0.144654088	0.030913978	GO:0009888	GO:BP	tissue development	FALSE
0.010989079	9	159	3	0.018867925	0.333333333	GO:0002251	GO:BP	organ or tissue specific immune response	FALSE
0.010989079	218	159	11	0.06918239	0.050458716	GO:0009617	GO:BP	response to bacterium	FALSE
0.012383694	1170	159	31	0.194968553	0.026495726	GO:0051239	GO:BP	regulation of multicellular organismal process	FALSE
0.012553743	470	159	17	0.106918239	0.036170213	GO:0045321	GO:BP	leukocyte activation	FALSE
0.013012891	910	159	26	0.163522013	0.028571429	GO:0009605	GO:BP	response to external stimulus	FALSE
0.013520867	520	159	18	0.113207547	0.034615385	GO:0001775	GO:BP	cell activation	FALSE
0.013842626	10	159	3	0.018867925	0.3	GO:0009309	GO:BP	amine biosynthetic process	FALSE
0.013842626	10	159	3	0.018867925	0.3	GO:0042401	GO:BP	biogenic amine biosynthetic process	TRUE
0.014942962	437	159	16	0.100628931	0.036613272	GO:0040012	GO:BP	regulation of locomotion	FALSE
0.014942962	44	159	5	0.031446541	0.113636364	GO:0007565	GO:BP	female pregnancy	FALSE
0.014942962	1247	159	32	0.201257862	0.025661588	GO:0009892	GO:BP	negative regulation of metabolic process	TRUE

0.018572771	403	159	15	0.094339623	0.037220844	GO:0030334	GO:BP	regulation of cell migration	FALSE
0.018572771	164	159	9	0.056603774	0.054878049	GO:0042330	GO:BP	taxis	FALSE
0.018572771	164	159	9	0.056603774	0.054878049	GO:0006935	GO:BP	chemotaxis	FALSE
0.018792549	637	159	20	0.125786164	0.031397174	GO:0009607	GO:BP	response to biotic stimulus	FALSE
0.018792549	47	159	5	0.031446541	0.106382979	GO:0044703	GO:BP	multi-organism reproductive process	FALSE
0.02219214	75	159	6	0.037735849	0.08	GO:0018108	GO:BP	peptidyl-tyrosine phosphorylation	TRUE
0.02219214	75	159	6	0.037735849	0.08	GO:0018212	GO:BP	peptidyl-tyrosine modification	FALSE
0.02219214	75	159	6	0.037735849	0.08	GO:0050730	GO:BP	regulation of peptidyl-tyrosine phosphorylation	FALSE
0.02219214	28	159	4	0.025157233	0.142857143	GO:0030225	GO:BP	macrophage differentiation	TRUE
0.02219214	28	159	4	0.025157233	0.142857143	GO:0031529	GO:BP	ruffle organization	TRUE
0.022384305	171	159	9	0.056603774	0.052631579	GO:0002250	GO:BP	adaptive immune response	FALSE
0.022707713	50	159	5	0.031446541	0.1	GO:0044706	GO:BP	multi-multicellular organism process	FALSE
0.023500896	13	159	3	0.018867925	0.230769231	GO:0001893	GO:BP	maternal placenta development	FALSE
0.023500896	608	159	19	0.119496855	0.03125	GO:0051707	GO:BP	response to other organism	FALSE
0.023500896	13	159	3	0.018867925	0.230769231	GO:0001732	GO:BP	formation of cytoplasmic translation initiation complex	FALSE
0.023500896	608	159	19	0.119496855	0.03125	GO:0043207	GO:BP	response to external biotic stimulus	FALSE
0.023500896	3470	159	67	0.421383648	0.019308357	GO:0050896	GO:BP	response to stimulus	FALSE
0.023500896	2247	159	48	0.301886792	0.021361816	GO:0048856	GO:BP	anatomical structure development	FALSE
0.023891579	422	159	15	0.094339623	0.035545024	GO:2000145	GO:BP	regulation of cell motility	FALSE
0.024149841	762	159	22	0.13836478	0.028871391	GO:0008283	GO:BP	cell population proliferation	TRUE
0.025195037	177	159	9	0.056603774	0.050847458	GO:0018193	GO:BP	peptidyl-amino acid modification	FALSE
0.02572051	1086	159	28	0.176100629	0.025782689	GO:0007166	GO:BP	cell surface receptor signaling pathway	TRUE
0.02646808	80	159	6	0.037735849	0.075	GO:0006413	GO:BP	translational initiation	FALSE
0.027520745	14	159	3	0.018867925	0.214285714	GO:0019730	GO:BP	antimicrobial humoral response	FALSE
0.028681394	987	159	26	0.163522013	0.026342452	GO:0006357	GO:BP	regulation of transcription by RNA polymerase II	FALSE
0.029553327	32	159	4	0.025157233	0.125	GO:0050830	GO:BP	defense response to Gram-positive bacterium	TRUE
0.030604723	782	159	22	0.13836478	0.028132992	GO:0008219	GO:BP	cell death	FALSE
0.030604723	782	159	22	0.13836478	0.028132992	GO:0012501	GO:BP	programmed cell death	FALSE
0.030604723	83	159	6	0.037735849	0.072289157	GO:0001659	GO:BP	temperature homeostasis	TRUE
0.030749545	56	159	5	0.031446541	0.089285714	GO:0030856	GO:BP	regulation of epithelial cell differentiation	TRUE
0.030828196	1051	159	27	0.169811321	0.025689819	GO:0006366	GO:BP	transcription by RNA polymerase II	FALSE
0.031127283	15	159	3	0.018867925	0.2	GO:0035767	GO:BP	endothelial cell chemotaxis	FALSE
0.031855237	150	159	8	0.050314465	0.053333333	GO:0014706	GO:BP	striated muscle tissue development	FALSE
0.033725244	2304	159	48	0.301886792	0.020833333	GO:0080090	GO:BP	regulation of primary metabolic process	TRUE
0.033978184	152	159	8	0.050314465	0.052631579	GO:0050900	GO:BP	leukocyte migration	FALSE
0.034572471	1696	159	38	0.238993711	0.02240566	GO:0006950	GO:BP	response to stress	FALSE
0.035628981	16	159	3	0.018867925	0.1875	GO:0021545	GO:BP	cranial nerve development	TRUE
0.035628981	119	159	7	0.044025157	0.058823529	GO:0032496	GO:BP	response to lipopolysaccharide	FALSE

0.036012973	403	159	14	0.088050314	0.034739454	GO:0046649	GO:BP	lymphocyte activation	FALSE
0.036277344	315	159	12	0.075471698	0.038095238	GO:0002521	GO:BP	leukocyte differentiation	FALSE
0.036277344	546	159	17	0.106918239	0.031135531	GO:1901700	GO:BP	response to oxygen-containing compound	FALSE
0.037418955	156	159	8	0.050314465	0.051282051	GO:0060537	GO:BP	muscle tissue development	FALSE
0.040772443	17	159	3	0.018867925	0.176470588	GO:0008334	GO:BP	histone mRNA metabolic process	TRUE
0.041531639	91	159	6	0.037735849	0.065934066	GO:0030595	GO:BP	leukocyte chemotaxis	FALSE
0.044012195	462	159	15	0.094339623	0.032467532	GO:0048646	GO:BP	anatomical structure formation involved in morphogenesis	FALSE
0.044012195	608	159	18	0.113207547	0.029605263	GO:0043067	GO:BP	regulation of programmed cell death	FALSE
0.044012195	2409	159	49	0.308176101	0.02034039	GO:0032502	GO:BP	developmental process	FALSE
0.044213647	5	159	2	0.012578616	0.4	GO:1904872	GO:BP	regulation of telomerase RNA localization to Cajal body	TRUE
0.044213647	18	159	3	0.018867925	0.166666667	GO:0060135	GO:BP	maternal process involved in female pregnancy	FALSE
0.044213647	5	159	2	0.012578616	0.4	GO:0031290	GO:BP	retinal ganglion cell axon guidance	TRUE
0.044213647	5	159	2	0.012578616	0.4	GO:0043435	GO:BP	response to corticotropin-releasing hormone	FALSE
0.044213647	127	159	7	0.044025157	0.05511811	GO:0002237	GO:BP	response to molecule of bacterial origin	FALSE
0.044213647	5	159	2	0.012578616	0.4	GO:0071376	GO:BP	cellular response to corticotropin-releasing hormone stimulus	TRUE
0.044213647	5	159	2	0.012578616	0.4	GO:0071028	GO:BP	nuclear mRNA surveillance	TRUE
0.044213647	1037	159	26	0.163522013	0.025072324	GO:0009653	GO:BP	anatomical structure morphogenesis	FALSE
0.044213647	93	159	6	0.037735849	0.064516129	GO:0097529	GO:BP	myeloid leukocyte migration	FALSE
0.046740328	1156	159	28	0.176100629	0.024221453	GO:0010605	GO:BP	negative regulation of macromolecule metabolic process	FALSE
0.04850376	2241	159	46	0.289308176	0.020526551	GO:0009889	GO:BP	regulation of biosynthetic process	FALSE
0.04850376	66	159	5	0.031446541	0.075757576	GO:0044403	GO:BP	biological process involved in symbiotic interaction	FALSE
0.04850376	1333	159	31	0.194968553	0.023255814	GO:0009966	GO:BP	regulation of signal transduction	TRUE
1.55E-14	71	159	17	0.106918239	0.23943662	GO:0022626	GO:CC	cytosolic ribosome	TRUE
2.34E-14	104	159	19	0.119496855	0.182692308	GO:0044391	GO:CC	ribosomal subunit	FALSE
6.07E-13	144	159	20	0.125786164	0.138888889	GO:0005840	GO:CC	ribosome	FALSE
6.02E-09	28	159	9	0.056603774	0.321428571	GO:0022625	GO:CC	cytosolic large ribosomal subunit	FALSE
6.58E-07	62	159	10	0.062893082	0.161290323	GO:0015934	GO:CC	large ribosomal subunit	FALSE
3.01E-06	27	159	7	0.044025157	0.259259259	GO:0022627	GO:CC	cytosolic small ribosomal subunit	FALSE
3.81E-06	42	159	8	0.050314465	0.19047619	GO:0015935	GO:CC	small ribosomal subunit	FALSE
0.006736436	2	159	2	0.012578616	1	GO:1990622	GO:CC	CHOP-ATF3 complex	TRUE
0.006736436	39	159	5	0.031446541	0.128205128	GO:0032040	GO:CC	small-subunit processome	TRUE
0.016882852	325	159	13	0.081761006	0.04	GO:0005615	GO:CC	extracellular space	TRUE
0.025400144	15	159	3	0.018867925	0.2	GO:0005852	GO:CC	eukaryotic translation initiation factor 3 complex	TRUE
0.025400144	56	159	5	0.031446541	0.089285714	GO:0030684	GO:CC	preribosome	FALSE

0.025400144	15	159	3	0.018867925	0.2	GO:0033290	GO:CC	eukaryotic 48S preinitiation complex	TRUE
0.02795852	16	159	3	0.018867925	0.1875	GO:0016282	GO:CC	eukaryotic 43S preinitiation complex	FALSE
0.02795852	2131	159	45	0.283018868	0.021116847	GO:0005829	GO:CC	cytosol	FALSE
0.030229813	17	159	3	0.018867925	0.176470588	GO:0070993	GO:CC	cytosolic translation preinitiation complex	FALSE
0.042544638	476	159	15	0.094339623	0.031512605	GO:0005576	GO:CC	extracellular region	FALSE
0.048730378	6	159	2	0.012578616	0.333333333	GO:0044194	GO:CC	cytolytic granule	TRUE
0.04963884	539	159	16	0.100628931	0.029684601	GO:0045202	GO:CC	synapse	TRUE
6.32E-16	96	159	20	0.125786164	0.208333333	GO:0003735	GO:MF	structural constituent of ribosome	TRUE
2.49E-09	214	159	20	0.125786164	0.093457944	GO:0005198	GO:MF	structural molecule activity	FALSE
8.69E-05	37	159	7	0.044025157	0.189189189	GO:0005125	GO:MF	cytokine activity	TRUE
0.000299827	165	159	12	0.075471698	0.072727273	GO:0001228	GO:MF	DNA-binding transcription activator activity, RNA polymerase II-specific	TRUE
0.000327623	170	159	12	0.075471698	0.070588235	GO:0001216	GO:MF	DNA-binding transcription activator activity	FALSE
0.003458642	95	159	8	0.050314465	0.084210526	GO:0005126	GO:MF	cytokine receptor binding	FALSE
0.004803268	7	159	3	0.018867925	0.428571429	GO:0017017	GO:MF	MAP kinase tyrosine/serine/threonine phosphatase activity	TRUE
0.004803268	7	159	3	0.018867925	0.428571429	GO:0033549	GO:MF	MAP kinase phosphatase activity	FALSE
0.010042224	9	159	3	0.018867925	0.333333333	GO:0008009	GO:MF	chemokine activity	FALSE
0.011429118	664	159	21	0.132075472	0.031626506	GO:0140110	GO:MF	transcription regulator activity	FALSE
0.011429118	92	159	7	0.044025157	0.076086957	GO:0048018	GO:MF	receptor ligand activity	FALSE
0.012409846	361	159	14	0.088050314	0.038781163	GO:0000981	GO:MF	DNA-binding transcription factor activity, RNA polymerase II-specific	FALSE
0.012409846	98	159	7	0.044025157	0.071428571	GO:0030545	GO:MF	signaling receptor regulator activity	FALSE
0.012409846	544	159	18	0.113207547	0.033088235	GO:0003690	GO:MF	double-stranded DNA binding	FALSE
0.012409846	398	159	15	0.094339623	0.037688442	GO:0003700	GO:MF	DNA-binding transcription factor activity	FALSE
0.012409846	498	159	17	0.106918239	0.034136546	GO:1990837	GO:MF	sequence-specific double-stranded DNA binding	TRUE
0.012409846	96	159	7	0.044025157	0.072916667	GO:0030546	GO:MF	signaling receptor activator activity	FALSE
0.015506016	29	159	4	0.025157233	0.137931034	GO:0008083	GO:MF	growth factor activity	FALSE
0.015556327	13	159	3	0.018867925	0.230769231	GO:0008138	GO:MF	protein tyrosine/serine/threonine phosphatase activity	FALSE
0.018620798	14	159	3	0.018867925	0.214285714	GO:0035035	GO:MF	histone acetyltransferase binding	TRUE
0.019944512	4	159	2	0.012578616	0.5	GO:0033550	GO:MF	MAP kinase tyrosine phosphatase activity	FALSE
0.019944512	395	159	14	0.088050314	0.035443038	GO:0038023	GO:MF	signaling receptor activity	FALSE
0.019944512	395	159	14	0.088050314	0.035443038	GO:0060089	GO:MF	molecular transducer activity	FALSE
0.019944512	15	159	3	0.018867925	0.2	GO:0042379	GO:MF	chemokine receptor binding	FALSE
0.019944512	4	159	2	0.012578616	0.5	GO:0008330	GO:MF	protein tyrosine/threonine phosphatase activity	TRUE
0.025263449	61	159	5	0.031446541	0.081967213	GO:0180051	GO:MF	translation factor activity	TRUE

0.029412595	64	159	5	0.031446541	0.078125	GO:0001221	GO:MF	transcription coregulator binding	TRUE
0.029412595	5	159	2	0.012578616	0.4	GO:0070180	GO:MF	large ribosomal subunit rRNA binding	TRUE
0.029867145	567	159	17	0.106918239	0.029982363	GO:0043565	GO:MF	sequence-specific DNA binding	FALSE
0.029867145	828	159	22	0.13836478	0.026570048	GO:0098772	GO:MF	molecular function regulator activity	FALSE
0.029867145	19	159	3	0.018867925	0.157894737	GO:0033293	GO:MF	monocarboxylic acid binding	TRUE
0.029867145	40	159	4	0.025157233	0.1	GO:0003743	GO:MF	translation initiation factor activity	FALSE
0.042882674	350	159	12	0.075471698	0.034285714	GO:0000987	GO:MF	cis-regulatory region sequence-specific DNA binding	TRUE
7.91E-20	89	159	22	0.13836478	0.247191011	KEGG:03010	KEGG	Ribosome	FALSE
1.40E-15	140	159	22	0.13836478	0.157142857	KEGG:05171	KEGG	Coronavirus disease - COVID-19	FALSE
9.97E-10	4554	159	104	0.65408805	0.022837066	KEGG:00000	KEGG	KEGG root term	FALSE
0.000300841	52	159	7	0.044025157	0.134615385	KEGG:05323	KEGG	Rheumatoid arthritis	FALSE
0.002717169	101	159	8	0.050314465	0.079207921	KEGG:04060	KEGG	Cytokine-cytokine receptor interaction	FALSE
0.005074456	85	159	7	0.044025157	0.082352941	KEGG:04668	KEGG	TNF signaling pathway	FALSE
0.021821502	31	159	4	0.025157233	0.129032258	KEGG:05219	KEGG	Bladder cancer	FALSE
0.030142429	122	159	7	0.044025157	0.057377049	KEGG:05202	KEGG	Transcriptional misregulation in cancer	FALSE
0.030142429	36	159	4	0.025157233	0.111111111	KEGG:04061	KEGG	Viral protein interaction with cytokine and cytokine receptor	FALSE
0.04361649	69	159	5	0.031446541	0.072463768	KEGG:05142	KEGG	Chagas disease	FALSE
0.04361649	211	159	9	0.056603774	0.042654028	KEGG:04151	KEGG	PI3K-Akt signaling pathway	FALSE

Pink Module GO Enrichment

p value	*term size*	*query size*	*overlap size*	*precision*	*recall*	*term id*	*source*	*term name*	*highlighted*
7.74E-05	363	212	24	0.113207547	0.066115702	GO:0001816	GO:BP	cytokine production	TRUE
7.74E-05	359	212	24	0.113207547	0.066852368	GO:0001817	GO:BP	regulation of cytokine production	FALSE
0.000254153	371	212	23	0.108490566	0.061994609	GO:0050778	GO:BP	positive regulation of immune response	FALSE
0.000254153	236	212	18	0.08490566	0.076271186	GO:0001819	GO:BP	positive regulation of cytokine production	FALSE
0.000319525	19	212	6	0.028301887	0.315789474	GO:0032620	GO:BP	interleukin-17 production	FALSE
0.000319525	483	212	26	0.122641509	0.053830228	GO:0032101	GO:BP	regulation of response to external stimulus	FALSE
0.000319525	19	212	6	0.028301887	0.315789474	GO:0032660	GO:BP	regulation of interleukin-17 production	FALSE
0.000319525	576	212	29	0.136792453	0.050347222	GO:0006629	GO:BP	lipid metabolic process	TRUE
0.000373852	727	212	33	0.155660377	0.045392022	GO:0002682	GO:BP	regulation of immune system process	FALSE
0.000390824	372	212	22	0.103773585	0.059139785	GO:0031347	GO:BP	regulation of defense response	FALSE
0.000512992	1736	212	58	0.273584906	0.033410138	GO:0048583	GO:BP	regulation of response to stimulus	FALSE
0.000512992	451	212	24	0.113207547	0.053215078	GO:0050776	GO:BP	regulation of immune response	FALSE
0.000512992	1170	212	44	0.20754717	0.037606838	GO:0051239	GO:BP	regulation of multicellular organismal process	FALSE

0.000512992	687	212	31	0.146226415	0.045123726	GO:0006796	GO:BP	phosphate-containing compound metabolic process	FALSE
0.000512992	688	212	31	0.146226415	0.04505814	GO:0006793	GO:BP	phosphorus metabolic process	FALSE
0.000512992	384	212	22	0.103773585	0.057291667	GO:1901698	GO:BP	response to nitrogen compound	FALSE
0.000512992	653	212	30	0.141509434	0.045941807	GO:0051240	GO:BP	positive regulation of multicellular organismal process	FALSE
0.000649861	526	212	26	0.122641509	0.049429658	GO:0002684	GO:BP	positive regulation of immune system process	FALSE
0.000683479	87	212	10	0.047169811	0.114942529	GO:0042742	GO:BP	defense response to bacterium	FALSE
0.000874697	606	212	28	0.132075472	0.04620462	GO:0042127	GO:BP	regulation of cell population proliferation	FALSE
0.001133864	8	212	4	0.018867925	0.5	GO:0032700	GO:BP	negative regulation of interleukin-17 production	FALSE
0.001367927	242	212	16	0.075471698	0.066115702	GO:0007169	GO:BP	cell surface receptor protein tyrosine kinase signaling pathway	FALSE
0.001858625	1333	212	46	0.216981132	0.034508627	GO:0009966	GO:BP	regulation of signal transduction	FALSE
0.001984777	4906	212	120	0.566037736	0.024459845	GO:0050789	GO:BP	regulation of biological process	FALSE
0.001984777	713	212	30	0.141509434	0.042075736	GO:0006955	GO:BP	immune response	FALSE
0.002039337	1470	212	49	0.231132075	0.033333333	GO:0010646	GO:BP	regulation of cell communication	FALSE
0.002073608	284	212	17	0.080188679	0.059859155	GO:0009725	GO:BP	response to hormone	FALSE
0.002073608	1474	212	49	0.231132075	0.033242877	GO:0023051	GO:BP	regulation of signaling	FALSE
0.002242678	762	212	31	0.146226415	0.040682415	GO:0008283	GO:BP	cell population proliferation	FALSE
0.002242678	689	212	29	0.136792453	0.042089985	GO:0006952	GO:BP	defense response	FALSE
0.002242678	546	212	25	0.117924528	0.045787546	GO:1901700	GO:BP	response to oxygen-containing compound	FALSE
0.00261242	484	212	23	0.108490566	0.047520661	GO:0019637	GO:BP	organophosphate metabolic process	FALSE
0.002798134	774	212	31	0.146226415	0.04005168	GO:0048585	GO:BP	negative regulation of response to stimulus	FALSE
0.002892559	1010	212	37	0.174528302	0.036633663	GO:0048584	GO:BP	positive regulation of response to stimulus	FALSE
0.002892559	1294	212	44	0.20754717	0.034003091	GO:0042221	GO:BP	response to chemical	FALSE
0.003168334	300	212	17	0.080188679	0.056666667	GO:0032103	GO:BP	positive regulation of response to external stimulus	FALSE
0.003168334	241	212	15	0.070754717	0.062240664	GO:1901699	GO:BP	cellular response to nitrogen compound	FALSE
0.003236477	242	212	15	0.070754717	0.061983471	GO:0031349	GO:BP	positive regulation of defense response	FALSE
0.003333965	5049	212	121	0.570754717	0.023965142	GO:0065007	GO:BP	biological regulation	FALSE
0.003549023	2241	212	65	0.306603774	0.029004909	GO:0009889	GO:BP	regulation of biosynthetic process	FALSE
0.003721706	218	212	14	0.066037736	0.064220183	GO:0009617	GO:BP	response to bacterium	FALSE
0.003976287	4750	212	115	0.54245283	0.024210526	GO:0050794	GO:BP	regulation of cellular process	FALSE
0.004346852	653	212	27	0.127358491	0.041347626	GO:0080134	GO:BP	regulation of response to stress	FALSE
0.004868134	254	212	15	0.070754717	0.059055118	GO:0002757	GO:BP	immune response-activating signaling pathway	FALSE

0.005029845	13	212	4	0.018867925	0.307692308	GO:1903426	GO:BP	regulation of reactive oxygen species biosynthetic process	FALSE
0.005287172	2801	212	76	0.358490566	0.027133167	GO:0019222	GO:BP	regulation of metabolic process	FALSE
0.005860195	123	212	10	0.047169811	0.081300813	GO:0070663	GO:BP	regulation of leukocyte proliferation	FALSE
0.006066447	261	212	15	0.070754717	0.057471264	GO:0002764	GO:BP	immune response-regulating signaling pathway	FALSE
0.006295772	637	212	26	0.122641509	0.040816327	GO:0009607	GO:BP	response to biotic stimulus	FALSE
0.006295772	423	212	20	0.094339623	0.047281324	GO:1901701	GO:BP	cellular response to oxygen-containing compound	FALSE
0.006295772	2347	212	66	0.311320755	0.028121006	GO:0048519	GO:BP	negative regulation of biological process	FALSE
0.006365059	151	212	11	0.051886792	0.072847682	GO:0050727	GO:BP	regulation of inflammatory response	FALSE
0.006409878	910	212	33	0.155660377	0.036263736	GO:0009605	GO:BP	response to external stimulus	FALSE
0.006409878	3470	212	89	0.419811321	0.025648415	GO:0050896	GO:BP	response to stimulus	FALSE
0.006409878	2590	212	71	0.33490566	0.027413127	GO:0048522	GO:BP	positive regulation of cellular process	FALSE
0.006813584	723	212	28	0.132075472	0.038727524	GO:0044281	GO:BP	small molecule metabolic process	TRUE
0.006813584	331	212	17	0.080188679	0.051359517	GO:0008284	GO:BP	positive regulation of cell population proliferation	FALSE
0.006813584	720	212	28	0.132075472	0.038888889	GO:0010647	GO:BP	positive regulation of cell communication	FALSE
0.006813584	725	212	28	0.132075472	0.03862069	GO:0042592	GO:BP	homeostatic process	FALSE
0.006813584	2466	212	68	0.320754717	0.02757502	GO:0023052	GO:BP	signaling	FALSE
0.006813584	501	212	22	0.103773585	0.043912176	GO:0009719	GO:BP	response to endogenous stimulus	FALSE
0.006813584	15	212	4	0.018867925	0.266666667	GO:1903409	GO:BP	reactive oxygen species biosynthetic process	FALSE
0.006813584	2314	212	65	0.306603774	0.028089888	GO:0007165	GO:BP	signal transduction	FALSE
0.006813584	130	212	10	0.047169811	0.076923077	GO:0001818	GO:BP	negative regulation of cytokine production	FALSE
0.006813584	156	212	11	0.051886792	0.070512821	GO:0002366	GO:BP	leukocyte activation involved in immune response	FALSE
0.006960967	727	212	28	0.132075472	0.038514443	GO:0023056	GO:BP	positive regulation of signaling	FALSE
0.006968574	2284	212	64	0.301886792	0.028021016	GO:0048523	GO:BP	negative regulation of cellular process	FALSE
0.006968574	304	212	16	0.075471698	0.052631579	GO:0006954	GO:BP	inflammatory response	FALSE
0.006968574	157	212	11	0.051886792	0.070063694	GO:0002263	GO:BP	cell activation involved in immune response	FALSE
0.007173318	2476	212	68	0.320754717	0.027463651	GO:0007154	GO:BP	cell communication	FALSE
0.007201446	1605	212	49	0.231132075	0.030529595	GO:0009893	GO:BP	positive regulation of metabolic process	FALSE
0.007664942	16	212	4	0.018867925	0.25	GO:0009595	GO:BP	detection of biotic stimulus	FALSE
0.007705727	477	212	21	0.099056604	0.044025157	GO:0098542	GO:BP	defense response to other organism	FALSE
0.007899181	2729	212	73	0.344339623	0.026749725	GO:0048518	GO:BP	positive regulation of biological process	FALSE
0.007899181	278	212	15	0.070754717	0.053956835	GO:0002253	GO:BP	activation of immune response	FALSE
0.00798716	667	212	26	0.122641509	0.03898051	GO:0009967	GO:BP	positive regulation of signal transduction	FALSE

0.00798716	1102	212	37	0.174528302	0.033575318	GO:0002376	GO:BP	immune system process	FALSE
0.00798716	897	212	32	0.150943396	0.03567447	GO:1902531	GO:BP	regulation of intracellular signal transduction	FALSE
0.00798716	7	212	3	0.014150943	0.428571429	GO:1901741	GO:BP	positive regulation of myoblast fusion	FALSE
0.00798716	7	212	3	0.014150943	0.428571429	GO:1901739	GO:BP	regulation of myoblast fusion	TRUE
0.00798716	111	212	9	0.04245283	0.081081081	GO:0002699	GO:BP	positive regulation of immune effector process	FALSE
0.00798716	88	212	8	0.037735849	0.090909091	GO:0072593	GO:BP	reactive oxygen species metabolic process	FALSE
0.00798716	163	212	11	0.051886792	0.067484663	GO:0070661	GO:BP	leukocyte proliferation	FALSE
0.00798716	818	212	30	0.141509434	0.036674817	GO:0141124	GO:BP	intracellular signaling cassette	FALSE
0.008369599	17	212	4	0.018867925	0.235294118	GO:0002828	GO:BP	regulation of type 2 immune response	FALSE
0.008549995	1195	212	39	0.183962264	0.032635983	GO:0009891	GO:BP	positive regulation of biosynthetic process	FALSE
0.008549995	48	212	6	0.028301887	0.125	GO:0014902	GO:BP	myotube differentiation	FALSE
0.008881219	114	212	9	0.04245283	0.078947368	GO:0050670	GO:BP	regulation of lymphocyte proliferation	FALSE
0.00976502	32	212	5	0.023584906	0.15625	GO:0051403	GO:BP	stress-activated MAPK cascade	FALSE
0.009898424	116	212	9	0.04245283	0.077586207	GO:0032944	GO:BP	regulation of mononuclear cell proliferation	FALSE
0.010448245	291	212	15	0.070754717	0.051546392	GO:0000165	GO:BP	MAPK cascade	FALSE
0.010448245	143	212	10	0.047169811	0.06993007	GO:0043434	GO:BP	response to peptide hormone	FALSE
0.010586983	608	212	24	0.113207547	0.039473684	GO:0051707	GO:BP	response to other organism	FALSE
0.010586983	608	212	24	0.113207547	0.039473684	GO:0043207	GO:BP	response to external biotic stimulus	FALSE
0.010670866	1696	212	50	0.235849057	0.029481132	GO:0006950	GO:BP	response to stress	FALSE
0.010773889	293	212	15	0.070754717	0.051194539	GO:0050865	GO:BP	regulation of cell activation	FALSE
0.010808245	8	212	3	0.014150943	0.375	GO:0070427	GO:BP	nucleotide-binding oligomerization domain containing 1 signaling pathway	FALSE
0.010883291	119	212	9	0.04245283	0.075630252	GO:0046434	GO:BP	organophosphate catabolic process	FALSE
0.010883291	688	212	26	0.122641509	0.037790698	GO:0044419	GO:BP	biological process involved in interspecies interaction between organisms	FALSE
0.010883291	119	212	9	0.04245283	0.075630252	GO:1901136	GO:BP	carbohydrate derivative catabolic process	TRUE
0.01097699	2	212	2	0.009433962	1	GO:0071641	GO:BP	negative regulation of macrophage inflammatory protein 1 alpha production	FALSE
0.011580409	96	212	8	0.037735849	0.083333333	GO:0010563	GO:BP	negative regulation of phosphorus metabolic process	FALSE
0.011580409	96	212	8	0.037735849	0.083333333	GO:0045936	GO:BP	negative regulation of phosphate metabolic process	FALSE
0.011580409	147	212	10	0.047169811	0.068027211	GO:0046651	GO:BP	lymphocyte proliferation	FALSE
0.012277733	74	212	7	0.033018868	0.094594595	GO:0001933	GO:BP	negative regulation of protein phosphorylation	FALSE
0.012277733	74	212	7	0.033018868	0.094594595	GO:0042326	GO:BP	negative regulation of phosphorylation	FALSE

0.01252508	35	212	5	0.023584906	0.142857143	GO:0031098	GO:BP	stress-activated protein kinase signaling cascade	FALSE
0.012718477	2949	212	76	0.358490566	0.025771448	GO:0051716	GO:BP	cellular response to stimulus	FALSE
0.012866108	150	212	10	0.047169811	0.066666667	GO:0007249	GO:BP	canonical NF-kappaB signal transduction	FALSE
0.01294617	369	212	17	0.080188679	0.046070461	GO:0098657	GO:BP	import into cell	TRUE
0.013251662	151	212	10	0.047169811	0.066225166	GO:0032943	GO:BP	mononuclear cell proliferation	FALSE
0.013251662	99	212	8	0.037735849	0.080808081	GO:0002822	GO:BP	regulation of adaptive immune response based on somatic recombination of immune receptors built from immunoglobulin superfamily domains	FALSE
0.013434111	477	212	20	0.094339623	0.041928721	GO:1902533	GO:BP	positive regulation of intracellular signal transduction	FALSE
0.013626561	272	212	14	0.066037736	0.051470588	GO:0002831	GO:BP	regulation of response to biotic stimulus	FALSE
0.014226475	211	212	12	0.056603774	0.056872038	GO:0002833	GO:BP	positive regulation of response to biotic stimulus	FALSE
0.014407665	274	212	14	0.066037736	0.051094891	GO:0002694	GO:BP	regulation of leukocyte activation	FALSE
0.014439991	21	212	4	0.018867925	0.19047619	GO:0036230	GO:BP	granulocyte activation	TRUE
0.014551123	410	212	18	0.08490566	0.043902439	GO:0051241	GO:BP	negative regulation of multicellular organismal process	FALSE
0.014649465	127	212	9	0.04245283	0.070866142	GO:0002237	GO:BP	response to molecule of bacterial origin	FALSE
0.014973633	78	212	7	0.033018868	0.08974359	GO:0070665	GO:BP	positive regulation of leukocyte proliferation	FALSE
0.015256405	128	212	9	0.04245283	0.0703125	GO:0015980	GO:BP	energy derivation by oxidation of organic compounds	TRUE
0.015735464	57	212	6	0.028301887	0.105263158	GO:2000377	GO:BP	regulation of reactive oxygen species metabolic process	FALSE
0.016379967	22	212	4	0.018867925	0.181818182	GO:0042092	GO:BP	type 2 immune response	FALSE
0.016379967	1086	212	35	0.16509434	0.032228361	GO:0007166	GO:BP	cell surface receptor signaling pathway	FALSE
0.016379967	22	212	4	0.018867925	0.181818182	GO:0032872	GO:BP	regulation of stress-activated MAPK cascade	FALSE
0.016498527	80	212	7	0.033018868	0.0875	GO:0032640	GO:BP	tumor necrosis factor production	FALSE
0.016498527	80	212	7	0.033018868	0.0875	GO:0032680	GO:BP	regulation of tumor necrosis factor production	FALSE
0.016755285	2409	212	64	0.301886792	0.02656704	GO:0032502	GO:BP	developmental process	TRUE
0.016755285	81	212	7	0.033018868	0.086419753	GO:1903555	GO:BP	regulation of tumor necrosis factor superfamily cytokine production	FALSE
0.016755285	219	212	12	0.056603774	0.054794521	GO:0022407	GO:BP	regulation of cell-cell adhesion	TRUE
0.016755285	420	212	18	0.08490566	0.042857143	GO:1901135	GO:BP	carbohydrate derivative metabolic process	FALSE
0.016755285	10	212	3	0.014150943	0.3	GO:0060142	GO:BP	regulation of syncytium formation by plasma membrane fusion	FALSE

0.016755285	10	212	3	0.014150943	0.3	GO:0060143	GO:BP	positive regulation of syncytium formation by plasma membrane fusion	FALSE
0.016755285	250	212	13	0.061320755	0.052	GO:0051249	GO:BP	regulation of lymphocyte activation	FALSE
0.016755285	81	212	7	0.033018868	0.086419753	GO:0071706	GO:BP	tumor necrosis factor superfamily cytokine production	FALSE
0.016755285	10	212	3	0.014150943	0.3	GO:0002829	GO:BP	negative regulation of type 2 immune response	FALSE
0.016758949	59	212	6	0.028301887	0.101694915	GO:0050728	GO:BP	negative regulation of inflammatory response	FALSE
0.016758949	189	212	11	0.051886792	0.058201058	GO:0006644	GO:BP	phospholipid metabolic process	FALSE
0.017125827	23	212	4	0.018867925	0.173913043	GO:0070302	GO:BP	regulation of stress-activated protein kinase signaling cascade	FALSE
0.017125827	82	212	7	0.033018868	0.085365854	GO:0071222	GO:BP	cellular response to lipopolysaccharide	FALSE
0.017125827	2179	212	59	0.278301887	0.027076641	GO:0010556	GO:BP	regulation of macromolecule biosynthetic process	FALSE
0.017125827	608	212	23	0.108490566	0.037828947	GO:0043067	GO:BP	regulation of programmed cell death	FALSE
0.017333418	107	212	8	0.037735849	0.074766355	GO:0002440	GO:BP	production of molecular mediator of immune response	FALSE
0.017333418	107	212	8	0.037735849	0.074766355	GO:0071216	GO:BP	cellular response to biotic stimulus	FALSE
0.018305112	108	212	8	0.037735849	0.074074074	GO:0002819	GO:BP	regulation of adaptive immune response	FALSE
0.018431193	811	212	28	0.132075472	0.034525277	GO:0070887	GO:BP	cellular response to chemical stimulus	FALSE
0.019513104	194	212	11	0.051886792	0.056701031	GO:0006091	GO:BP	generation of precursor metabolites and energy	FALSE
0.019665632	578	212	22	0.103773585	0.038062284	GO:0042981	GO:BP	regulation of apoptotic process	FALSE
0.019908219	11	212	3	0.014150943	0.272727273	GO:0098581	GO:BP	detection of external biotic stimulus	FALSE
0.021112596	42	212	5	0.023584906	0.119047619	GO:0002532	GO:BP	production of molecular mediator involved in inflammatory response	FALSE
0.021199735	86	212	7	0.033018868	0.081395349	GO:0071219	GO:BP	cellular response to molecule of bacterial origin	FALSE
0.021199735	3	212	2	0.009433962	0.666666667	GO:0070426	GO:BP	positive regulation of nucleotide-binding domain, leucine rich repeat containing receptor signaling pathway	FALSE
0.021199735	3	212	2	0.009433962	0.666666667	GO:2000049	GO:BP	positive regulation of cell-cell adhesion mediated by cadherin	TRUE
0.021199735	3	212	2	0.009433962	0.666666667	GO:0070345	GO:BP	negative regulation of fat cell proliferation	TRUE
0.021199735	3	212	2	0.009433962	0.666666667	GO:0070430	GO:BP	positive regulation of nucleotide-binding oligomerization domain containing 1 signaling pathway	FALSE

0.021345209	782	212	27	0.127358491	0.034526854	GO:0012501	GO:BP	programmed cell death	FALSE
0.021345209	782	212	27	0.127358491	0.034526854	GO:0008219	GO:BP	cell death	FALSE
0.021783377	744	212	26	0.122641509	0.034946237	GO:0006915	GO:BP	apoptotic process	FALSE
0.021783377	87	212	7	0.033018868	0.08045977	GO:0045333	GO:BP	cellular respiration	FALSE
0.021783377	1162	212	36	0.169811321	0.030981067	GO:0010557	GO:BP	positive regulation of macromolecule biosynthetic process	FALSE
0.021783377	1470	212	43	0.202830189	0.029251701	GO:0010604	GO:BP	positive regulation of macromolecule metabolic process	FALSE
0.021906809	169	212	10	0.047169811	0.059171598	GO:0002697	GO:BP	regulation of immune effector process	FALSE
0.021906809	113	212	8	0.037735849	0.07079646	GO:0032868	GO:BP	response to insulin	FALSE
0.022587376	88	212	7	0.033018868	0.079545455	GO:0042129	GO:BP	regulation of T cell proliferation	FALSE
0.022600153	628	212	23	0.108490566	0.036624204	GO:0009968	GO:BP	negative regulation of signal transduction	FALSE
0.023082294	201	212	11	0.051886792	0.054726368	GO:0045089	GO:BP	positive regulation of innate immune response	FALSE
0.023191191	65	212	6	0.028301887	0.092307692	GO:0002702	GO:BP	positive regulation of production of molecular mediator of immune response	FALSE
0.023389921	26	212	4	0.018867925	0.153846154	GO:0043331	GO:BP	response to dsRNA	FALSE
0.023404749	1707	212	48	0.226415094	0.028119508	GO:0051641	GO:BP	cellular localization	TRUE
0.023404749	89	212	7	0.033018868	0.078651685	GO:0002700	GO:BP	regulation of production of molecular mediator of immune response	FALSE
0.023506514	554	212	21	0.099056604	0.037906137	GO:0010628	GO:BP	positive regulation of gene expression	FALSE
0.023532363	234	212	12	0.056603774	0.051282051	GO:0045088	GO:BP	regulation of innate immune response	FALSE
0.023532363	2561	212	66	0.311320755	0.025771183	GO:0060255	GO:BP	regulation of macromolecule metabolic process	FALSE
0.023990944	301	212	14	0.066037736	0.046511628	GO:0008610	GO:BP	lipid biosynthetic process	FALSE
0.024087904	116	212	8	0.037735849	0.068965517	GO:0002285	GO:BP	lymphocyte activation involved in immune response	FALSE
0.024087904	173	212	10	0.047169811	0.057803468	GO:0002443	GO:BP	leukocyte mediated immunity	FALSE
0.024125701	2133	212	57	0.268867925	0.026722925	GO:0010468	GO:BP	regulation of gene expression	FALSE
0.024175927	45	212	5	0.023584906	0.111111111	GO:0032602	GO:BP	chemokine production	FALSE
0.024175927	45	212	5	0.023584906	0.111111111	GO:0032642	GO:BP	regulation of chemokine production	FALSE
0.024175927	45	212	5	0.023584906	0.111111111	GO:1904659	GO:BP	D-glucose transmembrane transport	TRUE
0.024501785	2472	212	64	0.301886792	0.025889968	GO:0051179	GO:BP	localization	FALSE
0.025254024	374	212	16	0.075471698	0.042780749	GO:0048871	GO:BP	multicellular organismal-level homeostasis	FALSE
0.025317254	447	212	18	0.08490566	0.040268456	GO:0071495	GO:BP	cellular response to endogenous stimulus	FALSE
0.025662762	146	212	9	0.04245283	0.061643836	GO:0002460	GO:BP	adaptive immune response based on somatic recombination of immune receptors built from immunoglobulin superfamily domains	FALSE

0.026480642	119	212	8	0.037735849	0.067226891	GO:0032496	GO:BP	response to lipopolysaccharide	FALSE
0.026480642	13	212	3	0.014150943	0.230769231	GO:0035872	GO:BP	nucleotide-binding domain, leucine rich repeat containing receptor signaling pathway	FALSE
0.026480642	119	212	8	0.037735849	0.067226891	GO:0071375	GO:BP	cellular response to peptide hormone stimulus	FALSE
0.026480642	13	212	3	0.014150943	0.230769231	GO:0060907	GO:BP	positive regulation of macrophage cytokine production	TRUE
0.026480642	377	212	16	0.075471698	0.042440318	GO:0007167	GO:BP	enzyme-linked receptor protein signaling pathway	FALSE
0.027321562	148	212	9	0.04245283	0.060810811	GO:0016042	GO:BP	lipid catabolic process	FALSE
0.027487339	69	212	6	0.028301887	0.086956522	GO:0050671	GO:BP	positive regulation of lymphocyte proliferation	FALSE
0.027487339	2247	212	59	0.278301887	0.026257232	GO:0048856	GO:BP	anatomical structure development	FALSE
0.027487339	47	212	5	0.023584906	0.106382979	GO:0008645	GO:BP	hexose transmembrane transport	FALSE
0.028088325	94	212	7	0.033018868	0.074468085	GO:0043123	GO:BP	positive regulation of canonical NF-kappaB signal transduction	FALSE
0.02826506	276	212	13	0.061320755	0.047101449	GO:0019220	GO:BP	regulation of phosphate metabolic process	FALSE
0.02826506	276	212	13	0.061320755	0.047101449	GO:0051174	GO:BP	regulation of phosphorus metabolic process	FALSE
0.028391468	243	212	12	0.056603774	0.049382716	GO:0043408	GO:BP	regulation of MAPK cascade	FALSE
0.028653694	70	212	6	0.028301887	0.085714286	GO:0032675	GO:BP	regulation of interleukin-6 production	FALSE
0.028653694	70	212	6	0.028301887	0.085714286	GO:0032635	GO:BP	interleukin-6 production	FALSE
0.028729471	48	212	5	0.023584906	0.104166667	GO:0032760	GO:BP	positive regulation of tumor necrosis factor production	FALSE
0.028729471	71	212	6	0.028301887	0.084507042	GO:0032946	GO:BP	positive regulation of mononuclear cell proliferation	FALSE
0.028729471	4	212	2	0.009433962	0.5	GO:0071640	GO:BP	regulation of macrophage inflammatory protein 1 alpha production	FALSE
0.028729471	29	212	4	0.018867925	0.137931034	GO:0031663	GO:BP	lipopolysaccharide-mediated signaling pathway	FALSE
0.028729471	280	212	13	0.061320755	0.046428571	GO:0002252	GO:BP	immune effector process	FALSE
0.028729471	4	212	2	0.009433962	0.5	GO:0097350	GO:BP	neutrophil clearance	TRUE
0.028729471	4	212	2	0.009433962	0.5	GO:0032497	GO:BP	detection of lipopolysaccharide	FALSE
0.028729471	4	212	2	0.009433962	0.5	GO:0070428	GO:BP	regulation of nucleotide-binding oligomerization domain containing 1 signaling pathway	FALSE
0.028729471	4	212	2	0.009433962	0.5	GO:0090400	GO:BP	stress-induced premature senescence	TRUE
0.028729471	14	212	3	0.014150943	0.214285714	GO:0070304	GO:BP	positive regulation of stress-activated protein kinase signaling cascade	FALSE
0.028729471	14	212	3	0.014150943	0.214285714	GO:0032874	GO:BP	positive regulation of stress-activated MAPK cascade	FALSE

0.028729471	4	212	2	0.009433962	0.5	GO:0032725	GO:BP	positive regulation of granulocyte macrophage colony-stimulating factor production	FALSE
0.028729471	29	212	4	0.018867925	0.137931034	GO:1900015	GO:BP	regulation of cytokine production involved in inflammatory response	FALSE
0.028729471	651	212	23	0.108490566	0.035330261	GO:0010648	GO:BP	negative regulation of cell communication	FALSE
0.028729471	4	212	2	0.009433962	0.5	GO:0043301	GO:BP	negative regulation of leukocyte degranulation	TRUE
0.028729471	49	212	5	0.023584906	0.102040816	GO:1903557	GO:BP	positive regulation of tumor necrosis factor superfamily cytokine production	FALSE
0.028729471	48	212	5	0.023584906	0.104166667	GO:0015749	GO:BP	monosaccharide transmembrane transport	FALSE
0.028729471	383	212	16	0.075471698	0.041775457	GO:0045087	GO:BP	innate immune response	FALSE
0.028729471	654	212	23	0.108490566	0.035168196	GO:0023057	GO:BP	negative regulation of signaling	FALSE
0.028729471	4	212	2	0.009433962	0.5	GO:1903977	GO:BP	positive regulation of glial cell migration	TRUE
0.028729471	29	212	4	0.018867925	0.137931034	GO:0002534	GO:BP	cytokine production involved in inflammatory response	FALSE
0.028729471	71	212	6	0.028301887	0.084507042	GO:0009060	GO:BP	aerobic respiration	FALSE
0.028729471	4	212	2	0.009433962	0.5	GO:0071608	GO:BP	macrophage inflammatory protein-1 alpha production	FALSE
0.028729471	151	212	9	0.04245283	0.059602649	GO:0002429	GO:BP	immune response-activating cell surface receptor signaling pathway	FALSE
0.028729471	4	212	2	0.009433962	0.5	GO:1900747	GO:BP	negative regulation of vascular endothelial growth factor signaling pathway	TRUE
0.028729471	1420	212	41	0.193396226	0.028873239	GO:0035556	GO:BP	intracellular signal transduction	FALSE
0.028729471	96	212	7	0.033018868	0.072916667	GO:0002685	GO:BP	regulation of leukocyte migration	FALSE
0.029045665	72	212	6	0.028301887	0.083333333	GO:1990845	GO:BP	adaptive thermogenesis	TRUE
0.029101383	248	212	12	0.056603774	0.048387097	GO:0005975	GO:BP	carbohydrate metabolic process	TRUE
0.029300698	352	212	15	0.070754717	0.042613636	GO:1902532	GO:BP	negative regulation of intracellular signal transduction	FALSE
0.029584963	30	212	4	0.018867925	0.133333333	GO:0035924	GO:BP	cellular response to vascular endothelial growth factor stimulus	FALSE
0.031309929	391	212	16	0.075471698	0.040920716	GO:0140546	GO:BP	defense response to symbiont	FALSE
0.032057299	2522	212	64	0.301886792	0.025376685	GO:0032501	GO:BP	multicellular organismal process	FALSE
0.03255897	74	212	6	0.028301887	0.081081081	GO:0006195	GO:BP	purine nucleotide catabolic process	TRUE
0.032566339	31	212	4	0.018867925	0.129032258	GO:0032655	GO:BP	regulation of interleukin-12 production	FALSE
0.032566339	15	212	3	0.014150943	0.2	GO:0032495	GO:BP	response to muramyl dipeptide	FALSE
0.032566339	31	212	4	0.018867925	0.129032258	GO:0032615	GO:BP	interleukin-12 production	FALSE

0.032566339	15	212	3	0.014150943	0.2	GO:0070861	GO:BP	regulation of protein exit from endoplasmic reticulum	TRUE
0.032566339	15	212	3	0.014150943	0.2	GO:0045920	GO:BP	negative regulation of exocytosis	TRUE
0.032582606	157	212	9	0.04245283	0.057324841	GO:0002768	GO:BP	immune response-regulating cell surface receptor signaling pathway	FALSE
0.032630982	253	212	12	0.056603774	0.04743083	GO:0008285	GO:BP	negative regulation of cell population proliferation	FALSE
0.033571186	254	212	12	0.056603774	0.047244094	GO:0070848	GO:BP	response to growth factor	FALSE
0.03565939	32	212	4	0.018867925	0.125	GO:0050830	GO:BP	defense response to Gram-positive bacterium	FALSE
0.03565939	102	212	7	0.033018868	0.068627451	GO:0002274	GO:BP	myeloid leukocyte activation	FALSE
0.03565939	32	212	4	0.018867925	0.125	GO:0045123	GO:BP	cellular extravasation	TRUE
0.036007346	191	212	10	0.047169811	0.052356021	GO:0046486	GO:BP	glycerolipid metabolic process	FALSE
0.036789249	53	212	5	0.023584906	0.094339623	GO:0071887	GO:BP	leukocyte apoptotic process	FALSE
0.036789249	53	212	5	0.023584906	0.094339623	GO:0050871	GO:BP	positive regulation of B cell activation	FALSE
0.036854396	131	212	8	0.037735849	0.061068702	GO:0031400	GO:BP	negative regulation of protein modification process	FALSE
0.036924007	103	212	7	0.033018868	0.067961165	GO:0050851	GO:BP	antigen receptor-mediated signaling pathway	FALSE
0.037498312	16	212	3	0.014150943	0.1875	GO:0019674	GO:BP	NAD metabolic process	FALSE
0.037498312	16	212	3	0.014150943	0.1875	GO:0007520	GO:BP	myoblast fusion	FALSE
0.038636447	33	212	4	0.018867925	0.121212121	GO:0046323	GO:BP	D-glucose import	FALSE
0.038851569	54	212	5	0.023584906	0.092592593	GO:0034219	GO:BP	carbohydrate transmembrane transport	FALSE
0.038851569	54	212	5	0.023584906	0.092592593	GO:0051606	GO:BP	detection of stimulus	FALSE
0.039364144	133	212	8	0.037735849	0.060150376	GO:0006066	GO:BP	alcohol metabolic process	FALSE
0.040742803	79	212	6	0.028301887	0.075949367	GO:0050864	GO:BP	regulation of B cell activation	FALSE
0.040742803	5	212	2	0.009433962	0.4	GO:1902548	GO:BP	negative regulation of cellular response to vascular endothelial growth factor stimulus	FALSE
0.040742803	79	212	6	0.028301887	0.075949367	GO:0071356	GO:BP	cellular response to tumor necrosis factor	TRUE
0.040742803	55	212	5	0.023584906	0.090909091	GO:0002367	GO:BP	cytokine production involved in immune response	FALSE
0.040742803	55	212	5	0.023584906	0.090909091	GO:0002718	GO:BP	regulation of cytokine production involved in immune response	FALSE
0.040742803	561	212	20	0.094339623	0.035650624	GO:0007155	GO:BP	cell adhesion	TRUE
0.040742803	5	212	2	0.009433962	0.4	GO:0032645	GO:BP	regulation of granulocyte macrophage colony-stimulating factor production	FALSE
0.040742803	5	212	2	0.009433962	0.4	GO:0032604	GO:BP	granulocyte macrophage colony-stimulating factor production	FALSE
0.041207978	165	212	9	0.04245283	0.054545455	GO:0002696	GO:BP	positive regulation of leukocyte activation	FALSE
0.041920087	17	212	3	0.014150943	0.176470588	GO:0002825	GO:BP	regulation of T-helper 1 type immune response	FALSE

0.041920087	851	212	27	0.127358491	0.03172738	GO:0071705	GO:BP	nitrogen compound transport	TRUE
0.041920087	17	212	3	0.014150943	0.176470588	GO:0006622	GO:BP	protein targeting to lysosome	TRUE
0.041920087	17	212	3	0.014150943	0.176470588	GO:0050829	GO:BP	defense response to Gram-negative bacterium	FALSE
0.041920087	17	212	3	0.014150943	0.176470588	GO:0043372	GO:BP	positive regulation of CD4-positive, alpha-beta T cell differentiation	TRUE
0.042165282	107	212	7	0.033018868	0.065420561	GO:0031348	GO:BP	negative regulation of defense response	FALSE
0.043938373	108	212	7	0.033018868	0.064814815	GO:0042098	GO:BP	T cell proliferation	FALSE
0.043938373	137	212	8	0.037735849	0.058394161	GO:0043122	GO:BP	regulation of canonical NF-kappaB signal transduction	FALSE
0.043938373	137	212	8	0.037735849	0.058394161	GO:0002449	GO:BP	lymphocyte mediated immunity	FALSE
0.043938373	81	212	6	0.028301887	0.074074074	GO:0009166	GO:BP	nucleotide catabolic process	FALSE
0.044415605	233	212	11	0.051886792	0.0472103	GO:0016310	GO:BP	phosphorylation	FALSE
0.045261338	201	212	10	0.047169811	0.049751244	GO:1903706	GO:BP	regulation of hemopoiesis	FALSE
0.045261338	82	212	6	0.028301887	0.073170732	GO:0046496	GO:BP	nicotinamide nucleotide metabolic process	FALSE
0.045261338	1252	212	36	0.169811321	0.028753994	GO:0008104	GO:BP	protein localization	FALSE
0.045261338	57	212	5	0.023584906	0.087719298	GO:0050868	GO:BP	negative regulation of T cell activation	TRUE
0.045261338	82	212	6	0.028301887	0.073170732	GO:0072523	GO:BP	purine-containing compound catabolic process	FALSE
0.045261338	82	212	6	0.028301887	0.073170732	GO:0019362	GO:BP	pyridine nucleotide metabolic process	FALSE
0.046204077	235	212	11	0.051886792	0.046808511	GO:0032870	GO:BP	cellular response to hormone stimulus	FALSE
0.047012218	18	212	3	0.014150943	0.166666667	GO:0042119	GO:BP	neutrophil activation	FALSE
0.047012218	18	212	3	0.014150943	0.166666667	GO:0061081	GO:BP	positive regulation of myeloid leukocyte cytokine production involved in immune response	FALSE
0.047129862	36	212	4	0.018867925	0.111111111	GO:0046635	GO:BP	positive regulation of alpha-beta T cell activation	TRUE
0.04714609	58	212	5	0.023584906	0.086206897	GO:0008643	GO:BP	carbohydrate transport	FALSE
0.047505154	171	212	9	0.04245283	0.052631579	GO:0050867	GO:BP	positive regulation of cell activation	FALSE
0.047505154	171	212	9	0.04245283	0.052631579	GO:0006163	GO:BP	purine nucleotide metabolic process	FALSE
0.047505154	171	212	9	0.04245283	0.052631579	GO:0002250	GO:BP	adaptive immune response	FALSE
0.047505154	1259	212	36	0.169811321	0.028594122	GO:0070727	GO:BP	cellular macromolecule localization	FALSE
0.001152919	3584	212	97	0.45754717	0.027064732	GO:0016020	GO:CC	membrane	TRUE
0.006289942	5580	212	131	0.617924528	0.023476703	GO:0005737	GO:CC	cytoplasm	TRUE
0.042828186	2	212	2	0.009433962	1	GO:0046696	GO:CC	lipopolysaccharide receptor complex	TRUE
0.015290761	3652	212	95	0.448113208	0.026013143	GO:0003824	GO:MF	catalytic activity	TRUE
0.046888469	619	212	25	0.117924528	0.040387722	GO:0060090	GO:MF	molecular adaptor activity	TRUE
0.002563445	47	212	7	0.033018868	0.14893617	KEGG:05140	KEGG	Leishmaniasis	FALSE
0.002563445	119	212	11	0.051886792	0.092436975	KEGG:04621	KEGG	NOD-like receptor signaling pathway	FALSE
0.003065176	112	212	10	0.047169811	0.089285714	KEGG:05135	KEGG	Yersinia infection	FALSE
0.010549271	188	212	12	0.056603774	0.063829787	KEGG:05132	KEGG	Salmonella infection	FALSE
0.014928627	52	212	6	0.028301887	0.115384615	KEGG:05133	KEGG	Pertussis	FALSE

0.014928627	96	212	8	0.037735849	0.083333333	KEGG:04613	KEGG	Neutrophil extracellular trap formation	FALSE
0.033764257	111	212	8	0.037735849	0.072072072	KEGG:05152	KEGG	Tuberculosis	FALSE
0.049200868	69	212	6	0.028301887	0.086956522	KEGG:05235	KEGG	PD-L1 expression and PD-1 checkpoint pathway in cancer	FALSE

Purple Module GO Enrichment

p value	*term size*	*query size*	*overlap size*	*precision*	*recall*	*term id*	*source*	*term name*	*highlighted*
0.010375746	724	133	23	0.172932331	0.031767956	GO:0006508	GO:BP	proteolysis	TRUE
0.011272481	566	133	19	0.142857143	0.033568905	GO:0030163	GO:BP	protein catabolic process	FALSE
0.011272481	455	133	17	0.127819549	0.037362637	GO:0051603	GO:BP	proteolysis involved in protein catabolic process	FALSE
0.019948134	1840	133	38	0.285714286	0.020652174	GO:0019538	GO:BP	protein metabolic process	FALSE
0.019948134	26	133	4	0.030075188	0.153846154	GO:0042773	GO:BP	ATP synthesis coupled electron transport	FALSE
0.019948134	71	133	6	0.045112782	0.084507042	GO:0009060	GO:BP	aerobic respiration	FALSE
0.019948134	776	133	22	0.165413534	0.028350515	GO:0009057	GO:BP	macromolecule catabolic process	FALSE
0.019948134	23	133	4	0.030075188	0.173913043	GO:0019646	GO:BP	aerobic electron transport chain	FALSE
0.019948134	26	133	4	0.030075188	0.153846154	GO:0006778	GO:BP	porphyrin-containing compound metabolic process	FALSE
0.019948134	24	133	4	0.030075188	0.166666667	GO:0042168	GO:BP	heme metabolic process	TRUE
0.019948134	45	133	5	0.037593985	0.111111111	GO:0006119	GO:BP	oxidative phosphorylation	TRUE
0.019948134	10	133	3	0.022556391	0.3	GO:0000028	GO:BP	ribosomal small subunit assembly	TRUE
0.019948134	26	133	4	0.030075188	0.153846154	GO:0042775	GO:BP	mitochondrial ATP synthesis coupled electron transport	FALSE
0.019948134	1301	133	30	0.22556391	0.023059185	GO:0009056	GO:BP	catabolic process	FALSE
0.019948134	352	133	13	0.097744361	0.036931818	GO:0006511	GO:BP	ubiquitin-dependent protein catabolic process	FALSE
0.022339343	361	133	13	0.097744361	0.03601108	GO:0019941	GO:BP	modification-dependent protein catabolic process	FALSE
0.022339343	362	133	13	0.097744361	0.035911602	GO:0043632	GO:BP	modification-dependent macromolecule catabolic process	FALSE
0.033471867	31	133	4	0.030075188	0.129032258	GO:0033013	GO:BP	tetrapyrrole metabolic process	FALSE
0.036657158	33	133	4	0.030075188	0.121212121	GO:0032981	GO:BP	mitochondrial respiratory chain complex I assembly	TRUE
0.036657158	33	133	4	0.030075188	0.121212121	GO:0010257	GO:BP	NADH dehydrogenase complex assembly	FALSE
0.036657158	87	133	6	0.045112782	0.068965517	GO:0045333	GO:BP	cellular respiration	FALSE
0.039307357	34	133	4	0.030075188	0.117647059	GO:0042255	GO:BP	ribosome assembly	FALSE
0.044964475	36	133	4	0.030075188	0.111111111	GO:0042440	GO:BP	pigment metabolic process	FALSE
0.044964475	36	133	4	0.030075188	0.111111111	GO:0022904	GO:BP	respiratory electron transport chain	FALSE
0.004523819	2131	133	44	0.330827068	0.020647583	GO:0005829	GO:CC	cytosol	TRUE
0.004523819	71	133	7	0.052631579	0.098591549	GO:0022626	GO:CC	cytosolic ribosome	FALSE
0.021544448	5580	133	84	0.631578947	0.015053763	GO:0005737	GO:CC	cytoplasm	FALSE
0.021544448	144	133	8	0.060150376	0.055555556	GO:0005840	GO:CC	ribosome	FALSE
0.028085807	57	133	5	0.037593985	0.087719298	GO:1905369	GO:CC	endopeptidase complex	TRUE
0.013902038	163	133	9	0.067669173	0.055214724	KEGG:05012	KEGG	Parkinson disease	FALSE
0.025657423	194	133	9	0.067669173	0.046391753	KEGG:05016	KEGG	Huntington disease	FALSE

0.041526742	66	133	5	0.037593985	0.075757576	KEGG:00190	KEGG	Oxidative phosphorylation	FALSE
0.041999343	909	133	21	0.157894737	0.02310231	KEGG:01100	KEGG	Metabolic pathways	FALSE
0.041999343	289	133	10	0.07518797	0.034602076	KEGG:05022	KEGG	Pathways of neurodegeneration - multiple diseases	FALSE
0.041999343	4554	133	69	0.518796992	0.015151515	KEGG:00000	KEGG	KEGG root term	FALSE
0.041999343	23	133	3	0.022556391	0.130434783	KEGG:00860	KEGG	Porphyrin metabolism	FALSE

Red Module GO Enrichment

p value	*term size*	*query size*	*overlap size*	*precision*	*recall*	*term id*	*source*	*term name*	*highlighted*
2.24E-09	3470	267	135	0.505617978	0.038904899	GO:0050896	GO:BP	response to stimulus	TRUE
4.64E-08	2949	267	117	0.438202247	0.039674466	GO:0051716	GO:BP	cellular response to stimulus	FALSE
4.36E-07	5049	267	166	0.621722846	0.032877798	GO:0065007	GO:BP	biological regulation	FALSE
4.46E-07	2466	267	100	0.374531835	0.0405515	GO:0023052	GO:BP	signaling	FALSE
4.46E-07	4906	267	162	0.606741573	0.033020791	GO:0050789	GO:BP	regulation of biological process	FALSE
4.63E-07	2476	267	100	0.374531835	0.040387722	GO:0007154	GO:BP	cell communication	FALSE
1.32E-06	2314	267	94	0.352059925	0.040622299	GO:0007165	GO:BP	signal transduction	FALSE
1.65E-06	4750	267	156	0.584269663	0.032842105	GO:0050794	GO:BP	regulation of cellular process	FALSE
3.26E-06	2729	267	104	0.389513109	0.038109198	GO:0048518	GO:BP	positive regulation of biological process	FALSE
3.85E-06	2522	267	98	0.367041199	0.038858049	GO:0032501	GO:BP	multicellular organismal process	FALSE
6.91E-06	2409	267	94	0.352059925	0.03902034	GO:0032502	GO:BP	developmental process	FALSE
1.31E-05	2590	267	98	0.367041199	0.037837838	GO:0048522	GO:BP	positive regulation of cellular process	FALSE
1.69E-05	1294	267	60	0.224719101	0.046367852	GO:0042221	GO:BP	response to chemical	FALSE
2.57E-05	910	267	47	0.176029963	0.051648352	GO:0009605	GO:BP	response to external stimulus	FALSE
4.61E-05	1736	267	72	0.269662921	0.041474654	GO:0048583	GO:BP	regulation of response to stimulus	FALSE
5.16E-05	1745	267	72	0.269662921	0.041260745	GO:0007275	GO:BP	multicellular organism development	FALSE
5.16E-05	2347	267	89	0.333333333	0.03792075	GO:0048519	GO:BP	negative regulation of biological process	FALSE
0.00010217	1474	267	63	0.235955056	0.042740841	GO:0023051	GO:BP	regulation of signaling	FALSE
0.00011179	2247	267	85	0.31835206	0.037828215	GO:0048856	GO:BP	anatomical structure development	FALSE
0.000139939	1102	267	51	0.191011236	0.046279492	GO:0002376	GO:BP	immune system process	FALSE
0.000139939	1427	267	61	0.228464419	0.042747022	GO:0048731	GO:BP	system development	FALSE
0.000167667	1470	267	62	0.232209738	0.042176871	GO:0010646	GO:BP	regulation of cell communication	FALSE
0.000187442	2284	267	85	0.31835206	0.037215412	GO:0048523	GO:BP	negative regulation of cellular process	FALSE
0.000207633	10	267	5	0.018726592	0.5	GO:0071498	GO:BP	cellular response to fluid shear stress	FALSE
0.000211698	253	267	20	0.074906367	0.079051383	GO:0008285	GO:BP	negative regulation of cell population proliferation	FALSE
0.000229963	1696	267	68	0.254681648	0.04009434	GO:0006950	GO:BP	response to stress	FALSE
0.000273837	1333	267	57	0.213483146	0.04276069	GO:0009966	GO:BP	regulation of signal transduction	FALSE
0.000367223	546	267	31	0.116104869	0.056776557	GO:1901700	GO:BP	response to oxygen-containing compound	FALSE
0.00050634	1602	267	64	0.239700375	0.039950062	GO:0030154	GO:BP	cell differentiation	FALSE
0.00050634	1602	267	64	0.239700375	0.039950062	GO:0048869	GO:BP	cellular developmental process	FALSE
0.000877734	782	267	38	0.142322097	0.04859335	GO:0012501	GO:BP	programmed cell death	FALSE

0.000877734	309	267	21	0.078651685	0.067961165	GO:0006897	GO:BP	endocytosis	FALSE
0.000877734	811	267	39	0.146067416	0.048088779	GO:0070887	GO:BP	cellular response to chemical stimulus	FALSE
0.000877734	782	267	38	0.142322097	0.04859335	GO:0008219	GO:BP	cell death	FALSE
0.000877734	689	267	35	0.131086142	0.050798258	GO:0006952	GO:BP	defense response	FALSE
0.000877734	309	267	21	0.078651685	0.067961165	GO:0001568	GO:BP	blood vessel development	FALSE
0.000887901	215	267	17	0.063670412	0.079069767	GO:0001525	GO:BP	angiogenesis	FALSE
0.000898026	1037	267	46	0.172284644	0.044358727	GO:0009653	GO:BP	anatomical structure morphogenesis	FALSE
0.000898026	1470	267	59	0.220973783	0.040136054	GO:0010604	GO:BP	positive regulation of macromolecule metabolic process	FALSE
0.000962267	218	267	17	0.063670412	0.077981651	GO:0043254	GO:BP	regulation of protein-containing complex assembly	FALSE
0.000962267	1010	267	45	0.168539326	0.044554455	GO:0048584	GO:BP	positive regulation of response to stimulus	FALSE
0.001006536	3	267	3	0.011235955	1	GO:0071499	GO:BP	cellular response to laminar fluid shear stress	FALSE
0.001082516	24	267	6	0.02247191	0.25	GO:0032526	GO:BP	response to retinoic acid	FALSE
0.001100874	449	267	26	0.097378277	0.057906459	GO:0035295	GO:BP	tube development	FALSE
0.001144601	1420	267	57	0.213483146	0.040140845	GO:0035556	GO:BP	intracellular signal transduction	FALSE
0.00121193	1123	267	48	0.179775281	0.042742654	GO:0048468	GO:BP	cell development	FALSE
0.00121193	744	267	36	0.134831461	0.048387097	GO:0006915	GO:BP	apoptotic process	FALSE
0.00121193	653	267	33	0.123595506	0.050535988	GO:0051240	GO:BP	positive regulation of multicellular organismal process	FALSE
0.00121193	2472	267	86	0.322097378	0.034789644	GO:0051179	GO:BP	localization	FALSE
0.001559021	328	267	21	0.078651685	0.06402439	GO:0001944	GO:BP	vasculature development	FALSE
0.001559113	462	267	26	0.097378277	0.056277056	GO:0048646	GO:BP	anatomical structure formation involved in morphogenesis	FALSE
0.001793026	358	267	22	0.082397004	0.061452514	GO:0035239	GO:BP	tube morphogenesis	FALSE
0.001848397	762	267	36	0.134831461	0.047244094	GO:0008283	GO:BP	cell population proliferation	FALSE
0.001985242	17	267	5	0.018726592	0.294117647	GO:0034405	GO:BP	response to fluid shear stress	FALSE
0.002020093	1086	267	46	0.172284644	0.042357274	GO:0007166	GO:BP	cell surface receptor signaling pathway	FALSE
0.002290997	1605	267	61	0.228464419	0.038006231	GO:0009893	GO:BP	positive regulation of metabolic process	FALSE
0.002339511	1195	267	49	0.183520599	0.041004184	GO:0009891	GO:BP	positive regulation of biosynthetic process	FALSE
0.002339511	264	267	18	0.06741573	0.068181818	GO:0048514	GO:BP	blood vessel morphogenesis	FALSE
0.002339511	1162	267	48	0.179775281	0.04130809	GO:0010557	GO:BP	positive regulation of macromolecule biosynthetic process	FALSE
0.002383199	369	267	22	0.082397004	0.059620596	GO:0098657	GO:BP	import into cell	FALSE
0.002383199	777	267	36	0.134831461	0.046332046	GO:0016192	GO:BP	vesicle-mediated transport	FALSE
0.002383199	423	267	24	0.08988764	0.056737589	GO:1901701	GO:BP	cellular response to oxygen-containing compound	FALSE
0.002454928	1938	267	70	0.262172285	0.036119711	GO:0006810	GO:BP	transport	FALSE
0.002526706	482	267	26	0.097378277	0.053941909	GO:0030097	GO:BP	hemopoiesis	FALSE
0.002526706	1170	267	48	0.179775281	0.041025641	GO:0051239	GO:BP	regulation of multicellular organismal process	FALSE
0.002643913	58	267	8	0.029962547	0.137931034	GO:1903034	GO:BP	regulation of response to wounding	FALSE
0.002669665	1074	267	45	0.168539326	0.041899441	GO:0051128	GO:BP	regulation of cellular component organization	FALSE

0.003009532	727	267	34	0.127340824	0.046767538	GO:0023056	GO:BP	positive regulation of signaling	FALSE
0.003116629	490	267	26	0.097378277	0.053061224	GO:0044087	GO:BP	regulation of cellular component biogenesis	FALSE
0.003611325	2115	267	74	0.277153558	0.03498818	GO:0051234	GO:BP	establishment of localization	FALSE
0.003787913	468	267	25	0.093632959	0.053418803	GO:0072359	GO:BP	circulatory system development	FALSE
0.003874548	11	267	4	0.014981273	0.363636364	GO:0002115	GO:BP	store-operated calcium entry	TRUE
0.004410696	33	267	6	0.02247191	0.181818182	GO:1903035	GO:BP	negative regulation of response to wounding	FALSE
0.004478567	309	267	19	0.071161049	0.061488673	GO:0030036	GO:BP	actin cytoskeleton organization	TRUE
0.004666418	972	267	41	0.153558052	0.04218107	GO:0050793	GO:BP	regulation of developmental process	FALSE
0.005002457	720	267	33	0.123595506	0.045833333	GO:0010647	GO:BP	positive regulation of cell communication	FALSE
0.005002457	688	267	32	0.119850187	0.046511628	GO:0044419	GO:BP	biological process involved in interspecies interaction between organisms	FALSE
0.005002457	236	267	16	0.059925094	0.06779661	GO:0001819	GO:BP	positive regulation of cytokine production	FALSE
0.005201409	12	267	4	0.014981273	0.333333333	GO:0031639	GO:BP	plasminogen activation	TRUE
0.005289464	818	267	36	0.134831461	0.04400978	GO:0141124	GO:BP	intracellular signaling cassette	FALSE
0.005692582	168	267	13	0.048689139	0.077380952	GO:1990778	GO:BP	protein localization to cell periphery	TRUE
0.005692582	344	267	20	0.074906367	0.058139535	GO:0030029	GO:BP	actin filament-based process	FALSE
0.005997406	667	267	31	0.116104869	0.046476762	GO:0009967	GO:BP	positive regulation of signal transduction	FALSE
0.006057576	637	267	30	0.112359551	0.047095761	GO:0009607	GO:BP	response to biotic stimulus	FALSE
0.006253496	608	267	29	0.108614232	0.047697368	GO:0043067	GO:BP	regulation of programmed cell death	FALSE
0.006496096	106	267	10	0.037453184	0.094339623	GO:0006909	GO:BP	phagocytosis	FALSE
0.006529601	322	267	19	0.071161049	0.059006211	GO:0051129	GO:BP	negative regulation of cellular component organization	FALSE
0.008000307	109	267	10	0.037453184	0.091743119	GO:0031334	GO:BP	positive regulation of protein-containing complex assembly	FALSE
0.008225029	713	267	32	0.119850187	0.044880785	GO:0006955	GO:BP	immune response	FALSE
0.00870037	278	267	17	0.063670412	0.061151079	GO:0033993	GO:BP	response to lipid	FALSE
0.00870037	653	267	30	0.112359551	0.045941807	GO:0080134	GO:BP	regulation of response to stress	FALSE
0.00870037	14	267	4	0.014981273	0.285714286	GO:1900407	GO:BP	regulation of cellular response to oxidative stress	FALSE
0.00870037	359	267	20	0.074906367	0.055710306	GO:0001817	GO:BP	regulation of cytokine production	FALSE
0.00870037	6	267	3	0.011235955	0.5	GO:0034616	GO:BP	response to laminar fluid shear stress	FALSE
0.00870037	331	267	19	0.071161049	0.057401813	GO:0022603	GO:BP	regulation of anatomical structure morphogenesis	FALSE
0.009062977	55	267	7	0.026217228	0.127272727	GO:0061515	GO:BP	myeloid cell development	FALSE
0.009830013	363	267	20	0.074906367	0.055096419	GO:0001816	GO:BP	cytokine production	FALSE
0.009994322	1054	267	42	0.157303371	0.039848197	GO:0048513	GO:BP	animal organ development	FALSE
0.009994322	364	267	20	0.074906367	0.054945055	GO:0043069	GO:BP	negative regulation of programmed cell death	FALSE

0.010912783	115	267	10	0.037453184	0.086956522	GO:2001234	GO:BP	negative regulation of apoptotic signaling pathway	FALSE
0.010917789	483	267	24	0.08988764	0.049689441	GO:0032101	GO:BP	regulation of response to external stimulus	FALSE
0.010917789	233	267	15	0.056179775	0.064377682	GO:0060627	GO:BP	regulation of vesicle-mediated transport	FALSE
0.011233204	606	267	28	0.104868914	0.04620462	GO:0042127	GO:BP	regulation of cell population proliferation	FALSE
0.011233204	606	267	28	0.104868914	0.04620462	GO:0051049	GO:BP	regulation of transport	FALSE
0.011707013	897	267	37	0.138576779	0.041248606	GO:1902531	GO:BP	regulation of intracellular signal transduction	FALSE
0.011844521	343	267	19	0.071161049	0.055393586	GO:0043066	GO:BP	negative regulation of apoptotic process	FALSE
0.011844521	578	267	27	0.101123596	0.046712803	GO:0042981	GO:BP	regulation of apoptotic process	FALSE
0.012772987	7	267	3	0.011235955	0.428571429	GO:0010755	GO:BP	regulation of plasminogen activation	FALSE
0.012772987	16	267	4	0.014981273	0.25	GO:0035855	GO:BP	megakaryocyte development	FALSE
0.012772987	238	267	15	0.056179775	0.06302521	GO:0044089	GO:BP	positive regulation of cellular component biogenesis	FALSE
0.01291372	552	267	26	0.097378277	0.047101449	GO:0045944	GO:BP	positive regulation of transcription by RNA polymerase II	FALSE
0.013281337	807	267	34	0.127340824	0.042131351	GO:0032879	GO:BP	regulation of localization	FALSE
0.01328471	142	267	11	0.041198502	0.077464789	GO:0072659	GO:BP	protein localization to plasma membrane	FALSE
0.01328471	554	267	26	0.097378277	0.046931408	GO:0010628	GO:BP	positive regulation of gene expression	FALSE
0.014126669	526	267	25	0.093632959	0.047528517	GO:0002684	GO:BP	positive regulation of immune system process	FALSE
0.014209446	588	267	27	0.101123596	0.045918367	GO:0045595	GO:BP	regulation of cell differentiation	FALSE
0.014209446	242	267	15	0.056179775	0.061983471	GO:0007169	GO:BP	cell surface receptor protein tyrosine kinase signaling pathway	FALSE
0.014260478	323	267	18	0.06741573	0.055727554	GO:0045597	GO:BP	positive regulation of cell differentiation	FALSE
0.015162685	17	267	4	0.014981273	0.235294118	GO:1903670	GO:BP	regulation of sprouting angiogenesis	FALSE
0.015195544	687	267	30	0.112359551	0.043668122	GO:0006796	GO:BP	phosphate-containing compound metabolic process	TRUE
0.015195544	2	267	2	0.007490637	1	GO:0042997	GO:BP	negative regulation of Golgi to plasma membrane protein transport	TRUE
0.015195544	2	267	2	0.007490637	1	GO:0032462	GO:BP	regulation of protein homooligomerization	TRUE
0.01536215	688	267	30	0.112359551	0.043604651	GO:0006793	GO:BP	phosphorus metabolic process	FALSE
0.015993908	564	267	26	0.097378277	0.046099291	GO:2000026	GO:BP	regulation of multicellular organismal development	FALSE
0.017244805	8	267	3	0.011235955	0.375	GO:0110075	GO:BP	regulation of ferroptosis	FALSE
0.017244805	8	267	3	0.011235955	0.375	GO:0097707	GO:BP	ferroptosis	FALSE
0.017452944	727	267	31	0.116104869	0.04264099	GO:0002682	GO:BP	regulation of immune system process	FALSE

0.01753906	31	267	5	0.018726592	0.161290323	GO:0030500	GO:BP	regulation of bone mineralization	FALSE
0.01753906	447	267	22	0.082397004	0.049217002	GO:0071495	GO:BP	cellular response to endogenous stimulus	FALSE
0.017815856	304	267	17	0.063670412	0.055921053	GO:0006954	GO:BP	inflammatory response	FALSE
0.018296453	150	267	11	0.041198502	0.073333333	GO:0001503	GO:BP	ossification	FALSE
0.019468661	85	267	8	0.029962547	0.094117647	GO:0016052	GO:BP	carbohydrate catabolic process	TRUE
0.019811882	48	267	6	0.02247191	0.125	GO:0032760	GO:BP	positive regulation of tumor necrosis factor production	FALSE
0.019811882	48	267	6	0.02247191	0.125	GO:0030282	GO:BP	bone mineralization	FALSE
0.019811882	280	267	16	0.059925094	0.057142857	GO:0002252	GO:BP	immune effector process	FALSE
0.019911152	608	267	27	0.101123596	0.044407895	GO:0043207	GO:BP	response to external biotic stimulus	FALSE
0.019911152	129	267	10	0.037453184	0.07751938	GO:0045765	GO:BP	regulation of angiogenesis	FALSE
0.019911152	608	267	27	0.101123596	0.044407895	GO:0051707	GO:BP	response to other organism	FALSE
0.021056492	49	267	6	0.02247191	0.12244898	GO:1901343	GO:BP	negative regulation of vasculature development	FALSE
0.021056492	49	267	6	0.02247191	0.12244898	GO:0016525	GO:BP	negative regulation of angiogenesis	FALSE
0.021056492	49	267	6	0.02247191	0.12244898	GO:1903557	GO:BP	positive regulation of tumor necrosis factor superfamily cytokine production	FALSE
0.021056492	49	267	6	0.02247191	0.12244898	GO:2000181	GO:BP	negative regulation of blood vessel morphogenesis	FALSE
0.021319097	774	267	32	0.119850187	0.041343669	GO:0048585	GO:BP	negative regulation of response to stimulus	FALSE
0.021319614	2561	267	81	0.303370787	0.03162827	GO:0060255	GO:BP	regulation of macromolecule metabolic process	FALSE
0.021609439	1080	267	41	0.153558052	0.037962963	GO:0065008	GO:BP	regulation of biological quality	FALSE
0.021667751	520	267	24	0.08988764	0.046153846	GO:0001775	GO:BP	cell activation	FALSE
0.021667751	399	267	20	0.074906367	0.050125313	GO:0051247	GO:BP	positive regulation of protein metabolic process	FALSE
0.021961344	132	267	10	0.037453184	0.075757576	GO:1901342	GO:BP	regulation of vasculature development	FALSE
0.023198732	20	267	4	0.014981273	0.2	GO:0031638	GO:BP	zymogen activation	FALSE
0.023198732	34	267	5	0.018726592	0.147058824	GO:0006900	GO:BP	vesicle budding from membrane	FALSE
0.023198732	344	267	18	0.06741573	0.052325581	GO:0097435	GO:BP	supramolecular fiber organization	FALSE
0.02352647	403	267	20	0.074906367	0.049627792	GO:0046649	GO:BP	lymphocyte activation	FALSE
0.024450883	346	267	18	0.06741573	0.052023121	GO:0098609	GO:BP	cell-cell adhesion	TRUE
0.024545234	987	267	38	0.142322097	0.038500507	GO:0006357	GO:BP	regulation of transcription by RNA polymerase II	FALSE
0.024545234	209	267	13	0.048689139	0.062200957	GO:0009611	GO:BP	response to wounding	FALSE
0.026552902	499	267	23	0.086142322	0.046092184	GO:0051094	GO:BP	positive regulation of developmental process	FALSE
0.026884335	21	267	4	0.014981273	0.19047619	GO:1902882	GO:BP	regulation of response to oxidative stress	FALSE
0.027352607	137	267	10	0.037453184	0.072992701	GO:0043122	GO:BP	regulation of canonical NF-kappaB signal transduction	FALSE
0.027426545	239	267	14	0.052434457	0.058577406	GO:0120035	GO:BP	regulation of plasma membrane bounded cell projection organization	FALSE
0.027426545	501	267	23	0.086142322	0.045908184	GO:0009719	GO:BP	response to endogenous stimulus	FALSE

0.027426545	410	267	20	0.074906367	0.048780488	GO:0051241	GO:BP	negative regulation of multicellular organismal process	FALSE
0.027514031	10	267	3	0.011235955	0.3	GO:0070935	GO:BP	3'-UTR-mediated mRNA stabilization	TRUE
0.027514031	323	267	17	0.063670412	0.052631579	GO:0051093	GO:BP	negative regulation of developmental process	FALSE
0.027514031	240	267	14	0.052434457	0.058333333	GO:0030099	GO:BP	myeloid cell differentiation	FALSE
0.027514031	138	267	10	0.037453184	0.072463768	GO:0001558	GO:BP	regulation of cell growth	FALSE
0.027514031	53	267	6	0.02247191	0.113207547	GO:0030278	GO:BP	regulation of ossification	FALSE
0.027514031	240	267	14	0.052434457	0.058333333	GO:0031344	GO:BP	regulation of cell projection organization	FALSE
0.028469169	383	267	19	0.071161049	0.049608355	GO:0045087	GO:BP	innate immune response	FALSE
0.029979511	22	267	4	0.014981273	0.181818182	GO:0045806	GO:BP	negative regulation of endocytosis	FALSE
0.03153444	3	267	2	0.007490637	0.666666667	GO:0002755	GO:BP	MyD88-dependent toll-like receptor signaling pathway	TRUE
0.03153444	3	267	2	0.007490637	0.666666667	GO:0032459	GO:BP	regulation of protein oligomerization	FALSE
0.03153444	3	267	2	0.007490637	0.666666667	GO:1905443	GO:BP	regulation of clathrin coat assembly	FALSE
0.03153444	3	267	2	0.007490637	0.666666667	GO:0010756	GO:BP	positive regulation of plasminogen activation	FALSE
0.03153444	358	267	18	0.06741573	0.05027933	GO:0051668	GO:BP	localization within membrane	FALSE
0.033206339	38	267	5	0.018726592	0.131578947	GO:0070167	GO:BP	regulation of biomineral tissue development	FALSE
0.033206339	2304	267	73	0.27340824	0.031684028	GO:0080090	GO:BP	regulation of primary metabolic process	FALSE
0.033206339	119	267	9	0.033707865	0.075630252	GO:0030100	GO:BP	regulation of endocytosis	FALSE
0.033928609	391	267	19	0.071161049	0.04859335	GO:0140546	GO:BP	defense response to symbiont	FALSE
0.033928609	56	267	6	0.02247191	0.107142857	GO:2001243	GO:BP	negative regulation of intrinsic apoptotic signaling pathway	FALSE
0.034172815	194	267	12	0.04494382	0.06185567	GO:0007159	GO:BP	leukocyte cell-cell adhesion	FALSE
0.034518381	392	267	19	0.071161049	0.048469388	GO:0034097	GO:BP	response to cytokine	FALSE
0.035146997	393	267	19	0.071161049	0.048346056	GO:1901652	GO:BP	response to peptide	FALSE
0.035146997	1051	267	39	0.146067416	0.037107517	GO:0006366	GO:BP	transcription by RNA polymerase II	FALSE
0.03561211	57	267	6	0.02247191	0.105263158	GO:0002040	GO:BP	sprouting angiogenesis	FALSE
0.03561211	57	267	6	0.02247191	0.105263158	GO:0051851	GO:BP	host-mediated perturbation of symbiont process	FALSE
0.03561211	424	267	20	0.074906367	0.047169811	GO:0009628	GO:BP	response to abiotic stimulus	FALSE
0.03561211	613	267	26	0.097378277	0.042414356	GO:0016477	GO:BP	cell migration	TRUE
0.036666192	307	267	16	0.059925094	0.052117264	GO:0072657	GO:BP	protein localization to membrane	FALSE
0.037561881	24	267	4	0.014981273	0.166666667	GO:0061045	GO:BP	negative regulation of wound healing	FALSE
0.038355665	58	267	6	0.02247191	0.103448276	GO:0007229	GO:BP	integrin-mediated signaling pathway	FALSE
0.038465933	490	267	22	0.082397004	0.044897959	GO:0051130	GO:BP	positive regulation of cellular component organization	FALSE
0.041098063	12	267	3	0.011235955	0.25	GO:0046471	GO:BP	phosphatidylglycerol metabolic process	TRUE
0.041098063	2801	267	85	0.31835206	0.030346305	GO:0019222	GO:BP	regulation of metabolic process	FALSE

0.041098063	59	267	6	0.02247191	0.101694915	GO:0031214	GO:BP	biomineral tissue development	FALSE
0.042189514	25	267	4	0.014981273	0.16	GO:0030193	GO:BP	regulation of blood coagulation	FALSE
0.042189514	495	267	22	0.082397004	0.044444444	GO:0040011	GO:BP	locomotion	TRUE
0.042189514	372	267	18	0.06741573	0.048387097	GO:0031347	GO:BP	regulation of defense response	FALSE
0.042189514	25	267	4	0.014981273	0.16	GO:0034389	GO:BP	lipid droplet organization	TRUE
0.042735611	150	267	10	0.037453184	0.066666667	GO:0007249	GO:BP	canonical NF-kappaB signal transduction	FALSE
0.043324571	758	267	30	0.112359551	0.039577836	GO:0045893	GO:BP	positive regulation of DNA-templated transcription	FALSE
0.043589994	725	267	29	0.108614232	0.04	GO:0042592	GO:BP	homeostatic process	TRUE
0.043963296	151	267	10	0.037453184	0.066225166	GO:0050727	GO:BP	regulation of inflammatory response	FALSE
0.043963296	760	267	30	0.112359551	0.039473684	GO:1902680	GO:BP	positive regulation of RNA biosynthetic process	FALSE
0.043963296	693	267	28	0.104868914	0.04040404	GO:0048870	GO:BP	cell motility	FALSE
0.043963296	203	267	12	0.04494382	0.0591133	GO:0007015	GO:BP	actin filament organization	FALSE
0.04456338	628	267	26	0.097378277	0.041401274	GO:0009968	GO:BP	negative regulation of signal transduction	FALSE
0.04456338	42	267	5	0.018726592	0.119047619	GO:0002532	GO:BP	production of molecular mediator involved in inflammatory response	FALSE
0.045315042	61	267	6	0.02247191	0.098360656	GO:0035821	GO:BP	modulation of process of another organism	FALSE
0.045315042	104	267	8	0.029962547	0.076923077	GO:0120032	GO:BP	regulation of plasma membrane bounded cell projection assembly	FALSE
0.045487049	377	267	18	0.06741573	0.047745358	GO:0007167	GO:BP	enzyme-linked receptor protein signaling pathway	FALSE
0.045487049	26	267	4	0.014981273	0.153846154	GO:0045453	GO:BP	bone resorption	TRUE
0.045487049	26	267	4	0.014981273	0.153846154	GO:0032732	GO:BP	positive regulation of interleukin-1 production	FALSE
0.045705791	470	267	21	0.078651685	0.044680851	GO:0045321	GO:BP	leukocyte activation	FALSE
0.045705791	598	267	25	0.093632959	0.04180602	GO:0022008	GO:BP	neurogenesis	FALSE
0.046758671	105	267	8	0.029962547	0.076190476	GO:0060491	GO:BP	regulation of cell projection assembly	FALSE
0.047176278	13	267	3	0.011235955	0.230769231	GO:0044794	GO:BP	positive regulation by host of viral process	TRUE
0.049035563	4	267	2	0.007490637	0.5	GO:1904997	GO:BP	regulation of leukocyte adhesion to arterial endothelial cell	TRUE
0.049035563	4	267	2	0.007490637	0.5	GO:0046985	GO:BP	positive regulation of hemoglobin biosynthetic process	FALSE
0.049035563	4	267	2	0.007490637	0.5	GO:0061757	GO:BP	leukocyte adhesion to arterial endothelial cell	FALSE
0.049035563	4	267	2	0.007490637	0.5	GO:0030948	GO:BP	negative regulation of vascular endothelial growth factor receptor signaling pathway	FALSE
4.21E-08	3584	267	132	0.494382022	0.036830357	GO:0016020	GO:CC	membrane	TRUE
0.000116313	1925	267	76	0.284644195	0.039480519	GO:0071944	GO:CC	cell periphery	FALSE
0.000451528	1765	267	69	0.258426966	0.039093484	GO:0005886	GO:CC	plasma membrane	FALSE
0.003958583	1933	267	70	0.262172285	0.03621314	GO:0012505	GO:CC	endomembrane system	TRUE
0.006633206	5580	267	159	0.595505618	0.028494624	GO:0005737	GO:CC	cytoplasm	TRUE
0.031353536	2131	267	71	0.265917603	0.033317691	GO:0005829	GO:CC	cytosol	FALSE

0.048472197	109	267	9	0.033707865	0.082568807	GO:0009925	GO:CC	basal plasma membrane	TRUE
0.048472197	20	267	4	0.014981273	0.2	GO:0002102	GO:CC	podosome	TRUE
0.012750758	165	267	14	0.052434457	0.084848485	GO:0001228	GO:MF	DNA-binding transcription activator activity, RNA polymerase II-specific	FALSE
0.012750758	170	267	14	0.052434457	0.082352941	GO:0001216	GO:MF	DNA-binding transcription activator activity	TRUE
0.020536267	361	267	21	0.078651685	0.058171745	GO:0000981	GO:MF	DNA-binding transcription factor activity, RNA polymerase II-specific	FALSE
0.022037265	398	267	22	0.082397004	0.055276382	GO:0003700	GO:MF	DNA-binding transcription factor activity	FALSE
0.049788953	2844	267	89	0.333333333	0.031293952	GO:0043167	GO:MF	ion binding	TRUE
0.049788953	1115	267	43	0.161048689	0.038565022	GO:0019899	GO:MF	enzyme binding	TRUE
0.049788953	2	267	2	0.007490637	1	GO:0015355	GO:MF	secondary active monocarboxylate transmembrane transporter activity	TRUE
0.049788953	17	267	4	0.014981273	0.235294118	GO:0008028	GO:MF	monocarboxylic acid transmembrane transporter activity	FALSE
0.049788953	664	267	29	0.108614232	0.043674699	GO:0140110	GO:MF	transcription regulator activity	FALSE
0.049804923	2938	267	91	0.34082397	0.030973451	GO:0036094	GO:MF	small molecule binding	FALSE
0.022315892	4554	267	135	0.505617978	0.029644269	KEGG:00000	KEGG	KEGG root term	FALSE
0.034707085	56	267	7	0.026217228	0.125	KEGG:05223	KEGG	Non-small cell lung cancer	FALSE

Salmon Module GO Enrichment

p value	*term size*	*query size*	*overlap size*	*precision*	*recall*	*term id*	*source*	*term name*	*highlighted*
0.008296082	8	70	3	0.042857143	0.375	GO:0001660	GO:BP	fever generation	FALSE
0.008296082	28	70	4	0.057142857	0.142857143	GO:0002526	GO:BP	acute inflammatory response	FALSE
0.008296082	24	70	4	0.057142857	0.166666667	GO:0048661	GO:BP	positive regulation of smooth muscle cell proliferation	TRUE
0.008296082	10	70	3	0.042857143	0.3	GO:0006953	GO:BP	acute-phase response	TRUE
0.008296082	10	70	3	0.042857143	0.3	GO:0031649	GO:BP	heat generation	FALSE
0.019927237	73	70	5	0.071428571	0.068493151	GO:0045766	GO:BP	positive regulation of angiogenesis	FALSE
0.019927237	75	70	5	0.071428571	0.066666667	GO:1904018	GO:BP	positive regulation of vasculature development	TRUE
0.019927237	40	70	4	0.057142857	0.1	GO:0048660	GO:BP	regulation of smooth muscle cell proliferation	FALSE
0.020769222	42	70	4	0.057142857	0.095238095	GO:0048659	GO:BP	smooth muscle cell proliferation	FALSE
0.020769222	129	70	6	0.085714286	0.046511628	GO:0045765	GO:BP	regulation of angiogenesis	FALSE
0.02142746	132	70	6	0.085714286	0.045454545	GO:1901342	GO:BP	regulation of vasculature development	FALSE
0.03525985	94	70	5	0.071428571	0.053191489	GO:0016485	GO:BP	protein processing	TRUE
0.03887912	22	70	3	0.042857143	0.136363636	GO:0010573	GO:BP	vascular endothelial growth factor production	TRUE
0.040121865	724	70	13	0.185714286	0.017955801	GO:0006508	GO:BP	proteolysis	FALSE
0.040121865	5	70	2	0.028571429	0.4	GO:0031620	GO:BP	regulation of fever generation	FALSE
0.04614434	163	70	6	0.085714286	0.036809816	GO:0042060	GO:BP	wound healing	TRUE
0.049956938	6	70	2	0.028571429	0.333333333	GO:0002544	GO:BP	chronic inflammatory response	TRUE

0.049956938	6	70	2	0.028571429	0.333333333	GO:0031650	GO:BP	regulation of heat generation	FALSE
0.024817128	476	70	10	0.142857143	0.021008403	GO:0005576	GO:CC	extracellular region	TRUE
0.024817128	6	70	2	0.028571429	0.333333333	GO:0034663	GO:CC	endoplasmic reticulum chaperone complex	TRUE
0.024817128	265	70	8	0.114285714	0.030188679	GO:0009986	GO:CC	cell surface	FALSE
0.024817128	108	70	5	0.071428571	0.046296296	GO:0009897	GO:CC	external side of plasma membrane	TRUE
0.007688508	4554	70	44	0.628571429	0.009661836	KEGG:00000	KEGG	KEGG root term	FALSE
0.026549878	101	70	5	0.071428571	0.04950495	KEGG:04060	KEGG	Cytokine-cytokine receptor interaction	FALSE
0.03193916	33	70	3	0.042857143	0.090909091	KEGG:05321	KEGG	Inflammatory bowel disease	FALSE
0.03193916	9	70	2	0.028571429	0.222222222	KEGG:05310	KEGG	Asthma	FALSE
0.03193916	30	70	3	0.042857143	0.1	KEGG:05144	KEGG	Malaria	FALSE
0.03193916	78	70	4	0.057142857	0.051282051	KEGG:04933	KEGG	AGE-RAGE signaling pathway in diabetic complications	FALSE
0.037532984	289	70	7	0.1	0.024221453	KEGG:05022	KEGG	Pathways of neurodegeneration - multiple diseases	FALSE
0.040669671	151	70	5	0.071428571	0.033112583	KEGG:05417	KEGG	Lipid and atherosclerosis	FALSE
0.046538472	233	70	6	0.085714286	0.025751073	KEGG:05014	KEGG	Amyotrophic lateral sclerosis	FALSE
0.046551161	164	70	5	0.071428571	0.030487805	KEGG:05020	KEGG	Prion disease	FALSE
0.047730177	105	70	4	0.057142857	0.038095238	KEGG:05164	KEGG	Influenza A	FALSE
0.047730177	52	70	3	0.042857143	0.057692308	KEGG:05323	KEGG	Rheumatoid arthritis	FALSE

Tan Module GO Enrichment

p value	*term size*	*query size*	*overlap size*	*precision*	*recall*	*term id*	*source*	*term name*	*highlighted*
1.41E-06	20	81	6	0.074074074	0.3	GO:0006779	GO:BP	porphyrin-containing compound biosynthetic process	TRUE
1.41E-06	20	81	6	0.074074074	0.3	GO:0033014	GO:BP	tetrapyrrole biosynthetic process	FALSE
1.41E-06	18	81	6	0.074074074	0.333333333	GO:0006783	GO:BP	heme biosynthetic process	FALSE
2.02E-06	11	81	5	0.061728395	0.454545455	GO:0046501	GO:BP	protoporphyrinogen IX metabolic process	FALSE
2.87E-06	24	81	6	0.074074074	0.25	GO:0042168	GO:BP	heme metabolic process	FALSE
4.05E-06	26	81	6	0.074074074	0.230769231	GO:0006778	GO:BP	porphyrin-containing compound metabolic process	FALSE
5.62E-06	28	81	6	0.074074074	0.214285714	GO:0046148	GO:BP	pigment biosynthetic process	FALSE
9.45E-06	31	81	6	0.074074074	0.193548387	GO:0033013	GO:BP	tetrapyrrole metabolic process	FALSE
1.77E-05	8	81	4	0.049382716	0.5	GO:0006785	GO:BP	heme B biosynthetic process	FALSE
1.77E-05	36	81	6	0.074074074	0.166666667	GO:0042440	GO:BP	pigment metabolic process	FALSE
1.77E-05	8	81	4	0.049382716	0.5	GO:0046492	GO:BP	heme B metabolic process	FALSE
2.38E-05	9	81	4	0.049382716	0.444444444	GO:0048034	GO:BP	heme O biosynthetic process	FALSE
2.38E-05	9	81	4	0.049382716	0.444444444	GO:0006782	GO:BP	protoporphyrinogen IX biosynthetic process	FALSE
2.38E-05	9	81	4	0.049382716	0.444444444	GO:0048033	GO:BP	heme O metabolic process	FALSE
3.46E-05	10	81	4	0.049382716	0.4	GO:0006784	GO:BP	heme A biosynthetic process	FALSE
3.46E-05	10	81	4	0.049382716	0.4	GO:0046160	GO:BP	heme a metabolic process	FALSE

0.002320807	723	81	16	0.197530864	0.022130014	GO:0044281	GO:BP	small molecule metabolic process	TRUE
0.002347744	10	81	3	0.037037037	0.3	GO:0009263	GO:BP	deoxyribonucleotide biosynthetic process	FALSE
0.002347744	10	81	3	0.037037037	0.3	GO:0046385	GO:BP	deoxyribose phosphate biosynthetic process	FALSE
0.002347744	10	81	3	0.037037037	0.3	GO:0009265	GO:BP	2'-deoxyribonucleotide biosynthetic process	FALSE
0.004654678	202	81	8	0.098765432	0.03960396	GO:0009117	GO:BP	nucleotide metabolic process	FALSE
0.006588744	274	81	9	0.111111111	0.032846715	GO:0006753	GO:BP	nucleoside phosphate metabolic process	FALSE
0.00806602	353	81	10	0.12345679	0.028328612	GO:0043436	GO:BP	oxoacid metabolic process	FALSE
0.00806602	286	81	9	0.111111111	0.031468531	GO:0055086	GO:BP	nucleobase-containing small molecule metabolic process	FALSE
0.00806602	351	81	10	0.12345679	0.028490028	GO:0019752	GO:BP	carboxylic acid metabolic process	FALSE
0.008308717	356	81	10	0.12345679	0.028089888	GO:0006082	GO:BP	organic acid metabolic process	FALSE
0.008885087	121	81	6	0.074074074	0.049586777	GO:1901293	GO:BP	nucleoside phosphate biosynthetic process	FALSE
0.010288582	80	81	5	0.061728395	0.0625	GO:0009165	GO:BP	nucleotide biosynthetic process	FALSE
0.018136844	93	81	5	0.061728395	0.053763441	GO:0030218	GO:BP	erythrocyte differentiation	TRUE
0.018136844	22	81	3	0.037037037	0.136363636	GO:0019692	GO:BP	deoxyribose phosphate metabolic process	FALSE
0.018136844	22	81	3	0.037037037	0.136363636	GO:0009394	GO:BP	2'-deoxyribonucleotide metabolic process	FALSE
0.018136844	22	81	3	0.037037037	0.136363636	GO:0009262	GO:BP	deoxyribonucleotide metabolic process	FALSE
0.018287479	265	81	8	0.098765432	0.030188679	GO:0090407	GO:BP	organophosphate biosynthetic process	FALSE
0.018287479	95	81	5	0.061728395	0.052631579	GO:0034101	GO:BP	erythrocyte homeostasis	FALSE
0.018287479	484	81	11	0.135802469	0.022727273	GO:0019637	GO:BP	organophosphate metabolic process	FALSE
0.024397816	6	81	2	0.024691358	0.333333333	GO:0009157	GO:BP	deoxyribonucleoside monophosphate biosynthetic process	FALSE
0.028399661	27	81	3	0.037037037	0.111111111	GO:0009124	GO:BP	nucleoside monophosphate biosynthetic process	FALSE
0.034165709	111	81	5	0.061728395	0.045045045	GO:0009141	GO:BP	nucleoside triphosphate metabolic process	FALSE
0.036066058	113	81	5	0.061728395	0.044247788	GO:0002262	GO:BP	myeloid cell homeostasis	FALSE
0.037650807	171	81	6	0.074074074	0.035087719	GO:0006163	GO:BP	purine nucleotide metabolic process	FALSE
0.039627799	8	81	2	0.024691358	0.25	GO:0071392	GO:BP	cellular response to estradiol stimulus	TRUE
0.003689182	846	81	18	0.222222222	0.021276596	GO:0005739	GO:CC	mitochondrion	TRUE
0.01735783	327	81	9	0.111111111	0.027522936	GO:0005740	GO:CC	mitochondrial envelope	FALSE
0.01735783	174	81	7	0.086419753	0.040229885	GO:0005743	GO:CC	mitochondrial inner membrane	FALSE
0.01735783	199	81	7	0.086419753	0.035175879	GO:0019866	GO:CC	organelle inner membrane	FALSE
0.01735783	300	81	9	0.111111111	0.03	GO:0031966	GO:CC	mitochondrial membrane	FALSE
0.01735783	5	81	2	0.024691358	0.4	GO:0170014	GO:CC	ankyrin-1 complex	TRUE
0.01735783	5580	81	54	0.666666667	0.009677419	GO:0005737	GO:CC	cytoplasm	FALSE
0.027555618	28	81	3	0.037037037	0.107142857	GO:0098803	GO:CC	respiratory chain complex	TRUE
0.008764783	96	81	6	0.074074074	0.0625	GO:0016829	GO:MF	lyase activity	FALSE
0.008764783	28	81	4	0.049382716	0.142857143	GO:0016830	GO:MF	carbon-carbon lyase activity	TRUE
0.009397845	3	81	2	0.024691358	0.666666667	GO:0098808	GO:MF	mRNA cap binding	TRUE

0.009397845	347	81	10	0.12345679	0.028818444	GO:0016491	GO:MF	oxidoreductase activity	TRUE
0.009397845	3	81	2	0.024691358	0.666666667	GO:0030492	GO:MF	hemoglobin binding	TRUE
0.018655413	20	81	3	0.037037037	0.15	GO:0016831	GO:MF	carboxy-lyase activity	FALSE
0.033109919	6	81	2	0.024691358	0.333333333	GO:0003810	GO:MF	protein-glutamine gamma-glutamyltransferase activity	TRUE
4.73E-05	23	81	5	0.061728395	0.217391304	KEGG:00860	KEGG	Porphyrin metabolism	FALSE
0.000151726	909	81	20	0.24691358	0.0220022	KEGG:01100	KEGG	Metabolic pathways	FALSE
0.01740773	54	81	4	0.049382716	0.074074074	KEGG:01232	KEGG	Nucleotide metabolism	FALSE
0.01740773	164	81	6	0.074074074	0.036585366	KEGG:05020	KEGG	Prion disease	FALSE
0.01740773	101	81	5	0.061728395	0.04950495	KEGG:01240	KEGG	Biosynthesis of cofactors	FALSE
0.01740773	163	81	6	0.074074074	0.036809816	KEGG:05012	KEGG	Parkinson disease	FALSE
0.022376092	33	81	3	0.037037037	0.090909091	KEGG:00983	KEGG	Drug metabolism - other enzymes	FALSE
0.022376092	9	81	2	0.024691358	0.222222222	KEGG:00591	KEGG	Linoleic acid metabolism	FALSE
0.024194616	194	81	6	0.074074074	0.030927835	KEGG:05016	KEGG	Huntington disease	FALSE
0.024194616	130	81	5	0.061728395	0.038461538	KEGG:05208	KEGG	Chemical carcinogenesis - reactive oxygen species	FALSE
0.024194616	39	81	3	0.037037037	0.076923077	KEGG:00240	KEGG	Pyrimidine metabolism	FALSE
0.046366743	229	81	6	0.074074074	0.026200873	KEGG:05010	KEGG	Alzheimer disease	FALSE
0.047260042	103	81	4	0.049382716	0.038834951	KEGG:04932	KEGG	Non-alcoholic fatty liver disease	FALSE

Turquoise Module GO Enrichment

p value	*term size*	*query size*	*overlap size*	*precision*	*recall*	*term id*	*source*	*term name*	*highlighted*
1.65E-15	4750	3538	1665	0.470604862	0.350526316	GO:0050794	GO:BP	regulation of cellular process	TRUE
8.44E-15	5049	3538	1750	0.494629734	0.346603288	GO:0065007	GO:BP	biological regulation	FALSE
8.44E-15	4906	3538	1706	0.48219333	0.347737464	GO:0050789	GO:BP	regulation of biological process	FALSE
2.25E-13	2561	3538	951	0.26879593	0.371339321	GO:0060255	GO:BP	regulation of macromolecule metabolic process	FALSE
1.65E-12	2304	3538	861	0.243357829	0.373697917	GO:0080090	GO:BP	regulation of primary metabolic process	FALSE
4.02E-12	130	3538	83	0.023459582	0.638461538	GO:0006399	GO:BP	tRNA metabolic process	TRUE
4.16E-12	2801	3538	1020	0.288298474	0.364155659	GO:0019222	GO:BP	regulation of metabolic process	FALSE
7.76E-12	1688	3538	651	0.184002261	0.385663507	GO:0019219	GO:BP	regulation of nucleobase-containing compound metabolic process	FALSE
1.04E-11	534	3538	245	0.069248163	0.458801498	GO:0006259	GO:BP	DNA metabolic process	FALSE
2.42E-11	2133	3538	796	0.224985868	0.37318331	GO:0010468	GO:BP	regulation of gene expression	FALSE
2.47E-11	172	3538	100	0.028264556	0.581395349	GO:0042254	GO:BP	ribosome biogenesis	FALSE
3.16E-11	2179	3538	810	0.228942906	0.371730151	GO:0010556	GO:BP	regulation of macromolecule biosynthetic process	FALSE
7.17E-11	2241	3538	828	0.234030526	0.369477912	GO:0009889	GO:BP	regulation of biosynthetic process	FALSE
2.40E-10	1517	3538	584	0.165065008	0.384970336	GO:0051252	GO:BP	regulation of RNA metabolic process	FALSE
2.56E-10	669	3538	289	0.081684568	0.431988042	GO:0022402	GO:BP	cell cycle process	FALSE
5.38E-10	96	3538	63	0.01780667	0.65625	GO:0006261	GO:BP	DNA-templated DNA replication	FALSE
6.85E-09	1747	3538	652	0.184284907	0.373211219	GO:0006996	GO:BP	organelle organization	FALSE
6.85E-09	512	3538	226	0.063877897	0.44140625	GO:0006974	GO:BP	DNA damage response	FALSE
6.85E-09	159	3538	89	0.025155455	0.559748428	GO:0006260	GO:BP	DNA replication	FALSE
1.21E-08	367	3538	171	0.048332391	0.465940054	GO:0010564	GO:BP	regulation of cell cycle process	FALSE

1.48E-08	1443	3538	548	0.154889768	0.37976438	GO:0006351	GO:BP	DNA-templated transcription	FALSE
1.50E-08	95	3538	60	0.016958734	0.631578947	GO:0009451	GO:BP	RNA modification	FALSE
1.53E-08	93	3538	59	0.016676088	0.634408602	GO:0008033	GO:BP	tRNA processing	FALSE
1.53E-08	833	3538	339	0.095816846	0.406962785	GO:0007049	GO:BP	cell cycle	FALSE
2.15E-08	118	3538	70	0.019785189	0.593220339	GO:0016072	GO:BP	rRNA metabolic process	FALSE
2.38E-08	96	3538	60	0.016958734	0.625	GO:0006364	GO:BP	rRNA processing	FALSE
8.75E-08	62	3538	43	0.012153759	0.693548387	GO:0006400	GO:BP	tRNA modification	FALSE
9.40E-08	1367	3538	517	0.146127756	0.378200439	GO:2001141	GO:BP	regulation of RNA biosynthetic process	FALSE
1.46E-07	224	3538	112	0.031656303	0.5	GO:1901987	GO:BP	regulation of cell cycle phase transition	FALSE
1.87E-07	1361	3538	513	0.144997174	0.376928729	GO:0006355	GO:BP	regulation of DNA-templated transcription	FALSE
2.85E-07	537	3538	228	0.064443188	0.424581006	GO:0051726	GO:BP	regulation of cell cycle	FALSE
3.36E-07	110	3538	64	0.018089316	0.581818182	GO:0000075	GO:BP	cell cycle checkpoint signaling	FALSE
3.97E-07	415	3538	183	0.051724138	0.440963855	GO:0006325	GO:BP	chromatin organization	TRUE
4.65E-07	335	3538	153	0.043244771	0.456716418	GO:0006281	GO:BP	DNA repair	FALSE
5.06E-07	280	3538	132	0.037309214	0.471428571	GO:0044770	GO:BP	cell cycle phase transition	FALSE
1.08E-06	398	3538	175	0.049462973	0.439698492	GO:1903047	GO:BP	mitotic cell cycle process	FALSE
1.22E-06	459	3538	197	0.055681176	0.4291939	GO:0000278	GO:BP	mitotic cell cycle	FALSE
2.93E-06	292	3538	134	0.037874505	0.45890411	GO:0051276	GO:BP	chromosome organization	FALSE
3.07E-06	156	3538	81	0.022894291	0.519230769	GO:0010948	GO:BP	negative regulation of cell cycle process	FALSE
9.87E-06	137	3538	72	0.02035048	0.525547445	GO:1901988	GO:BP	negative regulation of cell cycle phase transition	FALSE
1.45E-05	121	3538	65	0.018371962	0.537190083	GO:0000819	GO:BP	sister chromatid segregation	FALSE
1.95E-05	105	3538	58	0.016393443	0.552380952	GO:0032259	GO:BP	methylation	FALSE
2.20E-05	82	3538	48	0.013566987	0.585365854	GO:0007098	GO:BP	centrosome cycle	FALSE
2.53E-05	180	3538	88	0.024872809	0.488888889	GO:1901990	GO:BP	regulation of mitotic cell cycle phase transition	FALSE
3.10E-05	111	3538	60	0.016958734	0.540540541	GO:0000070	GO:BP	mitotic sister chromatid segregation	FALSE
3.16E-05	2284	3538	797	0.225268513	0.348949212	GO:0048523	GO:BP	negative regulation of cellular process	FALSE
3.20E-05	233	3538	108	0.030525721	0.463519313	GO:0044772	GO:BP	mitotic cell cycle phase transition	FALSE
3.23E-05	480	3538	198	0.055963821	0.4125	GO:0007017	GO:BP	microtubule-based process	FALSE
4.54E-05	86	3538	49	0.013849633	0.569767442	GO:0031023	GO:BP	microtubule organizing center organization	FALSE
4.58E-05	38	3538	27	0.00763143	0.710526316	GO:0051784	GO:BP	negative regulation of nuclear division	FALSE
4.58E-05	36	3538	26	0.007348785	0.722222222	GO:0045839	GO:BP	negative regulation of mitotic nuclear division	FALSE
4.58E-05	248	3538	113	0.031938949	0.455645161	GO:0007346	GO:BP	regulation of mitotic cell cycle	FALSE
4.72E-05	152	3538	76	0.021481063	0.5	GO:0140014	GO:BP	mitotic nuclear division	FALSE
5.62E-05	140	3538	71	0.020067835	0.507142857	GO:0033044	GO:BP	regulation of chromosome organization	FALSE
7.70E-05	2347	3538	813	0.229790842	0.346399659	GO:0048519	GO:BP	negative regulation of biological process	FALSE
8.28E-05	224	3538	103	0.029112493	0.459821429	GO:0007059	GO:BP	chromosome segregation	FALSE
8.55E-05	349	3538	149	0.042114189	0.426934097	GO:0006338	GO:BP	chromatin remodeling	FALSE
0.00013553	61	3538	37	0.010457886	0.606557377	GO:0140053	GO:BP	mitochondrial gene expression	TRUE
0.000154619	187	3538	88	0.024872809	0.470588235	GO:0045786	GO:BP	negative regulation of cell cycle	FALSE

0.000348914	56	3538	34	0.009609949	0.607142857	GO:2001251	GO:BP	negative regulation of chromosome organization	FALSE
0.000392141	54	3538	33	0.009327304	0.611111111	GO:0051028	GO:BP	mRNA transport	TRUE
0.000414944	956	3538	354	0.100056529	0.370292887	GO:0043412	GO:BP	macromolecule modification	FALSE
0.000437752	82	3538	45	0.01271905	0.548780488	GO:0007093	GO:BP	mitotic cell cycle checkpoint signaling	FALSE
0.000441732	37	3538	25	0.007066139	0.675675676	GO:0033047	GO:BP	regulation of mitotic sister chromatid segregation	FALSE
0.000442802	29	3538	21	0.005935557	0.724137931	GO:0071173	GO:BP	spindle assembly checkpoint signaling	FALSE
0.000442802	29	3538	21	0.005935557	0.724137931	GO:0071174	GO:BP	mitotic spindle checkpoint signaling	FALSE
0.000442802	31	3538	22	0.006218202	0.709677419	GO:0045841	GO:BP	negative regulation of mitotic metaphase/anaphase transition	FALSE
0.000442802	29	3538	21	0.005935557	0.724137931	GO:0007094	GO:BP	mitotic spindle assembly checkpoint signaling	FALSE
0.000442802	31	3538	22	0.006218202	0.709677419	GO:0033048	GO:BP	negative regulation of mitotic sister chromatid segregation	FALSE
0.000442802	31	3538	22	0.006218202	0.709677419	GO:0033046	GO:BP	negative regulation of sister chromatid segregation	FALSE
0.000442802	31	3538	22	0.006218202	0.709677419	GO:2000816	GO:BP	negative regulation of mitotic sister chromatid separation	FALSE
0.00075805	191	3538	87	0.024590164	0.455497382	GO:0006310	GO:BP	DNA recombination	FALSE
0.000783375	38	3538	25	0.007066139	0.657894737	GO:0043414	GO:BP	macromolecule methylation	FALSE
0.000783375	170	3538	79	0.022328999	0.464705882	GO:0098813	GO:BP	nuclear chromosome segregation	FALSE
0.000783375	38	3538	25	0.007066139	0.657894737	GO:0001510	GO:BP	RNA methylation	FALSE
0.000862453	224	3538	99	0.027981911	0.441964286	GO:0000280	GO:BP	nuclear division	FALSE
0.000882513	1247	3538	447	0.126342566	0.358460305	GO:0009892	GO:BP	negative regulation of metabolic process	FALSE
0.000895611	32	3538	22	0.006218202	0.6875	GO:1905819	GO:BP	negative regulation of chromosome separation	FALSE
0.000895611	32	3538	22	0.006218202	0.6875	GO:0051985	GO:BP	negative regulation of chromosome segregation	FALSE
0.000895611	32	3538	22	0.006218202	0.6875	GO:1902100	GO:BP	negative regulation of metaphase/anaphase transition of cell cycle	FALSE
0.000900877	338	3538	140	0.039570379	0.414201183	GO:0000226	GO:BP	microtubule cytoskeleton organization	FALSE
0.000900877	30	3538	21	0.005935557	0.7	GO:0007099	GO:BP	centriole replication	FALSE
0.000900877	30	3538	21	0.005935557	0.7	GO:0031577	GO:BP	spindle checkpoint signaling	FALSE
0.00100527	75	3538	41	0.011588468	0.546666667	GO:0031570	GO:BP	DNA integrity checkpoint signaling	FALSE
0.001094861	43	3538	27	0.00763143	0.627906977	GO:0030071	GO:BP	regulation of mitotic metaphase/anaphase transition	FALSE
0.001103257	1156	3538	416	0.117580554	0.359861592	GO:0010605	GO:BP	negative regulation of macromolecule metabolic process	FALSE
0.001120594	85	3538	45	0.01271905	0.529411765	GO:0051168	GO:BP	nuclear export	FALSE
0.001288136	50	3538	30	0.008479367	0.6	GO:0051304	GO:BP	chromosome separation	FALSE
0.001449852	76	3538	41	0.011588468	0.539473684	GO:0051236	GO:BP	establishment of RNA localization	FALSE
0.001449852	48	3538	29	0.008196721	0.604166667	GO:0051298	GO:BP	centrosome duplication	FALSE

0.001469853	678	3538	256	0.072357264	0.377581121	GO:0045934	GO:BP	negative regulation of nucleobase-containing compound metabolic process	FALSE
0.00160999	187	3538	84	0.023742227	0.449197861	GO:0006302	GO:BP	double-strand break repair	FALSE
0.00160999	46	3538	28	0.007914076	0.608695652	GO:0007091	GO:BP	metaphase/anaphase transition of mitotic cell cycle	FALSE
0.001629152	33	3538	22	0.006218202	0.666666667	GO:0032790	GO:BP	ribosome disassembly	FALSE
0.001718109	79	3538	42	0.011871114	0.53164557	GO:0045814	GO:BP	negative regulation of gene expression, epigenetic	TRUE
0.001718109	31	3538	21	0.005935557	0.677419355	GO:0098534	GO:BP	centriole assembly	FALSE
0.001730734	53	3538	31	0.008762012	0.58490566	GO:0033045	GO:BP	regulation of sister chromatid segregation	FALSE
0.001745544	29	3538	20	0.005652911	0.689655172	GO:0006334	GO:BP	nucleosome assembly	FALSE
0.001745544	44	3538	27	0.00763143	0.613636364	GO:1902099	GO:BP	regulation of metaphase/anaphase transition of cell cycle	FALSE
0.001800129	25	3538	18	0.00508762	0.72	GO:1901976	GO:BP	regulation of cell cycle checkpoint	FALSE
0.001889743	987	3538	358	0.101187111	0.362715299	GO:0006357	GO:BP	regulation of transcription by RNA polymerase II	FALSE
0.002132739	257	3538	109	0.030808366	0.424124514	GO:0048285	GO:BP	organelle fission	FALSE
0.002144011	40	3538	25	0.007066139	0.625	GO:0051306	GO:BP	mitotic sister chromatid separation	FALSE
0.002291524	56	3538	32	0.009044658	0.571428571	GO:0031123	GO:BP	RNA 3'-end processing	TRUE
0.002369617	38	3538	24	0.006783493	0.631578947	GO:0010965	GO:BP	regulation of mitotic sister chromatid separation	FALSE
0.002482855	47	3538	28	0.007914076	0.595744681	GO:0044784	GO:BP	metaphase/anaphase transition of cell cycle	FALSE
0.002582673	73	3538	39	0.011023177	0.534246575	GO:0050657	GO:BP	nucleic acid transport	FALSE
0.002582673	73	3538	39	0.011023177	0.534246575	GO:0050658	GO:BP	RNA transport	FALSE
0.002786101	66	3538	36	0.01017524	0.545454545	GO:0051983	GO:BP	regulation of chromosome segregation	FALSE
0.002786101	34	3538	22	0.006218202	0.647058824	GO:0045005	GO:BP	DNA-templated DNA replication maintenance of fidelity	FALSE
0.002786101	98	3538	49	0.013849633	0.5	GO:1901991	GO:BP	negative regulation of mitotic cell cycle phase transition	FALSE
0.002966734	71	3538	38	0.010740531	0.535211268	GO:0000077	GO:BP	DNA damage checkpoint signaling	FALSE
0.003010803	32	3538	21	0.005935557	0.65625	GO:0031297	GO:BP	replication fork processing	FALSE
0.003068636	1051	3538	377	0.106557377	0.358705994	GO:0006366	GO:BP	transcription by RNA polymerase II	FALSE
0.003194621	91	3538	46	0.013001696	0.505494505	GO:0006403	GO:BP	RNA localization	FALSE
0.003324459	57	3538	32	0.009044658	0.561403509	GO:0007088	GO:BP	regulation of mitotic nuclear division	FALSE
0.003577401	368	3538	147	0.041548898	0.399456522	GO:0006412	GO:BP	translation	FALSE
0.004403677	958	3538	345	0.097512719	0.360125261	GO:0033554	GO:BP	cellular response to stress	FALSE
0.004403677	46	3538	27	0.00763143	0.586956522	GO:1905818	GO:BP	regulation of chromosome separation	FALSE
0.004403677	37	3538	23	0.006500848	0.621621622	GO:0032543	GO:BP	mitochondrial translation	FALSE
0.004943208	226	3538	96	0.027133974	0.424778761	GO:0051301	GO:BP	cell division	TRUE
0.005131511	229	3538	97	0.02741662	0.423580786	GO:0007005	GO:BP	mitochondrion organization	FALSE
0.005324257	1009	3538	361	0.102035048	0.35777998	GO:0009890	GO:BP	negative regulation of biosynthetic process	FALSE

0.005728999	68	3538	36	0.01017524	0.529411765	GO:0006275	GO:BP	regulation of DNA replication	FALSE
0.005897716	73	3538	38	0.010740531	0.520547945	GO:0065004	GO:BP	protein-DNA complex assembly	FALSE
0.005905943	986	3538	353	0.099773884	0.35801217	GO:0010558	GO:BP	negative regulation of macromolecule biosynthetic process	FALSE
0.006267719	61	3538	33	0.009327304	0.540983607	GO:0031507	GO:BP	heterochromatin formation	FALSE
0.006313244	159	3538	71	0.020067835	0.446540881	GO:0045787	GO:BP	positive regulation of cell cycle	FALSE
0.006573275	66	3538	35	0.009892595	0.53030303	GO:0051783	GO:BP	regulation of nuclear division	FALSE
0.006646747	122	3538	57	0.016110797	0.467213115	GO:0045930	GO:BP	negative regulation of mitotic cell cycle	FALSE
0.006720175	71	3538	37	0.010457886	0.521126761	GO:0045727	GO:BP	positive regulation of translation	FALSE
0.006720175	130	3538	60	0.016958734	0.461538462	GO:0090068	GO:BP	positive regulation of cell cycle process	FALSE
0.009901273	10	3538	9	0.00254381	0.9	GO:0006999	GO:BP	nuclear pore organization	TRUE
0.010395397	250	3538	103	0.029112493	0.412	GO:0051052	GO:BP	regulation of DNA metabolic process	FALSE
0.011584681	181	3538	78	0.022046354	0.430939227	GO:0006913	GO:BP	nucleocytoplasmic transport	FALSE
0.011584681	181	3538	78	0.022046354	0.430939227	GO:0051169	GO:BP	nuclear transport	FALSE
0.012086717	173	3538	75	0.021198417	0.433526012	GO:0006401	GO:BP	RNA catabolic process	TRUE
0.012122535	106	3538	50	0.014132278	0.471698113	GO:0042770	GO:BP	signal transduction in response to DNA damage	FALSE
0.012236653	223	3538	93	0.026286037	0.417040359	GO:0140694	GO:BP	membraneless organelle assembly	FALSE
0.012340027	88	3538	43	0.012153759	0.488636364	GO:0071824	GO:BP	protein-DNA complex organization	FALSE
0.013815705	2729	3538	905	0.255794234	0.331623305	GO:0048518	GO:BP	positive regulation of biological process	FALSE
0.013816727	7	3538	7	0.001978519	1	GO:0060623	GO:BP	regulation of chromosome condensation	FALSE
0.013905141	44	3538	25	0.007066139	0.568181818	GO:0034728	GO:BP	nucleosome organization	FALSE
0.015244513	71	3538	36	0.01017524	0.507042254	GO:0051053	GO:BP	negative regulation of DNA metabolic process	FALSE
0.015244513	35	3538	21	0.005935557	0.6	GO:0043038	GO:BP	amino acid activation	FALSE
0.015244513	35	3538	21	0.005935557	0.6	GO:0072698	GO:BP	protein localization to microtubule cytoskeleton	TRUE
0.015404248	14	3538	11	0.003109101	0.785714286	GO:0090266	GO:BP	regulation of mitotic cell cycle spindle assembly checkpoint	FALSE
0.015404248	14	3538	11	0.003109101	0.785714286	GO:0090231	GO:BP	regulation of spindle checkpoint	FALSE
0.015404248	14	3538	11	0.003109101	0.785714286	GO:1903504	GO:BP	regulation of mitotic spindle checkpoint	FALSE
0.016731821	186	3538	79	0.022328999	0.424731183	GO:0141188	GO:BP	nucleic acid catabolic process	FALSE
0.017405954	285	3538	114	0.032221594	0.4	GO:0010608	GO:BP	post-transcriptional regulation of gene expression	FALSE
0.017535264	531	3538	198	0.055963821	0.372881356	GO:0043687	GO:BP	post-translational protein modification	TRUE
0.019382555	31	3538	19	0.005370266	0.612903226	GO:0000959	GO:BP	mitochondrial RNA metabolic process	TRUE
0.022005069	85	3538	41	0.011588468	0.482352941	GO:0044839	GO:BP	cell cycle G2/M phase transition	FALSE

0.023976339	522	3538	194	0.054833239	0.37164751	GO:0070925	GO:BP	organelle assembly	FALSE
0.024158643	36	3538	21	0.005935557	0.583333333	GO:0006406	GO:BP	mRNA export from nucleus	FALSE
0.024158643	70	3538	35	0.009892595	0.5	GO:2001252	GO:BP	positive regulation of chromosome organization	FALSE
0.024158643	36	3538	21	0.005935557	0.583333333	GO:0044380	GO:BP	protein localization to cytoskeleton	FALSE
0.024638507	9	3538	8	0.002261164	0.888888889	GO:0000012	GO:BP	single strand break repair	FALSE
0.025248725	96	3538	45	0.01271905	0.46875	GO:0000082	GO:BP	G1/S transition of mitotic cell cycle	FALSE
0.026141112	607	3538	222	0.062747315	0.365733114	GO:0051253	GO:BP	negative regulation of RNA metabolic process	FALSE
0.026211492	78	3538	38	0.010740531	0.487179487	GO:0000086	GO:BP	G2/M transition of mitotic cell cycle	FALSE
0.027436794	34	3538	20	0.005652911	0.588235294	GO:0043039	GO:BP	tRNA aminoacylation	FALSE
0.029549387	81	3538	39	0.011023177	0.481481481	GO:0034502	GO:BP	protein localization to chromosome	TRUE
0.029549387	23	3538	15	0.004239683	0.652173913	GO:0071539	GO:BP	protein localization to centrosome	TRUE
0.02979753	2590	3538	856	0.241944601	0.330501931	GO:0048522	GO:BP	positive regulation of cellular process	FALSE
0.029823897	46	3538	25	0.007066139	0.543478261	GO:0006405	GO:BP	RNA export from nucleus	FALSE
0.031131872	51	3538	27	0.00763143	0.529411765	GO:1903008	GO:BP	organelle disassembly	FALSE
0.031131872	11	3538	9	0.00254381	0.818181818	GO:2000232	GO:BP	regulation of rRNA processing	FALSE
0.031131872	11	3538	9	0.00254381	0.818181818	GO:1990166	GO:BP	protein localization to site of double-strand break	FALSE
0.031671171	105	3538	48	0.013566987	0.457142857	GO:0044843	GO:BP	cell cycle G1/S phase transition	FALSE
0.033797488	100	3538	46	0.013001696	0.46	GO:0000723	GO:BP	telomere maintenance	FALSE
0.033797488	100	3538	46	0.013001696	0.46	GO:0032200	GO:BP	telomere organization	FALSE
0.033797488	116	3538	52	0.014697569	0.448275862	GO:0000725	GO:BP	recombinational repair	FALSE
0.034067529	146	3538	63	0.01780667	0.431506849	GO:0006402	GO:BP	mRNA catabolic process	FALSE
0.034814183	17	3538	12	0.003391747	0.705882353	GO:0071459	GO:BP	protein localization to chromosome, centromeric region	FALSE
0.03625324	6	3538	6	0.001695873	1	GO:0006307	GO:BP	DNA alkylation repair	FALSE
0.03625324	6	3538	6	0.001695873	1	GO:2000234	GO:BP	positive regulation of rRNA processing	FALSE
0.039940451	504	3538	186	0.052572075	0.369047619	GO:0070647	GO:BP	protein modification by small protein conjugation or removal	FALSE
0.040646496	936	3538	328	0.092707744	0.35042735	GO:0045935	GO:BP	positive regulation of nucleobase-containing compound metabolic process	FALSE
0.043662123	57	3538	29	0.008196721	0.50877193	GO:0042274	GO:BP	ribosomal small subunit biogenesis	FALSE
0.043682497	553	3538	202	0.057094404	0.365280289	GO:1902679	GO:BP	negative regulation of RNA biosynthetic process	FALSE
0.044553102	26	3538	16	0.004522329	0.615384615	GO:0072344	GO:BP	rescue of stalled ribosome	FALSE
0.04456346	112	3538	50	0.014132278	0.446428571	GO:0015931	GO:BP	nucleobase-containing compound transport	FALSE
0.047316916	70	3538	34	0.009609949	0.485714286	GO:1902749	GO:BP	regulation of cell cycle G2/M phase transition	FALSE
0.047368045	115	3538	51	0.014414924	0.443478261	GO:0000724	GO:BP	double-strand break repair via homologous recombination	FALSE
4.42E-33	2745	3538	1102	0.31147541	0.401457195	GO:0043233	GO:CC	organelle lumen	FALSE

4.42E-33	2745	3538	1102	0.31147541	0.401457195	GO:0070013	GO:CC	intracellular organelle lumen	TRUE
4.42E-33	2745	3538	1102	0.31147541	0.401457195	GO:0031974	GO:CC	membrane-enclosed lumen	FALSE
2.26E-29	2518	3538	1010	0.285472018	0.401111994	GO:0031981	GO:CC	nuclear lumen	FALSE
4.63E-27	2579	3538	1020	0.288298474	0.395502133	GO:0043228	GO:CC	membraneless organelle	FALSE
4.63E-27	2579	3538	1020	0.288298474	0.395502133	GO:0043232	GO:CC	intracellular membraneless organelle	FALSE
2.33E-23	2171	3538	866	0.244771057	0.398894519	GO:0005654	GO:CC	nucleoplasm	FALSE
1.91E-18	5580	3538	1928	0.544940644	0.345519713	GO:0005737	GO:CC	cytoplasm	FALSE
3.52E-14	656	3538	296	0.083663086	0.451219512	GO:0005730	GO:CC	nucleolus	FALSE
7.45E-14	763	3538	334	0.094403618	0.43774574	GO:0005694	GO:CC	chromosome	FALSE
7.19E-12	846	3538	356	0.10062182	0.420803783	GO:0005739	GO:CC	mitochondrion	FALSE
1.30E-10	488	3538	221	0.062464669	0.452868852	GO:0005815	GO:CC	microtubule organizing center	FALSE
3.16E-10	433	3538	199	0.056246467	0.459584296	GO:0005813	GO:CC	centrosome	FALSE
1.10E-09	755	3538	314	0.088750707	0.41589404	GO:0015630	GO:CC	microtubule cytoskeleton	FALSE
3.53E-09	541	3538	235	0.066421707	0.434380776	GO:1990234	GO:CC	transferase complex	TRUE
1.35E-08	1035	3538	406	0.114754098	0.392270531	GO:1902494	GO:CC	catalytic complex	FALSE
2.00E-08	611	3538	257	0.07263991	0.420621931	GO:0140535	GO:CC	intracellular protein-containing complex	FALSE
3.90E-06	208	3538	100	0.028264556	0.480769231	GO:0098687	GO:CC	chromosomal region	FALSE
4.12E-06	160	3538	81	0.022894291	0.50625	GO:0000793	GO:CC	condensed chromosome	FALSE
8.01E-06	2131	3538	749	0.211701526	0.351478179	GO:0005829	GO:CC	cytosol	FALSE
1.88E-05	84	3538	48	0.013566987	0.571428571	GO:0005814	GO:CC	centriole	FALSE
3.46E-05	15	3538	14	0.003957038	0.933333333	GO:0019908	GO:CC	nuclear cyclin-dependent protein kinase holoenzyme complex	FALSE
3.57E-05	20	3538	17	0.004804975	0.85	GO:1990391	GO:CC	DNA repair complex	TRUE
4.14E-05	1139	3538	419	0.118428491	0.36786655	GO:0005856	GO:CC	cytoskeleton	FALSE
4.44E-05	135	3538	68	0.019219898	0.503703704	GO:0000228	GO:CC	nuclear chromosome	FALSE
5.66E-05	525	3538	210	0.059355568	0.4	GO:0016604	GO:CC	nuclear body	FALSE
5.74E-05	44	3538	29	0.008196721	0.659090909	GO:0005643	GO:CC	nuclear pore	FALSE
5.90E-05	172	3538	82	0.023176936	0.476744186	GO:0061695	GO:CC	transferase complex, transferring phosphorus-containing groups	FALSE
0.000106256	65	3538	38	0.010740531	0.584615385	GO:0030880	GO:CC	RNA polymerase complex	FALSE
0.000115221	96	3538	51	0.014414924	0.53125	GO:0098798	GO:CC	mitochondrial protein-containing complex	FALSE
0.000115534	63	3538	37	0.010457886	0.587301587	GO:0055029	GO:CC	nuclear DNA-directed RNA polymerase complex	FALSE
0.000156043	559	3538	219	0.061899378	0.39177102	GO:0031967	GO:CC	organelle envelope	FALSE
0.000182161	64	3538	37	0.010457886	0.578125	GO:0000428	GO:CC	DNA-directed RNA polymerase complex	FALSE
0.00021159	13	3538	12	0.003391747	0.923076923	GO:0032806	GO:CC	carboxy-terminal domain protein kinase complex	FALSE
0.000315781	171	3538	79	0.022328999	0.461988304	GO:0005759	GO:CC	mitochondrial matrix	FALSE
0.000315781	56	3538	33	0.009327304	0.589285714	GO:0030684	GO:CC	preribosome	TRUE
0.000356758	97	3538	50	0.014132278	0.515463918	GO:0000776	GO:CC	kinetochore	FALSE
0.000714683	140	3538	66	0.018654607	0.471428571	GO:0000775	GO:CC	chromosome, centromeric region	FALSE
0.001155316	103	3538	51	0.014414924	0.495145631	GO:0000779	GO:CC	condensed chromosome, centromeric region	FALSE
0.001452805	50	3538	29	0.008196721	0.58	GO:0005657	GO:CC	replication fork	FALSE
0.001618254	11	3538	10	0.002826456	0.909090909	GO:0031261	GO:CC	DNA replication preinitiation complex	FALSE
0.001859145	77	3538	40	0.011305822	0.519480519	GO:0000781	GO:CC	chromosome, telomeric region	FALSE
0.002005253	8	3538	8	0.002261164	1	GO:0031080	GO:CC	nuclear pore outer ring	FALSE

0.002005253	8	3538	8	0.002261164	1	GO:0000439	GO:CC	transcription factor TFIIH core complex	TRUE
0.002005253	8	3538	8	0.002261164	1	GO:0005675	GO:CC	transcription factor TFIIH holo complex	FALSE
0.00242484	73	3538	38	0.010740531	0.520547945	GO:1902554	GO:CC	serine/threonine protein kinase complex	FALSE
0.003505746	36	3538	22	0.006218202	0.611111111	GO:0000307	GO:CC	cyclin-dependent protein kinase holoenzyme complex	FALSE
0.00415096	10	3538	9	0.00254381	0.9	GO:0071162	GO:CC	CMG complex	FALSE
0.004608651	85	3538	42	0.011871114	0.494117647	GO:0090734	GO:CC	site of DNA damage	FALSE
0.004621798	80	3538	40	0.011305822	0.5	GO:0034451	GO:CC	centriolar satellite	FALSE
0.005300561	83	3538	41	0.011588468	0.493975904	GO:1902911	GO:CC	protein kinase complex	FALSE
0.010145704	106	3538	49	0.013849633	0.462264151	GO:0001650	GO:CC	fibrillar center	FALSE
0.011183629	9	3538	8	0.002261164	0.888888889	GO:0042555	GO:CC	MCM complex	TRUE
0.012868692	86	3538	41	0.011588468	0.476744186	GO:0036064	GO:CC	ciliary basal body	FALSE
0.01411642	327	3538	127	0.035895986	0.388379205	GO:0005740	GO:CC	mitochondrial envelope	FALSE
0.014393565	39	3538	22	0.006218202	0.564102564	GO:0032040	GO:CC	small-subunit processome	FALSE
0.016833947	6	3538	6	0.001695873	1	GO:0005958	GO:CC	DNA-dependent protein kinase-DNA ligase 4 complex	FALSE
0.016833947	6	3538	6	0.001695873	1	GO:0097504	GO:CC	Gemini of Cajal bodies	FALSE
0.017642125	300	3538	117	0.033069531	0.39	GO:0031966	GO:CC	mitochondrial membrane	FALSE
0.018537489	67	3538	33	0.009327304	0.492537313	GO:0034708	GO:CC	methyltransferase complex	FALSE
0.018537489	240	3538	96	0.027133974	0.4	GO:0005635	GO:CC	nuclear envelope	FALSE
0.018537489	35	3538	20	0.005652911	0.571428571	GO:0015030	GO:CC	Cajal body	FALSE
0.019320783	229	3538	92	0.026003392	0.401746725	GO:0005819	GO:CC	spindle	FALSE
0.026411588	8	3538	7	0.001978519	0.875	GO:0070419	GO:CC	nonhomologous end joining complex	FALSE
0.026411588	8	3538	7	0.001978519	0.875	GO:0044615	GO:CC	nuclear pore nuclear basket	FALSE
0.026411588	8	3538	7	0.001978519	0.875	GO:0000796	GO:CC	condensin complex	FALSE
0.027168139	36	3538	20	0.005652911	0.555555556	GO:0031519	GO:CC	PcG protein complex	TRUE
0.028976877	192	3538	78	0.022046354	0.40625	GO:0000151	GO:CC	ubiquitin ligase complex	FALSE
0.029148158	18	3538	12	0.003391747	0.666666667	GO:0000178	GO:CC	exosome (RNase complex)	TRUE
0.031465432	142	3538	60	0.016958734	0.422535211	GO:0005681	GO:CC	spliceosomal complex	TRUE
0.032273336	12	3538	9	0.00254381	0.75	GO:0005666	GO:CC	RNA polymerase III complex	FALSE
0.032273336	12	3538	9	0.00254381	0.75	GO:0000940	GO:CC	outer kinetochore	FALSE
0.032712337	572	3538	206	0.058224986	0.36013986	GO:0099080	GO:CC	supramolecular complex	FALSE
0.040105921	23	3538	14	0.003957038	0.608695652	GO:1905354	GO:CC	exoribonuclease complex	FALSE
0.040990687	30	3538	17	0.004804975	0.566666667	GO:0098800	GO:CC	inner mitochondrial membrane protein complex	FALSE
0.041697589	5	3538	5	0.001413228	1	GO:0030688	GO:CC	preribosome, small subunit precursor	FALSE
0.041697589	5	3538	5	0.001413228	1	GO:0070533	GO:CC	BRCA1-C complex	TRUE
2.46E-27	414	3538	237	0.066986998	0.572463768	GO:0140640	GO:MF	catalytic activity, acting on a nucleic acid	TRUE
4.66E-19	3652	3538	1335	0.377331826	0.365553122	GO:0003824	GO:MF	catalytic activity	FALSE
3.41E-18	2844	3538	1068	0.301865461	0.375527426	GO:0043167	GO:MF	ion binding	TRUE
6.58E-17	2938	3538	1091	0.308366309	0.371341048	GO:0036094	GO:MF	small molecule binding	FALSE
7.24E-16	240	3538	138	0.039005088	0.575	GO:0140098	GO:MF	catalytic activity, acting on RNA	FALSE
5.19E-14	984	3538	416	0.117580554	0.422764228	GO:0003677	GO:MF	DNA binding	TRUE
1.86E-11	1735	3538	662	0.187111362	0.381556196	GO:0043169	GO:MF	cation binding	FALSE
7.28E-11	1702	3538	647	0.182871679	0.380141011	GO:0046872	GO:MF	metal ion binding	FALSE
7.28E-11	403	3538	191	0.053985302	0.473945409	GO:0140657	GO:MF	ATP-dependent activity	FALSE
1.99E-10	174	3538	98	0.027699265	0.563218391	GO:0140097	GO:MF	catalytic activity, acting on DNA	FALSE

4.70E-10	95	3538	62	0.017524025	0.652631579	GO:0008094	GO:MF	ATP-dependent activity, acting on DNA	FALSE
9.37E-10	94	3538	61	0.017241379	0.64893617	GO:0140101	GO:MF	catalytic activity, acting on a tRNA	FALSE
3.60E-09	1388	3538	531	0.150084794	0.382564841	GO:1901265	GO:MF	nucleoside phosphate binding	FALSE
6.11E-09	1446	3538	549	0.155172414	0.37966805	GO:1901363	GO:MF	heterocyclic compound binding	FALSE
6.87E-09	1376	3538	525	0.14838892	0.381540698	GO:0000166	GO:MF	nucleotide binding	FALSE
4.27E-08	105	3538	63	0.01780667	0.6	GO:0004386	GO:MF	helicase activity	FALSE
6.32E-08	1229	3538	469	0.132560769	0.381611066	GO:0032553	GO:MF	ribonucleotide binding	FALSE
6.32E-08	1222	3538	467	0.131995478	0.382160393	GO:0032555	GO:MF	purine ribonucleotide binding	FALSE
6.32E-08	115	3538	67	0.018937253	0.582608696	GO:0120545	GO:MF	nucleic acid conformation isomerase activity	FALSE
6.32E-08	172	3538	91	0.025720746	0.529069767	GO:0120543	GO:MF	macromolecular conformation isomerase activity	FALSE
6.32E-08	141	3538	78	0.022046354	0.553191489	GO:0016741	GO:MF	transferase activity, transferring one-carbon groups	FALSE
6.32E-08	134	3538	75	0.021198417	0.559701493	GO:0008168	GO:MF	methyltransferase activity	FALSE
9.37E-08	1197	3538	457	0.129169022	0.381787803	GO:0035639	GO:MF	purine ribonucleoside triphosphate binding	FALSE
9.94E-08	1272	3538	482	0.136235161	0.378930818	GO:0017076	GO:MF	purine nucleotide binding	FALSE
1.76E-07	888	3538	350	0.098925947	0.394144144	GO:0032559	GO:MF	adenyl ribonucleotide binding	FALSE
1.86E-07	132	3538	73	0.020633126	0.553030303	GO:0042393	GO:MF	histone binding	TRUE
3.58E-07	876	3538	344	0.097230073	0.392694064	GO:0005524	GO:MF	ATP binding	FALSE
3.58E-07	246	3538	118	0.033352176	0.479674797	GO:0016853	GO:MF	isomerase activity	FALSE
4.15E-07	1456	3538	539	0.152345958	0.370192308	GO:0043168	GO:MF	anion binding	FALSE
4.89E-07	937	3538	364	0.102882985	0.388473853	GO:0030554	GO:MF	adenyl nucleotide binding	FALSE
7.76E-07	1453	3538	536	0.151498021	0.368891948	GO:0016740	GO:MF	transferase activity	FALSE
1.22E-06	15	3538	15	0.004239683	1	GO:0017056	GO:MF	structural constituent of nuclear pore	TRUE
1.73E-06	1337	3538	495	0.139909553	0.370231862	GO:0097367	GO:MF	carbohydrate derivative binding	FALSE
1.28E-05	194	3538	93	0.026286037	0.479381443	GO:0015631	GO:MF	tubulin binding	TRUE
1.98E-05	110	3538	59	0.016676088	0.536363636	GO:0008757	GO:MF	S-adenosylmethionine-dependent methyltransferase activity	FALSE
3.97E-05	41	3538	28	0.007914076	0.682926829	GO:0003678	GO:MF	DNA helicase activity	FALSE
8.21E-05	42	3538	28	0.007914076	0.666666667	GO:0008173	GO:MF	RNA methyltransferase activity	FALSE
8.35E-05	1657	3538	587	0.165912945	0.354254677	GO:0016787	GO:MF	hydrolase activity	FALSE
0.000312045	532	3538	209	0.059072923	0.392857143	GO:0046914	GO:MF	transition metal ion binding	FALSE
0.000335316	42	3538	27	0.00763143	0.642857143	GO:0000049	GO:MF	tRNA binding	TRUE
0.000389871	413	3538	167	0.047201809	0.404358354	GO:0008270	GO:MF	zinc ion binding	FALSE
0.000461039	142	3538	68	0.019219898	0.478873239	GO:0008017	GO:MF	microtubule binding	FALSE
0.000964412	179	3538	81	0.022894291	0.452513966	GO:0016887	GO:MF	ATP hydrolysis activity	FALSE
0.001002264	544	3538	210	0.059355568	0.386029412	GO:0003690	GO:MF	double-stranded DNA binding	FALSE
0.001195573	117	3538	57	0.016110797	0.487179487	GO:0004518	GO:MF	nuclease activity	FALSE
0.001323306	3253	3538	1081	0.305539853	0.332308638	GO:0005515	GO:MF	protein binding	FALSE
0.00150827	648	3538	244	0.068965517	0.37654321	GO:0016818	GO:MF	hydrolase activity, acting on acid anhydrides, in phosphorus-containing anhydrides	FALSE

0.00150827	648	3538	244	0.068965517	0.37654321	GO:0016817	GO:MF	hydrolase activity, acting on acid anhydrides	FALSE
0.001826693	644	3538	242	0.068400226	0.375776398	GO:0016462	GO:MF	pyrophosphatase activity	FALSE
0.001826693	567	3538	216	0.061051441	0.380952381	GO:0043565	GO:MF	sequence-specific DNA binding	FALSE
0.001865314	106	3538	52	0.014697569	0.490566038	GO:0043021	GO:MF	ribonucleoprotein complex binding	TRUE
0.003088059	498	3538	191	0.053985302	0.383534137	GO:1990837	GO:MF	sequence-specific double-stranded DNA binding	FALSE
0.003259608	134	3538	62	0.017524025	0.462686567	GO:0140993	GO:MF	histone modifying activity	TRUE
0.003710751	615	3538	230	0.065008479	0.37398374	GO:0017111	GO:MF	ribonucleoside triphosphate phosphatase activity	FALSE
0.004116134	29	3538	19	0.005370266	0.655172414	GO:0140658	GO:MF	ATP-dependent chromatin remodeler activity	FALSE
0.010250413	452	3538	172	0.048615037	0.380530973	GO:0000976	GO:MF	transcription cis-regulatory region binding	FALSE
0.011430257	453	3538	172	0.048615037	0.379690949	GO:0001067	GO:MF	transcription regulatory region nucleic acid binding	FALSE
0.011674955	110	3538	51	0.014414924	0.463636364	GO:0016874	GO:MF	ligase activity	FALSE
0.017262307	383	3538	147	0.041548898	0.38381201	GO:0000977	GO:MF	RNA polymerase II transcription regulatory region sequence-specific DNA binding	FALSE
0.017366453	43	3538	24	0.006783493	0.558139535	GO:0140034	GO:MF	methylation-dependent protein binding	FALSE
0.017448312	398	3538	152	0.042962125	0.381909548	GO:0003700	GO:MF	DNA-binding transcription factor activity	TRUE
0.018189366	664	3538	241	0.068117581	0.362951807	GO:0140110	GO:MF	transcription regulator activity	FALSE
0.019022667	294	3538	116	0.032786885	0.394557823	GO:0003682	GO:MF	chromatin binding	TRUE
0.019319415	41	3538	23	0.006500848	0.56097561	GO:0035064	GO:MF	methylated histone binding	FALSE
0.019606584	335	3538	130	0.036743923	0.388059701	GO:0000978	GO:MF	RNA polymerase II cis-regulatory region sequence-specific DNA binding	FALSE
0.021971818	11	3538	9	0.00254381	0.818181818	GO:0140102	GO:MF	catalytic activity, acting on a rRNA	FALSE
0.022737653	132	3538	58	0.016393443	0.439393939	GO:0019207	GO:MF	kinase regulator activity	TRUE
0.023514099	19	3538	13	0.003674392	0.684210526	GO:0008175	GO:MF	tRNA methyltransferase activity	FALSE
0.025943783	64	3538	32	0.009044658	0.5	GO:0008170	GO:MF	N-methyltransferase activity	FALSE
0.027912593	254	3538	101	0.028547202	0.397637795	GO:0004842	GO:MF	ubiquitin-protein transferase activity	TRUE
0.02814999	350	3538	134	0.037874505	0.382857143	GO:0000987	GO:MF	cis-regulatory region sequence-specific DNA binding	FALSE
0.033265424	33	3538	19	0.005370266	0.575757576	GO:0004812	GO:MF	aminoacyl-tRNA ligase activity	FALSE
0.033265424	33	3538	19	0.005370266	0.575757576	GO:0016875	GO:MF	ligase activity, forming carbon-oxygen bonds	FALSE
0.039033755	68	3538	33	0.009327304	0.485294118	GO:0004540	GO:MF	RNA nuclease activity	FALSE
0.040139638	63	3538	31	0.008762012	0.492063492	GO:0004519	GO:MF	endonuclease activity	FALSE
0.041717753	8	3538	7	0.001978519	0.875	GO:0016423	GO:MF	tRNA (guanine) methyltransferase activity	FALSE
0.043235107	29	3538	17	0.004804975	0.586206897	GO:0140938	GO:MF	histone H3 methyltransferase activity	TRUE
0.043235107	169	3538	70	0.019785189	0.414201183	GO:0061630	GO:MF	ubiquitin protein ligase activity	FALSE

0.043855091	66	3538	32	0.009044658	0.484848485	GO:0043022	GO:MF	ribosome binding	FALSE
0.044678506	114	3538	50	0.014132278	0.438596491	GO:0140030	GO:MF	modification-dependent protein binding	FALSE
0.044678506	114	3538	50	0.014132278	0.438596491	GO:0019887	GO:MF	protein kinase regulator activity	FALSE
0.046353982	181	3538	74	0.020915772	0.408839779	GO:0061659	GO:MF	ubiquitin-like protein ligase activity	FALSE
0.047480161	10	3538	8	0.002261164	0.8	GO:0008649	GO:MF	rRNA methyltransferase activity	FALSE
1.24E-06	57	3538	39	0.011023177	0.684210526	KEGG:03008	KEGG	Ribosome biogenesis in eukaryotes	FALSE
8.72E-06	85	3538	50	0.014132278	0.588235294	KEGG:03013	KEGG	Nucleocytoplasmic transport	FALSE
7.38E-05	206	3538	96	0.027133974	0.466019417	KEGG:05168	KEGG	Herpes simplex virus 1 infection	FALSE
0.000278526	37	3538	25	0.007066139	0.675675676	KEGG:00970	KEGG	Aminoacyl-tRNA biosynthesis	FALSE
0.000278526	4554	3538	1495	0.422555116	0.328282828	KEGG:00000	KEGG	KEGG root term	FALSE
0.002123816	134	3538	63	0.01780667	0.470149254	KEGG:04110	KEGG	Cell cycle	FALSE
0.002633302	11	3538	10	0.002826456	0.909090909	KEGG:03450	KEGG	Non-homologous end-joining	FALSE
0.003722014	42	3538	25	0.007066139	0.595238095	KEGG:03420	KEGG	Nucleotide excision repair	FALSE
0.004506834	25	3538	17	0.004804975	0.68	KEGG:03030	KEGG	DNA replication	FALSE
0.005239677	34	3538	21	0.005935557	0.617647059	KEGG:03440	KEGG	Homologous recombination	FALSE
0.00538844	41	3538	24	0.006783493	0.585365854	KEGG:03460	KEGG	Fanconi anemia pathway	FALSE
0.008264218	22	3538	15	0.004239683	0.681818182	KEGG:03020	KEGG	RNA polymerase	FALSE

Yellow Module GO Enrichment

p value	*term size*	*query size*	*overlap size*	*precision*	*recall*	*term id*	*source*	*term name*	*highlighted*
5.20E-10	4750	977	509	0.5209826	0.107157895	GO:0050794	GO:BP	regulation of cellular process	TRUE
1.05E-09	5049	977	531	0.543500512	0.10516934	GO:0065007	GO:BP	biological regulation	FALSE
1.05E-09	4906	977	519	0.531218014	0.10578883	GO:0050789	GO:BP	regulation of biological process	FALSE
4.19E-09	2801	977	327	0.334698055	0.11674402	GO:0019222	GO:BP	regulation of metabolic process	FALSE
1.40E-08	2590	977	304	0.311156602	0.117374517	GO:0048522	GO:BP	positive regulation of cellular process	FALSE
1.40E-08	1605	977	208	0.212896622	0.129595016	GO:0009893	GO:BP	positive regulation of metabolic process	FALSE
2.68E-08	776	977	119	0.121801433	0.153350515	GO:0009057	GO:BP	macromolecule catabolic process	FALSE
2.93E-08	2729	977	315	0.322415558	0.115426896	GO:0048518	GO:BP	positive regulation of biological process	FALSE
2.93E-08	2561	977	299	0.306038895	0.116751269	GO:0060255	GO:BP	regulation of macromolecule metabolic process	FALSE
3.08E-08	1840	977	229	0.234390993	0.124456522	GO:0019538	GO:BP	protein metabolic process	FALSE
3.08E-08	1470	977	192	0.196519959	0.130612245	GO:0010604	GO:BP	positive regulation of macromolecule metabolic process	FALSE
3.08E-08	2284	977	272	0.278403275	0.119089317	GO:0048523	GO:BP	negative regulation of cellular process	FALSE
8.92E-08	1247	977	167	0.170931423	0.133921411	GO:0009892	GO:BP	negative regulation of metabolic process	FALSE
1.05E-07	1301	977	172	0.17604913	0.132205995	GO:0009056	GO:BP	catabolic process	FALSE
1.05E-07	2304	977	271	0.277379734	0.117621528	GO:0080090	GO:BP	regulation of primary metabolic process	FALSE

1.05E-07	2347	977	275	0.2814739	0.117170856	GO:0048519	GO:BP	negative regulation of biological process	FALSE
5.55E-07	566	977	90	0.092118731	0.159010601	GO:0030163	GO:BP	protein catabolic process	FALSE
1.18E-06	1736	977	211	0.215967247	0.121543779	GO:0048583	GO:BP	regulation of response to stimulus	FALSE
1.72E-06	678	977	101	0.103377687	0.148967552	GO:0045934	GO:BP	negative regulation of nucleobase-containing compound metabolic process	FALSE
1.72E-06	607	977	93	0.095189355	0.153212521	GO:0051253	GO:BP	negative regulation of RNA metabolic process	FALSE
2.42E-06	1009	977	136	0.139201638	0.134786918	GO:0009890	GO:BP	negative regulation of biosynthetic process	FALSE
2.63E-06	1156	977	151	0.154554759	0.130622837	GO:0010605	GO:BP	negative regulation of macromolecule metabolic process	FALSE
2.63E-06	552	977	86	0.088024565	0.155797101	GO:0009894	GO:BP	regulation of catabolic process	FALSE
3.00E-06	807	977	114	0.116683726	0.141263941	GO:0045184	GO:BP	establishment of protein localization	FALSE
4.06E-06	2949	977	322	0.329580348	0.109189556	GO:0051716	GO:BP	cellular response to stimulus	FALSE
4.14E-06	3470	977	369	0.377686796	0.106340058	GO:0050896	GO:BP	response to stimulus	FALSE
4.93E-06	986	977	132	0.135107472	0.133874239	GO:0010558	GO:BP	negative regulation of macromolecule biosynthetic process	FALSE
6.03E-06	745	977	106	0.108495394	0.142281879	GO:0051246	GO:BP	regulation of protein metabolic process	FALSE
6.15E-06	2241	977	255	0.261003071	0.113788487	GO:0009889	GO:BP	regulation of biosynthetic process	FALSE
6.34E-06	2179	977	249	0.254861822	0.114272602	GO:0010556	GO:BP	regulation of macromolecule biosynthetic process	FALSE
7.55E-06	1259	977	159	0.162743091	0.126290707	GO:0070727	GO:BP	cellular macromolecule localization	FALSE
8.26E-06	1696	977	202	0.206755374	0.119103774	GO:0006950	GO:BP	response to stress	FALSE
1.09E-05	2472	977	275	0.2814739	0.111245955	GO:0051179	GO:BP	localization	FALSE
1.16E-05	2133	977	243	0.248720573	0.113924051	GO:0010468	GO:BP	regulation of gene expression	FALSE
1.32E-05	1252	977	157	0.160696008	0.125399361	GO:0008104	GO:BP	protein localization	FALSE
1.32E-05	550	977	83	0.084953941	0.150909091	GO:0045892	GO:BP	negative regulation of DNA-templated transcription	FALSE
1.34E-05	1333	977	165	0.16888434	0.123780945	GO:0009966	GO:BP	regulation of signal transduction	FALSE
1.41E-05	296	977	53	0.054247697	0.179054054	GO:0097190	GO:BP	apoptotic signaling pathway	FALSE
1.43E-05	553	977	83	0.084953941	0.150090416	GO:1902679	GO:BP	negative regulation of RNA biosynthetic process	FALSE
1.43E-05	2314	977	259	0.265097236	0.111927398	GO:0007165	GO:BP	signal transduction	FALSE
1.43E-05	782	977	108	0.110542477	0.138107417	GO:0012501	GO:BP	programmed cell death	FALSE
1.43E-05	782	977	108	0.110542477	0.138107417	GO:0008219	GO:BP	cell death	FALSE
1.43E-05	196	977	40	0.040941658	0.204081633	GO:0042176	GO:BP	regulation of protein catabolic process	FALSE
1.72E-05	275	977	50	0.051177073	0.181818182	GO:0051248	GO:BP	negative regulation of protein metabolic process	FALSE
1.90E-05	1455	977	176	0.180143296	0.120962199	GO:0033036	GO:BP	macromolecule localization	FALSE
1.96E-05	392	977	64	0.065506653	0.163265306	GO:0034097	GO:BP	response to cytokine	FALSE
2.07E-05	393	977	64	0.065506653	0.162849873	GO:1901652	GO:BP	response to peptide	FALSE
2.07E-05	2466	977	272	0.278403275	0.110300081	GO:0023052	GO:BP	signaling	FALSE
2.53E-05	1102	977	140	0.143295803	0.127041742	GO:0002376	GO:BP	immune system process	FALSE
2.60E-05	1474	977	177	0.181166837	0.120081411	GO:0023051	GO:BP	regulation of signaling	FALSE

3.41E-05	1470	977	176	0.180143296	0.119727891	GO:0010646	GO:BP	regulation of cell communication	FALSE
4.13E-05	2476	977	271	0.277379734	0.109450727	GO:0007154	GO:BP	cell communication	FALSE
4.27E-05	183	977	37	0.037871034	0.202185792	GO:0009895	GO:BP	negative regulation of catabolic process	FALSE
4.60E-05	2115	977	237	0.242579324	0.112056738	GO:0051234	GO:BP	establishment of localization	FALSE
5.28E-05	396	977	63	0.064483112	0.159090909	GO:0000122	GO:BP	negative regulation of transcription by RNA polymerase II	FALSE
5.79E-05	566	977	82	0.083930399	0.144876325	GO:0033365	GO:BP	protein localization to organelle	FALSE
5.79E-05	1051	977	133	0.136131013	0.126546147	GO:0006366	GO:BP	transcription by RNA polymerase II	FALSE
6.35E-05	744	977	101	0.103377687	0.135752688	GO:0006915	GO:BP	apoptotic process	FALSE
6.35E-05	936	977	121	0.123848516	0.129273504	GO:0045935	GO:BP	positive regulation of nucleobase-containing compound metabolic process	FALSE
6.35E-05	688	977	95	0.097236438	0.138081395	GO:0044419	GO:BP	biological process involved in interspecies interaction between organisms	FALSE
6.86E-05	727	977	99	0.101330604	0.136176066	GO:0002682	GO:BP	regulation of immune system process	FALSE
8.08E-05	655	977	91	0.093142272	0.138931298	GO:0015031	GO:BP	protein transport	FALSE
8.08E-05	1010	977	128	0.131013306	0.126732673	GO:0048584	GO:BP	positive regulation of response to stimulus	FALSE
0.000107905	354	977	57	0.058341863	0.161016949	GO:0071345	GO:BP	cellular response to cytokine stimulus	FALSE
0.000112824	24	977	11	0.011258956	0.458333333	GO:0051205	GO:BP	protein insertion into membrane	TRUE
0.000113858	689	977	94	0.096212897	0.136429608	GO:0006952	GO:BP	defense response	FALSE
0.000167616	1195	977	145	0.148413511	0.121338912	GO:0009891	GO:BP	positive regulation of biosynthetic process	FALSE
0.000204891	115	977	26	0.026612078	0.226086957	GO:0045732	GO:BP	positive regulation of protein catabolic process	FALSE
0.000212663	21	977	10	0.010235415	0.476190476	GO:0045048	GO:BP	protein insertion into ER membrane	FALSE
0.000227511	1162	977	141	0.144319345	0.121342513	GO:0010557	GO:BP	positive regulation of macromolecule biosynthetic process	FALSE
0.000234392	1688	977	192	0.196519959	0.113744076	GO:0019219	GO:BP	regulation of nucleobase-containing compound metabolic process	FALSE
0.000234392	246	977	43	0.044012282	0.174796748	GO:0072594	GO:BP	establishment of protein localization to organelle	FALSE
0.000288139	958	977	120	0.122824974	0.12526096	GO:0033554	GO:BP	cellular response to stress	FALSE
0.000294561	1938	977	215	0.220061412	0.110939112	GO:0006810	GO:BP	transport	FALSE
0.00031113	1707	977	193	0.197543501	0.113063855	GO:0051641	GO:BP	cellular localization	FALSE
0.000322122	651	977	88	0.090071648	0.135176651	GO:0010648	GO:BP	negative regulation of cell communication	FALSE
0.000383466	654	977	88	0.090071648	0.134556575	GO:0023057	GO:BP	negative regulation of signaling	FALSE
0.00042209	637	977	86	0.088024565	0.135007849	GO:0009607	GO:BP	response to biotic stimulus	FALSE
0.00042209	372	977	57	0.058341863	0.153225806	GO:0061024	GO:BP	membrane organization	FALSE
0.00042209	839	977	107	0.109518936	0.127532777	GO:0051254	GO:BP	positive regulation of RNA metabolic process	FALSE
0.000465593	304	977	49	0.050153531	0.161184211	GO:0006954	GO:BP	inflammatory response	FALSE

0.000479666	774	977	100	0.102354145	0.129198966	GO:0048585	GO:BP	negative regulation of response to stimulus	FALSE
0.000479666	1294	977	152	0.155578301	0.117465224	GO:0042221	GO:BP	response to chemical	FALSE
0.000492563	55	977	16	0.016376663	0.290909091	GO:0008630	GO:BP	intrinsic apoptotic signaling pathway in response to DNA damage	FALSE
0.000504417	190	977	35	0.035823951	0.184210526	GO:2001233	GO:BP	regulation of apoptotic signaling pathway	FALSE
0.00056947	807	977	103	0.10542477	0.127633209	GO:0032879	GO:BP	regulation of localization	FALSE
0.00057874	1517	977	173	0.177072671	0.11404087	GO:0051252	GO:BP	regulation of RNA metabolic process	FALSE
0.000583184	1086	977	131	0.13408393	0.120626151	GO:0007166	GO:BP	cell surface receptor signaling pathway	FALSE
0.000627858	608	977	82	0.083930399	0.134868421	GO:0051707	GO:BP	response to other organism	FALSE
0.000627858	608	977	82	0.083930399	0.134868421	GO:0043067	GO:BP	regulation of programmed cell death	FALSE
0.000627858	608	977	82	0.083930399	0.134868421	GO:0043207	GO:BP	response to external biotic stimulus	FALSE
0.000627858	713	977	93	0.095189355	0.130434783	GO:0006955	GO:BP	immune response	FALSE
0.000661642	628	977	84	0.085977482	0.133757962	GO:0009968	GO:BP	negative regulation of signal transduction	FALSE
0.000733006	194	977	35	0.035823951	0.180412371	GO:0006091	GO:BP	generation of precursor metabolites and energy	TRUE
0.00074876	399	977	59	0.060388946	0.147869674	GO:0051247	GO:BP	positive regulation of protein metabolic process	FALSE
0.00091472	653	977	86	0.088024565	0.131699847	GO:0080134	GO:BP	regulation of response to stress	FALSE
0.000915204	987	977	120	0.122824974	0.121580547	GO:0006357	GO:BP	regulation of transcription by RNA polymerase II	FALSE
0.00091864	305	977	48	0.04912999	0.157377049	GO:0009896	GO:BP	positive regulation of catabolic process	FALSE
0.000930603	12	977	7	0.00716479	0.583333333	GO:0071816	GO:BP	tail-anchored membrane protein insertion into ER membrane	FALSE
0.000935742	578	977	78	0.079836233	0.134948097	GO:0042981	GO:BP	regulation of apoptotic process	FALSE
0.001039767	851	977	106	0.108495394	0.124559342	GO:0071705	GO:BP	nitrogen compound transport	FALSE
0.001082888	1443	977	164	0.167860798	0.113652114	GO:0006351	GO:BP	DNA-templated transcription	FALSE
0.001470258	201	977	35	0.035823951	0.174129353	GO:1903706	GO:BP	regulation of hemopoiesis	FALSE
0.001479857	455	977	64	0.065506653	0.140659341	GO:0051603	GO:BP	proteolysis involved in protein catabolic process	FALSE
0.001731294	724	977	92	0.094165814	0.127071823	GO:0006508	GO:BP	proteolysis	FALSE
0.002051435	172	977	31	0.031729785	0.180232558	GO:0031647	GO:BP	regulation of protein stability	FALSE
0.002051435	554	977	74	0.075742068	0.133574007	GO:0010628	GO:BP	positive regulation of gene expression	FALSE
0.002272967	173	977	31	0.031729785	0.179190751	GO:1903311	GO:BP	regulation of mRNA metabolic process	FALSE
0.00250741	158	977	29	0.029682702	0.183544304	GO:1902105	GO:BP	regulation of leukocyte differentiation	FALSE
0.002566506	4	977	4	0.004094166	1	GO:1902337	GO:BP	regulation of apoptotic process involved in morphogenesis	FALSE
0.002566506	4	977	4	0.004094166	1	GO:1904747	GO:BP	positive regulation of apoptotic process involved in development	FALSE

0.002566506	4	977	4	0.004094166	1	GO:1902339	GO:BP	positive regulation of apoptotic process involved in morphogenesis	FALSE
0.002566506	4	977	4	0.004094166	1	GO:1904748	GO:BP	regulation of apoptotic process involved in development	FALSE
0.00257154	446	977	62	0.06345957	0.139013453	GO:0060341	GO:BP	regulation of cellular localization	FALSE
0.003295852	185	977	32	0.032753327	0.172972973	GO:0030162	GO:BP	regulation of proteolysis	FALSE
0.003360223	161	977	29	0.029682702	0.180124224	GO:0097193	GO:BP	intrinsic apoptotic signaling pathway	FALSE
0.003600444	910	977	109	0.111566018	0.11978022	GO:0009605	GO:BP	response to external stimulus	FALSE
0.003600444	93	977	20	0.020470829	0.215053763	GO:2000058	GO:BP	regulation of ubiquitin-dependent protein catabolic process	FALSE
0.004900162	364	977	52	0.053224156	0.142857143	GO:0043069	GO:BP	negative regulation of programmed cell death	FALSE
0.004933852	504	977	67	0.068577277	0.132936508	GO:0070647	GO:BP	protein modification by small protein conjugation or removal	TRUE
0.005149826	54	977	14	0.01432958	0.259259259	GO:0042177	GO:BP	negative regulation of protein catabolic process	FALSE
0.00523042	338	977	49	0.050153531	0.144970414	GO:0046903	GO:BP	secretion	FALSE
0.005269041	11	977	6	0.006141249	0.545454545	GO:0045723	GO:BP	positive regulation of fatty acid biosynthetic process	FALSE
0.005434595	48	977	13	0.013306039	0.270833333	GO:0008625	GO:BP	extrinsic apoptotic signaling pathway via death domain receptors	FALSE
0.005605883	42	977	12	0.012282497	0.285714286	GO:0072332	GO:BP	intrinsic apoptotic signaling pathway by p53 class mediator	FALSE
0.00578311	358	977	51	0.052200614	0.142458101	GO:0051668	GO:BP	localization within membrane	FALSE
0.005903441	470	977	63	0.064483112	0.134042553	GO:0045321	GO:BP	leukocyte activation	FALSE
0.006043799	368	977	52	0.053224156	0.141304348	GO:0006412	GO:BP	translation	FALSE
0.006362981	606	977	77	0.078812692	0.127062706	GO:0051049	GO:BP	regulation of transport	FALSE
0.006871186	361	977	51	0.052200614	0.141274238	GO:0019941	GO:BP	modification-dependent protein catabolic process	FALSE
0.006871186	43	977	12	0.012282497	0.279069767	GO:0048144	GO:BP	fibroblast proliferation	TRUE
0.006957938	343	977	49	0.050153531	0.142857143	GO:0043066	GO:BP	negative regulation of apoptotic process	FALSE
0.00699747	531	977	69	0.07062436	0.129943503	GO:0043687	GO:BP	post-translational protein modification	FALSE
0.00699747	307	977	45	0.046059365	0.146579805	GO:0072657	GO:BP	protein localization to membrane	FALSE
0.007022586	727	977	89	0.091095189	0.122420908	GO:0023056	GO:BP	positive regulation of signaling	FALSE
0.007053269	263	977	40	0.040941658	0.152091255	GO:0080135	GO:BP	regulation of cellular response to stress	FALSE
0.007053269	362	977	51	0.052200614	0.140883978	GO:0043632	GO:BP	modification-dependent macromolecule catabolic process	FALSE
0.007127352	26	977	9	0.009211873	0.346153846	GO:0042773	GO:BP	ATP synthesis coupled electron transport	FALSE
0.007127352	26	977	9	0.009211873	0.346153846	GO:0042775	GO:BP	mitochondrial ATP synthesis coupled electron transport	FALSE

0.007769242	720	977	88	0.090071648	0.122222222	GO:0010647	GO:BP	positive regulation of cell communication	FALSE
0.007769242	8	977	5	0.005117707	0.625	GO:0060561	GO:BP	apoptotic process involved in morphogenesis	FALSE
0.008201967	57	977	14	0.01432958	0.245614035	GO:0045861	GO:BP	negative regulation of proteolysis	FALSE
0.0082436	115	977	22	0.022517912	0.191304348	GO:2001234	GO:BP	negative regulation of apoptotic signaling pathway	FALSE
0.008595068	71	977	16	0.016376663	0.225352113	GO:0009060	GO:BP	aerobic respiration	FALSE
0.009151251	451	977	60	0.061412487	0.133037694	GO:0050776	GO:BP	regulation of immune response	FALSE
0.009441782	86	977	18	0.018423746	0.209302326	GO:0072524	GO:BP	pyridine-containing compound metabolic process	FALSE
0.009795763	45	977	12	0.012282497	0.266666667	GO:0006119	GO:BP	oxidative phosphorylation	FALSE
0.010063484	520	977	67	0.068577277	0.128846154	GO:0001775	GO:BP	cell activation	FALSE
0.010219298	22	977	8	0.008188332	0.363636364	GO:0042771	GO:BP	intrinsic apoptotic signaling pathway in response to DNA damage by p53 class mediator	FALSE
0.010219298	17	977	7	0.00716479	0.411764706	GO:0090207	GO:BP	regulation of triglyceride metabolic process	FALSE
0.010219298	1074	977	122	0.124872057	0.113594041	GO:0051128	GO:BP	regulation of cellular component organization	FALSE
0.010219298	149	977	26	0.026612078	0.174496644	GO:0090150	GO:BP	establishment of protein localization to membrane	FALSE
0.010300704	278	977	41	0.0419652	0.147482014	GO:0034655	GO:BP	nucleobase-containing compound catabolic process	FALSE
0.010417012	87	977	18	0.018423746	0.206896552	GO:0045333	GO:BP	cellular respiration	FALSE
0.011075464	352	977	49	0.050153531	0.139204545	GO:0006511	GO:BP	ubiquitin-dependent protein catabolic process	FALSE
0.011123188	1361	977	149	0.152507677	0.109478325	GO:0006355	GO:BP	regulation of DNA-templated transcription	FALSE
0.011123188	552	977	70	0.071647902	0.126811594	GO:0045944	GO:BP	positive regulation of transcription by RNA polymerase II	FALSE
0.01145064	262	977	39	0.039918117	0.148854962	GO:0010256	GO:BP	endomembrane system organization	FALSE
0.01145064	262	977	39	0.039918117	0.148854962	GO:1903131	GO:BP	mononuclear cell differentiation	FALSE
0.011508507	428	977	57	0.058341863	0.13317757	GO:0032446	GO:BP	protein modification by small protein conjugation	FALSE
0.01187548	111	977	21	0.021494371	0.189189189	GO:0050821	GO:BP	protein stabilization	FALSE
0.011967402	40	977	11	0.011258956	0.275	GO:0046889	GO:BP	positive regulation of lipid biosynthetic process	FALSE
0.012188493	477	977	62	0.06345957	0.129979036	GO:0098542	GO:BP	defense response to other organism	FALSE
0.012594127	526	977	67	0.068577277	0.127376426	GO:0002684	GO:BP	positive regulation of immune system process	FALSE
0.012710954	211	977	33	0.033776868	0.156398104	GO:0019221	GO:BP	cytokine-mediated signaling pathway	FALSE
0.01284743	897	977	104	0.106448311	0.115942029	GO:1902531	GO:BP	regulation of intracellular signal transduction	FALSE
0.01284743	1367	977	149	0.152507677	0.108997805	GO:2001141	GO:BP	regulation of RNA biosynthetic process	FALSE
0.012984518	393	977	53	0.054247697	0.134860051	GO:0032880	GO:BP	regulation of protein localization	FALSE

0.013061063	23	977	8	0.008188332	0.347826087	GO:0019646	GO:BP	aerobic electron transport chain	FALSE
0.013494303	9	977	5	0.005117707	0.555555556	GO:0032692	GO:BP	negative regulation of interleukin-1 production	TRUE
0.014015746	105	977	20	0.020470829	0.19047619	GO:0045619	GO:BP	regulation of lymphocyte differentiation	FALSE
0.014077266	1170	977	130	0.133060389	0.111111111	GO:0051239	GO:BP	regulation of multicellular organismal process	FALSE
0.014167891	41	977	11	0.011258956	0.268292683	GO:0022900	GO:BP	electron transport chain	FALSE
0.014242172	758	977	90	0.092118731	0.118733509	GO:0045893	GO:BP	positive regulation of DNA-templated transcription	FALSE
0.014242172	129	977	23	0.023541453	0.178294574	GO:1903050	GO:BP	regulation of proteolysis involved in protein catabolic process	FALSE
0.014331337	29	977	9	0.009211873	0.310344828	GO:2001244	GO:BP	positive regulation of intrinsic apoptotic signaling pathway	FALSE
0.014530237	35	977	10	0.010235415	0.285714286	GO:0048145	GO:BP	regulation of fibroblast proliferation	FALSE
0.014759883	482	977	62	0.06345957	0.128630705	GO:0030097	GO:BP	hemopoiesis	FALSE
0.015136575	760	977	90	0.092118731	0.118421053	GO:1902680	GO:BP	positive regulation of RNA biosynthetic process	FALSE
0.015429353	223	977	34	0.034800409	0.152466368	GO:0043068	GO:BP	positive regulation of programmed cell death	FALSE
0.015575096	1080	977	121	0.123848516	0.112037037	GO:0065008	GO:BP	regulation of biological quality	FALSE
0.016037942	69	977	15	0.015353122	0.217391304	GO:2001235	GO:BP	positive regulation of apoptotic signaling pathway	FALSE
0.01619367	62	977	14	0.01432958	0.225806452	GO:1901800	GO:BP	positive regulation of proteasomal protein catabolic process	FALSE
0.016268353	1420	977	153	0.156601842	0.107746479	GO:0035556	GO:BP	intracellular signal transduction	FALSE
0.017226359	216	977	33	0.033776868	0.152777778	GO:0030098	GO:BP	lymphocyte differentiation	FALSE
0.017226359	371	977	50	0.051177073	0.134770889	GO:0050778	GO:BP	positive regulation of immune response	FALSE
0.017226359	315	977	44	0.045035824	0.13968254	GO:0002521	GO:BP	leukocyte differentiation	FALSE
0.017533234	36	977	10	0.010235415	0.277777778	GO:0022904	GO:BP	respiratory electron transport chain	FALSE
0.017659183	30	977	9	0.009211873	0.3	GO:2000059	GO:BP	negative regulation of ubiquitin-dependent protein catabolic process	FALSE
0.018002744	100	977	19	0.019447288	0.19	GO:0045862	GO:BP	positive regulation of proteolysis	FALSE
0.018020495	391	977	52	0.053224156	0.132992327	GO:0140546	GO:BP	defense response to symbiont	FALSE
0.018020495	3	977	3	0.003070624	1	GO:0006122	GO:BP	mitochondrial electron transport, ubiquinol to cytochrome c	FALSE
0.018023456	132	977	23	0.023541453	0.174242424	GO:1903320	GO:BP	regulation of protein modification by small protein conjugation or removal	FALSE
0.018023456	56	977	13	0.013306039	0.232142857	GO:0007029	GO:BP	endoplasmic reticulum organization	FALSE
0.018023456	298	977	42	0.042988741	0.140939597	GO:0032940	GO:BP	secretion by cell	FALSE
0.018526283	777	977	91	0.093142272	0.117117117	GO:0016192	GO:BP	vesicle-mediated transport	FALSE
0.019095408	383	977	51	0.052200614	0.133159269	GO:0045087	GO:BP	innate immune response	FALSE

0.019129652	6	977	4	0.004094166	0.666666667	GO:0045647	GO:BP	negative regulation of erythrocyte differentiation	TRUE
0.019214042	667	977	80	0.081883316	0.11994003	GO:0009967	GO:BP	positive regulation of signal transduction	FALSE
0.019513073	245	977	36	0.036847492	0.146938776	GO:0002683	GO:BP	negative regulation of immune system process	FALSE
0.019550602	2247	977	228	0.233367451	0.101468625	GO:0048856	GO:BP	anatomical structure development	FALSE
0.019762107	403	977	53	0.054247697	0.131513648	GO:0046649	GO:BP	lymphocyte activation	FALSE
0.020043309	150	977	25	0.025588536	0.166666667	GO:0007249	GO:BP	canonical NF-kappaB signal transduction	TRUE
0.020043309	184	977	29	0.029682702	0.157608696	GO:1903530	GO:BP	regulation of secretion by cell	FALSE
0.020043309	50	977	12	0.012282497	0.24	GO:0019882	GO:BP	antigen processing and presentation	FALSE
0.020533149	10	977	5	0.005117707	0.5	GO:0061158	GO:BP	3'-UTR-mediated mRNA destabilization	TRUE
0.020533149	10	977	5	0.005117707	0.5	GO:0010866	GO:BP	regulation of triglyceride biosynthetic process	FALSE
0.020533149	10	977	5	0.005117707	0.5	GO:1904294	GO:BP	positive regulation of ERAD pathway	FALSE
0.021098418	79	977	16	0.016376663	0.202531646	GO:0071356	GO:BP	cellular response to tumor necrosis factor	FALSE
0.021264668	118	977	21	0.021494371	0.177966102	GO:0097191	GO:BP	extrinsic apoptotic signaling pathway	FALSE
0.021402835	151	977	25	0.025588536	0.165562914	GO:0050727	GO:BP	regulation of inflammatory response	FALSE
0.021650331	194	977	30	0.030706244	0.154639175	GO:0016236	GO:BP	macroautophagy	FALSE
0.021650331	2409	977	242	0.247697032	0.100456621	GO:0032502	GO:BP	developmental process	FALSE
0.022017107	44	977	11	0.011258956	0.25	GO:0071456	GO:BP	cellular response to hypoxia	FALSE
0.022419573	135	977	23	0.023541453	0.17037037	GO:1901873	GO:BP	regulation of post-translational protein modification	FALSE
0.022714754	359	977	48	0.04912999	0.133704735	GO:0001817	GO:BP	regulation of cytokine production	FALSE
0.023498062	285	977	40	0.040941658	0.140350877	GO:0010608	GO:BP	post-transcriptional regulation of gene expression	FALSE
0.023500971	204	977	31	0.031729785	0.151960784	GO:0051046	GO:BP	regulation of secretion	FALSE
0.023833524	564	977	69	0.07062436	0.122340426	GO:2000026	GO:BP	regulation of multicellular organismal development	FALSE
0.023833524	15	977	6	0.006141249	0.4	GO:1905710	GO:BP	positive regulation of membrane permeability	TRUE
0.023844224	972	977	109	0.111566018	0.112139918	GO:0050793	GO:BP	regulation of developmental process	FALSE
0.02388517	725	977	85	0.087001024	0.117241379	GO:0042592	GO:BP	homeostatic process	TRUE
0.024433402	447	977	57	0.058341863	0.127516779	GO:0060429	GO:BP	epithelium development	TRUE
0.025146731	214	977	32	0.032753327	0.14953271	GO:0043065	GO:BP	positive regulation of apoptotic process	FALSE
0.025645097	45	977	11	0.011258956	0.244444444	GO:0036294	GO:BP	cellular response to decreased oxygen levels	FALSE
0.025928008	81	977	16	0.016376663	0.197530864	GO:0046890	GO:BP	regulation of lipid biosynthetic process	FALSE
0.02609016	52	977	12	0.012282497	0.230769231	GO:0170062	GO:BP	nutrient storage	FALSE
0.02609016	52	977	12	0.012282497	0.230769231	GO:0019915	GO:BP	lipid storage	TRUE
0.02609016	52	977	12	0.012282497	0.230769231	GO:0045834	GO:BP	positive regulation of lipid metabolic process	FALSE
0.027153477	363	977	48	0.04912999	0.132231405	GO:0001816	GO:BP	cytokine production	FALSE

0.027228448	811	977	93	0.095189355	0.114673243	GO:0070887	GO:BP	cellular response to chemical stimulus	FALSE
0.027243341	74	977	15	0.015353122	0.202702703	GO:1903052	GO:BP	positive regulation of proteolysis involved in protein catabolic process	FALSE
0.027270389	588	977	71	0.072671443	0.120748299	GO:0045595	GO:BP	regulation of cell differentiation	FALSE
0.02849444	82	977	16	0.016376663	0.195121951	GO:0019362	GO:BP	pyridine nucleotide metabolic process	FALSE
0.02849444	82	977	16	0.016376663	0.195121951	GO:1903313	GO:BP	positive regulation of mRNA metabolic process	FALSE
0.02849444	82	977	16	0.016376663	0.195121951	GO:0046496	GO:BP	nicotinamide nucleotide metabolic process	FALSE
0.028810381	355	977	47	0.048106448	0.132394366	GO:0006886	GO:BP	intracellular protein transport	FALSE
0.029342639	90	977	17	0.017400205	0.188888889	GO:0006457	GO:BP	protein folding	TRUE
0.029558411	33	977	9	0.009211873	0.272727273	GO:0061077	GO:BP	chaperone-mediated protein folding	FALSE
0.029558411	53	977	12	0.012282497	0.226415094	GO:1903312	GO:BP	negative regulation of mRNA metabolic process	FALSE
0.029558411	114	977	20	0.020470829	0.175438596	GO:0006282	GO:BP	regulation of DNA repair	FALSE
0.029558411	106	977	19	0.019447288	0.179245283	GO:0043487	GO:BP	regulation of RNA stability	FALSE
0.029743272	337	977	45	0.046059365	0.133531157	GO:0060284	GO:BP	regulation of cell development	FALSE
0.029754684	11	977	5	0.005117707	0.454545455	GO:0006620	GO:BP	post-translational protein targeting to endoplasmic reticulum membrane	TRUE
0.029754684	11	977	5	0.005117707	0.454545455	GO:0090208	GO:BP	positive regulation of triglyceride metabolic process	FALSE
0.030015345	27	977	8	0.008188332	0.296296296	GO:0097300	GO:BP	programmed necrotic cell death	FALSE
0.030182393	300	977	41	0.0419652	0.136666667	GO:0006914	GO:BP	autophagy	FALSE
0.030182393	300	977	41	0.0419652	0.136666667	GO:0061919	GO:BP	process utilizing autophagic mechanism	FALSE
0.030587683	319	977	43	0.044012282	0.134796238	GO:0010498	GO:BP	proteasomal protein catabolic process	FALSE
0.030780579	83	977	16	0.016376663	0.192771084	GO:0046034	GO:BP	ATP metabolic process	TRUE
0.030878615	123	977	21	0.021494371	0.170731707	GO:0002573	GO:BP	myeloid leukocyte differentiation	FALSE
0.031472117	91	977	17	0.017400205	0.186813187	GO:0034612	GO:BP	response to tumor necrosis factor	FALSE
0.032350204	7	977	4	0.004094166	0.571428571	GO:0070230	GO:BP	positive regulation of lymphocyte apoptotic process	TRUE
0.032350204	7	977	4	0.004094166	0.571428571	GO:0032691	GO:BP	negative regulation of interleukin-1 beta production	FALSE
0.032350204	7	977	4	0.004094166	0.571428571	GO:0036151	GO:BP	phosphatidylcholine acyl-chain remodeling	TRUE
0.033083601	274	977	38	0.038894575	0.138686131	GO:0002694	GO:BP	regulation of leukocyte activation	FALSE
0.033206084	175	977	27	0.027635619	0.154285714	GO:0016032	GO:BP	viral process	TRUE
0.033736665	293	977	40	0.040941658	0.136518771	GO:0050865	GO:BP	regulation of cell activation	FALSE
0.03391664	158	977	25	0.025588536	0.158227848	GO:0030217	GO:BP	T cell differentiation	FALSE
0.034560871	108	977	19	0.019447288	0.175925926	GO:0045637	GO:BP	regulation of myeloid cell differentiation	FALSE
0.034651469	100	977	18	0.018423746	0.18	GO:0043484	GO:BP	regulation of RNA splicing	FALSE

0.034999185	322	977	43	0.044012282	0.133540373	GO:0051129	GO:BP	negative regulation of cellular component organization	FALSE
0.0352164	150	977	24	0.024564995	0.16	GO:0006605	GO:BP	protein targeting	FALSE
0.036310675	28	977	8	0.008188332	0.285714286	GO:0098586	GO:BP	cellular response to virus	TRUE
0.036392025	77	977	15	0.015353122	0.194805195	GO:0072331	GO:BP	signal transduction by p53 class mediator	FALSE
0.036638659	323	977	43	0.044012282	0.133126935	GO:0140352	GO:BP	export from cell	FALSE
0.036902415	41	977	10	0.010235415	0.243902439	GO:0061614	GO:BP	miRNA transcription	FALSE
0.036902415	41	977	10	0.010235415	0.243902439	GO:1902893	GO:BP	regulation of miRNA transcription	FALSE
0.03701068	85	977	16	0.016376663	0.188235294	GO:0006839	GO:BP	mitochondrial transport	FALSE
0.03701068	85	977	16	0.016376663	0.188235294	GO:0016052	GO:BP	carbohydrate catabolic process	FALSE
0.037298395	410	977	52	0.053224156	0.126829268	GO:0051241	GO:BP	negative regulation of multicellular organismal process	FALSE
0.037638891	240	977	34	0.034800409	0.141666667	GO:0030099	GO:BP	myeloid cell differentiation	FALSE
0.037638891	186	977	28	0.028659161	0.150537634	GO:0141188	GO:BP	nucleic acid catabolic process	FALSE
0.038316841	401	977	51	0.052200614	0.127182045	GO:0048878	GO:BP	chemical homeostasis	FALSE
0.038464002	126	977	21	0.021494371	0.166666667	GO:0019058	GO:BP	viral life cycle	FALSE
0.038585569	315	977	42	0.042988741	0.133333333	GO:0051050	GO:BP	positive regulation of transport	FALSE
0.038946292	169	977	26	0.026612078	0.153846154	GO:0002697	GO:BP	regulation of immune effector process	FALSE
0.039048016	250	977	35	0.035823951	0.14	GO:0051249	GO:BP	regulation of lymphocyte activation	FALSE
0.039048016	490	977	60	0.061412487	0.12244898	GO:0044087	GO:BP	regulation of cellular component biogenesis	FALSE
0.039176483	278	977	38	0.038894575	0.136690647	GO:0033993	GO:BP	response to lipid	FALSE
0.039176483	110	977	19	0.019447288	0.172727273	GO:0031396	GO:BP	regulation of protein ubiquitination	FALSE
0.039176483	35	977	9	0.009211873	0.257142857	GO:0022600	GO:BP	digestive system process	TRUE
0.039176483	35	977	9	0.009211873	0.257142857	GO:2000630	GO:BP	positive regulation of miRNA metabolic process	FALSE
0.039176483	110	977	19	0.019447288	0.172727273	GO:0061136	GO:BP	regulation of proteasomal protein catabolic process	FALSE
0.039176483	63	977	13	0.013306039	0.206349206	GO:0050779	GO:BP	RNA destabilization	FALSE
0.039176483	17	977	6	0.006141249	0.352941176	GO:0045923	GO:BP	positive regulation of fatty acid metabolic process	FALSE
0.039176483	35	977	9	0.009211873	0.257142857	GO:0007586	GO:BP	digestion	FALSE
0.039754272	94	977	17	0.017400205	0.180851064	GO:2001242	GO:BP	regulation of intrinsic apoptotic signaling pathway	FALSE
0.039989445	12	977	5	0.005117707	0.416666667	GO:0009648	GO:BP	photoperiodism	FALSE
0.039989445	12	977	5	0.005117707	0.416666667	GO:1902742	GO:BP	apoptotic process involved in development	FALSE
0.039989445	12	977	5	0.005117707	0.416666667	GO:0043153	GO:BP	entrainment of circadian clock by photoperiod	TRUE
0.040055649	127	977	21	0.021494371	0.165354331	GO:0002237	GO:BP	response to molecule of bacterial origin	FALSE
0.040253694	242	977	34	0.034800409	0.140495868	GO:0031349	GO:BP	positive regulation of defense response	FALSE
0.040253694	56	977	12	0.012282497	0.214285714	GO:0072526	GO:BP	pyridine-containing compound catabolic process	FALSE
0.041115911	119	977	20	0.020470829	0.168067227	GO:0032496	GO:BP	response to lipopolysaccharide	FALSE
0.041476192	29	977	8	0.008188332	0.275862069	GO:0032963	GO:BP	collagen metabolic process	TRUE

0.042109397	4	977	3	0.003070624	0.75	GO:0045843	GO:BP	negative regulation of striated muscle tissue development	FALSE
0.042109397	4	977	3	0.003070624	0.75	GO:0030388	GO:BP	fructose 1,6-bisphosphate metabolic process	TRUE
0.042109397	23	977	7	0.00716479	0.304347826	GO:0070498	GO:BP	interleukin-1-mediated signaling pathway	FALSE
0.042109397	4	977	3	0.003070624	0.75	GO:0048635	GO:BP	negative regulation of muscle organ development	FALSE
0.042109397	4	977	3	0.003070624	0.75	GO:1903935	GO:BP	response to sodium arsenite	FALSE
0.042109397	79	977	15	0.015353122	0.189873418	GO:0050684	GO:BP	regulation of mRNA processing	FALSE
0.042109397	128	977	21	0.021494371	0.1640625	GO:0015980	GO:BP	energy derivation by oxidation of organic compounds	FALSE
0.042109397	4	977	3	0.003070624	0.75	GO:1903936	GO:BP	cellular response to sodium arsenite	FALSE
0.042109397	4	977	3	0.003070624	0.75	GO:2000660	GO:BP	negative regulation of interleukin-1-mediated signaling pathway	FALSE
0.042109397	95	977	17	0.017400205	0.178947368	GO:0009205	GO:BP	purine ribonucleoside triphosphate metabolic process	FALSE
0.042109397	4	977	3	0.003070624	0.75	GO:1901862	GO:BP	negative regulation of muscle tissue development	FALSE
0.042109397	4	977	3	0.003070624	0.75	GO:1902075	GO:BP	cellular response to salt	FALSE
0.043740801	300	977	40	0.040941658	0.133333333	GO:0032103	GO:BP	positive regulation of response to external stimulus	FALSE
0.045499464	146	977	23	0.023541453	0.157534247	GO:0002460	GO:BP	adaptive immune response based on somatic recombination of immune receptors built from immunoglobulin superfamily domains	TRUE
0.046041029	50	977	11	0.011258956	0.22	GO:0072655	GO:BP	establishment of protein localization to mitochondrion	FALSE
0.046041029	96	977	17	0.017400205	0.177083333	GO:0010563	GO:BP	negative regulation of phosphorus metabolic process	FALSE
0.046041029	96	977	17	0.017400205	0.177083333	GO:0045936	GO:BP	negative regulation of phosphate metabolic process	FALSE
0.046041029	50	977	11	0.011258956	0.22	GO:2000628	GO:BP	regulation of miRNA metabolic process	FALSE
0.046834177	236	977	33	0.033776868	0.139830508	GO:0030855	GO:BP	epithelial cell differentiation	FALSE
0.046834177	164	977	25	0.025588536	0.152439024	GO:0042330	GO:BP	taxis	FALSE
0.046834177	164	977	25	0.025588536	0.152439024	GO:0006935	GO:BP	chemotaxis	FALSE
0.046848205	138	977	22	0.022517912	0.15942029	GO:0006887	GO:BP	exocytosis	FALSE
0.047195298	173	977	26	0.026612078	0.150289017	GO:0006401	GO:BP	RNA catabolic process	FALSE
0.047195298	477	977	58	0.059365404	0.121593291	GO:1902533	GO:BP	positive regulation of intracellular signal transduction	FALSE
0.047248898	8	977	4	0.004094166	0.5	GO:0002675	GO:BP	positive regulation of acute inflammatory response	TRUE
0.047248898	8	977	4	0.004094166	0.5	GO:0002467	GO:BP	germinal center formation	FALSE

0.047248898	8	977	4	0.004094166	0.5	GO:0010867	GO:BP	positive regulation of triglyceride biosynthetic process	FALSE
0.047248898	8	977	4	0.004094166	0.5	GO:0002357	GO:BP	defense response to tumor cell	FALSE
0.047248898	8	977	4	0.004094166	0.5	GO:0043302	GO:BP	positive regulation of leukocyte degranulation	TRUE
0.047248898	8	977	4	0.004094166	0.5	GO:0002922	GO:BP	positive regulation of humoral immune response	FALSE
0.047559215	274	977	37	0.037871034	0.135036496	GO:0042110	GO:BP	T cell activation	FALSE
0.04829248	156	977	24	0.024564995	0.153846154	GO:0034976	GO:BP	response to endoplasmic reticulum stress	FALSE
0.049024296	58	977	12	0.012282497	0.206896552	GO:2000060	GO:BP	positive regulation of ubiquitin-dependent protein catabolic process	FALSE
0.049024296	58	977	12	0.012282497	0.206896552	GO:0090087	GO:BP	regulation of peptide transport	FALSE
0.049557915	24	977	7	0.00716479	0.291666667	GO:0042304	GO:BP	regulation of fatty acid biosynthetic process	FALSE
1.57E-15	5580	977	599	0.613101331	0.10734767	GO:0005737	GO:CC	cytoplasm	TRUE
0.000128095	318	977	53	0.054247697	0.166666667	GO:0005773	GO:CC	vacuole	FALSE
0.000128095	260	977	46	0.047082907	0.176923077	GO:0005764	GO:CC	lysosome	FALSE
0.000128095	3584	977	370	0.378710338	0.103236607	GO:0016020	GO:CC	membrane	FALSE
0.000128095	1403	977	169	0.172978506	0.120456165	GO:0031090	GO:CC	organelle membrane	FALSE
0.000128095	260	977	46	0.047082907	0.176923077	GO:0000323	GO:CC	lytic vacuole	FALSE
0.000166472	748	977	100	0.102354145	0.13368984	GO:0098588	GO:CC	bounding membrane of organelle	FALSE
0.000166472	846	977	110	0.11258956	0.130023641	GO:0005739	GO:CC	mitochondrion	FALSE
0.000297727	176	977	34	0.034800409	0.193181818	GO:0005774	GO:CC	vacuolar membrane	FALSE
0.000893129	140	977	28	0.028659161	0.2	GO:0005765	GO:CC	lysosomal membrane	FALSE
0.000893129	140	977	28	0.028659161	0.2	GO:0098852	GO:CC	lytic vacuole membrane	FALSE
0.000962105	1933	977	211	0.215967247	0.109156751	GO:0012505	GO:CC	endomembrane system	FALSE
0.001307726	144	977	28	0.028659161	0.194444444	GO:0005840	GO:CC	ribosome	FALSE
0.002118917	96	977	21	0.021494371	0.21875	GO:0098798	GO:CC	mitochondrial protein-containing complex	FALSE
0.002256466	2171	977	230	0.235414534	0.105941962	GO:0005654	GO:CC	nucleoplasm	TRUE
0.002717941	10	977	6	0.006141249	0.6	GO:0072379	GO:CC	ER membrane insertion complex	FALSE
0.003108507	2131	977	225	0.230296827	0.105584233	GO:0005829	GO:CC	cytosol	FALSE
0.003112711	819	977	100	0.102354145	0.122100122	GO:0005783	GO:CC	endoplasmic reticulum	FALSE
0.005721291	2745	977	278	0.284544524	0.101275046	GO:0031974	GO:CC	membrane-enclosed lumen	FALSE
0.005721291	2745	977	278	0.284544524	0.101275046	GO:0043233	GO:CC	organelle lumen	FALSE
0.005721291	2745	977	278	0.284544524	0.101275046	GO:0070013	GO:CC	intracellular organelle lumen	FALSE
0.006268967	879	977	104	0.106448311	0.118316268	GO:0031982	GO:CC	vesicle	FALSE
0.009897929	217	977	34	0.034800409	0.156682028	GO:0010008	GO:CC	endosome membrane	FALSE
0.011171602	300	977	43	0.044012282	0.143333333	GO:0031966	GO:CC	mitochondrial membrane	FALSE
0.011171602	327	977	46	0.047082907	0.140672783	GO:0005740	GO:CC	mitochondrial envelope	FALSE
0.011171602	53	977	13	0.013306039	0.245283019	GO:0005811	GO:CC	lipid droplet	FALSE
0.013437609	146	977	25	0.025588536	0.171232877	GO:0005770	GO:CC	late endosome	FALSE
0.013437609	2579	977	259	0.265097236	0.100426522	GO:0043228	GO:CC	membraneless organelle	FALSE
0.013437609	2579	977	259	0.265097236	0.100426522	GO:0043232	GO:CC	intracellular membraneless organelle	FALSE
0.014027368	24	977	8	0.008188332	0.333333333	GO:0005771	GO:CC	multivesicular body	FALSE
0.014824803	3	977	3	0.003070624	1	GO:0071818	GO:CC	BAT3 complex	FALSE
0.014824803	3	977	3	0.003070624	1	GO:1990037	GO:CC	Lewy body core	TRUE
0.015215003	818	977	95	0.097236438	0.116136919	GO:0031410	GO:CC	cytoplasmic vesicle	FALSE
0.015432292	64	977	14	0.01432958	0.21875	GO:0031902	GO:CC	late endosome membrane	FALSE
0.015432292	461	977	59	0.060388946	0.127982646	GO:0005768	GO:CC	endosome	FALSE

0.015432292	559	977	69	0.07062436	0.123434705	GO:0031967	GO:CC	organelle envelope	FALSE
0.015432292	821	977	95	0.097236438	0.115712546	GO:0097708	GO:CC	intracellular vesicle	FALSE
0.018393243	15	977	6	0.006141249	0.4	GO:0071010	GO:CC	prespliceosome	FALSE
0.018393243	15	977	6	0.006141249	0.4	GO:0071004	GO:CC	U2-type prespliceosome	TRUE
0.023413202	375	977	49	0.050153531	0.130666667	GO:0012506	GO:CC	vesicle membrane	FALSE
0.023413202	2518	977	250	0.255885363	0.099285147	GO:0031981	GO:CC	nuclear lumen	FALSE
0.023413202	33	977	9	0.009211873	0.272727273	GO:0005762	GO:CC	mitochondrial large ribosomal subunit	FALSE
0.023413202	33	977	9	0.009211873	0.272727273	GO:0000315	GO:CC	organellar large ribosomal subunit	FALSE
0.023846299	148	977	24	0.024564995	0.162162162	GO:0090575	GO:CC	RNA polymerase II transcription regulator complex	TRUE
0.023846299	174	977	27	0.027635619	0.155172414	GO:0005743	GO:CC	mitochondrial inner membrane	FALSE
0.02969525	48	977	11	0.011258956	0.229166667	GO:0000313	GO:CC	organellar ribosome	FALSE
0.02969525	48	977	11	0.011258956	0.229166667	GO:0005761	GO:CC	mitochondrial ribosome	FALSE
0.032562163	12	977	5	0.005117707	0.416666667	GO:0005839	GO:CC	proteasome core complex	TRUE
0.032562163	12	977	5	0.005117707	0.416666667	GO:0030867	GO:CC	rough endoplasmic reticulum membrane	FALSE
0.03486828	4	977	3	0.003070624	0.75	GO:0097413	GO:CC	Lewy body	FALSE
0.03486828	366	977	47	0.048106448	0.128415301	GO:0030659	GO:CC	cytoplasmic vesicle membrane	FALSE
0.035940524	36	977	9	0.009211873	0.25	GO:0030666	GO:CC	endocytic vesicle membrane	FALSE
0.035940524	36	977	9	0.009211873	0.25	GO:0000421	GO:CC	autophagosome membrane	FALSE
0.036154073	104	977	18	0.018423746	0.173076923	GO:0044391	GO:CC	ribosomal subunit	FALSE
0.037713099	8	977	4	0.004094166	0.5	GO:0005665	GO:CC	RNA polymerase II, core complex	TRUE
0.037713099	8	977	4	0.004094166	0.5	GO:0044753	GO:CC	amphisome	TRUE
0.039829537	529	977	63	0.064483112	0.119092628	GO:0098796	GO:CC	membrane protein complex	FALSE
9.30E-09	3253	977	367	0.375639713	0.112818936	GO:0005515	GO:MF	protein binding	TRUE
0.001151349	316	977	52	0.053224156	0.164556962	GO:0008134	GO:MF	transcription factor binding	FALSE
0.003115364	1115	977	134	0.137154555	0.120179372	GO:0019899	GO:MF	enzyme binding	FALSE
0.003115364	266	977	44	0.045035824	0.165413534	GO:0140297	GO:MF	DNA-binding transcription factor binding	FALSE
0.007553664	387	977	56	0.057318321	0.144702842	GO:0019900	GO:MF	kinase binding	FALSE
0.007553664	96	977	21	0.021494371	0.21875	GO:0003735	GO:MF	structural constituent of ribosome	TRUE
0.007553664	189	977	33	0.033776868	0.174603175	GO:0044389	GO:MF	ubiquitin-like protein ligase binding	FALSE
0.009434442	176	977	31	0.031729785	0.176136364	GO:0031625	GO:MF	ubiquitin protein ligase binding	FALSE
0.023994994	344	977	49	0.050153531	0.14244186	GO:0019901	GO:MF	protein kinase binding	FALSE
0.024912077	57	977	14	0.01432958	0.245614035	GO:0003730	GO:MF	mRNA 3'-UTR binding	TRUE
0.024912077	320	977	46	0.047082907	0.14375	GO:0019904	GO:MF	protein domain specific binding	FALSE
0.035688077	68	977	15	0.015353122	0.220588235	GO:0043130	GO:MF	ubiquitin binding	FALSE
0.035688077	453	977	59	0.060388946	0.130242826	GO:0001067	GO:MF	transcription regulatory region nucleic acid binding	FALSE
0.035688077	170	977	28	0.028659161	0.164705882	GO:0001216	GO:MF	DNA-binding transcription activator activity	TRUE
0.035688077	82	977	17	0.017400205	0.207317073	GO:0032182	GO:MF	ubiquitin-like protein binding	FALSE
0.035688077	452	977	59	0.060388946	0.130530973	GO:0000976	GO:MF	transcription cis-regulatory region binding	TRUE
0.037435314	56	977	13	0.013306039	0.232142857	GO:0140375	GO:MF	immune receptor activity	TRUE

0.037435314	383	977	51	0.052200614	0.133159269	GO:0000977	GO:MF	RNA polymerase II transcription regulatory region sequence-specific DNA binding	FALSE
0.037435314	567	977	70	0.071647902	0.12345679	GO:0043565	GO:MF	sequence-specific DNA binding	TRUE
0.037435314	165	977	27	0.027635619	0.163636364	GO:0001228	GO:MF	DNA-binding transcription activator activity, RNA polymerase II-specific	FALSE
0.037435314	56	977	13	0.013306039	0.232142857	GO:0019955	GO:MF	cytokine binding	FALSE
0.037435314	993	977	112	0.114636643	0.112789527	GO:0042802	GO:MF	identical protein binding	FALSE
0.038239539	395	977	52	0.053224156	0.13164557	GO:0060089	GO:MF	molecular transducer activity	FALSE
0.038239539	64	977	14	0.01432958	0.21875	GO:0001221	GO:MF	transcription coregulator binding	FALSE
0.038239539	395	977	52	0.053224156	0.13164557	GO:0038023	GO:MF	signaling receptor activity	FALSE
0.042428788	350	977	47	0.048106448	0.134285714	GO:0000987	GO:MF	cis-regulatory region sequence-specific DNA binding	FALSE
0.045969304	179	977	28	0.028659161	0.156424581	GO:0003729	GO:MF	mRNA binding	FALSE
0.048246963	578	977	70	0.071647902	0.121107266	GO:0044877	GO:MF	protein-containing complex binding	TRUE
0.048246963	335	977	45	0.046059365	0.134328358	GO:0000978	GO:MF	RNA polymerase II cis-regulatory region sequence-specific DNA binding	FALSE
8.49E-14	4554	977	503	0.514841351	0.11045235	KEGG:00000	KEGG	KEGG root term	FALSE
2.30E-06	164	977	37	0.037871034	0.225609756	KEGG:05020	KEGG	Prion disease	FALSE
2.30E-06	111	977	29	0.029682702	0.261261261	KEGG:05152	KEGG	Tuberculosis	FALSE
2.55E-06	289	977	53	0.054247697	0.183391003	KEGG:05022	KEGG	Pathways of neurodegeneration - multiple diseases	FALSE
2.55E-06	233	977	46	0.047082907	0.197424893	KEGG:05014	KEGG	Amyotrophic lateral sclerosis	FALSE
9.14E-06	163	977	35	0.035823951	0.214723926	KEGG:05012	KEGG	Parkinson disease	FALSE
1.86E-05	99	977	25	0.025588536	0.252525253	KEGG:04217	KEGG	Necroptosis	FALSE
2.47E-05	194	977	38	0.038894575	0.195876289	KEGG:05016	KEGG	Huntington disease	FALSE
0.00016699	171	977	33	0.033776868	0.192982456	KEGG:05166	KEGG	Human T-cell leukemia virus 1 infection	FALSE
0.000167248	66	977	18	0.018423746	0.272727273	KEGG:00190	KEGG	Oxidative phosphorylation	FALSE
0.000449511	105	977	23	0.023541453	0.219047619	KEGG:05164	KEGG	Influenza A	FALSE
0.00049332	92	977	21	0.021494371	0.22826087	KEGG:04145	KEGG	Phagosome	FALSE
0.000878983	229	977	38	0.038894575	0.165938865	KEGG:05010	KEGG	Alzheimer disease	FALSE
0.000916241	149	977	28	0.028659161	0.187919463	KEGG:05163	KEGG	Human cytomegalovirus infection	FALSE
0.001128224	51	977	14	0.01432958	0.274509804	KEGG:05221	KEGG	Acute myeloid leukemia	FALSE
0.001128224	192	977	33	0.033776868	0.171875	KEGG:04010	KEGG	MAPK signaling pathway	FALSE
0.001692031	47	977	13	0.013306039	0.276595745	KEGG:05140	KEGG	Leishmaniasis	FALSE
0.001923804	103	977	21	0.021494371	0.203883495	KEGG:04932	KEGG	Non-alcoholic fatty liver disease	FALSE
0.002792388	137	977	25	0.025588536	0.182481752	KEGG:04714	KEGG	Thermogenesis	FALSE
0.003128151	123	977	23	0.023541453	0.18699187	KEGG:05415	KEGG	Diabetic cardiomyopathy	FALSE
0.003128151	188	977	31	0.031729785	0.164893617	KEGG:05132	KEGG	Salmonella infection	FALSE
0.003646028	87	977	18	0.018423746	0.206896552	KEGG:05418	KEGG	Fluid shear stress and atherosclerosis	FALSE
0.003646028	52	977	13	0.013306039	0.25	KEGG:04657	KEGG	IL-17 signaling pathway	FALSE
0.003646028	87	977	18	0.018423746	0.206896552	KEGG:04137	KEGG	Mitophagy - animal	FALSE
0.003782399	142	977	25	0.025588536	0.176056338	KEGG:05169	KEGG	Epstein-Barr virus infection	FALSE
0.006163105	189	977	30	0.030706244	0.158730159	KEGG:04144	KEGG	Endocytosis	FALSE

0.006603614	36	977	10	0.010235415	0.277777778	KEGG:04061	KEGG	Viral protein interaction with cytokine and cytokine receptor	FALSE
0.007799377	37	977	10	0.010235415	0.27027027	KEGG:04612	KEGG	Antigen processing and presentation	FALSE
0.007799377	64	977	14	0.01432958	0.21875	KEGG:05146	KEGG	Amoebiasis	FALSE
0.008730351	119	977	21	0.021494371	0.176470588	KEGG:04621	KEGG	NOD-like receptor signaling pathway	FALSE
0.008730351	95	977	18	0.018423746	0.189473684	KEGG:05162	KEGG	Measles	FALSE
0.00954772	112	977	20	0.020470829	0.178571429	KEGG:05135	KEGG	Yersinia infection	FALSE
0.010360005	74	977	15	0.015353122	0.202702703	KEGG:04659	KEGG	Th17 cell differentiation	FALSE
0.010763902	130	977	22	0.022517912	0.169230769	KEGG:05208	KEGG	Chemical carcinogenesis - reactive oxygen species	FALSE
0.010763902	122	977	21	0.021494371	0.172131148	KEGG:05202	KEGG	Transcriptional misregulation in cancer	FALSE
0.011307531	131	977	22	0.022517912	0.167938931	KEGG:05167	KEGG	Kaposi sarcoma-associated herpesvirus infection	FALSE
0.011717318	140	977	23	0.023541453	0.164285714	KEGG:05171	KEGG	Coronavirus disease - COVID-19	FALSE
0.012873721	100	977	18	0.018423746	0.18	KEGG:04530	KEGG	Tight junction	FALSE
0.013406791	101	977	18	0.018423746	0.178217822	KEGG:04060	KEGG	Cytokine-cytokine receptor interaction	FALSE
0.013406791	85	977	16	0.016376663	0.188235294	KEGG:04668	KEGG	TNF signaling pathway	FALSE
0.013406791	101	977	18	0.018423746	0.178217822	KEGG:04142	KEGG	Lysosome	FALSE
0.017838324	72	977	14	0.01432958	0.194444444	KEGG:04670	KEGG	Leukocyte transendothelial migration	FALSE
0.019605189	18	977	6	0.006141249	0.333333333	KEGG:00030	KEGG	Pentose phosphate pathway	FALSE
0.019717599	24	977	7	0.00716479	0.291666667	KEGG:03060	KEGG	Protein export	FALSE
0.019717599	89	977	16	0.016376663	0.179775281	KEGG:03010	KEGG	Ribosome	FALSE
0.022989157	52	977	11	0.011258956	0.211538462	KEGG:05133	KEGG	Pertussis	FALSE
0.022989157	124	977	20	0.020470829	0.161290323	KEGG:04218	KEGG	Cellular senescence	FALSE
0.025938499	92	977	16	0.016376663	0.173913043	KEGG:04071	KEGG	Sphingolipid signaling pathway	FALSE
0.027792554	93	977	16	0.016376663	0.172043011	KEGG:04380	KEGG	Osteoclast differentiation	FALSE
0.027792554	144	977	22	0.022517912	0.152777778	KEGG:05170	KEGG	Human immunodeficiency virus 1 infection	FALSE
0.030345949	94	977	16	0.016376663	0.170212766	KEGG:05160	KEGG	Hepatitis C	FALSE
0.03039893	33	977	8	0.008188332	0.242424242	KEGG:05321	KEGG	Inflammatory bowel disease	FALSE
0.03039893	40	977	9	0.009211873	0.225	KEGG:04721	KEGG	Synaptic vesicle cycle	FALSE
0.030665883	103	977	17	0.017400205	0.165048544	KEGG:04210	KEGG	Apoptosis	FALSE
0.032194617	87	977	15	0.015353122	0.172413793	KEGG:03082	KEGG	ATP-dependent chromatin remodeling	FALSE
0.034064109	96	977	16	0.016376663	0.166666667	KEGG:04613	KEGG	Neutrophil extracellular trap formation	FALSE
0.039621884	42	977	9	0.009211873	0.214285714	KEGG:04330	KEGG	Notch signaling pathway	FALSE
0.040031031	35	977	8	0.008188332	0.228571429	KEGG:03050	KEGG	Proteasome	FALSE
0.040598226	133	977	20	0.020470829	0.15037594	KEGG:05203	KEGG	Viral carcinogenesis	FALSE
0.04541744	36	977	8	0.008188332	0.222222222	KEGG:05134	KEGG	Legionellosis	FALSE
0.04541744	29	977	7	0.00716479	0.24137931	KEGG:04962	KEGG	Vasopressin-regulated water reabsorption	FALSE
0.046509823	315	977	39	0.039918117	0.123809524	KEGG:05200	KEGG	Pathways in cancer	FALSE
0.049002533	210	977	28	0.028659161	0.133333333	KEGG:05165	KEGG	Human papillomavirus infection	FALSE
0.049467161	60	977	11	0.011258956	0.183333333	KEGG:04658	KEGG	Th1 and Th2 cell differentiation	FALSE
0.049467161	76	977	13	0.013306039	0.171052632	KEGG:04625	KEGG	C-type lectin receptor signaling pathway	FALSE
0.049467161	52	977	10	0.010235415	0.192307692	KEGG:05323	KEGG	Rheumatoid arthritis	FALSE

0.049467161	93	977	15	0.015353122	0.161290323	KEGG:04936	KEGG	Alcoholic liver disease	FALSE
