# Supplementary material for: Signatures of Radiation‐Induced Stress and Putative Selection on Immune Targets in Chornobyl Wolves
Source: Mol Ecol. 2026 Apr 28;35:e70308. doi: 10.1111/mec.70308 (PMC13123633; doi:10.1111/mec.70308)
Supplement: Supplementary file 2 — Data S2: mec70308‐sup‐0002‐DataS2.pdf. [file MEC-35-e70308-s003.pdf]

Total Dose Correlated Genes - GO Enrichment									
p value	*term size*	*query size*	*overlap size*	*precision*	*recall*	*term id*	*source*	*term name*	*highlighted*
0.00466771	4554	336	169	0.5029762	0.03711023	KEGG:00000	KEGG	KEGG root term	FALSE
0.0060912	77	336	10	0.0297619	0.12987013	KEGG:04064	KEGG	NF-kappa B signaling pathway	FALSE
0.0060912	25	336	6	0.0178571	0.24	KEGG:05340	KEGG	Primary immunodeficiency	FALSE
0.01546657	142	336	13	0.0386905	0.0915493	KEGG:05169	KEGG	Epstein-Barr virus infection	FALSE
0.02835282	37	336	6	0.0178571	0.16216216	KEGG:04612	KEGG	Antigen processing and presentation	FALSE
0.02835282	119	336	11	0.0327381	0.09243697	KEGG:04621	KEGG	NOD-like receptor signaling pathway	FALSE
0.03540979	206	336	15	0.0446429	0.07281553	KEGG:05168	KEGG	Herpes simplex virus 1 infection	FALSE
0.03947579	57	336	7	0.0208333	0.12280702	KEGG:03008	KEGG	Ribosome biogenesis in eukaryotes	FALSE
0.04435422	76	336	8	0.0238095	0.10526316	KEGG:04625	KEGG	C-type lectin receptor signaling pathway	FALSE
