# Supplementary material for: Signatures of Radiation‐Induced Stress and Putative Selection on Immune Targets in Chornobyl Wolves
Source: Mol Ecol. 2026 Apr 28;35:e70308. doi: 10.1111/mec.70308 (PMC13123633; doi:10.1111/mec.70308)
Supplement: Supplementary file 3 — Data S3: mec70308‐sup‐0003‐DataS3.pdf. [file MEC-35-e70308-s002.pdf]

Extreme PBE Outlier - GO Enrichment								
p value	*term size*	*query size*	*overlap size*	*precision*	*recall*	*term id*	*source*	*term name*
0.014041635	27	9	4	0.4444444	0.148148148	GO:0005938	GO:CC	cell cortex
0.032078988	22	9	3	0.3333333	0.136363636	KEGG:04015	KEGG	Rap1 signaling pathway
0.032078988	4	9	2	0.2222222	0.5	KEGG:04310	KEGG	Wnt signaling pathway
0.032078988	5	9	2	0.2222222	0.4	KEGG:04972	KEGG	Pancreatic secretion
0.032078988	36	9	4	0.4444444	0.111111111	KEGG:05132	KEGG	Salmonella infection
0.044312609	7	9	2	0.2222222	0.285714286	KEGG:04928	KEGG	oid hormone synthesis, secretion ar
0.048956098	8	9	2	0.2222222	0.25	KEGG:04961	KEGG	ınd other factor-regulated calcium r

highlighted
TRUE
FALSE
FALSE
FALSE
FALSE
FALSE
FALSE
