# Supplementary material for: Signatures of Radiation‐Induced Stress and Putative Selection on Immune Targets in Chornobyl Wolves
Source: Mol Ecol. 2026 Apr 28;35:e70308. doi: 10.1111/mec.70308 (PMC13123633; doi:10.1111/mec.70308)
Supplement: Supplementary file 4 — Figure S1: mec70308‐sup‐0004‐AppendixS1.pdf. Figure S2: mec70308‐sup‐0004‐AppendixS1.pdf. Figure S3: mec70308‐sup‐0004‐AppendixS1.pdf. Figure S4: mec70308‐sup‐0004‐AppendixS1.pdf. Figure S5: mec70308‐sup‐0004‐AppendixS1.pdf. Figure S6: mec70308‐sup‐0004‐AppendixS1.pdf. Figure S7: mec70308‐sup‐0004‐AppendixS1.pdf. Figure S8: mec70308‐sup‐0004‐AppendixS1.pdf. Figure S9: mec70308‐sup‐0004‐AppendixS1.pdf. Table S1: mec70308‐sup‐0004‐AppendixS1.pdf. Table S2: mec70308‐sup‐0004‐AppendixS1.pdf. Table S3: mec70308‐sup‐0004‐AppendixS1.pdf. Table S4: mec70308‐sup‐0004‐AppendixS1.pdf. Table S5: mec70308‐sup‐0004‐AppendixS1.pdf. [file MEC-35-e70308-s001.pdf]

MOLECULAR ECOLOGY

Supplemental Information for:

Signatures of radiation-induced stress and putative selection on immune targets in Chornobyl wolves

Cara N Love, Stacey L Lance, Nicolas Rochette, Thomas G Hinton, James C Beasley, Dmitry Shamovich, Michael E Byrne, Brian Nadel, Sarah C Webster, Shane C Campbell-Staton

Additional Sample Collection and Demography Details

To test for demographic differences in age, we performed a *t*-test with the *stats* (v4.2.1) package in R between the CEZ and BLR sites. Then to compare sex differences between sites, we performed Fisher's exact test with the *stats* package in R. No significant difference was observed for either characterization (age; t(13.37) = -1.03, P = 0.31, sex; P = 1). We additionally calculated individual relatedness (ϕ) using the relatedness2 option in VCFtools (Danecek et al., 2011). Individuals from CEZ appear to have higher relatedness than individuals from BLR (Fig. S6). Given the reduced wolf home range size observed within the CEZ, this could suggest decreased familial dispersal from the CEZ, though further examination is needed to definitively say.

To examine population connectivity and structure, we filter the SNP dataset for 'silent' variants, as described in the main manuscript. Here we also present ADMIXTURE and PCA assessment across all SNPs identified within the wolf blood transcriptome. We do not filter for 'silent' variants yet still filter for linkage disequilibrium in the same manner as described previously, filtering parameters (--indep-pairwise 50 10 0.1) in PLINK (Purcell et al., 2007). LD filtering of the whole SNP dataset results in 5,005 SNPs. Resulting population structure assessment is presented in Fig. S6. Finally, we examine the impacts of missing data on PCA of genetic diversity as this could contribute to observed differences between populations. We filtered for 0% missing data using vcftools (--max-missing 1) (Danecek et al., 2011) and performed PCA in PLINK as described previously. These approaches do not alter the main take aways and figures can be found in Fig. S5.

We additionally investigated admixture history and gene flow in the 5 gray wolf study groups with F statistics in ADMIXTOOLS 2 package in R (Maier et al., 2023). We utilized the LD filtered 'silent' SNP dataset, computed f2 statistics between each wolf site pair and estimated demographic models with f4 while implementing *qpGraph* with two and three migration events, Yellowstone set as the outgroup, and 250 iterations. The resulting best fit models varied depending on run and admixture events, however consensus assessment of *qpGraph* phylogeny with migration events shows a large proportion of CEZ and BLR ancestry are shared, with gene flow between BLR than CEZ (Fig. S7).

MOLECULAR ECOLOGY

PCA of genetic diversity (Fig 2) describes one sample more diverged along the PC1 axis. To help examine whether this is due to true genetic diversity, we confirmed lack of bacterial contamination using Kraken (Wood and Salzberg, 2014), examined transcriptome expression with pairwise correlations between samples to identify outlier samples (see WGCNA methods) and observed no further outlier behavior of this individual in subsequent gene expression analyses, etc.

We assessed genetic relatedness using vcftools (--relatedness2) and present results in Fig. S8.

Dose rate quantification and characterization

Radioactive emissions from the Chornobyl accident were composed of a complex mixture of radionuclides with physical half-lives that varied from seconds to thousands of years (*1*). Acute dose rates from radiation were greatest during the first year of the accident when short-lived radionuclides were still present. Radioactive decay of the short-lived isotopes has resulted in dose rates now being < 1% of the original (*1*). The chronic dose rates currently experienced by biota are dominated by ^{90}Sr and ^{137}Cs (Beresford et al., 2020), radioisotopes with half-lives of 28 and 30 years, respectively.

Determining radiation dose rates to biota is challenging because there are internal and external components that need to be considered. At Chornobyl, internal doses to biota occur from the intake of ^{90}Sr and ^{137}Cs contaminated food, water, or air. In contrast, external doses occur because wildlife are irradiated, as they traverse through their home ranges, by radioactive components of their environments (e.g., soil, vegetation, litter). Strontium-90 emits a beta particle that can contribute to internal doses but lacks sufficient energy to be a major contributor to external dose. ^{137}Cs, however, emits a relatively high energy gamma photon that contributes to both the internal and external dose received by Chornobyl wildlife.

Dose rates are seldom measured on free-ranging wildlife. Instead, dose rates are typically derived from soil radioactivity concentrations (Stark et al., 2017). Concentration Ratios (CRs) are used to convert soil radioactivity concentrations (Bq/kg) to radioactivity concentrations in animal tissues (Bq/kg). Dose Coefficients (DCs) are then applied to convert radioactivity concentrations in the animal to a dose rate (μGy/h) from internal exposures. Similarly, DCs are used to convert soil contamination levels to dose rates from external exposures. Empirical ratios, such as CRs and DCs, are radionuclide- and organism-specific and have been tabulated as well as incorporated into commonly used wildlife dose models (e.g., ERICA Tool (Brown et al., 2016); BiotaDC (Ulanovsky et al., 2017)). Estimating dose rates with empirical ratios is a pragmatic solution to a complex problem, but one that generates massive uncertainties. Typically, CRs vary over several orders of magnitude (Agency, 2010) and DCs do not account for the spatial-temporal aspects of external radiation exposures to free-ranging wildlife (Bontrager et al., 2024). Our ^{137}Cs dose rate estimates of Chornobyl wolves measure both internal and external components derived from direct measurements on each animal, rather than

through empirical ratios. We additionally provide measures of total dose, combining these ^{137}Cs dose rate estimates with ^{90}Sr internal dose rates for all CEZ wolves (table S1).

To quantify dose rates for each CEZ wolf, our ^{137}Cs dose rate estimates integrate both the internal and external components derived from direct measurements on each animal, rather than through empirical ratios. External dose rates were quantified with a GPS-dosimeter attached to each animal at the time of capture (Hinton et al., 2019). We tracked each wolf from 165 to 180 days, with GPS locations and radiation exposure readings transmitted every 35 min, resulting in ~6600 individual locations and ^{137}Cs external dose rates per wolf (Hinton et al., 2019). The average external dose rate (μGy/h) was then used as a component in the total dose rate for each wolf (Table S1). To measure each animal's internal ^{137}Cs contamination level (Bq/kg; Table S1), we utilized a calibrated 1-cm^3 Cadmium-Zinc-Telluride (CZT) radiation detector system, as described in (Hinton et al., 2019). This detector was operated by a portable computer and was placed under the animal's flank, while the animal was anesthetized, to quantify activity concentrations (Bq/kg) of ^{137}Cs in each animal. A ^{137}Cs dose coefficient of 2.7e-4 was obtained from ICRP (Ulanovsky et al., 2017), based on an average wolf mass of 35 kg, and used to convert internal ^{137}Cs activity concentrations to internal dose rates (μGy/h; Table S1).

To quantify internal ^{137}Cs concentrations from wolves in BLR, we analysed lyophilized and homogenized muscle tissues from each individual. We performed laboratory analyses of ^{137}Cs using a Packard Cobra II auto-gamma counter (Model Cobra II 5003; Packard Instruments Co., Meriden, CT, USA). We used an ROI of 580-754 keV and conducted auto-calibration daily during the sample analysis using a traceable ^{137}Cs source (SREL-0113). We derived counter yield from matrix-specific standards as described in (Kennamer et al., 2017) and conducted background corrected ^{137}Cs counts on each sample. To assess minimum detectable concentrations (MDCs, Bq/g, dry mass) for each sample, we followed previously described methods (Currie, 1968). Lastly, we converted dry activity concentrations (Bq/g, dry weight) to wet activity concentrations (Bq/g, wet mass) using wet:dry tissue mass ratios. To convert internal activity concentrations to dose rates (μGy/h; Table S1), the ^{137}Cs internal dose coefficients used for the CEZ wolves were used on wolves from BLR. Furthermore, to characterize external dose rates for BLR wolves we derived rates from ^{137}Cs soil contamination maps for northern Belarus (Guermentchuk et al., n.d.) using an external dose coefficient of 1.1e-4 (Ulanovsky et al., 2017).

Characterization of Co-expression Modules

We described co-expression models with Weighted Gene Co-expression Network Analysis (WGCNA), using the WGCNA package in R (Langfelder and Horvath, 2008) and whole blood transcriptomes of wolves from CEZ (2014 individuals only) and BLR sites, as well as previously published data from geographically removed wolves from North American (YLS; (Charruau et al., 2016)). Prior to running WGCNA we implemented gene filtering for average read count >10, and log normalized all counts using the *cpm* command in edgeR (Robinson et al., 2009). We additionally identified and filtered outlier samples utilizing pairwise correlations between samples. Two outlier samples which exhibited low mean correlation with other samples, GB5 and SRR3402519, were removed from further analyses (Fig S1A).

MOLECULAR ECOLOGY

While performing WGCNA, we approximated a scale free topological network with a soft thresholding approach to compare an adjacency matrix, and utilized a power of 15 to construct co-expression modules (Fig. S1B). We then performed topological overlap to create a cluster dendrogram with signed Pearson correlations while implementing a minimum cluster size of 40 genes and merging closely correlated modules (R^2 = 0.95, Fig S1B). We utilized student asymptotic p-value for correlation using WGCNA's *corPvalueStudent* function to examine trait:module relationships.

To characterize gene ontology categories for each module we performed gene otology enrichment with gprofiler2 (v0.2.2) (Kolberg et al., 2020), implementing an unordered query while filtering for only significant results using FDR correction for multiple tests (corrected P <0.05), and a custom background of the wolf blood transcriptome data (Data S1).

Putative Genes Under Selection and Candidate Gene Significance

In identifying candidate genes under selection along the Chornobyl lineage, we utilized the population branch excess (PBE (Shpak et al., 2024)) test, which allows us to characterize significant increases in branch length along the focal lineage as compared to expected branch length. We applied a conservative significance threshold (PBE FDR- corrected -log10(p) > 6) to identify candidate genes under selection and characterized the three most highly diverged SNPs, falling within two putative genes under selection. The significance values for these gene targets are as follows: EMC6 corrected P < 9.38e-15 and APBB1IP corrected P = 2.62e-13. Functional annotation of putative SNPs under selection was performed with SNPeff (Cingolani et al., 2012).

References

1. Hinton TG, Byrne ME, Webster SC, Love CN, Broggio D, Trompier F, et al. GPS-coupled contaminant monitors on free-ranging Chornobyl wolves challenge a fundamental assumption in exposure assessments. Environment International. 2019;133: 105152. doi:10.1016/j.envint.2019.105152
2. Gipson PS, Ballard WB, Nowak RM, Mech LD. Accuracy and Precision of Estimating Age of Gray Wolves by Tooth Wear. The Journal of Wildlife Management. 2014;64: 752–758.
3. Beresford NA, Barnett CL, Gashchak S, Maksimenko A, Guliaichenko E, Wood MD, et al. Radionuclide transfer to wildlife at a 'Reference site' in the Chornobyl Exclusion Zone and resultant radiation exposures. Journal of Environmental Radioactivity. 2020;211: 105661. doi:10.1016/j.jenvrad.2018.02.007
4. Stark K, Goméz-Ros JM, Vives i Batlle J, Lindbo Hansen E, Beaugelin-Seiller K, Kapustka LA, et al. Dose assessment in environmental radiological protection: State of the art and perspectives. Journal of Environmental Radioactivity. 2017;175–176: 105–114. doi:10.1016/j.jenvrad.2017.05.001
5. Brown JE, Alfonso B, Avila R, Beresford NA, Copplestone D, Hosseini A. A new version of the ERICA tool to facilitate impact assessments of radioactivity on wild plants and animals. Journal of Environmental Radioactivity. 2016;153: 141–148. doi:10.1016/j.jenvrad.2015.12.011

6. Ulanovsky A, Copplestone D, Vives I Batlle J. ICRP Publication 136: Dose Coefficients for Non-human Biota Environmentally Exposed to Radiation - A. Ulanovsky, D. Copplestone, J. Vives i Batlle, 2017. Annals of the ICRP. 2017;46. doi:10.1177/0146645317728022
7. Agency IAE. Handbook of Parameter Values for the Prediction of Radionuclide Transfer in Terrestrial and Freshwater Environments. Handbook of Parameter Values for the Prediction of Radionuclide Transfer in Terrestrial and Freshwater Environments. International Atomic Energy Agency; 2010 pp. 1–194. Available: https://www.iaea.org/publications/8201/handbook-of-parameter-values-for-the-prediction-of-radionuclide-transfer-in-terrestrial-and-freshwater-environments
8. Bontrager HL, Hinton TG, Okuda K, Beasley JC. The impact of sampling scale: A comparison of methods for estimating external contaminant exposure in free-ranging wildlife. Science of The Total Environment. 2024; 171012. doi:10.1016/j.scitotenv.2024.171012
9. Guermentchuk MG, Zhukova OM, Shagalova ED, Matveenko II. RADIOECOLOGICAL MAPPING OF THE TERRITORY OF BELARUS ON THE BASE INFORMATION OF RADIATION MONITORING.
10. Langfelder P, Horvath S. WGCNA: An R package for weighted correlation network analysis. BMC Bioinformatics. 2008;9. doi:10.1186/1471-2105-9-559
11. Charruau P, Johnston R, Stahler DR, Lea AJ, Snyder-Mackler N, Smith DW, et al. Pervasive Effects of Aging on Gene Expression in Wild Wolves. Molecular Biology and Evolution. 2016; 1–18.
12. Robinson MD, McCarthy DJ, Smyth GK. edgeR: A Bioconductor package for differential expression analysis of digital gene expression data. Bioinformatics. 2009;26: 139–140. doi:10.1093/bioinformatics/btp616
13. Kolberg L, Raudvere U, Kuzmin I, Vilo J, Peterson H. gprofiler2 -- an R package for gene list functional enrichment analysis and namespace conversion toolset g:Profiler. F1000Res. 2020;9: ELIXIR-709. doi:10.12688/f1000research.24956.2
14. R Core Team. R: A Language and Environment for Statistical Computing. Vienna, Austria: R Foundation for Statistical Computing; 2024. Available: https://www.R-project.org/
15. Shpak M, Lawrence KN, Pool JE. The Precision and Power of Population Branch Statistics in Identifying the Genomic Signatures of Local Adaptation. bioRxiv. 2024; 2024.05.14.594139. doi:10.1101/2024.05.14.594139
16. Cingolani P, Platts A, Wang LL, Coon M, Nguyen T, Wang L, et al. A program for annotating and predicting the effects of single nucleotide polymorphisms, SnpEff. Fly (Austin). 2012;6: 80–92. doi:10.4161/fly.19695

MOLECULAR ECOLOGY

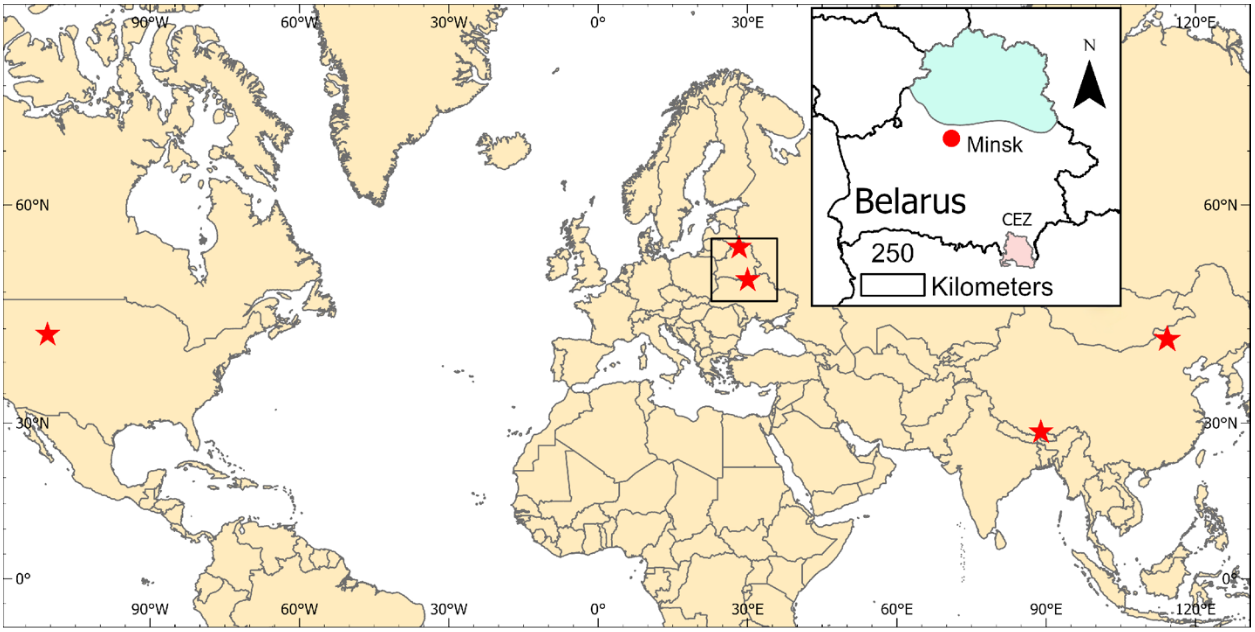

Fig. S1. Sample collection locations. Wolf RNAseq data was utilized from numerous regions around the globe. Stars represent sample collection locations: Chornobyl, N. Belarus, USA (Yellowstone), Inner Mongolia, and Tibet. The insert further details regions where samples were collected in N. Belarus (green) and the Chornobyl Exclusion Zone (CEZ; red).

MOLECULAR ECOLOGY

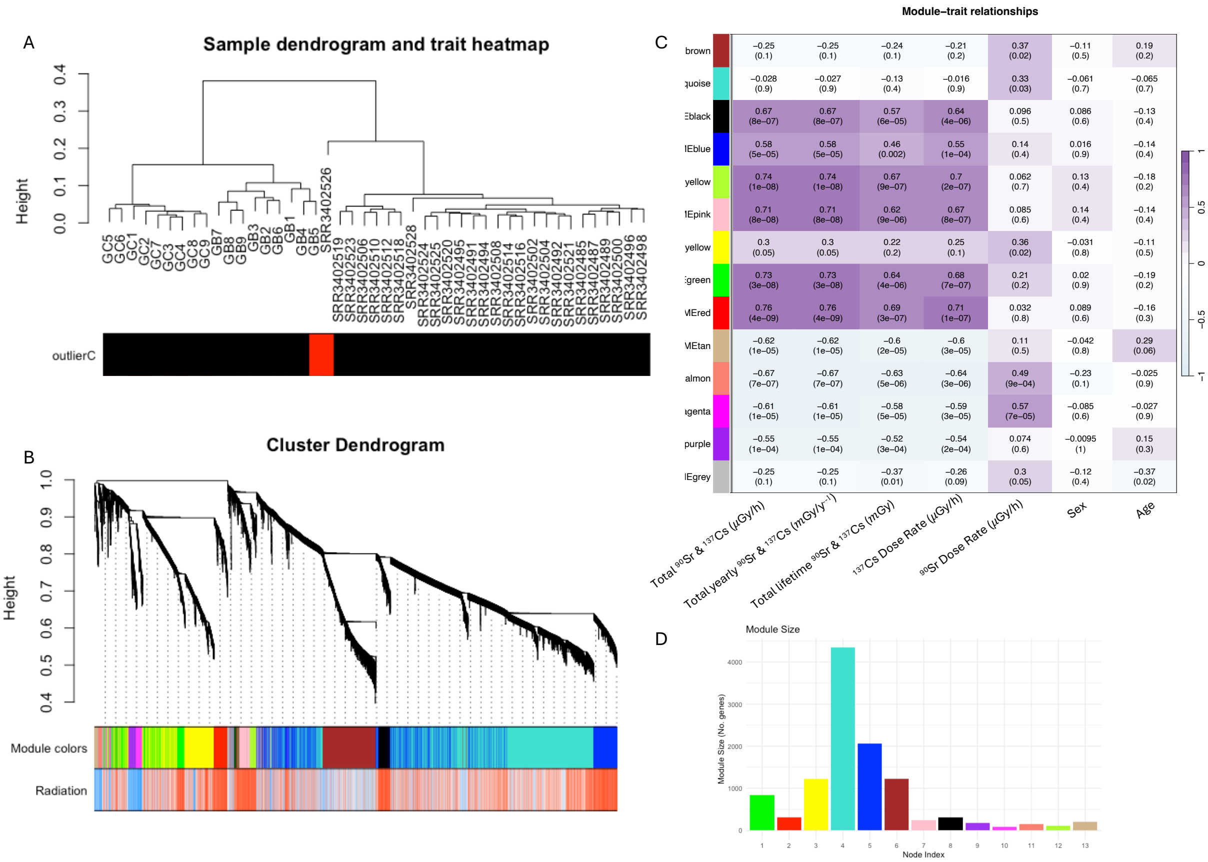

Fig. S2. WGCNA Module Characterization (A) Sample dendrogram depiction of mean pairwise correlation between all transcriptome samples utilized in creating co-regulatory modules with WGCNA. Individuals identified as outliers due to low mean correlation with other samples (denoted in red) were removed from further module analyses. (**B**) Cluster dendrogram of gene expression patterns within the gray wolf blood transcriptome identified through topological overlap. Each branch represents an individual gene. Closely correlated genes were merged into seven modules, characterized by the module colors and identified as modules 1-6. (**C**) Heatmap of module-trait correlations within Chornobyl and Belarus wolves**.** Correlation of each module's eigengene to each trait. Each cell contains the eigengene correlation value with each trait (top) and Pearson's R for significant correlations ($p < 0.05$, bottom). The bar to the right of the graph is color-coded by correlation, purple indicating a positive correlation and blue a negative correlation. (**D**) Number of genes per module.

MOLECULAR ECOLOGY

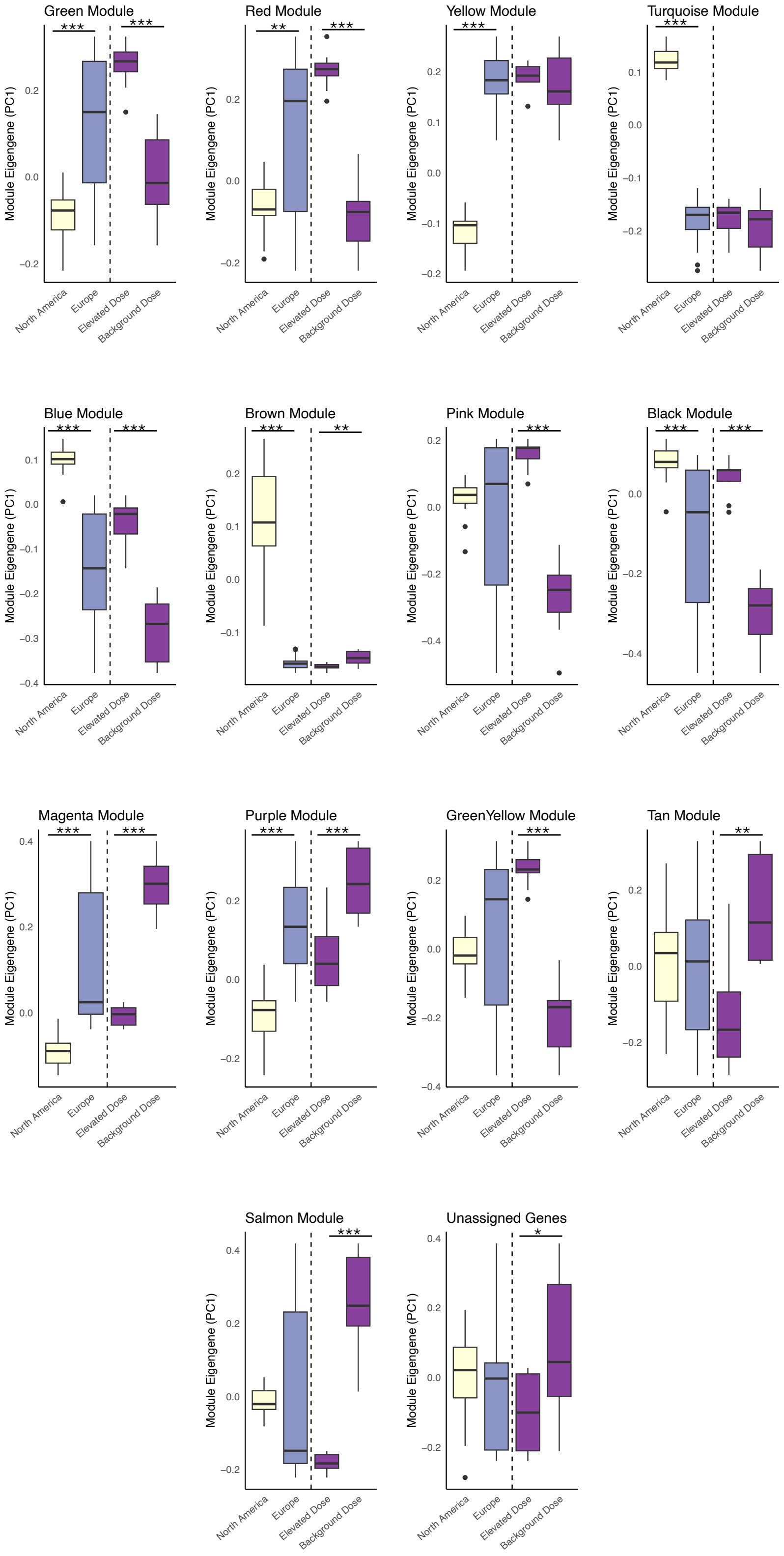

MOLECULAR ECOLOGY

Fig. S3. Identification of coregulatory modules associated with radiation exposure. Weighted gene correlation network analysis (WGCNA) of blood transcriptomes from Eastern Europe (*N* = 17) and North American (Yellowstone, USA; *N* = 26) wolves characterized thirteen regulatory modules. Ten of the thirteen modules are uniquely associated with population (Eastern European or North American), and of these modules, seven display significantly divergent module eigengene expression patterns significantly between elevated radiation exposed (CEZ = Chornobyl, *N* = 9) and background exposed (BLR = Belarus, *N* = 8) wolves from Eastern Europe.

MOLECULAR ECOLOGY

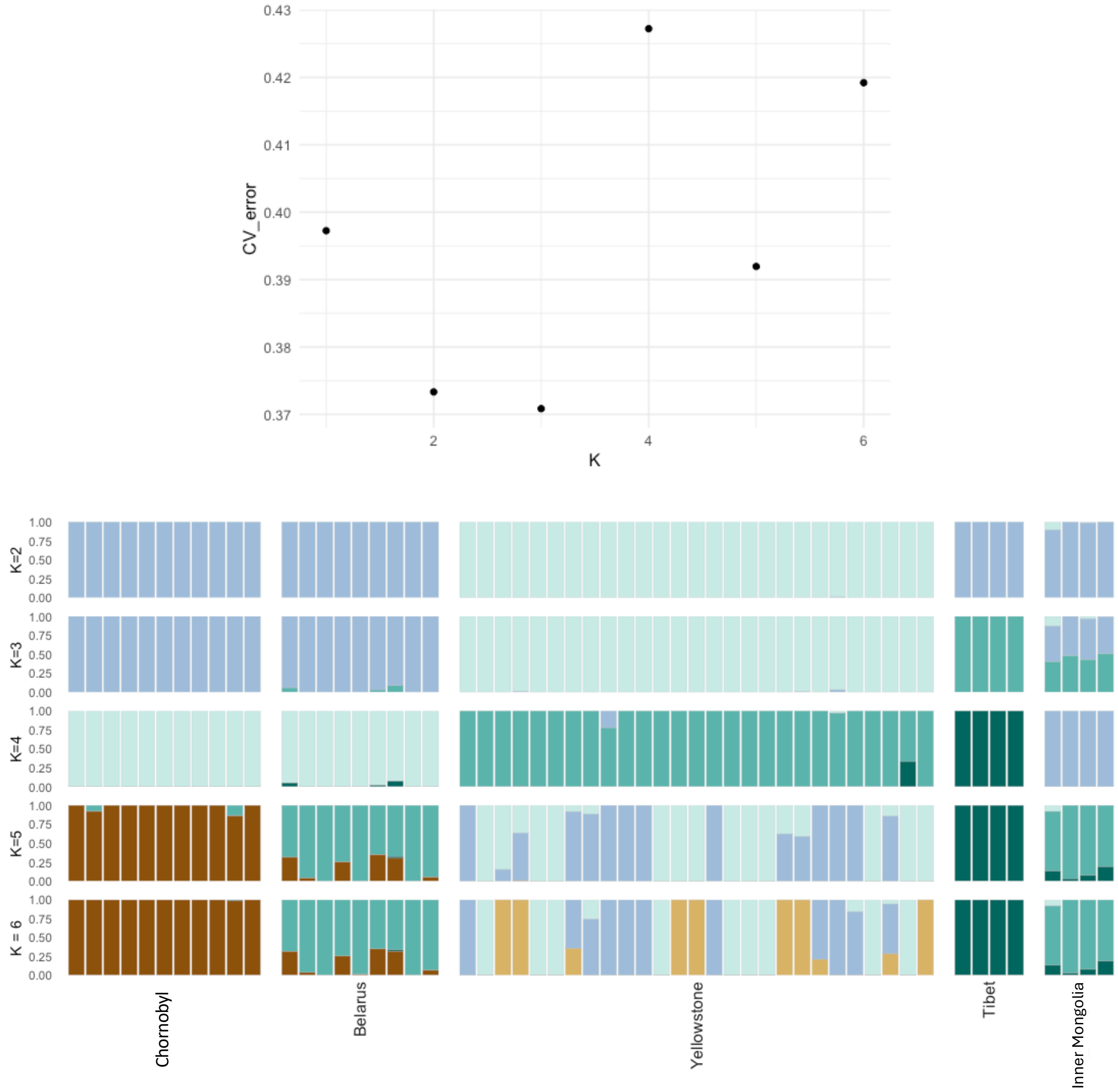

Fig. S4. ADMIXTURE analyses of Eastern European (Chornobyl and Belarus), Asian (Tibet and Mongolia), and North American (United States of America, Yellowstone) populations (K=2-6), with CV error (K=2-6) depicted above. Analyses performed on whole blood RNAseq SNPs filtered for synonymous SNPs.

MOLECULAR ECOLOGY

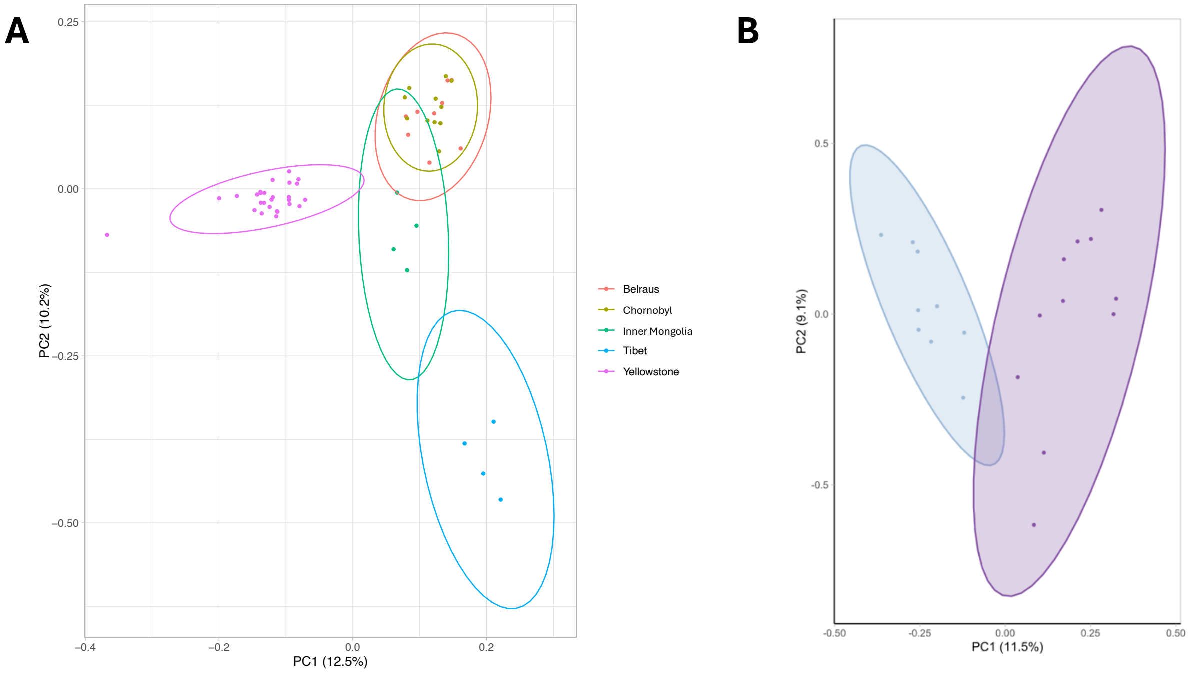

Fig. S5. PCA of genetic diversity **(A)** across Eastern European (Chornobyl and Belarus) and North American (United States of America, Yellowstone), and Asian (Tibet and Inner Mongolia) gray wolf populations using 0% missing synonymous SNPs. As well as **(B)** across Chornobyl (purple) and Belarus (Blue) with 0% missing synonymous SNPs

MOLECULAR ECOLOGY

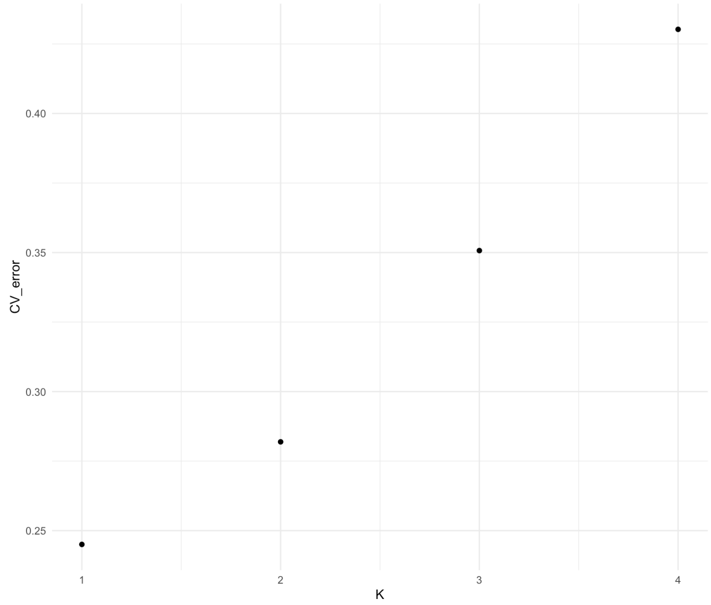

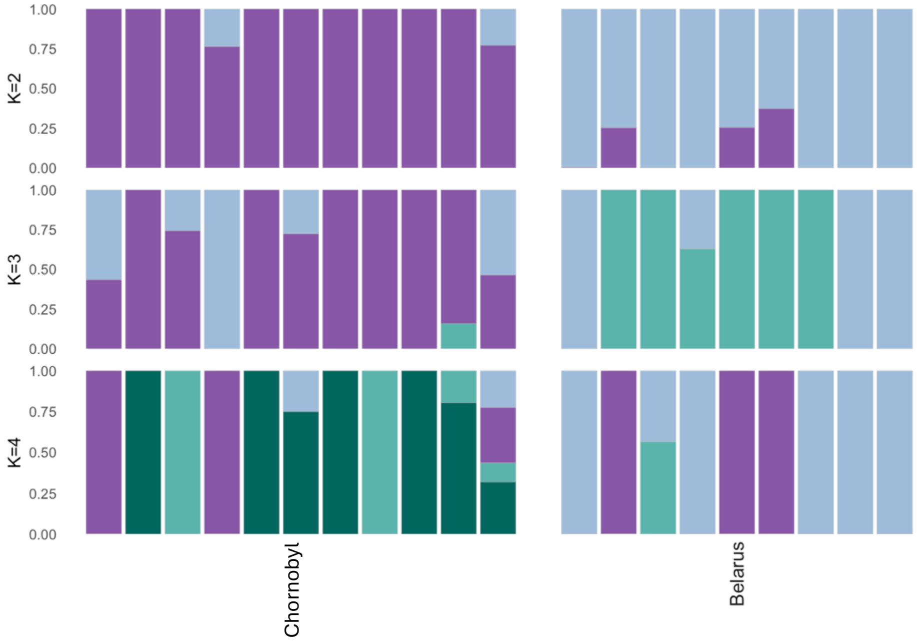

Fig. S6. ADMIXTURE analyses of Northern Belarus and Chornobyl wolf populations (K=2-4), with CV error (K=1-3).

MOLECULAR ECOLOGY

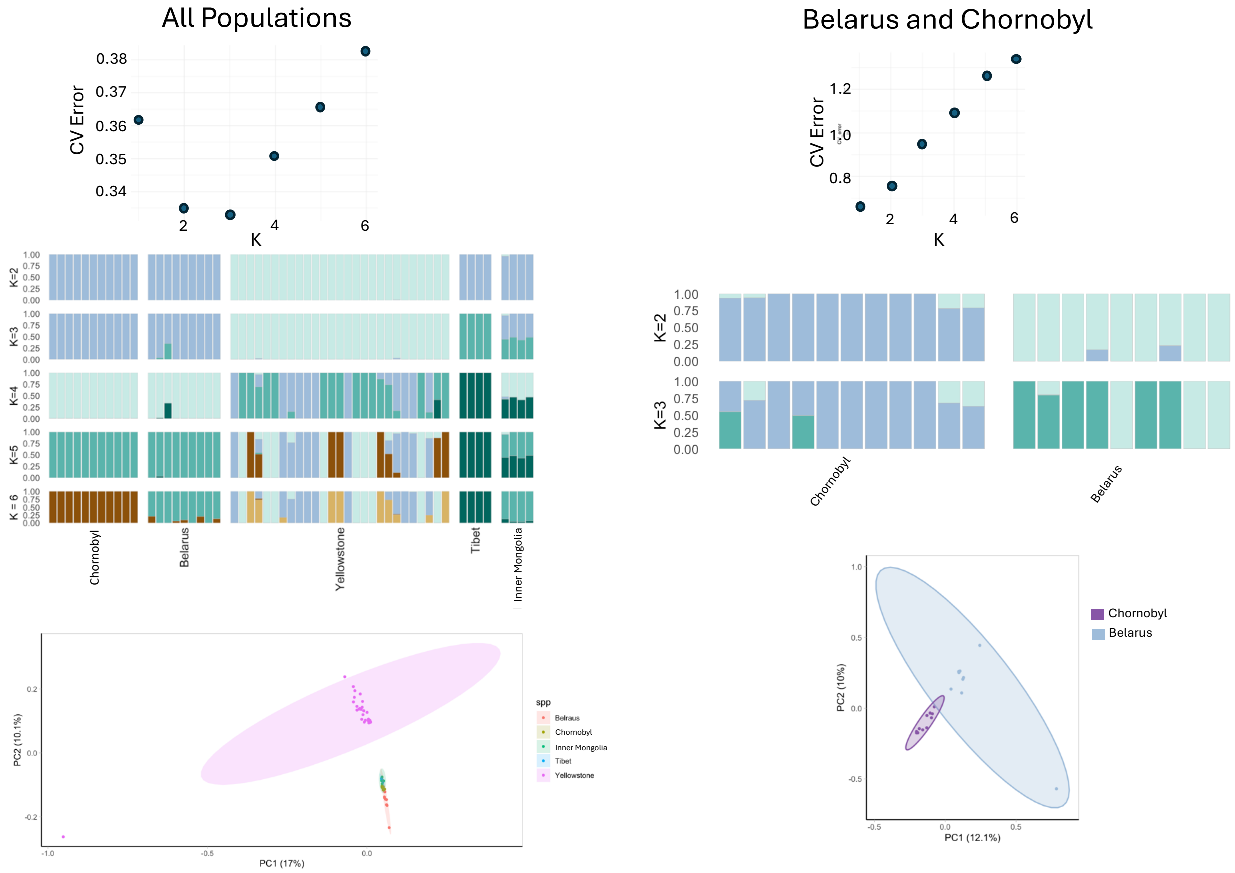

Fig. S7 Population structure assessed using all SNPs. Population structure analyses performed with all (synonymous and non-synonymous) SNPs from gray wolf whole blood transcriptomes, and filtered for LD.

MOLECULAR ECOLOGY

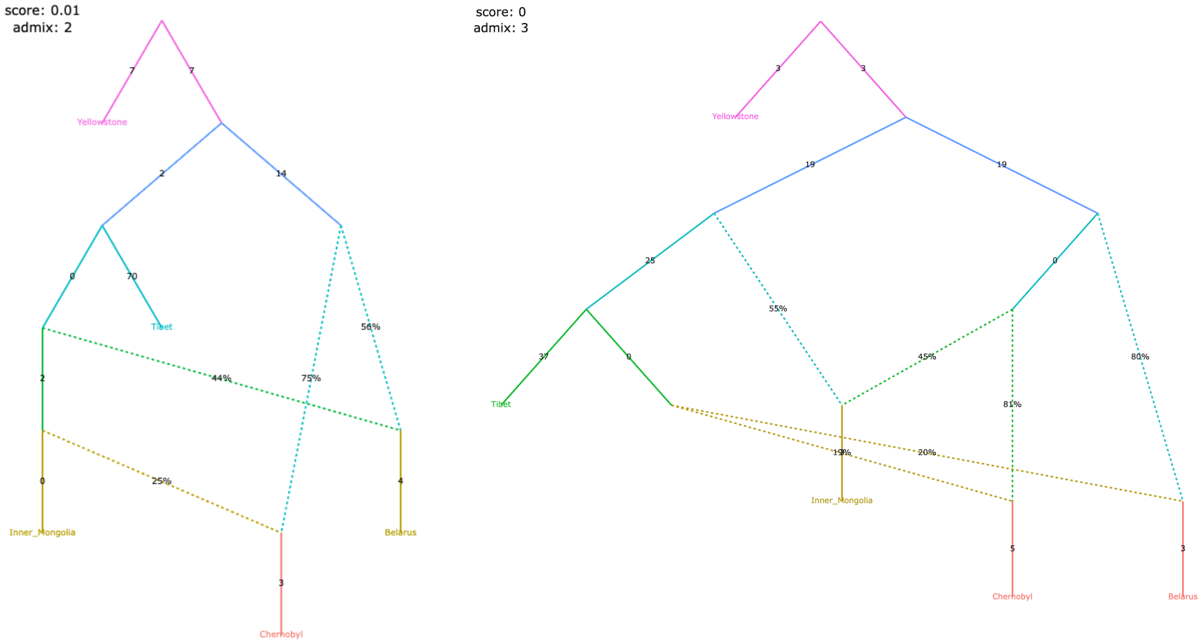

Fig. S8 Population gene flow and demographic history estimated with qpGraph in ADMIXTOOLS2. Solid lines represent genetic drift or genealogical descent and dashed lines represent admixture weights. Multiple graphs are presented to represent the variability in model fit and characterization with differing admixture events.

MOLECULAR ECOLOGY

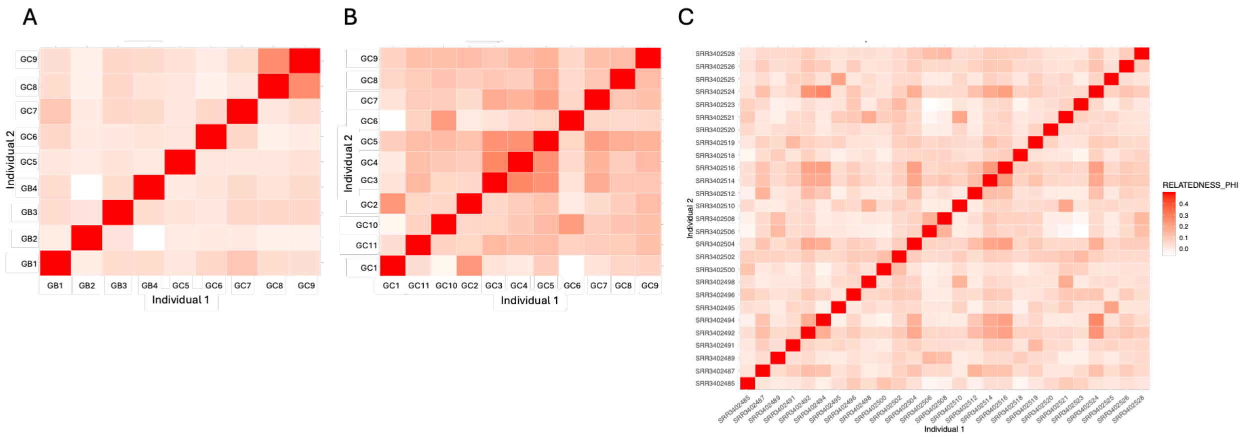

Fig. S9 Wolf relatedness. Wolf relatedness (ϕ) statistic quantifying the degree of genetic similarity between individuals from each site: (A) N. Belarus, (B) Chornobyl, and (C) Yellowstone.

MOLECULAR ECOLOGY

Table S1.

Summary of dose rate (µGy/h) and lifetime dose (mGy(Danecek et al., 2011)) data for Chornobyl wolves (C-#) and control animals from north of the Chornobyl Exclusion Zone in N. Belarus (B-#). Total Dose Rate, Total Year Dose, and Total Lifetime Dose (mGy) are calculated as combined (^{137}Cs + ^{90}Sr) internal + external dose for Chornobyl wolves, and total (internal + external) ^{137}Cs dose for Northern Belarus wolves.

Site	Wolf ID	^{137}Cs Internal Dose Rate (µGy/h)	^{137}Cs External Dose Rate (µGy/h)	^{90}Sr Internal Dose Rate (µGy/h)	Total (Internal + External) Dose Rate (µGy/h)	Total (Internal + External) Year Dose (mGy/y)	Total, Lifetime Dose (mGy)
Chornobyl	C-1	5	3.3	0.7	9	78.8	197
Chornobyl	C-2	3.8	4.1	1.8	9.6	84.5	127
Chornobyl	C-3	1.8	3.2	0.5	5.5	48.5	73
Chornobyl	C-4	3.9	3	1.1	8	70.1	105
Chornobyl	C-5	3	1.4	1	5.4	47.1	259
Chornobyl	C-6	0.8	0.3	0.2	1.3	11.5	17
Chornobyl	C-7	0.8	0.5	0.6	1.9	16.2	20
Chornobyl	C-8	0.7	0.9	0.4	2	17.8	62
Chornobyl	C-9	2.1	3.2	0.5	5.8	50.8	254
	**ẋ ± sd**	**2.4 ± 1.6**	**2.2 ± 1.4**	**0.7 ± 0.5**	**5.4 ± 3.1**	**47.3 ± 27.5**	**124 ± 93**
N. Belarus	B-1	0.01	0.0039	nd	0.012	0.11	0.37
N. Belarus	B-2	0.02	0.0039	nd	0.021	0.18	0.73
N. Belarus	B-3	0.01	0.0016	nd	0.012	0.11	0.32
N. Belarus	B-4	0.03	0.0016	nd	0.031	0.27	0.96
N. Belarus	B-5	0.01	0.0016	nd	0.014	0.13	0.44
N. Belarus	B-6	0.01	0.0016	nd	0.015	0.13	0.45
N. Belarus	B-7	0.01	0.0016	nd	0.012	0.1	0.52
N. Belarus	B-8	0.003	0.0039	nd	0.007	0.06	0.12
N. Belarus	B-9	0.04	0.0039	nd	0.044	0.39	0.78
	**ẋ ± sd**	**0.02 ± 0.01**	**0.003 ± 0.001**	nd	**0.02 ± 0.01**	**0.15 ± 0.1**	**0.5 ± 0.3**

MOLECULAR ECOLOGY

Table S2. Pairwise Fst estimates for gray wolves from the CEZ and other global sites.

	Belarus	Chornobyl	Inner Mongolia	Tibet	Yellowstone
**Belarus**	0	0.0699	0.0951	0.467	0.165
**Chornobyl**	0.0699	0	0.12	0.505	0.193
**Inner Mongolia**	0.0951	0.12	0	0.448	0.141
**Tibet**	0.467	0.505	0.448	0	0.481
**Yellowstone**	0.165	0.193	0.141	0.481	0

MOLECULAR ECOLOGY

Table S3. F4-statistics estimated with ADMIXTOOLS2 for gray wolves from Chornobyl, Belarus, Tibet, and Inner Mongolia sites. Populations are defined as Bel = Belarus, Che = Chornobyl, Tib = Tibet, Mongolia = Inner Mongolia, Yel = Yellowstone.

pop1	pop2	pop3	pop4	F4	se	z-score	p.value
Mongolia	Bel	Che	Tib	-0.0061	0.00084	-7.28	3.28E-13
Bel	Che	Tib	Mongolia	0.00054	0.00066	0.82	0.41211
Bel	Che	Inn	Tib	-0.00054	0.00066	-0.82	0.41211
Tib	Che	Bel	Mongolia	-0.0061	0.00084	-7.28	3.28E-13
Tib	Che	Inn	Bel	0.0061	0.00084	7.28	3.28E-13
Tib	Mongolia	Bel	Che	0.00054	0.00066	0.82	0.41211
Bel	Mongolia	Che	Yel	0.0049	0.00055	8.92	< 2.22e-16
Mongolia	Bel	Che	Yel	-0.0049	0.00055	-8.92	< 2.22e-16
Mongolia	Bel	Che	Tib	-0.0061	0.00084	-7.28	3.28E-13

MOLECULAR ECOLOGY

Table S4. F3-statistics estimated ADMIXTOOLS2 for gray wolves across the European and Asian sites.

	pop1	pop2	pop3	est	se	z	p.value
1	Bel	Che	Inn	0.003	0.0006	4.66	3.2111E-06
2	Bel	Che	Tib	0.002	0.0007	2.82	0.0048614
3	Bel	Che	Yel	0.003	0.0006	4.3	1.7308E-05
4	Bel	Inn	Tib	0.009	0.0009	9.33	< 2.22e-16
5	Bel	Inn	Yel	0.008	0.0007	10.09	< 2.22e-16
6	Bel	Tib	Yel	0.008	0.001	7.42	1.1342E-13
7	Che	Inn	Bel	0.005	0.0005	9.79	< 2.22e-16
8	Che	Inn	Tib	0.011	0.0009	12.28	< 2.22e-16
9	Che	Inn	Yel	0.01	0.0007	14.62	< 2.22e-16
10	Che	Tib	Bel	0.005	0.0007	7.84	4.6071E-15
11	Che	Tib	Yel	0.01	0.0009	11.93	< 2.22e-16
12	Che	Yel	Bel	0.005	0.0005	10.02	< 2.22e-16
13	Inn	Bel	Che	0.008	0.0006	13.08	< 2.22e-16
14	Inn	Tib	Bel	0.002	0.0007	3.08	0.0020536
15	Inn	Tib	Che	0.002	0.0008	2.23	0.0259608

MOLECULAR ECOLOGY

Table S4. Sample Characterization. Sample list and total read count after all hemoglobin reads are removed.

Record ID	Sample ID	Total Read Counts After Filtering
GW1	GB1	1,641,543
GW2	GB2	1,484,711
GW3	GB3	1,543,677
GW4	GB4	2,284,750
GW5	GB5	1,449,870
GW6	GB6	1,102,196
GW7	GB7	1,985,224
GW8	GB8	1,910,768
GW9	GB9	2,280,966
GW10	GC1	6,023,331
GW11	GC2	4,000,795
GW12	GC3	4,913,137
GW13	GC4	5,706,451
GW14	GC5	3,411,996
GW15	GC6	2,446,392
GW16	GC7	2,399,040
GW17	GC8	7,036,018
GW18	GC9	12,106,597
GW19	GC10	5,581,606
GW20	GC11	5,039,175
GW21	SRR3402528	1,632,876
GW22	SRR3402525	5,328,403
GW23	SRR3402526	714,332
GW24	SRR3402485	2,332,801
GW25	SRR3402487	2,823,180
GW26	SRR3402489	1,587,035
GW27	SRR3402491	6,232,786
GW28	SRR3402492	3,452,188
GW29	SRR3402494	8,429,566
GW30	SRR3402495	7,135,189
GW31	SRR3402496	2,531,621
GW32	SRR3402498	2,133,581
GW33	SRR3402500	2,198,955

GW34	SRR3402502	2,408,168
GW35	SRR3402504	2,853,713
GW36	SRR3402506	5,754,053
GW37	SRR3402508	2,230,389
GW38	SRR3402510	2,965,087
GW39	SRR3402512	3,014,785
GW40	SRR3402514	2,557,491
GW41	SRR3402516	4,052,574
GW42	SRR3402518	8,313,364
GW43	SRR3402519	10,591,540
GW44	SRR3402520	6,396,615
GW45	SRR3402521	5,784,129
GW46	SRR3402523	7,188,119
GW47	SRR3402524	5,463,091

MOLECULAR ECOLOGY

Data S1. (separate file) WGCNA Module Gene Ontology Enrichment. Significant gene enrichment categories for modules identified through WGCNA and enriched with gprofiler2 using the wolf whole blood transcriptome for a custom background.

MOLECULAR ECOLOGY

Data S2. (separate file) Radiation-associated candidate genes Gene Ontology enrichment. Significant gene enrichment categories for top 359 genes showing correlation with total ^{90}Sr and ^{137}Cs dose rate of Chornobyl wolves. Enrichment characterized utilizing gprofiler2 and the wolf whole blood transcriptome as a customized background.

MOLECULAR ECOLOGY

Data S3. (separate file) Chornobyl Outlier Gene Ontology Enrichment. Significant gene enrichment categories for the 17 SNPs exhibiting significant divergence along the Chornobyl lineage.
